# Genome-wide analysis in over 1 million individuals of European ancestry yields improved polygenic risk scores for blood pressure traits

**DOI:** 10.1038/s41588-024-01714-w

**Published:** 2024-04-30

**Authors:** Jacob M. Keaton, Zoha Kamali, Tian Xie, Ahmad Vaez, Ariel Williams, Slavina B. Goleva, Alireza Ani, Evangelos Evangelou, Jacklyn N. Hellwege, Loic Yengo, William J. Young, Matthew Traylor, Ayush Giri, Zhili Zheng, Jian Zeng, Daniel I. Chasman, Andrew P. Morris, Mark J. Caulfield, Shih-Jen Hwang, Jaspal S. Kooner, David Conen, John R. Attia, Alanna C. Morrison, Ruth J. F. Loos, Kati Kristiansson, Reinhold Schmidt, Andrew A. Hicks, Peter P. Pramstaller, Christopher P. Nelson, Nilesh J. Samani, Lorenz Risch, Ulf Gyllensten, Olle Melander, Harriette Riese, James F. Wilson, Harry Campbell, Stephen S. Rich, Bruce M. Psaty, Yingchang Lu, Jerome I. Rotter, Xiuqing Guo, Kenneth M. Rice, Peter Vollenweider, Johan Sundström, Claudia Langenberg, Martin D. Tobin, Vilmantas Giedraitis, Jian’an Luan, Jaakko Tuomilehto, Zoltan Kutalik, Samuli Ripatti, Veikko Salomaa, Giorgia Girotto, Stella Trompet, J. Wouter Jukema, Pim van der Harst, Paul M. Ridker, Franco Giulianini, Veronique Vitart, Anuj Goel, Hugh Watkins, Sarah E. Harris, Ian J. Deary, Peter J. van der Most, Albertine J. Oldehinkel, Bernard D. Keavney, Caroline Hayward, Archie Campbell, Michael Boehnke, Laura J. Scott, Thibaud Boutin, Chrysovalanto Mamasoula, Marjo-Riitta Järvelin, Annette Peters, Christian Gieger, Edward G. Lakatta, Francesco Cucca, Jennie Hui, Paul Knekt, Stefan Enroth, Martin H. De Borst, Ozren Polašek, Maria Pina Concas, Eulalia Catamo, Massimiliano Cocca, Ruifang Li-Gao, Edith Hofer, Helena Schmidt, Beatrice Spedicati, Melanie Waldenberger, David P. Strachan, Maris Laan, Alexander Teumer, Marcus Dörr, Vilmundur Gudnason, James P. Cook, Daniela Ruggiero, Ivana Kolcic, Eric Boerwinkle, Michela Traglia, Terho Lehtimäki, Olli T. Raitakari, Andrew D. Johnson, Christopher Newton-Cheh, Morris J. Brown, Anna F. Dominiczak, Peter J. Sever, Neil Poulter, John C. Chambers, Roberto Elosua, David Siscovick, Tõnu Esko, Andres Metspalu, Rona J. Strawbridge, Markku Laakso, Anders Hamsten, Jouke-Jan Hottenga, Eco de Geus, Andrew D. Morris, Colin N. A. Palmer, Ilja M. Nolte, Yuri Milaneschi, Jonathan Marten, Alan Wright, Eleftheria Zeggini, Joanna M. M. Howson, Christopher J. O’Donnell, Tim Spector, Mike A. Nalls, Eleanor M. Simonsick, Yongmei Liu, Cornelia M. van Duijn, Adam S. Butterworth, John N. Danesh, Cristina Menni, Nicholas J. Wareham, Kay-Tee Khaw, Yan V. Sun, Peter W. F. Wilson, Kelly Cho, Peter M. Visscher, Joshua C. Denny, Million Veteran Program, Lifelines Cohort Study, CHARGE consortium, Cornelia M. van Duijn, ICBP Consortium, Adam S. Butterworth, Ahmad Vaez, Alexander Teumer, Andrew D. Johnson, Andrew D. Morris, Annette Peters, Anuj Goel, Archie Campbell, Bernard D. Keavney, Caroline Hayward, Christopher Newton-Cheh, Christopher P. Nelson, Daniel I. Chasman, Daniel Levy, Daniela Ruggiero, Eco de Geus, Edith Hofer, Eleftheria Zeggini, Eric Boerwinkle, Giorgia Girotto, Helen R. Warren, Hugh Watkins, Ivana Kolcic, J. Wouter Jukema, Jennie Hui, Joanna M. M. Howson, Johan Sundström, John C. Chambers, John N. Danesh, Lorenz Risch, Mark J. Caulfield, Markku Laakso, Martin D. Tobin, Martin H. De Borst, Melanie Waldenberger, Nilesh J. Samani, Olle Melander, Olli T. Raitakari, Ozren Polašek, Patricia B. Munroe, Paul M. Ridker, Pim van der Harst, Roberto Elosua, Samuli Ripatti, Terho Lehtimäki, William J. Young, Zoha Kamali, Zoltan Kutalik, Daniel Levy, Todd L. Edwards, Patricia B. Munroe, Harold Snieder, Helen R. Warren

**Affiliations:** 1 Center for Precision Health Research, National Human Genome Research Institute, National Institutes of Health, Bethesda, MD USA; 2 Division of Epidemiology, Department of Medicine, Vanderbilt University Medical Center, Nashville, TN USA; 3 Department of Epidemiology, University of Groningen, University Medical Center Groningen, Groningen, the Netherlands; 4 Department of Bioinformatics, Isfahan University of Medical Sciences, Isfahan, Iran; 5 Department of Epidemiology and Biostatistics, Imperial College London, London, UK; 6 Department of Hygiene and Epidemiology, University of Ioannina Medical School, Ioannina, Greece; 7 Department of Biomedical Research, Institute of Molecular Biology and Biotechnology, Foundation for Research and Technology-Hellas, Ioannina, Greece; 8 Division of Genetic Medicine, Department of Medicine, Vanderbilt University Medical Center, Nashville, TN USA; 9 Vanderbilt Genetics Institute, Vanderbilt University Medical Center, Nashville, TN USA; 10 Biomedical Laboratory Research and Development, Tennessee Valley Healthcare System (626)/Vanderbilt University, Nashville, TN USA; 11 Institute for Molecular Bioscience, University of Queensland, Brisbane, Queensland Australia; 12 Clinical Pharmacology, William Harvey Research Institute, Barts and The London School of Medicine and Dentistry, Queen Mary University of London, London, UK; 13 Barts Heart Centre, St Bartholomew’s Hospital, Barts Health NHS Trust, London, UK; 14 Department of Genetics, Novo Nordisk Research Centre Oxford, Oxford, UK; 15 Division of Quantitative Sciences, Department of Obstetrics and Gynecology, Vanderbilt University Medical Center, Nashville, TN USA; 16 Medical and Population Genetics, Broad Institute of Harvard and MIT, Cambridge, MA USA; 17 Division of Preventive Medicine Brigham and Women’s Hospital, Boston, MA USA; 18 Harvard Medical School, Boston, MA USA; 19 Centre for Genetics and Genomics Versus Arthritis, Centre for Musculoskeletal Research, The University of Manchester, Manchester, UK; 20 NIHR Barts Cardiovascular Biomedical Research Centre, Barts and The London School of Medicine and Dentistry, Queen Mary University of London, London, UK; 21 Population Sciences Branch, NHLBI Framingham Heart Study, Framingham, MA USA; 22 Department of Biostatistics, Boston University, Boston, MA USA; 23 National Heart and Lung Institute, Imperial College London, London, UK; 24 Population Health Research Institute, McMaster University, Hamilton, Ontario, Canada; 25 Faculty of Health and Medicine, University of Newcastle, New Lambton Heights, Newcastle, New South Wales Australia; 26 Human Genetics Center, Department of Epidemiology, Human Genetics, and Environmental Sciences, School of Public Health, The University of Texas Health Science Center at Houston, Houston, TX USA; 27 The Charles Bronfman Institute for Personalized Medicine, Icahn School of Medicine at Mount Sinai, New York, NY USA; 28 The Mindich Child Health and Development Institute, Icahn School of Medicine at Mount Sinai, New York, NY USA; 29 Novo Nordisk Foundation Center for Basic Metabolic Research, Faculty of Health and Medical Sciences, University of Copenhagen, Copenhagen, Denmark; 30 Department of Public Health and Welfare, Finnish Institute for Health and Welfare, Helsinki, Finland; 31 Department of Neurology/Medical University Graz, Graz, Austria; 32 Institute for Biomedicine, Eurac Research, Bolzano, Italy; 33 University of Lübeck, Lübeck, Germany; 34 Department of Cardiovascular Sciences, University of Leicester, Leicester, UK; 35 NIHR Leicester Biomedical Research Centre, Leicester, UK; 36 Faculty of Medical Sciences, Private University of the Principality of Liechtenstein, Triesen, Liechtenstein; 37 Department of Laboratory Medicine, Dr. Risch Anstalt, Vaduz, Liechtenstein; 38 Immunology, Genetics and Pathology, Uppsala University, Uppsala, Sweden; 39 Department of Clinical Sciences Malmö, Lund University, Malmö, Sweden; 40 Department of Internal Medicine, Skåne University Hospital, Malmö, Sweden; 41 Interdisciplinary Center Psychopathology and Emotional Regulation (ICPE), Department of Psychiatry, University of Groningen, University Medical Center Groningen, Groningen, the Netherlands; 42 Centre for Global Health Research, Usher Institute, University of Edinburgh, Edinburgh, Scotland; 43 MRC Human Genetics Unit, Institute of Genetics and Cancer, University of Edinburgh, Edinburgh, Scotland; 44 Center for Public Health Genomics, University of Virginia, Charlottesville, VA USA; 45 Cardiovascular Health Research Unit, Departments of Medicine, Epidemiology, and Health Systems and Population Health, University of Washington, Seattle, WA USA; 46 Vanderbilt Genetic Institute, Division of Genetic Medicine, Vanderbilt University Medical Center, Nashville, TN USA; 47 The Institute for Translational Genomics and Population Sciences, Department of Pediatrics, The Lundquist Institute for Biomedical Innovation at Harbor-UCLA Medical Center, Torrance, CA USA; 48 Department of Biostatistics, University of Washington, Seattle, WA USA; 49 Department of Medicine, Internal Medicine, Lausanne University Hospital and University of Lausanne, Lausanne, Switzerland; 50 Department of Medical Sciences, Uppsala University, Uppsala, Sweden; 51 The George Institute for Global Health, University of New South Wales, Sydney, New South Wales Australia; 52 MRC Epidemiology Unit, University of Cambridge, Cambridge, UK; 53 Computational Medicine, Berlin Institute of Health (BIH) at Charité - Universitätsmedizin Berlin, Berlin, Germany; 54 Precision Healthcare University Research Institute, Queen Mary University of London, London, UK; 55 Department of Health Sciences, University of Leicester, Leicester, UK; 56 Leicester NIHR Respiratory Biomedical Research Centre, Glenfield Hospital, Leicester, UK; 57 Department of Public Health and Caring Sciences, Uppsala University, Uppsala, Sweden; 58 Department of Public Health, University of Helsinki, Helsinki, Finland; 59 Diabetes Research Group, King Abdulaziz University, Jeddah, Saudi Arabia; 60 Center for Primary Care and Public Health (Unisanté), University of Lausanne, Lausanne, Switzerland; 61 Swiss Institute of Bioinformatics, Lausanne, Switzerland; 62 Institute for Molecular Medicine Finland (FIMM), HiLIFE, University of Helsinki, Helsinki, Finland; 63 Department of Public Health, Faculty of Medicine, University of Helsinki, Helsinki, Finland; 64 Department of Medicine, Surgery and Health Sciences, University of Trieste, Trieste, Italy; 65 Institute for Maternal and Child Health - IRCCS, Burlo Garofolo, Trieste, Italy; 66 Department Internal Medicine, Section of Gerontology and Geriatrics, Leiden University Medical Center, Leiden, the Netherlands; 67 Department of Cardiology, Leiden University Medical Center, Leiden, the Netherlands; 68 Netherlands Heart Institute, Utrecht, the Netherlands; 69 Department of Cardiology, Division of Heart and Lungs, University Medical Center Utrecht, Utrecht, the Netherlands; 70 Department of Genetics, University of Groningen, University Medical Center Groningen, Groningen, the Netherlands; 71 Wellcome Centre for Human Genetics, University of Oxford, Oxford, UK; 72 Radcliffe Department of Medicine, University of Oxford, Oxford, UK; 73 Lothian Birth Cohorts Group, Department of Psychology, The University of Edinburgh, Edinburgh, UK; 74 Department of Psychiatry, University of Groningen, University Medical Center Groningen, Groningen, the Netherlands; 75 Division of Cardiovascular Sciences, Faculty of Biology, Medicine and Health, The University of Manchester, Manchester, UK; 76 Manchester Heart Institute, Manchester University NHS Foundation Trust, Manchester Academic Health Science Centre, Manchester, UK; 77 Centre for Genomic and Experimental Medicine, IGC, University of Edinburgh, Edinburgh, UK; 78 Usher Institute, University of Edinburgh, Edinburgh, UK; 79 Department of Biostatistics, Center for Statistical Genetics, University of Michigan, Ann Arbor, MI USA; 80 Population Health Sciences Institute, Newcastle University, Newcastle, UK; 81 Department of Epidemiology and Biostatistics, MRC-PHE Centre for Environment and Health, School of Public Health, Imperial College London, London, UK; 82 Center for Life Course Health Research, Faculty of Medicine, University of Oulu, Oulu, Finland; 83 Unit of Primary Health Care, Oulu University Hospital, OYS, Oulu, Finland; 84 Institute of Epidemiology, Helmholtz Zentrum München, German Research Center for Environmental Health, Neuherberg, Germany; 85 Lehrstuhl für Epidemiologie, Institut für Medizinische Informationsverarbeitung, Biometrie und Epidemiologie (IBE), Ludwig-Maximilians-Universität München, Neuherberg, Germany; 86 Research Unit of Molecular Epidemiology, Institute of Epidemiology, Helmholtz Zentrum München, German Research Center for Environmental Health, Neuherberg, Germany; 87 Laboratory of Cardiovascular Science, National Institute on Aging, National Institutes of Health, Baltimore, MD USA; 88 Institute of Genetic and Biomedical Research, National Research Council (CNR), Monserrato, Italy; 89 Busselton Population Medical Research Institute, Perth, Western Australia Australia; 90 School of Population and Global Health, The University of Western Australia, Nedlands, Western Australia Australia; 91 Population Health Unit, Department of Public Health and Welfare, Finnish Institute for Health and Welfare, Helsinki, Finland; 92 Department of Immunology, Genetics, and Pathology, Biomedical Center, Science for Life Laboratory (SciLifeLab) Uppsala, Uppsala University, Uppsala, Sweden; 93 Department of Medicine, Division of Nephrology, University Medical Center Groningen, University of Groningen, Groningen, the Netherlands; 94 University of Split School of Medicine, Split, Croatia; 95 Algebra University College, Zagreb, Croatia; 96 Department of Clinical Epidemiology, Leiden University Medical Center, Leiden, the Netherlands; 97 Clinical Division of Neurogeriatrics, Department of Neurology, Medical University of Graz, Graz, Austria; 98 Institute for Medical Informatics, Statistics and Documentation, Medical University of Graz, Graz, Austria; 99 Gottfried Schatz Research Center for Cell Signaling, Metabolism and Aging, Medical University of Graz, Graz, Austria; 100 German Center for Cardiovascular Research (DZHK), Partner Site Munich Heart Alliance, Munich, Germany; 101 Population Health Sciences Institute St George’s, University of London, London, UK; 102 Institute of Biomedicine and Translational Medicine, University of Tartu, Tartu, Estonia; 103 Institute for Community Medicine, University Medicine Greifswald, Greifswald, Germany; 104 DZHK (German Center for Cardiovascular Research), Partner Site Greifswald, Greifswald, Germany; 105 Department of Internal Medicine B, University Medicine Greifswald, Greifswald, Germany; 106 Icelandic Heart Association, Kopavogur, Iceland; 107 Faculty of Medicine, University of Iceland, Kopavogur, Iceland; 108 Department of Health Data Science, University of Liverpool, Liverpool, UK; 109 IRCCS Neuromed, Pozzilli, Italy; 110 Institute of Genetics and Biophysics - ‘A. Buzzati-Traverso’, National Research Council of Italy, Naples, Italy; 111 Department of Public Health, University of Split School of Medicine, Split, Croatia; 112 Human Genome Sequencing Center, Baylor College of Medicine, Houston, TX USA; 113 Division of Genetics and Cell Biology, San Raffaele Scientific Institute, Milan, Italy; 114 Department of Clinical Chemistry, Fimlab Laboratories, Tampere, Finland; 115 Department of Clinical Chemistry, Finnish Cardiovascular Research Center - Tampere, Faculty of Medicine and Health Technology, Tampere University, Tampere, Finland; 116 Centre for Population Health Research, University of Turku and Turku University Hospital, Turku, Finland; 117 Research Centre of Applied and Preventive Cardiovascular Medicine, University of Turku, Turku, Finland; 118 The Framingham Heart Study, Framingham, MA USA; 119 Cardiovascular Research Center and Center for Genomic Medicine, Massachusetts General Hospital, Boston, MA USA; 120 Institute of Cardiovascular and Medical Sciences, University of Glasgow, Glasgow, UK; 121 International Centre for Circulatory Health, Imperial College London, London, UK; 122 School of Public Health, Imperial College London, London, UK; 123 Lee Kong Chian School of Medicine, Nanyang Technological University Singapore, Singapore, Singapore; 124 Hospital del Mar Research Institute (IMIM), Barcelona, Spain; 125 CIBER Enfermedades Cardiovasculares (CIBERCV), Barcelona, Spain; 126 Faculty of Medicine, University of Vic-Central University of Catalonia (UVic-UCC), Vic, Spain; 127 New York Academy of Medicine, New York, NY USA; 128 Institute of Genomics, University of Tartu, Tartu, Estonia; 129 Institute of Health and Wellbeing, University of Glasgow, Glasgow, UK; 130 Health Data Research UK, Glasgow, UK; 131 Division of Cardiovascular Medicine, Department of Medicine, Karolinska Institutet, Stockholm, Sweden; 132 Institute of Clinical Medicine, Internal Medicine, University of Eastern Finland, Kuopio, Finland; 133 Kuopio University Hospital, Kuopio, Finland; 134 Department of Biological Psychology, Faculty of Behavioural and Movement Sciences, Vrije Universiteit, Amsterdam, the Netherlands; 135 Amsterdam Public Health Research Institute, Amsterdam University Medical Centre, Amsterdam, the Netherlands; 136 Data Science, University of Edinburgh, Edinburgh, UK; 137 Health Data Research UK, London, UK; 138 Population Health and Genomics, School of Medicine, University of Dundee, Dundee, UK; 139 Amsterdam UMC, Vrije Universiteit Amsterdam, Department of Psychiatry, Amsterdam Public Health Research Institute, Amsterdam, the Netherlands; 140 Institute of Translational Genomics, Helmholtz Zentrum München – German Research Center for Environmental Health, Neuherberg, Germany; 141 Technical University of Munich (TUM) and Klinikum Rechts der Isar, TUM School of Medicine, Munich, Germany; 142 British Heart Foundation Cardiovascular Epidemiology Unit, Department of Public Health and Primary Care, University of Cambridge, Cambridge, UK; 143 VA Boston Healthcare System, Brigham and Women’s Hospital, Harvard Medical School, Boston, MA USA; 144 Department of Twin Research, King’s College London, London, UK; 145 Center for Alzheimer’s and Related Dementias, NIA/NINDS, NIH, Bethesda, MD USA; 146 Laboratory of Neurogenetics, NIA, NIH, Bethesda, MD USA; 147 DataTecnica LLC, Washington, DC, USA; 148 Intramural Research Program, National Institute on Aging, National Institutes of Health, Baltimore, MD USA; 149 Division of Cardiology, Duke University School of Medicine, Durham, NC USA; 150 Nuffield Department of Population Health, Big Data Institute, University of Oxford, Oxford, UK; 151 British Heart Foundation Centre of Research Excellence, University of Cambridge, Cambridge, UK; 152 Health Data Research UK Cambridge, Wellcome Genome Campus and University of Cambridge, Cambridge, UK; 153 Victor Phillip Dahdaleh Heart and Lung Research Institute, University of Cambridge, Cambridge, UK; 154 National Institute for Health and Care Research Blood and Transplant Research Unit in Donor Health and Behaviour, University of Cambridge, Cambridge, UK; 155 Department of Human Genetics, The Wellcome Sanger Institute, Wellcome Genome Campus, Hinxton, UK; 156 Department of Twin Research and Genetic Epidemiology, London, UK; 157 Department of Public Health and Primary Care, University of Cambridge School of Clinical Medicine, Cambridge, UK; 158 Department of Epidemiology, Emory University Rollins School of Public Health, Atlanta, Georgia USA; 159 VA Atlanta Healthcare System, Decatur, GA USA; 160 Emory Clinical Cardiovascular Research Institute, Atlanta, GA USA; 161 Massachusetts Veterans Epidemiology Research and Information Center (MAVERIC), VA Boston Healthcare System, Boston, MA USA; 162 Division of Aging, Department of Medicine, Brigham and Women’s Hospital, Boston, MA USA; 163 Department of Medicine, Harvard Medical School, Boston, MA USA; 164 Cardiovascular Health Research Unit, Departments of Medicine and Epidemiology, University of Washington, Seattle, WA USA

**Keywords:** Hypertension, Metagenomics

## Abstract

Hypertension affects more than one billion people worldwide. Here we identify 113 novel loci, reporting a total of 2,103 independent genetic signals (*P* < 5 × 10^−8^) from the largest single-stage blood pressure (BP) genome-wide association study to date (*n* = 1,028,980 European individuals). These associations explain more than 60% of single nucleotide polymorphism-based BP heritability. Comparing top versus bottom deciles of polygenic risk scores (PRSs) reveals clinically meaningful differences in BP (16.9 mmHg systolic BP, 95% CI, 15.5–18.2 mmHg, *P* = 2.22 × 10^−126^) and more than a sevenfold higher odds of hypertension risk (odds ratio, 7.33; 95% CI, 5.54–9.70; *P* = 4.13 × 10^−44^) in an independent dataset. Adding PRS into hypertension-prediction models increased the area under the receiver operating characteristic curve (AUROC) from 0.791 (95% CI, 0.781–0.801) to 0.826 (95% CI, 0.817–0.836, ∆AUROC, 0.035, *P* = 1.98 × 10^−34^). We compare the 2,103 loci results in non-European ancestries and show significant PRS associations in a large African-American sample. Secondary analyses implicate 500 genes previously unreported for BP. Our study highlights the role of increasingly large genomic studies for precision health research.

## Abstract (web summary)

Genome-wide association analysis in over one million individuals of European ancestry identifies 2,103 independent genetic signals (including 113 new loci) associated with blood pressure traits.

## Main

Over 30% of adults worldwide have hypertension, which is a leading modifiable risk factor for cardiovascular disease and death^1–3^. Hypertension is defined by elevated levels of systolic BP (SBP) and/or diastolic BP (DBP). SBP, the maximal arterial pressure exerted as the heart is beating, continuously increases with older age, whereas DBP, the arterial pressure between heartbeats, gradually plateaus by mid-life. Pulse pressure (PP), defined as the difference between SBP and DBP, is an indicator of arterial stiffness. BP is highly heritable, and multiple genome-wide association studies (GWAS) have highlighted its complex, polygenic architecture^4–9^.

Two recent large-scale GWAS meta-analyses with over 750,000 participants of European descent^4,5^, incorporating available data from biobanks and consortia such as the UK Biobank (UKB), the International Consortium for Blood Pressure (ICBP) and the Million Veteran Program (MVP), identified more than 1,000 independent loci associated with BP. Results from these studies have been applied to fine-mapping and candidate gene prioritization follow-up studies to further investigate the underlying BP biology^10–12^. Experience from prior BP-GWAS reveals that an increase in sample size can result in an enriched catalog of BP-associated genetic loci as well as an increase in the proportion of inter-individual variation in BP explained by the lead variants.

In this study, we conducted a single-stage GWAS meta-analysis combining all available genetic data from the UKB, ICBP and MVP from the previous two papers, using their existing GWAS summary statistics data together with new data (*n* ~ 50,000) from Vanderbilt University’s biorepository of DNA linked to de-identified medical records (BioVU)^13^. We accumulated data from over one million individuals of European descent, the largest sample size to date in a single-stage GWAS for BP. The analysis was performed using ~7.5 million imputed single nucleotide polymorphisms (SNPs) with a minor allele frequency (MAF) > 1% as the contributing GWAS data focused on common variants.

Our goals were to identify novel BP variants, reveal new biology underlying BP and generate a new BP PRS. Herein, we report the discovery of 113 novel loci for BP traits. The large sample size and current statistical methods increased the SNP-based heritability (${h}_{{\rm{SNP}}}^{2}$) of BP traits explained by GWAS variants to >60%. We developed genome-wide BP PRSs and tested these for the prediction of BP traits and hypertension risk in two independent datasets of European and African-American ancestry individuals.

We also applied methods that leverage the statistical precision of the GWAS and independent reference data from cardiovascular tissues to infer relationships between BP traits and gene expression, and we observed evidence of association with BP biology of 500 previously unreported genes. Many of these genes are located in previously mapped regions of the genome but were not identified by nearest-gene annotations in the literature, allowing the scientific yield from BP genetic studies to advance from lists of loci to lists of genes. These analyses provide insights into both the extent to which regulatory effects mediate genetic associations with BP traits as well as a principled data-driven mapping of associated loci with linked biology. This knowledge can be used to identify potential drug targets, develop testable hypotheses in model systems and advance understanding of BP regulation at the level of tissues and systems.

## Results

**Fig. 1:**
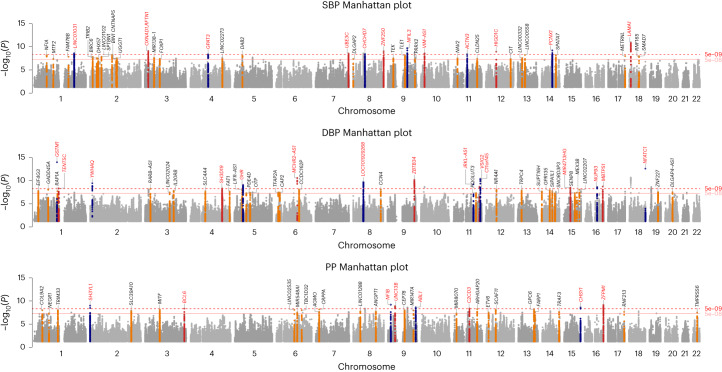
Manhattan plots of SBP, DBP and PP GWAS meta-analyses, illustrating 113 novel loci. Manhattan plots from top to bottom show novel results of SBP, DBP and PP GWAS meta-analysis, respectively, using inverse variance-weighted method. All loci are reported at genome-wide significance threshold (5 × 10^−8^). Annotated in red are loci reaching the more stringent *P* value of 5 × 10^−9^.

**Extended Data Fig. 1:**
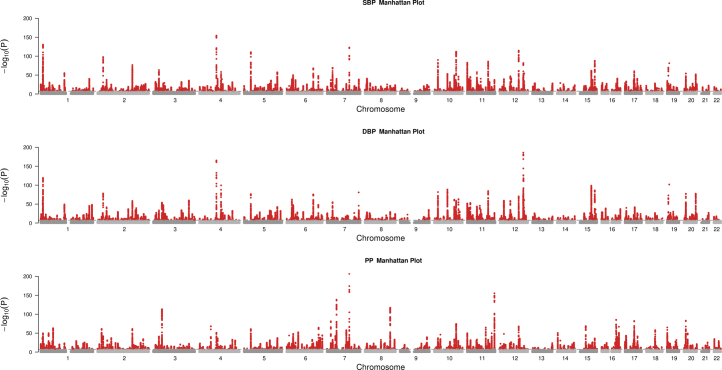
Manhattan plots of meta-analysis full results. Manhattan plots of meta-analysis full results using inverse variance-weighted method, showing 1,495, 1,504, and 1,318 significant loci for systolic (SBP, top plot), diastolic (DBP, middle plot), and pulse pressure (PP, bottom plot) in total (r^2^ < 0.05 and 1 Mb distance).

**Extended Data Fig. 2:**
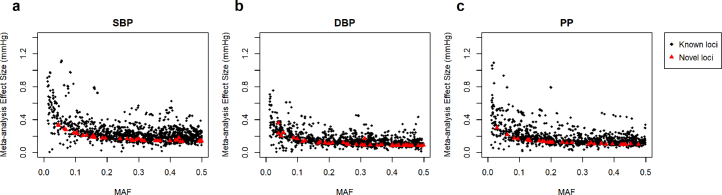
Comparison of the newly discovered loci with the known loci in effect size distribution. Comparison of the newly discovered loci with the known loci in effect size distribution, plotting Minor Allele Frequency (MAF) on the x-axis, vs GWAS effect estimate size on the y-axis, from the meta-analysis for SBP (a), DBP (b), PP (c).

Within our one-stage meta-analysis study of 7,584,058 SNPs in up to 1,028,980 individuals, there are a total of 1,495, 1,504 and 1,318 significant loci (*P* < 5 × 10^−8^) from the GWAS of SBP, DBP and PP, respectively (linkage disequilibrium (LD) *r*^2^ < 0.1 and 1 Mb distance; Extended Data Fig. 1). After excluding all known loci and their correlated variants in LD (LD *r*^2^ > 0.1 at ±500 kb) and applying clumping and LD-pruning methods to the remaining SNPs to identify independent loci ≥1 Mb apart and not in strong LD (*r*^2^ < 0.1), we detected sentinel SNPs indexing 113 novel loci for robust signficant association with at least one of the three continuous BP traits: (1) achieving genome-wide significance (*P* < 5 × 10^−8^) (Fig. 1 and Tables 1–3); (2) with consistent direction of effect in all available studies (Supplementary Table 1); and (3) no evidence of heterogeneity across studies (Tables 1–3 and Supplementary Figs. 1 and 2). Of these 113 novel loci, 35 reached a more stringent one-stage significance threshold of *P* < 5 × 10^−9^. Of all 113 novel loci (Supplementary Fig. 3), 40, 42 and 31 sentinel SNPs were significantly associated with SBP, DBP and PP, respectively, as the most significant trait with consistent effect direction. As in prior studies, the newly discovered loci had smaller effect sizes than previously reported SNPs, owing to the larger sample size and increased power to detect common variants with smaller effect sizes (Extended Data Fig. 2).

**Table 1:** 40 of the 113 novel loci (*P* < 5 × 10^−8^) identified with SBP as the primary trait

SNP	CHR:BP	Trait	Gene	A1	A2	EAF	Effect	s.e.	*P* value	*n*_eff_	*P*_het_
rs880132	18:7131618	SBP;DBP	*LAMA1*	C	T	0.573	−0.182	0.027	1.04 × 10^−11^	846,466	0.347
rs10991952	9:94252964	SBP;PP	*NFIL3*	G	A	0.299	0.169	0.027	1.98 × 10^−10^	978,737	0.641
rs36563	14:71352648	SBP	*PCNX1*	G	T	0.845	0.206	0.033	6.21 × 10^−10^	1,001,700	0.908
rs538180	3:16363689	SBP;DBP	*OXNAD1*	A	T	0.417	−0.151	0.025	8.47 × 10^−10^	989,900	0.833
rs2978398	8:146130326	SBP	*ZNF250*	A	G	0.423	−0.152	0.025	9.06 × 10^−10^	964,640	0.645
rs76637716	12:51355243	SBP;DBP	*HIGD1C*	A	G	0.055	−0.337	0.055	1.19 × 10^−9^	912,832	0.594
rs817140	1:193271526	SBP	*LINC01031*	C	T	0.276	0.161	0.027	2.52 × 10^−9^	995,667	0.558
rs10904910	10:17266389	SBP	*VIM-AS1*	A	C	0.31	0.155	0.026	2.56 × 10^−9^	996,726	0.481
rs2286130	7:156990554	SBP	*UBE3C*	T	C	0.241	−0.167	0.028	2.82 × 10^−9^	1,004,680	0.142
rs11988716	8:57153503	SBP	*CHCHD7*	G	A	0.134	0.211	0.036	3.62 × 10^−9^	975,452	0.84
rs61890399	11:66325484	SBP	*ACTN3*	C	T	0.104	−0.244	0.042	4.02 × 10^−9^	927,273	0.896
rs10018970	4:84452950	SBP	*GPAT3*	A	G	0.501	−0.142	0.024	4.24 × 10^−9^	995,118	0.322
rs9596839	13:54264395	SBP	*LINC00558*	A	G	0.291	−0.155	0.027	5.87 × 10^−9^	985,600	0.471
rs6729623	2:105205551	SBP	*LINC01102*	G	A	0.496	−0.141	0.024	5.88 × 10^−9^	984,559	0.319
rs7160184	14:88825415	SBP	*SPATA7*	T	C	0.094	−0.241	0.042	6.22 × 10^−9^	993,970	0.175
rs13162174	5:39444718	SBP	*DAB2*	T	G	0.601	−0.143	0.025	6.45 × 10^−9^	998,700	0.863
rs2224858	9:83432105	SBP	*TLE1*	G	A	0.815	0.179	0.031	7.37 × 10^−9^	1,006,540	0.092
rs2092867	1:61877445	SBP	*NFIA*	A	C	0.647	0.145	0.025	7.40 × 10^−9^	997,972	0.928
rs9675039	17:81036344	SBP	*METRNL*	A	G	0.367	0.146	0.026	1.07 × 10^−8^	953,745	0.372
rs72917789	18:46461487	SBP;PP	*SMAD7*	T	C	0.069	−0.277	0.049	1.14 × 10^−8^	973,426	0.329
rs17766830	18:44040660	SBP	*RNF165*	C	T	0.263	0.165	0.029	1.16 × 10^−8^	919,040	0.98
rs4573493	1:166023209	SBP	*FAM78B*	C	T	0.491	−0.138	0.024	1.42 × 10^−8^	976,312	0.204
rs75243511	2:54738168	SBP	*SPTBN1*	C	T	0.045	0.338	0.06	1.42 × 10^−8^	959,440	0.343
rs6723772	2:12994692	SBP	*TRIB2*	T	C	0.105	−0.227	0.04	1.55 × 10^−8^	965,181	0.383
rs9877020	3:43992455	SBP	*MIR138-1*	T	C	0.161	0.184	0.033	2.57 × 10^−8^	988,044	0.688
rs190533862	13:40671137	SBP	*LINC00332*	A	T	0.064	0.288	0.052	2.75 × 10^−8^	913,864	0.154
rs10172510	2:32620888	SBP	*BIRC6*	A	G	0.439	0.134	0.024	2.85 × 10^−8^	1,002,860	0.389
rs13022015	2:128822702	SBP	*UGGT1*	C	A	0.185	−0.173	0.031	3.08 × 10^−8^	981,303	0.417
rs9886857	9:27230388	SBP	*TEK*	A	G	0.151	−0.187	0.034	3.13 × 10^−8^	993,289	0.944
rs1319701	11:19736996	SBP	*NAV2*	G	T	0.497	0.134	0.024	3.19 × 10^−8^	984,376	0.499
rs11123059	2:125429006	SBP	*CNTNAP5*	A	G	0.568	0.134	0.024	3.47 × 10^−8^	994,840	0.954
rs56350535	2:39061959	SBP	*DHX57*	A	G	0.123	−0.207	0.038	3.85 × 10^−8^	955,006	0.953
rs7665985	4:153006312	SBP	*LINC02273*	C	T	0.641	−0.141	0.026	3.85 × 10^−8^	954,739	0.167
rs17542254	11:113655696	SBP	*CLDN25*	G	A	0.278	0.148	0.027	3.98 × 10^−8^	991,891	0.368
rs844218	3:71607861	SBP	*FOXP1*	G	A	0.688	−0.143	0.026	4.03 × 10^−8^	989,767	0.465
rs12145044	1:93524045	SBP	*MTF2*	G	T	0.047	−0.328	0.06	4.10 × 10^−8^	931,542	0.068
rs278123	12:120124578	SBP	*CIT*	A	G	0.318	0.142	0.026	4.37 × 10^−8^	996,302	0.3
rs11136373	8:1212030	SBP	*DLGAP2*	G	C	0.633	0.141	0.026	4.40 × 10^−8^	943,472	0.37
rs11690153	2:127839534	SBP	*BIN1*	C	T	0.194	0.174	0.032	4.48 × 10^−8^	925,702	0.381
rs3861882	9:132465304	SBP	*PRRX2*	C	T	0.281	−0.147	0.027	4.79 × 10^−8^	994,844	0.137

40 of the 113 novel loci (*P* < 5 × 10^−8^) with concordant direction of effect in all available studies after distance-based (±500 kb) and LD (*r*^2^ > 0.1) pruning, identified with SBP as the primary trait. SNPs are ordered by two-sided *P* value for the most significant BP association in inverse variance-weighted meta-analyses. SNP, dbSNP accession number; CHR:BP, chromosome and build 37 position; Trait, primary BP trait for which the most significant association was observed and for which summary statistics are provided in subsequent columns; for novel loci that reach genome-wide significance (*P* < 5 × 10^−8^) for a second trait, this second trait is also listed; Nearest Gene, most proximal gene within 250 kb of sentinel SNP; A1, allele corresponding to measured effect on the outcome; A2, allele not corresponding to measured effect on the outcome; EAF, effect allele frequency in the meta-analysis; Effect, measured effect in the meta-analysis (mmHg); s.e., standard error of the measured effect in the meta-analysis; *P* value, association *P* value for the measured effect in the meta-analysis; *n*_eff_, effective number of subjects in the GWAS meta-analysis (calculated at study-level as *n* × SNP imputation quality INFO); *P*_het_, value for Cochran’s Q test of statistical heterogeneity in the GWAS meta-analysis.

**Table 2:** 42 of the 113 novel loci (*P* < 5 × 10^−8^) identified with DBP as the primary trait

SNP	CHR:BP	Trait	Gene	A1	A2	EAF	Effect	s.e.	*P* value	*n*_eff_	*P*_het_
rs36209093	1:110229787	DBP	*GSTM1*	T	C	0.688	0.17	0.022	9.94 × 10^−15^	566,609	0.64
rs117777118	18:77161324	DBP; SBP	*NFATC1*	A	G	0.04	−0.358	0.049	2.40 × 10^−13^	636,875	0.446
rs57989773	6:100629078	DBP	*MCHR2-AS1*	C	T	0.245	−0.123	0.018	2.49 × 10^−11^	909,846	0.141
rs3765618	11:128769876	DBP	*C11orf45*	G	C	0.088	−0.18	0.027	3.87 × 10^−11^	974,839	0.266
rs10819246	9:129643296	DBP; SBP	*ZBTB34*	T	G	0.099	0.166	0.025	5.91 × 10^−11^	995,493	0.341
rs10087280	8:49391836	DBP; SBP	*LOC101929268*	G	A	0.171	−0.127	0.02	1.86 × 10^−10^	1,011,420	0.079
rs57503539	2:9803203	DBP	*YWHAQ*	A	G	0.21	−0.118	0.019	3.42 × 10^−10^	968,278	0.988
rs61909958	11:96151677	DBP	*JRKL-AS1*	G	C	0.188	−0.123	0.02	6.11 × 10^−10^	941,830	0.165
rs62370646	5:42515027	DBP	*GHR*	C	A	0.188	−0.119	0.019	7.97 × 10^−10^	1,008,790	0.701
rs8056413	16:84082650	DBP	*MBTPS1*	T	G	0.599	−0.093	0.016	1.75 × 10^−9^	989,746	0.718
rs11604175	11:124619407	DBP	*VSIG2*	T	C	0.256	0.104	0.017	1.99 × 10^−9^	1,000,810	0.47
rs12919839	16:56859216	DBP	*NUP93*	T	C	0.286	−0.099	0.017	2.15 × 10^−9^	1,013,420	0.471
rs28490942	15:51559845	DBP	*MIR4713HG*	C	G	0.449	−0.089	0.015	3.25 × 10^−9^	1,015,690	0.524
rs7671332	4:152163489	DBP; SBP	*SH3D19*	C	T	0.039	0.233	0.04	4.27 × 10^−9^	969,793	0.803
rs6669446	1:118223275	DBP	*TENT5C*	C	T	0.421	−0.089	0.015	4.29 × 10^−9^	1,016,030	0.876
rs2306623	3:25424929	DBP	*RARB-AS1*	C	T	0.67	0.093	0.016	5.22 × 10^−9^	1,013,160	0.202
rs10889711	1:68143195	DBP	*GADD45A*	C	T	0.631	−0.091	0.016	6.57 × 10^−9^	1,000,190	0.628
rs172906	5:38616887	DBP	*LIFR-AS1*	C	A	0.558	0.095	0.016	7.13 × 10^−9^	853,173	0.837
rs1546722	6:109625797	DBP	*CCDC162P*	G	A	0.517	−0.087	0.015	7.46 × 10^−9^	1,017,590	0.35
rs7174977	15:94214587	DBP	*LINC02207*	T	A	0.637	0.091	0.016	8.15 × 10^−9^	993,989	0.598
rs1732235	12:52418075	DBP	*NR4A1*	C	T	0.498	−0.087	0.015	8.18 × 10^−9^	1,010,320	0.293
rs2774052	14:59900020	DBP	*GPR135*	G	A	0.543	0.087	0.015	1.07 × 10^−8^	1,007,010	0.517
rs56312513	13:38249726	DBP	*TRPC4*	A	C	0.261	0.098	0.017	1.12 × 10^−8^	1,007,860	0.013
rs2320590	1:21155195	DBP	*EIF4G3*	T	C	0.55	0.085	0.015	1.38 × 10^−8^	1,021,190	0.559
rs73231988	3:136692308	DBP	*IL20RB*	A	G	0.116	0.135	0.024	1.45 × 10^−8^	982,317	0.956
rs6822301	4:72002332	DBP	*SLC4A4*	G	A	0.198	0.108	0.019	1.73 × 10^−8^	978,454	0.493
rs565522	1:112261533	DBP	*RAP1A*	C	T	0.435	−0.086	0.015	1.74 × 10^−8^	986,922	0.857
rs6982341	8:134229535	DBP	*CCN4*	G	A	0.581	0.085	0.015	1.74 × 10^−8^	1,026,530	0.203
rs7350752	14:21841154	DBP	*SUPT16H*	A	G	0.123	−0.147	0.026	1.89 × 10^−8^	782,069	0.27
rs9685837	4:187818466	DBP	*FAT1*	A	G	0.307	−0.092	0.016	1.98 × 10^−8^	990,892	0.731
rs2125578	19:44746657	DBP	*ZNF227*	T	C	0.539	−0.083	0.015	2.70 × 10^−8^	1,022,260	0.556
rs146827176	20:35169916	DBP	*DLGAP4-AS1*	T	C	0.048	−0.205	0.037	2.75 × 10^−8^	940,533	0.727
rs9477605	6:10034452	DBP	*TFAP2A*	A	G	0.353	0.087	0.016	3.22 × 10^−8^	1,013,520	0.917
rs6805393	3:117492152	DBP	*LINC02024*	A	G	0.508	−0.083	0.015	3.27 × 10^−8^	1,021,160	0.244
rs9370995	6:17477425	DBP	*CAP2*	G	C	0.536	0.084	0.015	3.31 × 10^−8^	993,051	0.46
rs2041330	14:71874638	DBP	*SIPA1L1*	G	A	0.44	0.084	0.015	3.42 × 10^−8^	1,002,690	0.698
rs983353	15:82186535	DBP	*MEX3B*	G	A	0.302	0.091	0.017	3.65 × 10^−8^	995,889	0.217
rs34237622	5:76884661	DBP	*OTP*	A	G	0.164	−0.114	0.021	3.72 × 10^−8^	974,348	0.349
rs11212666	11:108350451	DBP	*POGLUT3*	T	A	0.413	0.085	0.016	4.39 × 10^−8^	966,213	0.971
rs2034879	15:72429989	DBP	*SENP8*	A	G	0.737	0.097	0.018	4.39 × 10^−8^	946,840	0.98
rs10061553	5:58352210	DBP	*PDE4D*	T	C	0.312	−0.089	0.016	4.60 × 10^−8^	1,002,160	0.953
rs12883344	14:84911548	DBP	*SNORD3P3*	A	C	0.399	0.083	0.015	4.94 × 10^−8^	1,019,700	0.507

42 of the 113 novel loci (*P* < 5 × 10^−8^) with concordant direction of effect in all available studies after distance-based (±500 kb) and LD (*r*^2^ > 0.1) pruning, identified with DBP as the primary trait. SNPs are ordered by two-sided *P* value for the most significant BP association in inverse variance-weighted meta-analyses. SNP, dbSNP accession number; CHR:BP, chromosome and build 37 position; Trait, primary BP trait for which the most significant association was observed and for which summary statistics are provided in subsequent columns; for novel loci which reach genome-wide significance (*P* < 5 × 10^−8^) for a second trait, this second trait is also listed; Nearest Gene, most proximal gene within 250 kb of sentinel SNP; A1, allele corresponding to measured effect on the outcome; A2, allele not corresponding to measured effect on the outcome; EAF, effect allele frequency in the meta-analysis; Effect, measured effect in the meta-analysis (mmHg); s.e., standard error of the measured effect in the meta-analysis; *P* value, association *P* value for the measured effect in the meta-analysis; *n*_eff_, effective number of subjects in the GWAS meta-analysis (calculated at study-level as *n* × SNP imputation quality INFO); *P*_het_, value for Cochran’s Q test of statistical heterogeneity in the GWAS meta-analysis.

**Table 3:** 31 of the 113 novel loci (*P* < 5 × 10^−8^) identified with PP as the primary trait

SNP	CHR:BP	Trait	Gene	A1	A2	EAF	Effect	s.e.	*P* value	*n*_eff_	*P*_het_
rs34361301	9:14535119	PP	*NFIB*	C	T	0.266	0.122	0.02	6.76 × 10^−10^	974,146	0.322
rs34139656	16:88534923	PP	*ZFPM1*	G	A	0.327	−0.117	0.019	7.30 × 10^−10^	939,836	0.819
rs300753	2:209622	PP	*SH3YL1*	T	C	0.548	0.106	0.017	1.06 × 10^−9^	975,060	0.487
rs61241090	9:35191014	PP	*UNC13B*	C	T	0.236	−0.123	0.02	1.40 × 10^−9^	991,997	0.658
rs116643984	15:101791212	PP	*CHSY1*	A	C	0.163	−0.143	0.024	2.45 × 10^−9^	954,926	0.806
rs2987903	9:133711263	PP	*ABL1*	A	G	0.128	−0.154	0.026	2.49 × 10^−9^	995,802	0.631
rs4944038	11:73783478	PP	*C2CD3*	T	A	0.477	−0.101	0.017	4.30 × 10^−9^	1,006,450	0.33
rs3821817	3:187456904	PP	*BCL6*	G	C	0.178	−0.135	0.023	4.59 × 10^−9^	944,591	0.274
rs77759442	11:110657616	PP	*ARHGAP20*	T	C	0.132	0.15	0.026	5.91 × 10^−9^	960,047	0.358
rs75177877	7:16117030	PP	*CRPPA*	T	C	0.172	0.136	0.024	7.02 × 10^−9^	941,294	0.169
rs62253186	3:69919744	PP	*MITF*	G	C	0.061	0.217	0.038	7.14 × 10^−9^	920,630	0.94
rs4517643	13:94417873	PP	*GPC6*	C	A	0.566	0.101	0.018	7.47 × 10^−9^	990,764	0.633
rs12828693	12:46385848	PP	*SCAF11*	T	C	0.205	0.125	0.022	8.20 × 10^−9^	970,335	0.806
rs4053778	6:85988429	PP	*LINC02535*	G	A	0.395	−0.103	0.018	8.67 × 10^−9^	968,001	0.332
rs71664847	1:115019239	PP	*TRIM33*	T	A	0.19	0.126	0.022	9.99 × 10^−9^	993,341	0.633
rs12134085	1:40763095	PP	*COL9A2*	T	C	0.198	−0.133	0.023	1.05 × 10^−8^	864,823	0.003
rs9320778	6:121258543	PP	*TBC1D32*	T	C	0.75	0.115	0.02	1.05 × 10^−8^	973,244	0.75
rs112324977	9:80751434	PP	*CEP78*	A	T	0.131	−0.147	0.026	1.07 × 10^−8^	992,833	0.763
rs72751391	9:122890934	PP	*MIR147A*	T	C	0.125	0.156	0.027	1.23 × 10^−8^	907,738	0.942
rs10208493	2:196590414	PP	*SLC39A10*	T	C	0.571	−0.099	0.017	1.36 × 10^−8^	986,727	0.186
rs2953937	8:34164285	PP	*LINC01288*	C	A	0.133	0.143	0.026	1.76 × 10^−8^	993,180	0.135
rs36036692	8:108319395	PP	*ANGPT1*	G	C	0.374	0.1	0.018	1.89 × 10^−8^	997,711	0.167
rs12943001	17:78238645	PP	*RNF213*	C	T	0.641	−0.111	0.02	2.29 × 10^−8^	830,757	0.064
rs67615620	7:15421023	PP	*AGMO*	C	T	0.199	0.122	0.022	2.32 × 10^−8^	973,513	0.463
rs9554446	13:98859019	PP	*FARP1*	A	T	0.095	0.166	0.03	2.32 × 10^−8^	974,068	0.586
rs9671694	14:103330144	PP	*TRAF3*	G	C	0.337	0.104	0.019	2.40 × 10^−8^	961,925	0.743
rs72943226	6:99548729	PP	*MIR548AI*	A	G	0.319	0.102	0.018	3.12 × 10^−8^	999,371	0.245
rs1062298	12:12045264	PP	*ETV6*	T	G	0.421	0.097	0.018	3.24 × 10^−8^	977,558	0.084
rs855791	22:37462936	PP	*TMPRSS6*	G	A	0.563	−0.096	0.017	3.24 × 10^−8^	996,826	0.861
rs12084868	1:72229240	PP	*NEGR1*	A	G	0.028	0.302	0.055	4.67 × 10^−8^	898,662	0.546
rs11022023	11:11793978	PP	*MIR8070*	A	G	0.083	−0.174	0.032	4.80 × 10^−8^	964,778	0.539

31 of the 113 novel loci (*P* < 5 × 10^−8^) with concordant direction of effect in all available studies after distance-based (±500 kb) and LD (*r*^2^ > 0.1) pruning, identified with PP as the primary trait. SNPs are ordered by two-sided *P* value for the most significant BP association in inverse variance-weighted meta-analyses. SNP, dbSNP accession number; CHR:BP, chromosome and build 37 position; Trait, primary BP trait for which the most significant association was observed and for which summary statistics are provided in subsequent columns; for novel loci which reach genome-wide significance (*P* < 5 × 10^−8^) for a second trait, this second trait is also listed; Nearest Gene, most proximal gene within 250 kb of sentinel SNP; A1, allele corresponding to measured effect on the outcome; A2, allele not corresponding to measured effect on the outcome; EAF, effect allele frequency in the meta-analysis; Effect, measured effect in the meta-analysis (mmHg); s.e., standard error of the measured effect in the meta-analysis; *P* value, association *P* value for the measured effect in the meta-analysis; *n*_eff_, effective number of subjects in the GWAS meta-analysis (calculated at study-level as *n* × SNP imputation quality INFO); *P*_het_, value for Cochran’s Q test of statistical heterogeneity in the GWAS meta-analysis.

### LD score regression intercepts

In our overall meta-analyses, genomic inflation factors (*λ*_GC_) were calculated and *λ*_GC_ values were 1.82, 1.76 and 1.70 for SBP, DBP and PP, respectively. We calculated the LD score regression (LDSR) intercepts in our overall GWAS meta-analysis data as well as in the GWAS data remaining after the exclusion of all known BP loci to evaluate whether inflation of our test statistics was a result of polygenicity or residual population substructure (Supplementary Table 2). Attenuation ratios^14^ in overall analyses were 0.0884, 0.0844 and 0.0794, while attenuation ratios in the novel partition of our results were 0.0996, 0.0722 and 0.1085 for SBP, DBP and PP, respectively. LDSR intercepts in overall analyses were 1.2254, 1.2037 and 1.1756, while intercepts in the novel partition of our results were 1.0931, 1.0624 and 1.0806 for SBP, DBP and PP, respectively. These LDSR intercepts and attenuation ratios suggest that any observed inflation in our data is caused primarily by polygenicity.

### Known loci

Using our data to assign all 3,800 SNPs previously reported for BP traits into loci resulted in the identification of 1,165 independent loci that were ≥1 Mb apart and not in strong LD (*r*^2^ < 0.1) with each other or with known BP loci (Supplementary Table 3). LD pruning resulted in 1,723 pairwise-independent genetic signals from known SNPs (Supplementary Table 4).

As many of these known SNPs were previously identified using data contained within our meta-analysis, we did not seek to provide any replication of these published SNPs, but we did use the opportunity provided by our large-scale meta-analysis to present up-to-date and accurate results for the significance and effect estimates of the BP associations of all these SNPs (Supplementary Tables 4–6). Considering the sentinel SNPs of the 1,165 independent known loci, 1,092 of these were covered in our GWAS data, and 963 (88%) of these exact SNPs or close proxies (*r*^2^ > 0.8 and <500 kb) reached genome-wide significance in our data and 1,017 (93%) reached genome-wide significance at the locus level (Supplementary Tables 3 and 6), with less significant SNPs corresponding to associations originally reported from analyses of non-European ancestry, exome-chip studies or non-standard analyses that are not main-effect BP-GWAS analyses. Of 298 previously reported SNPs unavailable in our data, 227 (76%) were identified in rare-variant, non-European ancestry and/or in gene–environment interaction analyses. MAF and effect sizes of previously reported SNPs in our meta-analyses are concordant with published results (Supplementary Figs. 4 and 5).

### Conditional analysis

Genome-wide conditional analysis of SBP, DBP and PP meta-analyses identified a total of 267 additional independent significant secondary SNPs reaching a significance threshold of *P* < 5 × 10^−8^ in the conditional joint model (Supplementary Table 7). Of the 267 SNPs, 203 secondary SNPs also reached *P* < 5 × 10^−8^ in our primary meta-analyses and 23 mapped to one of our 113 novel BP loci.

### GWAS results summary

In summary, we report 1,723 pairwise-independent genetic signals among SNPs previously published for BP, 113 genome-wide significant novel loci from our meta-analyses and 267 additional independent significant secondary SNPs from conditional analysis, yielding a total of 2,103 independent genetic signals across all three BP traits.

### Variance explained

**Extended Data Fig. 3:**
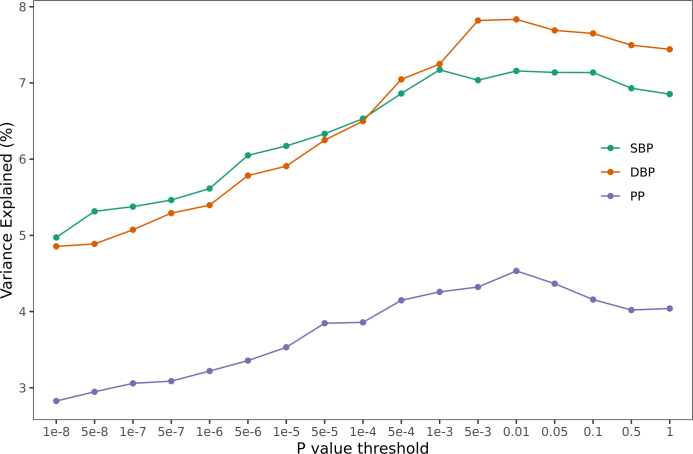
Variance explained by Polygenic Risk Scores (PRSs) at different P value thresholds. Variance explained by clumping and threshold Polygenic Risk Scores (PRSs) at different P value thresholds of inverse variance- weighted meta-analysis results, for SBP, DBP and PP, in the independent Lifelines cohort data.

Within the independent sample of 10,210 Lifelines participants (who were not included in the discovery GWAS), the genetic risk score (GRS) of our 113 novel loci explained a small but statistically significant proportion of BP variance: 0.06%, 0.08% and 0.02% for SBP, DBP and PP, respectively. Our findings contributed a small gain in the percentage of variance explained (%VE) for SBP, DBP and PP. For example, for SBP, the %VE by GRS increased from 6.77% for the 1,723 previously published SNPs to 6.80% after adding the 113 novel sentinel SNPs, and to 6.93% for all 2,103 independent BP genetic signals after also adding 267 independent secondary SNPs (Table 4). Furthermore, we first constructed a benchmark PRS based on the standard clumping and thresholding procedure for each BP trait (*P* value threshold, 1 × 10^−3^, 0.01 and 0.01 for SBP, DBP and PP, respectively). These PRSs captured a total of 7.17%, 7.83% and 4.53% of the variance in SBP, DBP and PP, respectively (Extended Data Fig. 3). Second, we calculated BP PRSs using SBayesRC^15^, which integrates GWAS data with functional genomic annotations and has been shown to have better prediction accuracy than other state-of-the-art PRS methods. We observed striking improvements in the percentages of variance explained by the SBayesRC PRS to 11.37%, 12.12% and 7.30% for SBP, DBP and PP, respectively (Table 4). The SBayesRC PRSs were used in all further PRS analyses in the Lifelines (European ancestry) and All-Of-Us (African ancestry) databases.

**Table 4:** Variance explained in SBP, DBP and PP for all four GRSs, the clumping and thresholding PRS and the SBayesRC PRS analyzed in an independent Lifelines dataset (*n* = 10,210) of European-descent individuals

Risk score	SBP		DBP		PP	
	VE (%)	*P* value	VE (%)	*P* value	VE (%)	*P* value
(1) All 1,723 known SNPs	6.77	3.60 × 10^−158^	6.77	8.52 × 10^−158^	4.29	5.96 × 10^−100^
(2) 113 novel sentinel SNPs	0.06	0.00927	0.08	0.00298	0.02	0.0741
(3) 1,723 known + 113 sentinel SNPs	6.80	6.67 × 10^−159^	6.83	3.48 × 10^−159^	4.29	7.05 × 10^−100^
(4) 1,723 known + 113 sentinel SNPs + 267 secondary SNPs	6.93	4.97 × 10^−162^	6.92	2.55 × 10^−161^	4.47	3.73 × 10^−104^
(5) Clumping and thresholding PRS	7.17	7.25 × 10^−168^	7.83	3.63 × 10^−183^	4.53	1.60 × 10^−105^
(6) SBayesRC PRS	11.37	6.34 × 10^−271^	12.12	1.06 × 10^−288^	7.30	4.17 × 10^−171^

GRS, genetic risk score; SBP, systolic blood pressure; DBP, diastolic blood pressure; PP, pulse pressure; VE, variance explained by the risk score for the respective BP trait expressed as a percentage; *P* value, two-sided association *P* value for the risk score with the respective blood pressure trait; PRS, polygenic risk score.

### Analyses of PRS in Lifelines

**Fig. 2:**
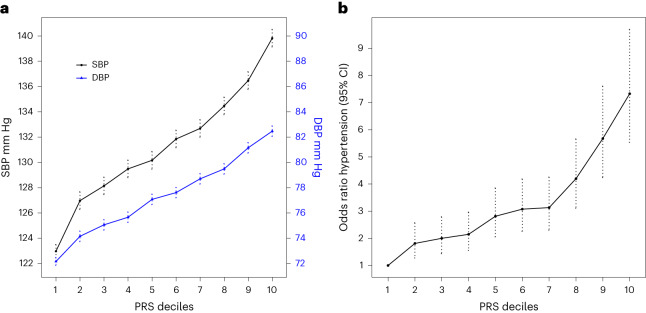
Relationship of deciles of the SBayesRC PRSs with SBP and DBP and risk of hypertension in European ancestry individuals from Lifelines cohort (*n* = 10,210). **a**,**b**, Plots show sex-adjusted SBP and DBP (**a**) and sex-adjusted odds ratios of hypertension (**b**) comparing each of the upper nine PRS deciles with the lowest decile. Dotted lines represent mean; error bars, s.e.m. in **a** and 95% CI in **b**.

**Extended Data Fig. 4:**
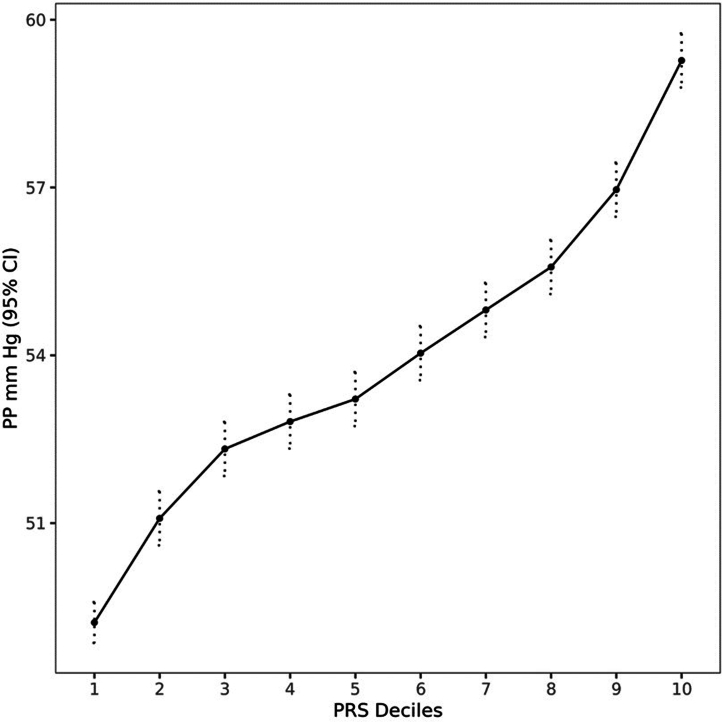
Relationship of deciles of the SBayesRC PRS with Pulse Pressure (PP) in Lifelines. Relationship of deciles of the SBayesRC PRS with Pulse Pressure (PP) in Lifelines of European ancestry (n = 10,210). Plot shows sex-adjusted mean PP comparing each of the upper nine PRS deciles with the lowest decile. Dotted lines represent 95% confidence intervals.

The SBayesRC PRSs showed sex-adjusted differences between top and bottom deciles of the PRS distribution of 16.9 mmHg for SBP (95% CI, 15.5–18.2 mmHg, *P* = 2.22 × 10^−126^), 10.3 mmHg for DBP (95% CI, 9.5–11.1 mmHg, *P* = 2.96 × 10^−130^) and 10.0 mmHg for PP (95% CI, 9.1–11.0 mmHg, *P* = 3.11 × 10^−94^) in 10,210 Lifelines participants. In addition, we observed more than a sevenfold higher sex-adjusted odds of hypertension (odds ratio (OR), 7.33, 95% CI, 5.54–9.70, *P* = 4.13 × 10^−44^) between the top and bottom deciles of the SBayesRC PRS in Lifelines when modeling both the SBP and DBP PRSs (Fig. 2, Extended Data Fig. 4 and Supplementary Table 8a). Alternatively, compared with middle deciles of the PRS distribution, individuals in the top decile had on average 8.82 mmHg higher SBP, 5.13 mmHg higher DBP, 5.64 mmHg higher PP and over twofold higher odds of hypertension (OR, 2.48) (Supplementary Table 8b).

### Hypertension model performance and calibration in Lifelines

**Extended Data Fig. 5:**
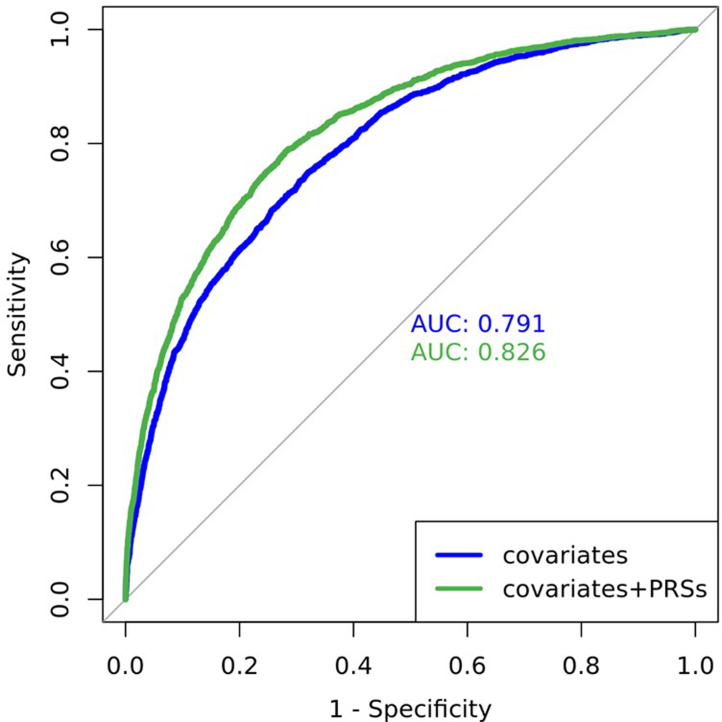
Area under the ROC curve of the two models for Hypertension prediction in Lifelines. Area under the ROC curve (AUROC) of the two models (covariates only and covariates plus SBayesRC PRS) for Hypertension prediction in Lifelines (n = 10,210) cohort of European ancestry.

The area under the receiver operating characteristic curve (AUROC) for model 1, which included only covariates, was 0.791 (95% CI, 0.781–0.801) and increased to 0.826 (95% CI, 0.817–0.836) for model 2, which included covariates as well as the SBP and DBP SBayesRC PRSs, a small but statistically significant difference of 0.035 (*P* = 1.98 × 10^−34^; Extended Data Fig. 5 and Supplementary Table 9a). Brier scores for model 1 (0.14) and model 2 (0.13) indicate that our models were reasonably well-calibrated. The Youden indices for model 1 and model 2 were 1.43 and 1.51, respectively, and correspond to the 58th and 60th percentile of the total sample. Hypertension prevalence in Lifelines was 23.6%. Addition of PRSs improved classification for a net of 4.72% of individuals (*n* = 114) with hypertension and 3.26% of individuals (*n* = 254) without hypertension (net reclassification index (NRI), 0.080, 95% CI, 0.063–0.097, *P* = 7.9 × 10^−22^; Supplementary Table 9b).

### Heritability in Lifelines

The GCTA-GREML^16^ SNP-based heritability (*h*^2^_SNP_) estimates in Lifelines data (*n* = 10,210) were 17.4%, 18.8% and 16.1% for SBP, DBP and PP, respectively. These GCTA-GREML^16^ *h*^2^_SNP_ estimates were used in the denominator of %VE / *h*^2^_SNP_ calculations, as both %VE and *h*^2^_SNP_ were derived from the same dataset. Hence, the total proportions of common SNP heritability that our GWAS explained, either for all 2,103 independent BP genetic signals combined or for the full clumping and thresholding PRSs capturing all genome-wide common SNP variation, were 39.8% (6.93% out of 17.4%) and 41.2% (7.17% out of 17.4%), respectively, for SBP, 36.8% (6.92% out of 18.8%) and 41.6% (7.83% out of 18.8%), respectively, for DBP and 27.8% (4.47% out of 16.1%) and 28.1% (4.53% out of 16.1%), respectively, for PP. Our improved PRSs using SBayesRC explained 65.4% (11.37% out of 17.4%), 64.5% (12.12% out of 18.8%) and 45.3% (7.30% out of 16.1%) of the common SNP heritability for SBP, DBP and PP, respectively.

### Association of BP variants in non-European ancestries

**Extended Data Fig. 6:**
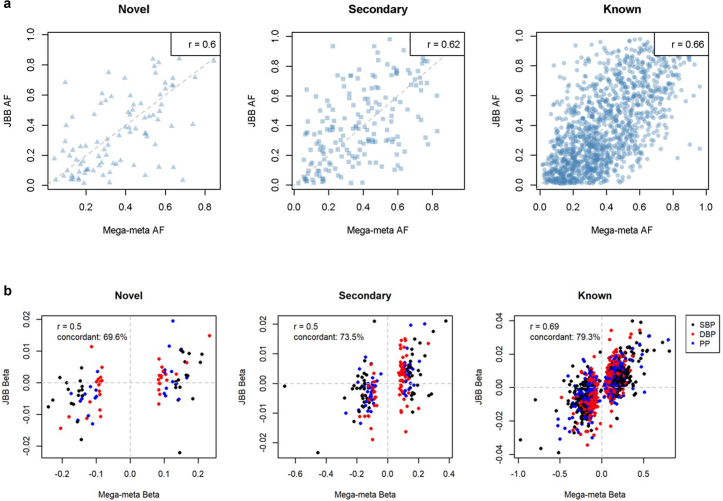
Pairwise allele frequency and effect size comparisons of 2103 GRS SNPs between our Mega-meta results and Japan Biobank. Pairwise allele frequency (a) and effect size (b) comparisons of 2103 GRS SNPs between our Mega-meta results and Japan Biobank (JBB) (n∼145k). Comparisons are separately made for the 113 novel SNPs (‘Novel’), 267 additional novel SNPs from conditional analysis (‘Secondary’), and 1723 known SNPs (‘Known’). Black, red and blue represent SNPs with SBP, DBP, and PP as the best associated traits, respectively. r = Pearson’s Correlation coefficient. ‘concordant’ means the proportion of SNPs showing directional concordance between European and Japanese populations. Please note that JBB effect sizes are standardized by Z-score transformation.

**Extended Data Fig. 7:**
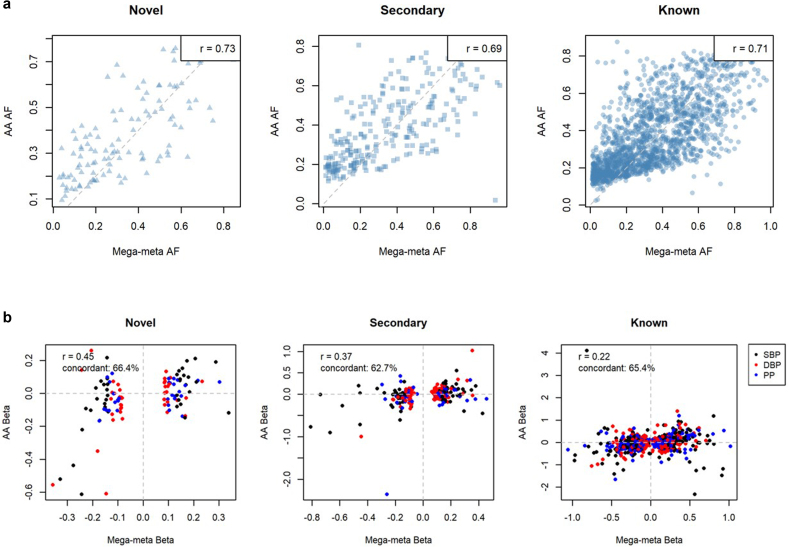
Pairwise allele frequency and effect size comparisons of 2103 GRS SNPs between our Mega-meta results and a meta-analysis of African-American ancestry individuals. Pairwise allele frequency (a) and effect size (b) comparisons of 2103 GRS SNPs between our Mega-meta results and a meta-analysis of African-American ancestry individuals (N = 83,890). Comparisons are separately made for the 113 novel SNPs (‘Novel’), 267 additional novel SNPs from conditional analysis (‘Secondary’), and 1723 known SNPs (‘Known’). Black, red and blue represent SNPs with SBP, DBP, and PP as the best associated traits, respectively. r = Pearson’s Correlation coefficient. ‘concordant’ means the proportion of SNPs showing directional concordance between European and African-American populations.

When comparing the distributions of allele frequency and effect sizes for the 2,103 independent BP-associated SNPs reported from our European meta-analysis within other ancestries, there was greater concordance within the Japanese population (Japan Biobank (JBB); *n* = 145,000, *r* = 0.69 and 0.5 correlation of effects, with 79% and 70% concordance in effect direction for known and novel SNPs, respectively) than within an African-ancestry meta-analysis sample (*n* = 83,890, *r* = 0.22 and 0.45 correlation, with 65% and 66% concordance for known and novel SNPs) (Extended Data Figs. 6 and 7 and Supplementary Table 10). Our novel loci showed weaker concordance than known loci for the Japanese comparisons but higher correlation than known loci for the African comparisons.

### PRS analyses in African-American ancestry

**Fig. 3:**
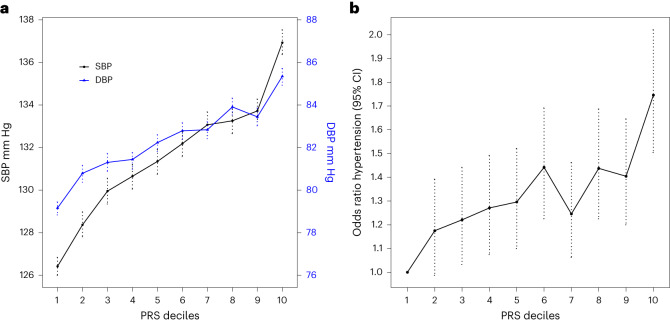
Relationship of deciles of the SBayesRC PRSs with SBP and DBP and risk of hypertension in African-American ancestry individuals from All-Of-Us cohort (*n* = 21,843). **a**,**b**, Plots show sex-adjusted mean SBP and DBP (**a**) and sex-adjusted odds ratios of hypertension (**b**) comparing each of the upper nine PRS deciles with the lowest decile. Dotted lines represent mean; error bars, s.e.m. in **a** and 95% CI in **b**.

**Extended Data Fig. 8:**
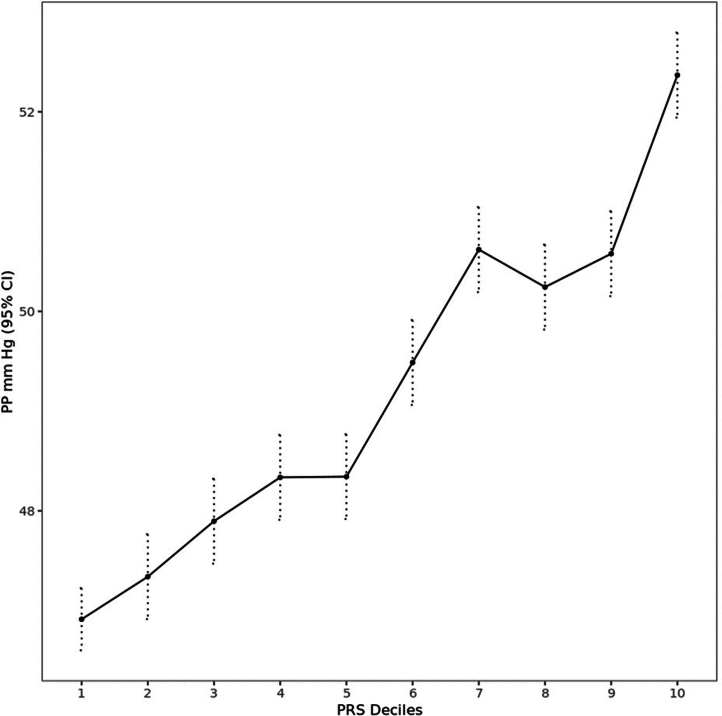
PRS for PP in AA. Relationship of deciles of the SBayesRC PRS with PP in African-American Ancestry individuals from All-Of-Us Cohort (n = 21,843). Plots show sex-adjusted mean PP comparing each of the upper nine PRS deciles with the lowest decile. Dotted lines represent 95% confidence intervals.

The SBayesRC PRS generated from our European meta-analysis is also associated with higher BP in an African-American ancestry sample (*n* = 21,843) from the All-Of-Us cohort: for example, with sex-adjusted differences between top and bottom deciles of the PRS distribution of 10.6 mmHg for SBP (95% CI, 9.4–11.8 mmHg, *P* = 1.20 × 10^−71^) and increased sex-adjusted odds of hypertension (OR, 1.73, 95% CI, 1.5–2.0, *P* = 2.33 × 10^−13^) (Fig. 3, Extended Data Fig. 8 and Supplementary Table 11). We observe a significant (*P* = 1.16 × 10^−5^) incremental increase in the AUROC from the covariate-only model (0.671; 95% CI, 0.666–0.680) to the model also including the PRS (0.676; 95% CI, 0.670–0.685) (Supplementary Table 12 and Supplementary Fig. 6). Of note, hypertension prevalence of 37% in the African-American subset of All-Of-Us is higher than in the European Lifelines cohort (Supplementary Table 13). The addition of the PRSs led to a non-significant reclassification result (NRI, 0.01, 95% CI, 0.006–0.021, *P* = 7.6 × 10^−2^), with only slight improvements in classification for a net of 0.22% of individuals (*n* = 49) with hypertension and 0.51% of individuals (*n* = 111) without hypertension (Supplementary Table 14).

### Variant functions of novel loci

More than 90% of the novel sentinel SNPs lie within non-coding regions (Supplementary Table 15). One novel sentinel SNP (rs855791) and seven highly correlated SNPs (*r*^2^ > 0.8) are non-synonymous variants in genes at six novel loci: *TMPRSS6*, *GLRX2*, *RLF*, *HELQ*, *ZNF235* and *UNC13B*; three of these non-synonymous SNPs reside in *UNC13B* (Supplementary Table 16).

### Overlap of novel loci across BP traits and with other traits

Across all 113 novel loci, we see concordance in the associations across the three BP traits (Supplementary Figs. 7 and 8), especially between SBP and DBP and between SBP and PP, which are known to be the more highly correlated BP trait pairs, so this is consistent with previous observations^4,5,7^. The Pearson correlation values for comparison of the effect estimates across all 113 novel loci are *r* = 0.82 for SBP vs DBP; *r* = 0.83 for SBP vs PP; and *r* = 0.37 for DBP vs PP. Nine of the 113 novel loci are genome-wide significant for a second BP trait in addition to their primary associated trait (as indicated in Tables 1–3).

Shared associations with at least one other disease trait reported within the GWAS Catalog or PhenoScanner database were observed for 41 out of the 113 novel loci; that is, sentinel SNPs and all SNPs in high LD (*r*^2^ > 0.8).

**Extended Data Fig. 9:**
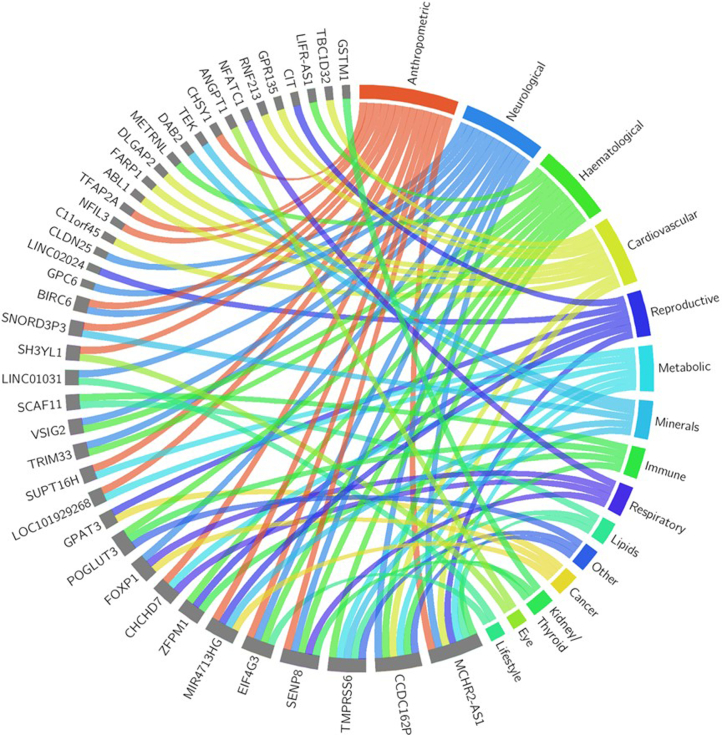
Cross-trait associations for 41 Blood Pressure novel loci with other diseases/traits. Cross-trait associations for 41 of the 113 Blood Pressure novel loci with other disease/trait categories from lookups within GWAS Catalog and Phenoscanner. Segment size depends on the number of locus-trait category associations.

The novel locus with the most shared associations was *MCHR2-AS1*, which has significant associations with seven disease or trait categories: anthropometric, reproductive, lipids, thyroid, cardiovascular, neurological and metabolic. Other loci showed associations with hematological traits (for example, hemoglobin, red blood cell count, white blood cell count, and so on), immune system (for example, inflammation, allergy, autoimmune, and so on), respiratory traits (for example, vital capacity, expiratory volume, expiratory flow, and so on) and minerals (for example, iron metabolism) (Extended Data Fig. 9 and Supplementary Table 17).

### Inferred gene expression and colocalization analysis

Applying S-PrediXcan analysis to infer the effects of genetically predicted gene expression on BP traits, we identified 5,538 statistically significant gene–tissue combinations that are genetically predictive of BP traits (Supplementary Table 18 and Supplementary Fig. 9). These combinations correspond to 1,873 unique genes, of which 569 (30%) have been identified by nearest-gene mapping of previously reported BP SNPs or novel sentinel SNPs identified in our meta-analyses. A total of 468 (25%) unique genes were previously identified in the equivalent S-PrediXcan and colocalization analyses^4^. We identified 1,029 (55%) unique genes in this analysis that have not previously been reported in BP-GWAS (Supplementary Table 18). The majority of associations were observed in arterial tissues (*n* = 1,503 for tibial artery; *n* = 1,205 for aorta). Associations were evenly distributed across all three BP traits (*n* = 1,851 for SBP; *n* = 1,962 for DBP; *n* = 1,725 for PP).

Additionally, we used COLOC to identify the subset of significant genes for which there was a high posterior probability that a SNP in the S-PrediXcan model for each gene exhibited colocalized association with both gene expression and changes in quantitative measures of BP traits. This analysis refined our S-PrediXcan analysis by characterizing the contribution of underlying expression quantitative trait loci (eQTLs) within our gene models to the observed S-PrediXcan associations. We detected 2,793 gene–tissue pairs in which there was a statistically significant S-PrediXcan association with at least one BP trait and high posterior probability (PP.H_4_ > 0.9) of colocalization, corresponding to a total of 1,070 distinct genes (642, 431 and 647 genes for SBP, DBP and PP, respectively). Of these 1,070 genes, 500 (47%) have not been previously annotated for SNP associations with BP traits.

### Druggable targets from transcriptome-wide association studies and colocalization results

We collated evidence for genes that mapped to our novel sentinel SNPs or mapped to our secondary SNPs but did not map from our primary GWAS or previous GWAS. We then found the intersection with genes that were significant in our inferred gene expression analyses and highlighted noteworthy examples (Table 5). We identified 38 genes satisfying this criterion, including an established drug target for BP medications (*ADRA1A*) and five genes targeted by other approved drugs (Supplementary Table 19).

**Table 5:** Prioritized genes through converging evidence across analyses

					TWAS^a^			
Gene	SNP	GWAS *P*_min_	GWAS Trait_min_	Prior TWAS	SBP	DBP	PP	DGI
*GSTM1*	rs36209093	9.94 × 10^−15^	DBP	No	-----	----↓	-----	-
*CASQ2*	rs4073778	5.00 × 10^−13^	PP	Yes	-----	-----	-↑^*^↑^*^↑^*^-	-
*MEF2D*	rs1185700	9.68 × 10^−12^	PP	No	-↑^*^---	-----	-↑^*^---	-
*BTN2A1*	rs2893856	3.00 × 10^−11^	DBP	No	----↑	----↑	-----	-
*MYL12A*	rs7811	1.14 × 10^−^^10^	PP	No	-----	-----	↓^*^↓^*^↓^*^--	-
*CCDC97*	rs56254331	1.18 × 10^−10^	DBP	No	-----	---↑-	-----	-
*CKB*	rs8017780	1.40 × 10^−10^	PP	No	-----	↓----	-----	-
*FOXN3*	rs7151849	1.77 × 10^−10^	PP	No				-
*ACTN4*	rs2303040	2.00 × 10^−10^	PP	No	-----	-----	↑^*^-↑^*^↑^*^-	-
*AMZ1*	rs798538	3.50 × 10^−10^	DBP	No	-----	--↑--	-----	-
*PCNX*	rs36563	6.21 × 10^−10^	SBP	No	↑↑^*^↑^*^--	-----	-----	-
*FUBP1*	rs750720	9.01 × 10^−10^	PP	No	-↑---	↑↑↑--	-----	-
*ADRA1A*	rs58623861	9.07 × 10^−10^	DBP	No	-↑---	-↑---	-----	¥
*GRB10*	rs79617314	1.36 × 10^−9^	PP	No	-----	↓----	↑^*^----	-
*NOTCH4*	rs2849017	1.78 × 10^−9^	DBP	Yes	↓^*^↓↓--	-----	↓^*^↓^*^↓↓^*^-	-
*ARID3B*	rs74781061	1.81 × 10^−9^	DBP	No	-----	-↓---	-----	-
*UBE3C*	rs2286130	2.82 × 10^−9^	SBP	No	-↓^*^---	-----	-↓^*^---	-
*FGFR2*	rs12255289	2.97 × 10^−9^	DBP	No	-----	↑^*^↑---	-----	¥
*LNPEP*	rs114772891	5.72 × 10^−9^	SBP	No	-↑^*^---	-↑^*^--↑^*^	-----	-
*TMEM51*	rs7553381	6.48 × 10^−9^	SBP	No	---↑-	-----	-----	-
*GPC6*	rs4517643	7.47 × 10^−9^	PP	No	-----	-----	↑^*^↑^*^↑^*^--	-
*SCAF11*	rs12828693	8.20 × 10^−9^	PP	No	-----	-----	----↓	-
*TRIM33*	rs71664847	9.99 × 10^−9^	PP	No	-----	-----	-↑^*^---	-
*COL9A2*	rs12134085	1.05 × 10^−8^	PP	Yes	-----	-----	↓^*^↓^*^---	-
*KLHL23*	rs78843689	1.08 × 10^−8^	DBP	No	-↓---	-----	-----	-
*RP11-460N16.1*	rs9868203	1.28 × 10^−8^	PP	No	-----	-----	-↓^*^---	-
*SLC39A10*	rs10208493	1.36 × 10^−8^	PP	No	-----	-----	-↓^*^↓^*^↓^*^↓^*^	-
*IL20RB*	rs73231988	1.45 × 10^−8^	DBP	No	↓↓---	↓----	-----	-
*CLIP2*	rs229872	2.08 × 10^−8^	DBP	No	-----	↑----	-----	-
*ABCC8*	rs77889556	2.91 × 10^−8^	PP	Yes	-----	-----	-↓^*^---	¥
*GTF2IRD1*	rs37613	3.08 × 10^−8^	SBP	No	↑^*^----	-----	-----	-
*SLC15A2*	rs9842387	3.20 × 10^−8^	SBP	No	↑^*^-↑^*^↑^*^-	-----	-----	¥
*DNAJC13*	rs2369796	3.32 × 10^−8^	DBP	No	-----	↑---↑	-----	-
*ANKH*	rs2921604	4.17 × 10^−8^	DBP	No	----↓	----↓	-----	-
*BIN1*	rs11690153	4.48 × 10^−8^	SBP	No	↑^*^---↑^*^	-----	-----	-
*PRRX2*	rs3861882	4.79 × 10^−8^	SBP	No	↑^*^----	-----	↑^*^↑^*^---	-
*NAGLU*	rs86312	4.94 × 10^−8^	SBP	No	-----	-----	↑^*^↑^*^---	¥

Table is sorted by minimum *P* value across all GWAS meta-analyses. Selection criteria: evidence from S-PrediXcan analysis and nearest-gene mapping of sentinel SNPs from GWAS meta-analysis. Gene, gene was significant in genetically predicted gene expression analysis using S-PrediXcan for aorta, tibial artery, left ventricle, atrial appendage and whole blood tissues and was annotated using ANNOVAR as the gene nearest the sentinel SNP at that locus. SNP, sentinel SNP from GWAS meta-analyses for each independent locus. GWAS *P*_min_, minimum *P* value across all inverse variance-weighted GWAS meta-analyses. GWAS Trait_min_, BP trait corresponding to the GWAS *P*_min_. Prior TWAS indicates whether the association was replicated in the previous S-PrediXcan analysis^4^ (where TWAS (transcriptome-wide association study) here refers to an inferred gene expression analysis using S-PrediXcan). TWAS indicates the direction of effect for significant associations in the SBP, DBP and PP S-PrediXcan analyses in aorta, tibial artery, left ventricle, atrial appendage and whole blood tissues, respectively; if the gene met the posterior probability threshold of ≥90% for colocalization of SBP, DBP and PP association and gene expression in aorta, tibial artery, left ventricle, atrial appendage and whole blood tissues, a small superscript (*) at the right of each arrow is shown. DGI, drug–gene interaction column summarizing if there are available drugs targeting genes that were identified (¥) according to the following databases: Guide to Pharmacology Interactions, DTC, DrugBank, JAX-CKB, My Cancer Genome, PharmGKB, Clearity Foundation Clinical Trials, TDG Clinical Trials, TALC, TTD, TEND and/or ChEMBL Interactions.

^a^Indicates whether gene expression was positively associated (↑), negatively associated (↓), or non-significant (−) in S-PrediXcan analyses.

### Pathway analyses

We input all 1,070 significant genes from S-PrediXcan and colocalization analyses into downstream enrichment analyses using FUMA^17^ (Supplementary Figs. 10–13 and Supplementary Tables 20–23). Results for tissue specificity were similar across all BP traits, with high enrichment in cardiovascular tissues (heart, arterial and whole blood), as expected, and in brain tissues of the central nervous system, given that hypertension associates with sympathetic nervous system activity. Enrichment in liver and pancreas tissues may be representative of the broader pleiotropy of BP genes and cardiometabolic diseases. The pathway analyses reveal a total of 4,617 unique significant terms (adjusted *P* < 0.05) across 20 different databases of functional annotations, boasting the complex biology of BP regulation. Some newly identified gene ontology annotations, not overlapping with pathway analysis results from previous BP studies, which are robustly reported across all BP trait input genes, include endoplasmic reticulum stress, carbohydrate and/or lipid metabolism, cell polarity, response to UV, DNA damage, autophagy, apoptotic mitochondrial envelop changes and (metal) ion transport.

## Discussion

In the largest single-stage common-variant GWAS of BP to date including more than one million European-ancestry adults, we report >2,000 independent BP signals from known and 113 novel loci as well as new secondary signals. The richness of results permitted the creation of PRSs that captured substantial interindividual variation in BP traits. These full PRSs are publicly accessible and can be used by the global research community to explore the contributions of BP to a variety of health outcomes.

This GWAS provides additional insights into the genetic contribution of BP and suggests that expansions of statistical power will continue to yield the discovery of additional loci primarily harboring common variants with smaller effect sizes, as has been recently achieved from GWAS of height^18^.

Our results demonstrate that the biology of BP is highly complex and polygenic, influenced by thousands of SNPs with extremely subtle effect sizes. In aggregate, these associations explain large differences in average BP and have a very strong influence on the risk of hypertension. Understanding the heritable influences on BP has the potential to provide foreknowledge of severe hypertension and its sequelae^19,20^. This study is, therefore, another key step toward understanding one of the most complex and highly regulated biological systems in humans that has significant implications for health, disease treatment and prevention.

We used a novel Bayesian method that fits genome-wide SNPs as random effects with a multi-component functionally informed prior for the PRS calculation^15^. These SBayesRC PRSs showed striking improvements in %VE for the different BP traits compared to the standard clumping and thresholding method, which includes only a subset of SNPs with ascertainment. For example, the SBayesRC PRS for SBP explained 65.4% of its common SNP-based heritability. This is more than double the 26.8% of the SBP *h*^2^_SNP_ explained and previously reported^5^. The remarkable improvement in the variance explained for all BP traits suggests a complex genetic architecture with common causal variants enriched in functionally important genomic regions. Even though we demonstrate that a large proportion of the genetic variance in BP is discoverable by GWAS, another gap remains between the common-variant-based heritability and the total pedigree-based h^2^ estimates that were recently reported to range from 25–30% for SBP, DBP and PP^21^. This gap is probably attributable to rare variants, as has been reported recently for height and body mass index (BMI) on the basis of whole genome sequencing data^22^. Rare variants associated with BP have been recently reported from separate large-scale exome-chip analyses^23^.

Application of the SBayesRC PRS in an external independent study (Lifelines), comparing top versus bottom deciles of the PRS distribution, demonstrated large BP differences; for example, 16.9 mmHg for SBP and 7.3-fold increased odds of hypertension. AUROC analyses indicated significant improvement in discrimination and calibration with the PRS included in the predictive model for hypertension. The observed negative predictive value of 91.6% for the full model Youden index cut-off demonstrates accurate discrimination of false negatives, an important goal in the classification of hypertension susceptibility. The improved performance of our PRS may allow for the identification of causal contributions of BP for many hypertension-related diseases. Furthermore, we found that the addition of the PRS to the model significantly improved the classification of hypertension. Nonetheless, the clinical utility of even our improved PRS will remain limited, given the uncertainty in individual PRS estimation for complex traits including hypertension as shown in a recent publication^24^.

In addition to mapping genomic locations, our pathway analyses also demonstrate the complexity of BP biology from the vast number of biological pathways enriched by BP genes. Furthermore, we show that many loci are associated with BP traits through regulatory effects on gene expression. We identified significant colocalized associations between BP traits and genetically predicted gene expression of 1,070 genes, 500 of which have not been identified in prior BP-GWAS. Of these 500 genes, 314 remain novel, at the time of submission, after updated searches within the GWAS Catalog and cross-referencing with a recently published list of prioritized BP genes from a post-GWAS candidate gene prioritization study^10^.

These new gene observations can provide opportunities for further experimentation in model systems and elucidate candidate targets for drug development or repurposing.

Among novel loci, *TMPRSS6* (rs855791; PP *P* = 3.20 × 10^−8^) is a promising candidate as a potential drug target. This gene, encoding transmembrane serine protease 6, has been implicated in the attenuation of dietary iron overload in heart tissue leading to cardioprotective effects^25,26^. Genetic variation at *TMPRSS6* is also associated with biomarkers of iron overload^27^. *SMAD7* (rs72917789; SBP *P* = 1.14 × 10^−8^) has been shown to modulate the expression of hepcidin, a key regulator of intestinal iron absorption^28,29^. Additionally, *GSTM1* (rs36209093; DBP *P* = 9.94 × 10^−15^), encoding glutathione *S*-transferase Mu 1, has been implicated in cardiomyopathy resulting from iron overload^30,31^. These results suggest that altered iron metabolism may have a role in BP regulation and hypertension-related cardiovascular disease and are consistent with previous studies linking high iron stores to cardiovascular disease^32^.

Evaluation of the intersection of inferred gene expression and colocalization results with novel and secondary loci highlights several genes targeted by approved medications or with compelling biological evidence supporting their role in BP physiology. *ADRA1A*, encoding the α−1-adrenergic receptor 1A, the product of which is a well-known target for medications treating both hypertension and hypotension^33^, was previously unreported in BP-GWAS. Considering our conditional analysis and inferred gene expression associations at this locus, *cis*-regulatory variants for *ADRA1* may affect the efficacy of targeted medications. *ABCC8*, an established diabetes GWAS locus^34^, the product of which is targeted by sulfonylurea medications^35,36^, harbors rare variants contributing to pulmonary arterial hypertension^37–39^. *FGFR2*, targeted by anti-angiogenesis medications in the treatment of cancer^40^, is involved in sexual dimorphism of the baroreflex afferent function on BP regulation in rats^41^ and has been implicated in parenchymal and vascular remodeling in pulmonary arterial hypertension^42^. These findings are biologically plausible, and the ADRA1A receptor protein is targeted to manipulate BP, demonstrating that our approach detects genes with biological and pharmacological impact. This suggests that additional genes from our analysis may be viable options for drug targeting and further study.

This study has several limitations. Owing to the large sample size, independent study samples to replicate our findings in a more traditional two-stage design are not readily available, so it is not possible to report loci with formal validation as has been done for previous two-stage BP-GWAS analyses. We have attempted to address this limitation by implementing robust reporting criteria appropriate for a single-stage discovery analysis, with rigorous post-quality-control (QC) filtering of the meta-analysis data, requiring full concordance in the direction of novel SNP effects across all four datasets in the meta-analysis in addition to no evidence of heterogeneity across these four datasets, and highlighting SNP results that meet a higher 5 × 10^−9^ significance threshold. Owing to the available GWAS datasets, our study is restricted to the analysis of common variants only with MAF > 1%, but it is important for future analyses to consider both common and rare variants, especially now with sample sizes exceeding one million individuals.

Although our discovery GWAS was limited to non-Hispanic white participants, we provide plots to illustrate the concordance of the effects of BP variants in Japanese and African individuals. As the levels of correlation vary between the comparisons with Japanese versus African ancestries and between novel versus known loci, it highlights the importance of further testing of BP variants derived from European studies within different non-European populations in the future, to clarify which genetic signals are shared and which may have ancestry-specific effects^43^.

We do show a significant association of our European-derived PRS with BP and hypertension in an African-American sample. However, the nominal increases in AUROC or NRI statistics when adding the PRS into hypertension-prediction models in African-American individuals shows that substantial studies that include individuals of non-European ancestry, or alternative methodological approaches^44^, are essential to understand ancestrally related disparities in hypertension, observations that mirror those for other complex traits^45,46^.

Our study results suggest that efforts should continue for future BP-GWAS to leverage large-scale biobank resources and cohort studies to expand the sample size further, as well as extending to diverse ancestries. The benefits of this approach may include improved homogeneity of associations if the data are collected under uniform conditions, as in the UKB^47^. Our data also show high concordance in GWAS results between studies of different designs (Supplementary Fig. 14), supporting a continuing role for the inclusion of large electronic health record (EHR)-derived studies within meta-analysis projects. Future studies should also continue to evaluate associations with genetically predicted gene expression to stimulate other avenues of investigation. These goals, if accomplished, will provide researchers with translational knowledge to mitigate disparities and reduce the global impact of health outcomes for which hypertension is a highly common risk factor.

## Methods

We conducted a single-stage BP-GWAS meta-analysis of individuals of European ancestry, evaluating common SNPs, as the GWAS summary statistics data used had already previously been filtered to MAF ≥ 1%. SBP, DBP and PP GWAS summary statistics from each study were obtained from linear regression models analyzing SNP associations adjusted for age at BP measurement, age^2^, sex, BMI and the top ten genetic principal components. Inferences were limited to SNPs with imputation quality (INFO) scores of 0.1 or higher, Hardy–Weinberg equilibrium *P* values of ≥1 × 10^−6^ and MAF ≥ 1%. PP was calculated in each study as the difference between SBP and DBP.

### Study populations

The total sample size for this investigation was up to 1,028,980 adults from the meta-analysis of four existing BP-GWAS datasets: UKB, ICBP, MVP and BioVU. Characteristics of these studies are presented in Supplementary Table 24. We acknowledge the different demographics of MVP, being predominantly male (only 7.1% female compared to 58.4% and 54.2% for BioVU and UKB, respectively), and note the higher proportion of individuals taking anti-hypertensive medication (48.9% and 59.5% for MVP and BioVU, respectively, compared to only 20.6% for UKB) probably because the data were drawn from EHR data within a clinical environment. ICBP is a large meta-analysis of 77 studies; therefore, descriptive characteristics were not available. More detailed information on study populations is provided in the Supplementary Notes.

### Study-level QC

We applied a harmonized QC procedure for each BP trait in all four studies (that is, 12 GWAS datasets in total) using the *GWASInspector* R package^48^. The 1000 Genomes Project reference panel^49^, supplemented with the Haplotype Reference Consortium data panel^50–53^, was used as the reference dataset for appropriate flipping and/or switching of the alleles, checking for allele frequency concordance with the 1000 Genomes reference, annotating dbSNP rs accession numbers and constructing harmonized identifiers for meta-analyses. Allele frequency differences between the reference and individual GWAS data were not used for filtering the variants unless an unexplained off-diagonal cross line could be distinguished in the correlation scatterplot. In this case, we used a difference of 0.25 between the reference and individual GWAS data as the cut-off to filter out variants with seemingly flipped alleles. This was the case for only a very small number of variants within the MVP cohort, requiring the removal of about 12,000 SNPs (<0.15% of the data). SNP effect sizes from ICBP were considered as the reference to validate the reported effect sizes from the other three GWAS datasets (Supplementary Figs. 15–17)^7,54^.

The following criteria were then used for filtering the GWAS datasets: (1) SNPs only (that is, no insertions or deletions, copy number variants, and so forth); (2) MAF ≥ 1%; (3) INFO scores greater than 0.1; (4) Hardy–Weinberg equilibrium *P* ≥ 1 × 10^−6^. Effective sample size was calculated as the product of the total sample size and INFO for each SNP.

### Meta-analysis

We initially applied LDSR^14^ to the summary statistics for three of our four component datasets (UKB, MVP and BioVU) to calculate the LDSR intercepts that were used to correct for pre-meta-analysis genomic inflation. ICBP summary statistics, as a meta-analysis of 77 independent cohorts, were previously corrected for genomic inflation^5^. HapMap3 (ref. ^55^) SNP alleles and pre-calculated LD scores from 1000 Genomes Project^49^ European reference data supplied with the package were used to calculate LDSR intercepts. Observed LDSR intercepts for SBP, DBP and PP, respectively were as follows for each dataset: 1.2177, 1.2195 and 1.1851 for UKB; 1.0530, 1.0247 and 1.0413 for MVP; and 1.0288, 1.0127 and 1.0207 for BioVU. Inverse variance-weighted fixed-effects meta-analysis of common (MAF ≥ 0.01) bi-allelic SNPs with INFO scores greater than or equal to 0.1 across our four studies was performed using METAL^56^ software. No further GC correction was applied to the meta-analysis results, which combined our four datasets.

### QC of the meta-analysis results

Similar to study-level QC, we used the *GWASInspector* R package^48^ to ensure standardization and perform QC of post-meta-analysis summary statistics. Analyses included checks of allele frequency concordance with the 1000 Genomes reference and concordance of effect sizes with ICBP (Supplementary Fig. 18) as well as evaluation of *Q–Q* plots and genomic inflation factors (Supplementary Fig. 18) and evaluation of bivariate scatterplots of key summary statistics to identify patterns indicating the presence of low-quality SNPs (Supplementary Fig. 19).

These analyses revealed the presence of SNPs in our data with low effective sample sizes and large standard errors as well as a sub-peak of SNPs with higher effective sample sizes and large standard errors. Based on these observations, we applied a filtering threshold for SNPs that were present in at least three of our four studies or SNPs that reached an effective sample size greater than or equal to 60% of the maximum (Supplementary Figs. 20–22). Application of these criteria to achieve an optimal balance between the quality of retained SNPs and sample size resulted in 7,584,058 SNPs available for analysis.

### Distinguishing known from novel loci

#### Published BP SNPs

We collated published BP-GWAS and compiled all 3,800 unique BP SNPs reported to date (Supplementary Tables 5 and 25). In many BP-GWAS papers, the list of previously reported BP variants has focused on the lead sentinel variant, with validated evidence from independent replication. To expand to a fully comprehensive list of known variants, we curated a list of all published common and rare variants, including results from studies conducted in non-European ancestries, all types of methodological analyses including interaction analyses, results from both one-stage and two-stage study designs, and secondary variants reported from conditional or fine-mapping analyses. We began with the list of all 984 SNPs from the total of 901 previously known and novel loci previously reported^5^, then added (1) any secondary SNPs reported from conditional analyses in publications up to 2018 (refs. ^5,7,9,57^); (2) SNPs reported from a large one-stage discovery analysis before 2018 (ref. ^8^); (3) SNPs reported in a previous publication from 2019 (ref. ^4^) and all other SNPs from GWAS published between 2018 and the end of 2020 (refs. ^23,58–63^). We removed duplicated SNPs to generate a unique set of ~3,800 SNPs. Subsequent checks of our results in GWAS Catalog^64^ and PhenoScanner^65^ confirmed that all published BP variants had been successfully captured. For QC purposes, we compared the allele frequencies and the resulting effect estimates of these published SNPs in our GWAS meta-analysis data with the published data.

#### LD analyses

LD was calculated using PLINK-2 (ref. ^66^) with 1000 Genomes Project^49^ phase 3 version 5 European reference genotypes. LD proxies were captured for the ~3,800 previously reported BP SNPs at an *r*^2^ threshold of >0.8 and a maximum distance of 500 kb. Furthermore, we identified the most strongly associated SNP within 500 kb of each known SNP regardless of LD (that is, ‘distance proxies’). The strongest trait-specific associations of these previously reported SNPs, their best LD proxies and best distance proxies in our meta-analyses are presented in Supplementary Table 6.

We partitioned our data into known and unknown subsets. To identify the ‘unknown’ portion of our GWAS results, we removed previously reported SNPs, SNPs within 500 kb of previously reported SNPs, LD proxies for previously reported SNPs at an *r*^2^ threshold of >0.1 and a maximum distance of 5 Mb, and SNPs within the human leukocyte antigen region of chromosome 6 (25–34 Mb) from each of our meta-analyses. *Q–Q* plots of all SNPs versus unknown SNPs are shown in Supplementary Fig. 23.

#### Reporting criteria for novel loci

All remaining SNPs reaching genome-wide significance (*P* < 5 × 10^−8^) and consistent direction of effect in all available studies were clumped into 1 Mb regions, and the most significant SNP for any trait was selected from each region as a sentinel variant for the locus. Novel sentinel SNPs were checked for pairwise LD against all other novel sentinel SNPs at an *r*^2^ > 0.1 to confirm independence. Considering our one-stage study design, we imposed two additional stringent reporting criteria in addition to achieving genome-wide significance. To declare a novel sentinel SNP, we required genome-wide significance *P* < 5 × 10^−8^ in the meta-analysis; consistent direction of effect across all the available sub-datasets; and no evidence of heterogeneity across the four datasets with heterogeneity *P* < 1 × 10^−4^. We also highlight how many of these novel loci reach a stricter significance threshold of *P* < 5 × 10^−9^.

#### Categorizing known variants into independent loci

Similarly, previously reported SNPs, their best LD proxy if the SNP was unavailable in our data or the best distance proxy if neither was available, were clumped into 1 Mb regions and the most significant SNP for any trait was selected. Selected SNPs were then checked for pairwise LD against all other selected SNPs at an *r*^2^ > 0.1 to confirm independence. The most significant SNP for any trait was selected within each LD block, and these independent SNPs were designated as known sentinel SNPs.

### LDSR approach for determination of polygenicity

We applied LDSR to each of our three meta-analyses (SBP, DBP and PP) as well as the novel proportion of each meta-analysis and compared these values with genomic inflation factors to determine whether inflation of our test statistics was a result of population substructure or polygenicity.

### Functional annotation and associations of novel loci

Novel signals were extended to their correlated variants in LD (*r*^2^ > 0.5) using an in silico sequencing approach^67^. PLINK^66^ was used for LD calculations and ANNOVAR^68^ software was used to annotate the nearest genes for novel signals and to annotate variant functions. Then the extended loci (*r*^2^ > 0.8) were used to search the GWAS Catalog^64^ as well as PhenoScanner^65^ for shared associations (*P* < 5 × 10^−8^).

### Conditional analysis

Genome-wide joint conditional analysis was performed using GCTA-COJO v1.93 (ref. ^69^), specifying a 5 Mb LD window and a genome-wide significance threshold of 5 × 10^−8^ and using UKB European-ancestry sample genotypes as the LD reference. For each of our three BP traits, summary statistics were analyzed by chromosome to build a stepwise joint conditional model that selected independently associated SNPs. Pairwise LD was calculated in both the 1000 Genomes Project^49^ phase 3 version 5 European reference genotypes and UKB European-ancestry sample genotypes. SNPs in LD (*r*^2^ > 0.1 in either UKB or 1000 Genomes reference at ±5 Mb) with known or novel sentinel SNPs from our primary analysis or in LD with known SNPs not available in our data were excluded. Among SNPs identified in the conditional analysis, the most significant SNP for any trait was selected within each LD block, and these independent SNPs were designated as secondary SNPs. Secondary SNPs were further evaluated to determine whether they fell within the novel portion of our data.

### GRS and PRS construction and variance explained

**Extended Data Fig. 10:**
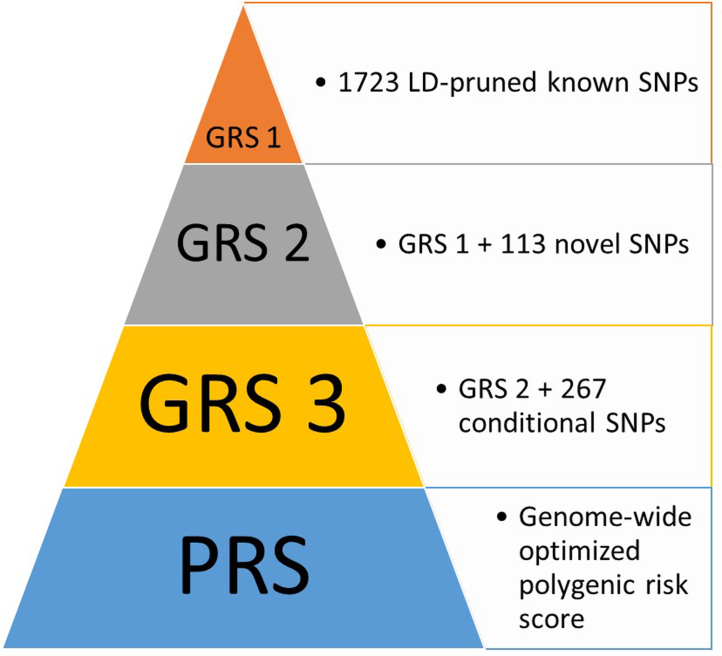
GRSs and PRS tested for percent variance explained in Lifelines cohort. Two PRSs were calculated: 1) a standard ‘benchmark’ clumping and thresholding PRS, and; 2) an ‘optimized’ PRS based on SBayesRC. GRS = Genetic Risk Score; PRS = Polygenic Risk Score; SNP = Single Nucleotide Polymorphism.

For our study, GRS is defined as a risk score comprising SNPs reaching genome-wide significance (*P* < 5 × 10^−8^) in our analyses or in previously published studies, and PRS is a full genome-wide risk score calculated by the standard clumping and thresholding method or SBayesRC^15^ (R package v.0.2.2). We calculated GRS and PRS and assessed variance explained in the Lifelines data (Extended Data Fig. 10). Both GRS and PRS were calculated as the sum of an individual’s risk alleles, weighted by BP trait-specific risk allele effect sizes. In SBayesRC, the risk allele effects of genome-wide SNPs were estimated from the GWAS data with a multi-normal mixture prior incorporating functional genomic annotations from BaselineLD (v.2.2)^70^. In addition to the SNP QC above, we further removed around 5,000 SNPs for which the per-SNP sample size in the meta-analyzed GWAS result was more than four standard deviations away from the mean value, before the SBayesRC analysis.

To calculate the percentage of BP variance explained by genetic variants in an independent dataset, we generated the residuals from a regression of each BP trait against sex, age, age^2^ and BMI in 10,210 Lifelines individuals^71^. We then fit a second linear model for the trait residuals with the top ten principal components and a third linear model for the trait residuals with ten principal components plus GRS. The difference in the adjusted *R*^2^ between the third and the second model is the estimation of the percentage of variance of the dependent (BP) variable explained by the GRS. To evaluate the contribution of previously reported BP loci as well as novel and secondary loci detected in our analyses, to observed variance in BP traits and to test the predictive value of our genome-wide results, we constructed four different GRSs and two PRSs: (1) GRS of 1,723 pairwise-independent (LD-pruned with *r*^2^ < 0.1) SNPs from published known loci; (2) GRS of 113 sentinel SNPs at genome-wide significant (*P* < 5 × 10^−8^) novel loci; (3) GRS of 1,723 known SNPs plus 113 sentinel SNPs at genome-wide significant novel loci; (4) GRS of 1,723 known SNPs plus 113 SNPs from novel loci plus 267 secondary SNPs; (5) standard clumping and thresholding PRSs at optimally selected *P* value thresholds (1 × 10^−3^, 0.01 and 0.01 for SBP, DBP and and PP, respectively) that maximized variance explained in the Lifelines data; and (6) full PRS calculated using SBayesRC, a Bayesian method that incorporates functional genomic annotations into the PRS calculation^15^. SBayesRC has been shown to have better prediction accuracy in both European ancestry and trans-ancestry prediction than other state-of-the-art PRS methods^15^.

We generated GRS and PRS by multiplying the risk allele dosages for each SNP by its respective effect size as weight and then summed all SNPs in the score. For PRS calculated by SBayesRC, the functional annotation-informed effect sizes were used as SNP weights. The four different GRS included the same set of SNPs for all three BP traits (SBP, DBP and PP) but were weighted by the trait-specific beta coefficients from the GWAS results for SBP, DBP and PP. Summary statistics for all SNPs in the GRS are displayed in Supplementary Table 4.

For each BP trait, we calculated full PRS by the clumping and thresholding approach^72^. Summary statistics of final GWAS results for each trait and the LD reference panel of 503 European ancestry samples from 1000 Genomes phase 3 (ref. ^49^) were used. SNPs with ambiguous strands (A/T or C/G) were removed for the score derivation. An LD-driven clumping procedure was then performed by PLINK version 1.90 (*r*^2^ < 0.1, 1,000 kb window). Finally, the clumping and thresholding PRSs were generated at 17 selected *P* value thresholds (1 × 10^−8^, 5 × 10^−8^, 1 × 10^−7^, 5 × 10^−7^, 1 × 10^−6^, 5 × 10^−6^, 1 × 10^−5^, 5 × 10^−5^, 1 × 10^−4^, 5 × 10^−4^, 1 × 10^−3^, 5 × 10^−3^, 0.01, 0.05, 0.1, 0.5 and 1). For optimum *P* value thresholds maximizing the variance explained in each trait, summary statistics of all SNPs are displayed in Supplementary Table 26a–c. We also applied the SBayesRC algorithm^15^ on summary statistics of final GWAS results for each BP trait and derived the effect estimates weighted by the functional annotations. These new effect estimates were made publicly available through the Polygenic Score Catalog (www.pgscatalog.org). We compared the performance of the PRS calculated by the classic clumping and thresholding approach with the PRS calculated by SBayesRC. The PRS method that explained more variance in BP traits of the Lifelines data was used in all further PRS analyses as described below.

### Decile analyses of BP PRS in Lifelines

To evaluate to what extent BP PRS were predictive for SBP, DBP, PP and hypertension, we tested the PRS of SBP, DBP and PP for decile analyses of their respective traits and modeled the joint effect of the PRS for SBP and DBP for hypertension analyses. Then we applied linear and logistic regression with adjustment for sex to compare BP levels and risk of hypertension, respectively, in all deciles versus the bottom decile of the PRS distribution of 10,210 Lifelines individuals. We also compared BP levels and risk of hypertension, respectively, in all deciles versus the middle deciles of the PRS distribution. *P* values were calculated from the normal distribution for BP traits and from a chi-squared distribution with two degrees of freedom for hypertension.

### Hypertension model performance and calibration in Lifelines

Hypertension-prediction model discrimination and calibration were examined by calculating the AUROC^73,74^ and Brier score^75,76^, respectively. Discrimination AUROC quantifies the ability of a model to classify cases and controls correctly, and specifically is the probability that a randomly chosen case will have a higher posterior probability of being a case than a randomly chosen control. Calibration quantifies the similarity of the posterior probability of being a case with the observed proportion of cases in that quantile of the ranked posterior probabilities from the model. These analyses were implemented using the *pROC* R package^77^ with tenfold cross-validation to mitigate overfitting, which occurs when predictions are made using the same data on which the model parameters were estimated. An AUROC value of 0.5 indicates no discrimination or random classification, while a value of 1 is perfect discrimination or perfect classification. The Brier score is the average squared difference between predicted probability and observed outcome, with values approaching zero indicating high calibration. The cut-off value of hypertension odds to predict high risk were identified using the Youden index (max(sensitivity + specificity)), the point on the AUROC at which sensitivity and specificity are maximized. Other cut-off points could be chosen to maximize performance for other parameters, but the Youden index is a reasonable starting point that balances several aspects of predictive performance. Statistics were calculated for two models: a model including covariates used in GWAS meta-analyses (sex, age, age^2^, BMI; model 1); and a model including covariates and PRS for SBP and DBP (model 2). We also calculated the NRI to indicate what proportion of the subjects are reclassified as high-risk or low-risk when the PRSs are added to the model.

### Comparison of restricted maximum likelihood methods to calculate heritability

The *h*^2^_SNP_ of BP traits has previously been calculated within the *n* ~ 457,000 UKB cohort GWAS dataset using the restricted maximum likelihood (REML) method BOLT-REML v2.3 (ref. ^78^); for example, with *h*^2^_SNP_ = 21.3% for SBP^5^. To check the consistency across different software and to compare to previously published results, we calculated *h*^2^_SNP_ of SBP within the UKB BP-GWAS dataset using GCTA-GREML^69^. The full imputed genetic data was converted from BGEN dosage format into hard-call genotyped PLINK format. SNPs were filtered according to MAF > 1% and high imputation quality with INFO ≥ 0.9 from the central UKB QC and then restricted to only the set of SNPs present in our full meta-analysis dataset. Owing to the high amount of RAM that GCTA software requires, we selected a representative subset from UKB for our analysis. We calculated percentiles of principal components PC1 and PC2 of all individuals from the centrally provided UKB QC data and extracted the most homogeneous subset of individuals centered around the median data points with both PC1 and PC2 within the 40–60th percentile range, resulting in a subset sample size of *n* = 19,410. Within GCTA, the genetic relatedness matrix was generated for each autosome separately, then merged together and filtered for relatedness according to a 0.2 cut-off to remove any first-degree and second-degree relatives. Then *h*^2^_SNP_ for SBP was calculated with adjustment of the same covariates applied to the UKB BP-GWAS; namely sex, age, age^2^, BMI, genotyping chip array and the top ten PCs. One-tailed *P* values were calculated according to the *h*^2^_SNP_ and standard error results in base R.

This SNP-based heritability analysis of SBP in the small subset of the UKB data (*n* = 19,410) yielded an *h*^2^_SNP_ estimate of 22.8%, which is consistent with the estimate of 21.3% reported previously^5^ using BOLT-REML, demonstrating that the GCTA-GREML approach is also appropriate to use for calculation of heritability within our other smaller Lifelines cohort.

### Heritability analyses in Lifelines data

We used GCTA-GREML^16^ to calculate *h*^2^_SNP_ for BP in the same Lifelines dataset as in the %VE analyses (*n* = 10,210). SNPs in Lifelines were restricted to the same list of SNPs used in the UKB GCTA-GREML^16^ analyses. Then *h*^2^_SNP_ for SBP, DBP and PP was calculated with adjustment of sex, age, age^2^, BMI and ten PCs.

### BP-GWAS in African-Americans from All-Of-Us (*n* = 21,843)

We performed regression association tests with additive models for untransformed medication-adjusted BP traits (SBP, DBP, PP) and hypertension case or control status using HAIL (10.5281/zenodo.6807412). Models were adjusted for age, age^2^, sex at birth, BMI and ten PCs. For quantitative BP traits, age at median SBP was used. Age at first hypertension ICD9/10 code was used for cases with a hypertension phecode, and age at median SBP measurement was used for controls and cases with only anti-hypertensive medication use. Sex was restricted to male or female at birth. BMI on the date of, or nearest to, median SBP measurement was extracted from the EHR and was restricted to the range of 10–100 kg m^−2^.

### Association of BP variants in other ancestries

We looked up the lead SNP at each of the 2,103 BP-associated loci reported in our European meta-analysis, within two different non-European ancestry samples. We extracted results from a BP-GWAS on over 145,000 individuals from the JBB^79^. We also performed a new African-ancestry BP-GWAS meta-analysis (AA-meta) comprising *n* = 83,890 African-ancestry individuals from four different datasets: UKB (*n* = 3,277), BioVU (*n* = 9,277) and MVP (*n* = 49,493) with existing GWAS results; plus results from a new BP-GWAS that we conducted in *n* = 21,843 African-American ancestry individuals from the All-Of-Us cohort. Of the total 2,103 SNPs, 1,671 and 2,102 were available and 1,613 and 2,092 SNPs remained in the JBB and AA-meta-datasets, respectively, after excluding any SNPs that were rare (MAF < 0.01) in either of the non-European datasets, for comparison of common SNPs only. We then compared the allele frequencies and the effect sizes between our European meta-analysis and each of the two non-European datasets by calculating Pearson correlations and the percentage of concordance in the direction of SNP effects. We used only the best associated BP trait for each SNP with the same trait from the non-European dataset and performed our comparisons for novel, secondary and known SNPs separately.

### BP PRS association analyses in African-American ancestry

To evaluate to what extent BP PRSs were predictive for hypertension in non-European ancestry individuals, we performed analyses of our European ancestry PRS within an African-American ancestry sample (*n* = 21,843) from the All-Of-Us cohort. We conducted the same PRS analysis pipeline as used for the European Lifelines cohort (Methods).

### In silico transcriptome-wide association study

#### Genetically predicted gene expression analysis

Our in silico transcriptome-wide association study of inferred gene expression was performed using S-PrediXcan^80^, an approach that imputes genetically predicted gene expression in a given tissue and tests predicted expression for association with a GWAS outcome using SNP-level summary statistics. For this study, input included summary statistics from each of the meta-analyses (SBP, DBP and PP) and gene-expression references for five tissues from GTEx^81^ v.7 including aorta, tibial artery, left ventricle, atrial appendage and whole blood. Our analyses incorporated covariance matrices based on 1000 Genomes^49^ European populations to account for LD structure. The Bonferroni-corrected significance threshold was 1.55 × 10^−6^ to account for the total number of gene models assessed across all tissues in these analyses.

#### Colocalization analysis

The hypothesis that a single variant underlies GWAS and eQTL associations at a given locus (that is, colocalization) was tested using COLOC^82^, a Bayesian gene-level test that evaluates GWAS and eQTL association summary statistics at each SNP at the locus and provides gene-level and SNP-level posterior probabilities for colocalization. For this analysis, inputs included results for common variants in our study and eQTL summary statistics corresponding to the gene-expression references used in the S-PrediXcan analysis, restricting to only variants included in the S-PrediXcan models. Output includes posterior probabilities for the null hypothesis (PP.H_0_) that SNPs at the locus are associated with neither gene expression nor the outcome (that is SBP, DBP or PP), the first alternative hypothesis (PP.H_1_) that SNPs are associated with expression but not the outcome, the second alternative hypothesis (PP.H_2_) that SNPs are associated with the outcome but not expression, the third alternative hypothesis (PP.H_3_) that SNPs are associated with both expression and the outcome but not colocalized and the fourth alternative hypothesis (PP.H_4_) that SNPs associated with both expression and the outcome are colocalized. Also included are annotations of the SNPs with the highest PP.H_4_ at each locus and the corresponding posterior probability. A PP.H_4_ of greater than 90% was considered evidence of colocalization.

#### Pathway analyses

Downstream analyses were performed using the functional mapping and annotation of genome-wide association studies (FUMA-GWAS)^17,83^ online software tool. The list of all 1,070 genes from the inferred gene expression analyses that were significant from S-PrediXcan and filtered after the colocalization and eQTL analyses was used as the input into FUMA, and Genotype–Tissue Expression (GTEx) v.7 was used as the gene expression dataset. All other parameters selected were chosen to be consistent with the options used for the S-PrediXcan analysis. We conducted FUMA analyses for tissue specificity tests and for gene set enrichment analyses to yield pathway analysis results according to different pathway datasets: KEGG, Reactome and WikiPathways. Four different analyses were performed according to different BP traits: a ‘unified’ analysis based on the list of all unique significant genes across all three BP traits and three trait-specific analyses for each of SBP, DBP and PP. When presenting the outputs, the adjusted *P* value results take multiple testing into account, and all results tables are filtered by adjusted *P* < 0.05.

### Ethics statement

Our study is based on meta-analysis of previously published, publicly available data for which appropriate site-specific Institutional Review Boards and ethical review at local institutions have previously approved the use of this data.

### Reporting summary

Further information on research design is available in the Nature Portfolio Reporting Summary linked to this article.

## Online content

Any methods, additional references, Nature Portfolio reporting summaries, source data, extended data, supplementary information, acknowledgements, peer review information; details of author contributions and competing interests; and statements of data and code availability are available at 10.1038/s41588-024-01714-w.

### Supplementary information

Supplementary Information Supplementary Notes and Figs. 1–23.

Reporting Summary

Supplementary Tables 1–26 Supplementary Tables 1–26, provided in separate sheets of a single workbook.

## Data Availability

Full GWAS summary statistics of our meta-analyses are publicly available on the GWAS Catalog website data repository (https://www.ebi.ac.uk/gwas) with data accession codes GCST90310294, GCST90310295 and GCST90310296 for SBP, DBP and PP, respectively. The SBayesRC PRS data for SBP, DBP and PP are deposited on the PGS Catalog website (https://www.pgscatalog.org), with data accession codes PGS004603, PGS004604 and PGS004605 for SBP, DBP and PP, respectively, alongside publication ID PGP000581. The standard clumping and threshold PRSs for SBP, DBP and PP; summary statistics for sentinel SNPs for each BP trait as well as optimized PRS; and statistically significant reports for S-PrediXcan results for all five tissues for all BP traits evaluated are available in the Supplementary Tables.

## Code availability

All software programs used in the study are publicly available as described in Methods and the Reporting Summary.

## References

[1] Mills KT (2016). Global disparities of hypertension prevalence and control: a systematic analysis of population-based studies from 90 countries. Circulation.

[2] GBD 2017 Causes of Death Collaborators. (2018). Global, regional, and national age-sex-specific mortality for 282 causes of death in 195 countries and territories, 1980–2017: a systematic analysis for the Global Burden of Disease Study 2017. Lancet.

[3] GBD 2017 Risk Factor Collaborators. (2018). Global, regional, and national comparative risk assessment of 84 behavioural, environmental and occupational, and metabolic risks or clusters of risks for 195 countries and territories, 1990–2017: a systematic analysis for the Global Burden of Disease Study 2017. Lancet.

[4] Giri A (2019). Trans-ethnic association study of blood pressure determinants in over 750,000 individuals. Nat. Genet..

[5] Evangelou E (2018). Genetic analysis of over 1 million people identifies 535 new loci associated with blood pressure traits. Nat. Genet..

[6] Wain LV (2017). Novel blood pressure locus and gene discovery using genome-wide association study and expression data sets from blood and the kidney. Hypertension..

[7] Warren HR (2017). Genome-wide association analysis identifies novel blood pressure loci and offers biological insights into cardiovascular risk. Nat. Genet..

[8] Hoffmann TJ (2017). Genome-wide association analyses using electronic health records identify new loci influencing blood pressure variation. Nat. Genet..

[9] Ehret GB (2016). The genetics of blood pressure regulation and its target organs from association studies in 342,415 individuals. Nat. Genet..

[10] Kamali Z (2022). Large-scale multi-omics studies provide new insights into blood pressure regulation. Int. J. Mol. Sci..

[11] Eales JM (2021). Uncovering genetic mechanisms of hypertension through multi-omic analysis of the kidney. Nat. Genet..

[12] van Duijvenboden S. et al. Integration of genetic fine-mapping and multi-omics data reveals candidate effector genes for hypertension. *Am J Hum Genet*. **110**, 1718–1734 (2023). 10.1016/j.ajhg.2023.08.009 PMC10577090 37683633

[13] Roden DM (2008). Development of a large-scale de-identified DNA biobank to enable personalized medicine. Clin. Pharmacol. Ther..

[14] Bulik-Sullivan BK (2015). LD score regression distinguishes confounding from polygenicity in genome-wide association studies. Nat. Genet..

[15] Zheng, Z. et al. Leveraging functional genomic annotations and genome coverage to improve polygenic prediction of complex traits within and between ancestries. *Nat. Genet.*10.1038/s41588-024-01704-y (2024). 10.1038/s41588-024-01704-y PMC11096109 38689000

[16] Yang J, Lee SH, Goddard ME, Visscher PM (2011). GCTA: a tool for genome-wide complex trait analysis. Am. J. Hum. Genet..

[17] Watanabe K, Taskesen E, van Bochoven A, Posthuma D (2017). Functional mapping and annotation of genetic associations with FUMA. Nat. Commun..

[18] Yengo L (2022). A saturated map of common genetic variants associated with human height. Nature.

[19] Sakaue S (2020). Trans-biobank analysis with 676,000 individuals elucidates the association of polygenic risk scores of complex traits with human lifespan. Nat. Med..

[20] Vaura F (2021). Polygenic risk scores predict hypertension onset and cardiovascular risk. Hypertension.

[21] Tegegne BS (2020). Heritability and the genetic correlation of heart rate variability and blood pressure in >29000 families: the Lifelines Cohort Study. Hypertension.

[22] Wainschtein, P. et al. Assessing the contribution of rare variants to complex trait heritability from whole-genome sequence data. *Nat Genet*. **54**, 263–273 (2022). 10.1038/s41588-021-00997-7 PMC9119698 35256806

[23] Surendran P (2020). Discovery of rare variants associated with blood pressure regulation through meta-analysis of 1.3 million individuals. Nat. Genet..

[24] Ding Y (2022). Large uncertainty in individual polygenic risk score estimation impacts PRS-based risk stratification. Nat. Genet..

[25] Du X (2008). The serine protease TMPRSS6 is required to sense iron deficiency. Science.

[26] Truksa J (2009). Suppression of the hepcidin-encoding gene *Hamp* permits iron overload in mice lacking both hemojuvelin and matriptase-2/TMPRSS6. Br. J. Haematol..

[27] Benyamin B (2014). Novel loci affecting iron homeostasis and their effects in individuals at risk for hemochromatosis. Nat. Commun..

[28] Charlebois E, Pantopoulos K (2021). Iron overload inhibits BMP/SMAD and IL-6/STAT3 signaling to hepcidin in cultured hepatocytes. PLoS One.

[29] Kautz L (2008). Iron regulates phosphorylation of Smad1/5/8 and gene expression of *Bmp6*, *Smad7*, *Id1*, and *Atoh8* in the mouse liver. Blood.

[30] Singh MM, Kumar R, Tewari S, Agarwal S (2019). Association of GSTT1/GSTM1 and ApoE variants with left ventricular diastolic dysfunction in thalassaemia major patients. Hematology.

[31] Wu K-H (2006). Glutathione *S*-transferase M1 gene polymorphisms are associated with cardiac iron deposition in patients with β-thalassemia major. Hemoglobin.

[32] Salonen JT (1992). High stored iron levels are associated with excess risk of myocardial infarction in eastern Finnish men. Circulation.

[33] Martínez-Salas SG (2007). α_1A_-Adrenoceptors predominate in the control of blood pressure in mouse mesenteric vascular bed. Auton. Autacoid. Pharmacol..

[34] Mahajan A (2018). Fine-mapping type 2 diabetes loci to single-variant resolution using high-density imputation and islet-specific epigenome maps. Nat. Genet..

[35] Hambrock A, Löffler-Walz C, Quast U (2002). Glibenclamide binding to sulphonylurea receptor subtypes: dependence on adenine nucleotides. Br. J. Pharmacol..

[36] Qin X, Zhong J, Lan D (2020). The use of glimepiride for the treatment of neonatal diabetes mellitus caused by a novel mutation of the *ABCC8* gene. J. Pediatr. Endocrinol. Metab..

[37] Lago-Docampo M (2020). Characterization of rare *ABCC8* variants identified in Spanish pulmonary arterial hypertension patients. Sci. Rep..

[38] Le Ribeuz H (2020). Implication of potassium channels in the pathophysiology of pulmonary arterial hypertension. Biomolecules.

[39] Southgate L, Machado RD, Gräf S, Morrell NW (2020). Molecular genetic framework underlying pulmonary arterial hypertension. Nat. Rev. Cardiol..

[40] Eichholz A, Merchant S, Gaya AM (2010). Anti-angiogenesis therapies: their potential in cancer management. Onco. Targets Ther..

[41] Chen P (2020). FGF-21 ameliorates essential hypertension of SHR via baroreflex afferent function. Brain Res. Bull..

[42] El Agha E (2018). Is the fibroblast growth factor signaling pathway a victim of receptor tyrosine kinase inhibition in pulmonary parenchymal and vascular remodeling?. Am. J. Physiol. Lung Cell. Mol. Physiol..

[43] Qiao J (2023). Evaluating significance of European-associated index SNPs in the East Asian population for 31 complex phenotypes. BMC Genomics.

[44] Kurniansyah N (2023). Evaluating the use of blood pressure polygenic risk scores across race/ethnic background groups. Nat. Commun..

[45] Fritsche LG (2021). On cross-ancestry cancer polygenic risk scores. PLoS Genet..

[46] Barroso I (2021). The importance of increasing population diversity in genetic studies of type 2 diabetes and related glycaemic traits. Diabetologia.

[47] Bycroft C (2018). The UK Biobank resource with deep phenotyping and genomic data. Nature.

[48] Ani A, van der Most PJ, Snieder H, Vaez A, Nolte IM (2021). GWASinspector: comprehensive quality control of genome-wide association study results. Bioinformatics.

[49] 1000 Genomes Project Consortium. (2015). A global reference for human genetic variation. Nature.

[50] McCarthy S (2016). A reference panel of 64,976 haplotypes for genotype imputation. Nat. Genet..

[51] Loh P-R (2016). Reference-based phasing using the Haplotype Reference Consortium panel. Nat. Genet..

[52] Das S (2016). Next-generation genotype imputation service and methods. Nat. Genet..

[53] Marchini J, Howie B, Myers S, McVean G, Donnelly P (2007). A new multipoint method for genome-wide association studies by imputation of genotypes. Nat. Genet..

[54] International Consortium for Blood Pressure Genome-Wide Association Studies. (2011). Genetic variants in novel pathways influence blood pressure and cardiovascular disease risk. Nature.

[55] Altshuler DM (2010). Integrating common and rare genetic variation in diverse human populations. Nature.

[56] Willer CJ, Li Y, Abecasis GR (2010). METAL: fast and efficient meta-analysis of genomewide association scans. Bioinformatics.

[57] Surendran P (2016). Trans-ancestry meta-analyses identify rare and common variants associated with blood pressure and hypertension. Nat. Genet..

[58] Feitosa MF (2018). Novel genetic associations for blood pressure identified via gene–alcohol interaction in up to 570 K individuals across multiple ancestries. PLoS One.

[59] Takeuchi F (2018). Interethnic analyses of blood pressure loci in populations of East Asian and European descent. Nat. Commun..

[60] de Las Fuentes L (2021). Gene–educational attainment interactions in a multi-ancestry genome-wide meta-analysis identify novel blood pressure loci. Mol. Psychiatry.

[61] Sung YJ (2019). A multi-ancestry genome-wide study incorporating gene–smoking interactions identifies multiple new loci for pulse pressure and mean arterial pressure. Hum. Mol. Genet..

[62] Sung YJ (2018). A large-scale multi-ancestry genome-wide study accounting for smoking behavior identifies multiple significant loci for blood pressure. Am. J. Hum. Genet..

[63] Kichaev G (2019). Leveraging polygenic functional enrichment to improve GWAS power. Am. J. Hum. Genet..

[64] Buniello A (2019). The NHGRI-EBI GWAS Catalog of published genome-wide association studies, targeted arrays and summary statistics 2019. Nucleic Acids Res..

[65] Staley JR (2016). PhenoScanner: a database of human genotype–phenotype associations. Bioinformatics.

[66] Purcell S (2007). PLINK: a tool set for whole-genome association and population-based linkage analyses. Am. J. Hum. Genet..

[67] Vaez A (2015). In silico post genome-wide association studies analysis of C-reactive protein loci suggests an important role for interferons. Circ. Cardiovasc. Genet..

[68] Wang K, Li M, Hakonarson H (2010). ANNOVAR: functional annotation of genetic variants from high-throughput sequencing data. Nucleic Acids Res..

[69] Yang J (2012). Conditional and joint multiple-SNP analysis of GWAS summary statistics identifies additional variants influencing complex traits. Nat. Genet..

[70] Gazal S (2017). Linkage disequilibrium-dependent architecture of human complex traits shows action of negative selection. Nat. Genet..

[71] Scholtens S (2015). Cohort Profile: LifeLines, a three-generation cohort study and biobank. Int. J. Epidemiol..

[72] International Schizophrenia Consortium. Common polygenic variation contributes to risk of schizophrenia and bipolar disorder. *Nature* **460**, 748–752 (2009). 10.1038/nature08185 PMC3912837 19571811

[73] Bilimoria KY (2013). Development and evaluation of the universal ACS NSQIP surgical risk calculator: a decision aid and informed consent tool for patients and surgeons. J. Am. Coll. Surg..

[74] Pencina MJ, D’Agostino RB (2015). Evaluating discrimination of risk prediction models: the C statistic. JAMA.

[75] Steyerberg EW (2010). Assessing the performance of prediction models: a framework for traditional and novel measures. Epidemiology.

[76] Arkes HR (1995). The covariance decomposition of the probability score and its use in evaluating prognostic estimates. SUPPORT Investigators. Med. Decis. Making.

[77] Robin X (2011). pROC: an open-source package for R and S+ to analyze and compare ROC curves. BMC Bioinf..

[78] Loh P-R, Kichaev G, Gazal S, Schoech AP, Price AL (2018). Mixed-model association for biobank-scale datasets. Nat. Genet..

[79] Sakaue S (2021). A cross-population atlas of genetic associations for 220 human phenotypes. Nat. Genet..

[80] Barbeira AN (2018). Exploring the phenotypic consequences of tissue specific gene expression variation inferred from GWAS summary statistics. Nat. Commun..

[81] GTEx Consortium. (2015). Human genomics. The Genotype–Tissue Expression (GTEx) pilot analysis: multitissue gene regulation in humans. Science.

[82] Giambartolomei C (2014). Bayesian test for colocalisation between pairs of genetic association studies using summary statistics. PLoS Genet..

[83] *Functional Mapping and Annotation of Genome-Wide Association Studies*; https://fuma.ctglab.nl/

