# Supplementary material for: Genome-wide analysis in over 1 million individuals of European ancestry yields improved polygenic risk scores for blood pressure traits
Source: Nat Genet. 2024 Apr 30;56(5):778–91. doi: 10.1038/s41588-024-01714-w (PMC11096100; doi:10.1038/s41588-024-01714-w)
Supplement: Supplementary file 1 — Supplementary Notes and Figs. 1–23. [file 41588_2024_1714_MOESM1_ESM.pdf]

nature genetics

Article https://doi.org/10.1038/s41588-024-01714-w

Genome-wide analysis in over 1 million individuals of European ancestry yields improved polygenic risk scores for blood pressure traits

In the format provided by the authors and unedited

Supplementary Notes

Study Populations

UKB

UKB includes ~500,000 volunteers aged 40-69 years of age ascertained through NHS registers. Following informed consent participants completed a standardized questionnaire on life course exposures, medical history and treatments and underwent a standardized portfolio of phenotypic tests including two BP measurements taken seated after two minutes rest using an appropriate cuff and an Omron HEM-7015IT digital BP monitor. A manual sphygmomanometer was used if the standard automated device could not be employed. Body mass index (BMI) was calculated as weight (kg) divided by height squared (m^2) with weight measured using an electronic weighing scale (Tanita BC-418). The participants undergo longitudinal life course linkage to electronic health data including Hospital Episode Statistics and Office for National Statistics cause of death data.

DNA extraction and genotyping for UKB has been previously described[47]. Briefly, UKB genetic data includes genotypes for 488,377 individuals. DNA was extracted from stored blood samples and genotyping was carried out by Affymetrix Research Services Laboratory. 49,950 participants involved in the UK Biobank Lung Exome Variant Evaluation (UK BiLEVE) study were genotyped at 807,411 markers using the Affymetrix UK BiLEVE Axiom Array and 438,427 participants were genotyped using the Affymetrix UK Biobank Axiom Array (825,927 markers), which shares 95% of marker content with the UK BiLEVE Axiom Array.

Variants were imputed centrally by UK Biobank using a reference panel that merged the UK10K and 1000 Genomes Phase 3 panel as well as the Haplotype Reference Consortium (HRC) panel[48]. For current analysis only SNPs imputed from the HRC panel were analyzed (N=39,235,157), of which ~7.1 million SNPs with minor allele frequency (MAF) >1% and imputation quality INFO >0.1 are analyzed here for GWAS.

For the UKB GWAS, we calculated the mean SBP and DBP values from two automated (N=418,755) or two manual (N=25,888) BP measurements. For individuals with one manual and one automated BP measurement (N=13,521), we used the mean of these two values. For individuals with only one available BP measurement (N=413), we used this single value. Following both genetic and phenotypic data QC and by excluding pregnant women (n=372) and those individuals who had withdrawn consent (N=36), the sample size for analysis

therefore included N=458,577 and N=458,575 self-reported European-ancestry individuals for SBP and DBP, respectively. For measures taken while a patient was on an antihypertensive medication, we added 15 mm Hg to SBP and 10 mm Hg to DBP. We performed linear mixed model (LMM) association testing under an additive genetic model of the three (untransformed) continuous, medication-adjusted BP traits (SBP, DBP, PP) for all measured and imputed genetic variants in dosage format using the BOLT-LMM (v2.3) software.

ICBP

ICBP GWAS is an international consortium to investigate BP genetics and has been previously described elsewhere[6,49]. All study participants were of European descent and were imputed to either the 1000 Genomes Project Phase 1 integrated release version 3 [March 2012] all ancestry reference panel or the HRC panel. The final ICBP GWAS dataset included 77 studies comprising data from 299,024 individuals. Three quantitative BP traits were analyzed: SBP, DBP, and PP. Within each study, BP measures were adjusted for medication use by adding 15 and 10 mm Hg to SBP and DBP, respectively.

Prior to meta-analysis of all 77 ICBP GWAS studies, we undertook central quality control checks across all studies. This included checks to ensure allele frequency consistency (across studies and with reference populations), checks of effect size and standard error distributions (i.e., to highlight phenotype issues) and generation of quantile-quantile (QQ) plots and genomic inflation factor lambdas to check for over- or under-inflation of test statistics. Genomic control was applied (if lambda>1) at study-level. Variants with imputation quality <0.1 were excluded prior to meta-analysis. EasyQC was used for the quality control process[50]. Finally, data were filtered to SNPs with MAF ≥1% and effective sample size (reflecting the quality of genotype imputation) >60% of the total effective sample size. Meta-analysis was performed using METAL software employing inverse variance weighted fixed-effects models[51]. Between-study heterogeneity was assessed using the Cochran’s Q statistic and we performed additional filtering removing heterogeneous variants with Cochran’s Q $p<1x10^{-4}$.

MVP Study

The MVP study is a large cohort of fully consented participants who were recruited from the patient populations of 63 Department of Veterans Affairs (VA) medical facilities. Summary statistics from the analysis of 220,501 self-reported non-Hispanic white participants were

included in our meta-analysis. These results have been previously reported by Giri et al[4]. Briefly, DNA was extracted from whole blood and genotyped using a custom Affymetrix array (Axiom Biobank; Thermo Fischer Scientific Inc, Waltham, MA, USA). Genotype calling and QC were performed centrally and genotypes were phased using EAGLE v2[52] and imputed from the 1000 Genomes Project phase 3 version 5 reference panel using Minimac3[53] software. Participants included adults (age ≥18 years) with non-Emergency Department outpatient SBP and DBP measures available in their electronic health record. For individuals with greater than or equal to three measures available, median SBP and corresponding DBP were used in analysis. For rare cases where fewer than three measures were available, the lowest available SBP and corresponding DBP were used. We observed an average of 220 measures across individuals. In individuals in whom the median SBP value was observed at multiple clinical encounters on distinct dates, we used the earliest of those measures to identify the DBP, age, BMI, and anti-hypertensive treatment status of the individual at that time. Measures were ineligible if they occurred at or after an International Classification of Diseases Ninth Revision (ICD-9) code from the groups 585 (chronic kidney disease), 405 (secondary hypertension), or 428 (heart failure). If pain scores were available, BP measures taken during encounters when a pain score ≥5 was recorded were also ineligible. BP measures were adjusted for medication use by adding 15 and 10 mm Hg to SBP and DBP, respectively. Linear regression association tests were conducted using additive models for untransformed medication-adjusted BP traits (SBP, DBP, PP) using SNPTEST-v2.5.4-beta[54].

BioVU

The BioVU DNA Repository is a deidentified database of electronic health records that are linked to patient DNA samples at Vanderbilt University Medical Center. Summary statistics from the analysis of 50,649 self-reported non-Hispanic white participants were included in our meta-analysis. A detailed description of the database and how it is maintained has been published elsewhere[13]. BioVU participant DNA samples were genotyped on a custom Illumina Multi-Ethnic Genotyping Array (MEGA-ex; Illumina Inc., San Diego, CA, USA). Quality control (QC) was conducted, excluding samples or variants with missingness rates above 2%. Samples were also excluded if consent had been revoked, sample was duplicated, or failed sex concordance checks. Imputation was performed on the Michigan Imputation Server v1.2.4[53] using Minimac4 and the Haplotype Reference Consortium panel v1.1[48].

Among BioVU participants, we selected unrelated self-reported adults of European ancestry (age ≥ 18 years) and used the earliest median eligible non-Emergency Department outpatient

measured SBP in the electronic health record, and the corresponding DBP. For individuals with fewer than three measurements available (N=2,933), the lowest available SBP and corresponding DBP were used. On average, there were 69 SBP measures per individual. Measures were considered ineligible if they occurred at or after an ICD-9/10 billing code from the groups 585/N18 (chronic kidney disease), 405/I15 (secondary hypertension), or 428/I50 (heart failure). For measures taken while a patient was on an antihypertensive medication, we added 15 mm Hg to SBP and 10 mm Hg to DBP. We performed linear regression association tests with additive models for untransformed medication-adjusted BP traits (SBP, DBP, PP) using SNPTEST-v2.5.4-beta[54].

Lifelines Cohort Study genotype and phenotype data

The Lifelines cohort is a large prospective population-based cohort study performed in 167,729 individuals living in the North of the Netherlands with a unique three generation design, aiming at investigating risk factors for multifactorial diseases[72]. It was approved by the medical ethics committee of the University Medical Center Groningen and conducted in accordance with Helsinki Declaration Guidelines. All participants signed an informed consent form prior to enrollment.

A subset of 38,030 volunteers were genotyped using the Infinium Global Screening Array MultiEthnic Disease Version, according to manufacturer's instructions, at the Human Genomics Facility of the Erasmus Medical Center, Rotterdam and the Department of Genetics, University Medical Center Groningen. Standard QC was performed on both samples and markers. Samples with a genotyping call rate<99%, outliers for heterozygosity and sex mismatches were excluded, as well as samples that did not show consistent information between reported familial information and observed identity-by-descent sharing with family members, and between genotypes available from this and previous studies. Variants with a genotyping call rate <99%, Hardy-Weinberg equilibrium P $<1\times10^{-6}$ or excess of Mendelian errors in families (>1% of the parent-offspring pairs) were removed. A total of 36,339 samples and 571,420 autosomal and X-chromosome markers passed quality checks. The genotyping dataset was then imputed at the Sanger imputation server1 using the HRC panel v1.1.

From the set of 36,339 samples, we selected 10,782 unrelated individuals who are also independent from Lifelines samples that were included in a previous ICBP meta-GWAS[5]. After excluding 552 children (age<18 years), 12 pregnant women, five individuals without

SBP or DBP, and three individuals without BMI, a final total number of 10,210 individuals were included for analyses that broadly represent the total Lifelines sample (see Supplementary Table 24b).

In Lifelines, BP was measured every minute during a period of ten minutes using an automated DINAMAP Monitor (GE Healthcare) and the average of the final three readings was recorded for SBP and DBP. Participants with a measured BP ≥140/90 mm Hg irrespective of treatment and those taking antihypertensive medication (ATC codes C02, C03, C07, C08, C09) irrespective of BP were defined as having hypertension. In continuous trait analyses, 15 mm Hg was added to SBP and 10 mm Hg was added to DBP for 1,236 individuals who were taking antihypertensive medication. PP was calculated using these medication-adjusted BP values.

African-American Cohort from the All of Us Research Program

The NIH All of Us Research Program is a deidentified database of electronic health records linked to participant genomic data from contributing medical centers nationwide. Analysis considered 24,718 predicted African-ancestry individuals (from PC clustering) before phenotyping, and for whom whole genome sequencing data was available. Briefly, we selected unrelated individuals over the age of 18 and extracted the earliest median eligible non-Emergency Department outpatient measured SBP in the electronic health record, and the corresponding DBP. For individuals with an even number of SBP measures in their record, the lower value was used to compute the median. For individuals with fewer than three measurements available, the lowest available SBP and corresponding DBP were used. Measures were considered ineligible if they occurred at or after an ICD-9/10 billing code from the groups 585/N18 (chronic kidney disease), 405/I15 (secondary hypertension), or 428/I50 (heart failure). For participants who had started an antihypertensive medication before the date of their median SBP measurement, 15 mm Hg was added to SBP and 10 mm Hg to DBP. Eligible SBP measures were restricted to a range of 30 to 300 mmHg. Eligible DBP measures were restricted to values over 30 mmHg. Sample size for SBP, DBP, and PP GWAS analysis included 21,843 individuals. Pulse pressure was defined as SBP minus DBP. Hypertension status was defined by phecodes 401* and/or antihypertensive medication use. Sample size for hypertension case/control GWAS included 21,843 individuals (n = 8,098 cases and 13,745 controls). See Supplementary Table 13 for demographics of the All-Of-Us cohort.

Cohort Acknowledgements

MVP

This research is based in part on data from the Million Veteran Program, Office of Research and Development, Veterans Health Administration. This publication does not represent the views of the Department of Veterans Affairs or the United States Government.

UKB

This research has been conducted using the UK Biobank Resource under Application Numbers 236 and 10035. This research was supported by the British Heart Foundation (grant SP/13/2/30111). Large-scale comprehensive genotyping of UK Biobank for cardiometabolic traits and diseases: UK CardioMetabolic Consortium (UKCMC). UK Biobank research acknowledges NHS Digital, as this work uses data provided by patients and collected by the NHS as part of their care and support: Copyright © 2022, NHS Digital. Re-used with the permission of the NHS Digital and UK Biobank. All rights reserved. UK Biobank research acknowledges Public Health Scotland. This research used data assets made available by National Safe Haven as part of the Data and Connectivity National Core Study, led by Health Data Research UK in partnership with the Office for National Statistics and funded by UK Research and Innovation (research which commenced between 1st October 2020 – 31st March 2021 grant ref MC_PC_20029; 1st April 2021 -30th September 2022 grant ref MC_PC_20058).

BioVU

BioVU is supported by institutional funding, the 1S10RR025141-01 instrumentation award, and by the Vanderbilt CTSA grant UL1TR000445 from NCATS/NIH. This work was conducted in part using the resources of the Advanced Computing Center for Research and Education at Vanderbilt University, Nashville, TN, supported in part by an S10 instrumentation award (1S10OD023680-01).

Lifelines Cohort Study

The Lifelines Biobank initiative has been made possible by funding from the Dutch Ministry of Health, Welfare and Sport, the Dutch Ministry of Economic Affairs, the University Medical Center Groningen (UMCG the Netherlands), University of Groningen and the Northern Provinces of the Netherlands. The generation and management of GWAS genotype data for the Lifelines Cohort Study is supported by the UMCG Genetics Lifelines Initiative (UGLI). UGLI is partly supported by a Spinoza Grant from NWO, awarded to Cisca Wijmenga.

The authors wish to acknowledge the services of the Lifelines Cohort Study, the contributing research centers delivering data to Lifelines, and all the study participants.

UMCG Genetics Lifelines Initiative (UGLI) group author LifeLines Cohort Study:

Raul Aguirre-Gamboa (1), Patrick Deelen (1), Lude Franke (1), Jan A Kuivenhoven (2), Esteban A Lopera Maya (1), Ilja M Nolte (3), Serena Sanna (1), Harold Snieder (3), Morris A Swertz (1), Peter M. Visscher (3,4), Judith M Vonk (3), Cisca Wijmenga (1)

(1) *Department of Genetics, University of Groningen, University Medical Center Groningen, The Netherlands*

(2) *Department of Pediatrics, University of Groningen, University Medical Center Groningen, The Netherlands*

(3) *Department of Epidemiology, University of Groningen, University Medical Center Groningen, The Netherlands*

(4) *Institute for Molecular Bioscience, The University of Queensland, Brisbane, Queensland, Australia.*

KORA Study

The KORA study was initiated and financed by the Helmholtz Zentrum München –German Research Center for Environmental Health, which is funded by the German Federal Ministry of Education and Research (BMBF) and by the State of Bavaria. Furthermore, KORA research was supported within the Munich Center of Health Sciences (MC-Health), Ludwig-Maximilians-Universität, as part of LMUinnovativ and funded by the Bavarian State Ministry of Health and Care through the research project DigiMed Bayern (www.digimed-bayern.de). Group authors include Annette Peters (PI) and Christian Gieger (CoPI) and Melanie Waldenberger. The informed consents given by KORA study participants do not cover data posting in public databases. However, data are available upon request from KORA Project Application Self-Service Tool (https://epi.helmholtz-muenchen.de/) Data requests can be submitted online and are subject to approval by the KORA Board.

Cohorts for Heart and Aging Research in Genomic Epidemiology (CHARGE) Consortium

The CHARGE cohorts were supported in part by HL105756.

Cardiovascular Health Study (CHS)

This CHS research was supported by NHLBI contracts HHSN268201200036C, HHSN268200800007C, HHSN268201800001C, N01HC55222, N01HC85079, N01HC85080, N01HC85081, N01HC85082, N01HC85083, N01HC85086, 75N92021D00006; and NHLBI grants U01HL080295, R01HL087652, R01HL105756, R01HL103612, R01HL120393, and U01HL130114 with additional contribution from the National Institute of Neurological Disorders and Stroke (NINDS). Additional support was provided through R01AG023629 from the National Institute on Aging (NIA). A full list of principal CHS investigators and institutions can be found at CHS-NHLBI.org. The provision of genotyping data was supported in part by the National Center for Advancing Translational Sciences, CTSI grant UL1TR001881, and the National Institute of Diabetes and Digestive and Kidney Disease Diabetes Research Center (DRC) grant DK063491 to the Southern California Diabetes Endocrinology Research Center.

MESA

MESA and the MESA SHARe projects are conducted and supported by the National Heart, Lung, and Blood Institute (NHLBI) in collaboration with MESA investigators. Support for MESA is provided by contracts 75N92020D00001, HHSN268201500003I, N01-HC-95159, 75N92020D00005, N01-HC-95160, 75N92020D00002, N01-HC-95161, 75N92020D00003, N01-HC-95162, 75N92020D00006, N01-HC-95163, 75N92020D00004, N01-HC-95164, 75N92020D00007, N01-HC-95165, N01-HC-95166, N01-HC-95167, N01-HC-95168, N01-HC-95169, UL1-TR-000040, UL1-TR-001079, and UL1-TR-001420, UL1TR001881, DK063491, and R01HL105756. Funding for SHARe genotyping was provided by NHLBI Contract N02-HL-64278. Genotyping was performed at Affymetrix (Santa Clara, California, USA) and the Broad Institute of Harvard and MIT (Boston, Massachusetts, USA) using the Affymetrix Genome-Wide Human SNP Array 6.0. The authors thank the other investigators, the staff, and the participants of the MESA study for their valuable contributions. A full list of participating MESA investigators and institutes can be found at http://www.mesa-nhlbi.org

National Institutes of Health (NIH)

This research was supported in part by the Intramural Research Program of the NIH, National Human Genome Research Institute (NHGRI; project number Z01 HG200417), National Institute on Aging (NIA; project number Z01 AG000535), National Institutes of Health, Department of Health and Human Services, as well as the National Institute of Neurological Disorders and Stroke.

CROATIA Study

The CROATIA cohorts were funded by grants from the MRC (United Kingdom), European Commission Framework 6 project EUROSPAN (contract number LSHG-CT-2006-018947), Croatian Science Foundation (grant 8875), the Republic of Croatia Ministry of Science, Education and Sports (216-1080315-0302) and the Croatian National Center of Research Excellence in Personalized Healthcare (grant number KK.01.1.1.01.0010) and the Center of Competence in Molecular Diagnostics (KK.01.2.2.03.0006).

Young Finns Study

The Young Finns Study has been financially supported by the Academy of Finland: grants 322098, 286284, 134309 (Eye), 126925, 121584, 124282, 255381, 256474, 283115, 319060, 320297, 314389, 338395, 330809, 104821, 129378 (Salve), 117797 (Gendi), and 141071 (Skidi); the Social Insurance Institution of Finland; Competitive State Research Financing of the Expert Responsibility area of Kuopio, Tampere and Turku University Hospitals (grant X51001); Juho Vainio Foundation; Paavo Nurmi Foundation; Finnish Foundation for Cardiovascular Research; Finnish Cultural Foundation; The Sigrid Juselius Foundation; Tampere Tuberculosis Foundation; Emil Aaltonen Foundation; Yrjö Jahnsson Foundation; Signe and Ane Gyllenberg Foundation; Diabetes Research Foundation of Finnish Diabetes Association; EU Horizon 2020 (grant 755320 for TAXINOMISIS and grant 848146 for To Aition); European Research Council (grant 742927 for MULTIEPIGEN project); Tampere University Hospital Supporting Foundation, Finnish Society of Clinical Chemistry and the Cancer Foundation Finland.

HTO

Collection of the HTO cohort was funded by the Wellcome Trust. Genotyping was funded by the British Heart Foundation (BHF). BK holds a BHF Personal Chair (CH/13/2/30154).

Women's Genome Health Study (WGHS)

The WGHS is supported by the National Heart, Lung, and Blood Institute (HL043851 and HL080467) and the National Cancer Institute (CA047988 and UM1CA182913) with funding for genotyping provided by Amgen.

Justification for the Use of Statins in Prevention: an Intervention Trial Evaluating Rosuvastatin (JUPITER)

JUPITER and the genotyping in the JUPITER study population were funded by AstraZeneca.

Lothian Birth Cohorts of 1921 and 1936 (LBC)

The authors thank all LBC study participants and research team members who have contributed, and continue to contribute, to ongoing studies. LBC1921 was supported by the UKRI™ Biotechnology and Biological Sciences Research Council (BBSRC), The Royal Society, and The Chief Scientist Office of the Scottish Government. LBC1936 is supported by the BBSRC, and the Economic and Social Research Council [BB/W008793/1], Age UK (Disconnected Mind project), the Milton Damerel Trust, and the University of Edinburgh. Genotyping of the cohorts was funded by the BBSRC (BB/F019394/1).

Generation Scotland

Generation Scotland received core support from the Chief Scientist Office of the Scottish Government Health Directorates [CZD/16/6] and the Scottish Funding Council [HR03006] and is currently supported by the Wellcome Trust [216767/Z/19/Z]. Genotyping of the GS:SFHS samples was carried out by the Genetics Core Laboratory at the Edinburgh Clinical Research Facility, University of Edinburgh, Scotland and was funded by the Medical Research Council UK and the Wellcome Trust (Wellcome Trust Strategic Award Stratifying Resilience and Depression Longitudinally" (STRADL) Reference 104036/Z/14/Z). CH was supported by an MRC Human Genetics Unit programme grant 'Quantitative traits in health and disease' (U. MC_UU_00007/10).

CHD Exome+ Consortium:

This work was supported by core funding from: the UK Medical Research Council (G0800270; MR/L003120/1), the British Heart Foundation (SP/09/002; RG/13/13/30194; RG/18/13/33946) and the National Institute for Health Research [Cambridge Biomedical Research Centre at the Cambridge University Hospitals NHS Foundation Trust]*. Work was also funded by the European Research Council (268834), the European Commission Framework Programme 7 (HEALTH-F2- 2012-279233), Pfizer, Novartis and Merck.

Consortia Acknowledgements

The Million Veterans Program (MVP)

Authors on our paper from MVP:

Todd L. Edwards; Jacklyn N. Hellwege, Ayush Giri, Christopher J. O'Donnell; Peter W.F. Wilson; Yan V. Sun; Kelly Cho

MVP acknowledgement: VA Award BX004821 (Wilson/Cho)

UMCG Genetics Lifelines Initiative (UGLI) group author: LifeLines Cohort Study

Raul Aguirre-Gamboa (1), Patrick Deelen (1), Lude Franke (1), Jan A Kuivenhoven (2), Esteban A Lopera Maya (1), Ilja M Nolte (3), Serena Sanna (1), Harold Snieder (3), Morris A Swertz (1), Peter M. Visscher (3,4), Judith M Vonk (3), Cisca Wijmenga (1), Naomi Wray (4)

(1) *Department of Genetics, University of Groningen, University Medical Center Groningen, The Netherlands*

(2) *Department of Pediatrics, University of Groningen, University Medical Center Groningen, The Netherlands*

(3) *Department of Epidemiology, University of Groningen, University Medical Center Groningen, The Netherlands*

(4) *Institute for Molecular Bioscience, The University of Queensland, Brisbane, Queensland, Australia.*

Acknowledgements for Lifelines Cohort Study:

The Lifelines Biobank initiative has been made possible by funding from the Dutch Ministry of Health, Welfare and Sport, the Dutch Ministry of Economic Affairs, the University Medical Center Groningen (UMCG the Netherlands), University of Groningen and the Northern Provinces of the Netherlands. The generation and management of GWAS genotype data for the Lifelines Cohort Study is supported by the UMCG Genetics Lifelines Initiative (UGLI). UGLI is partly supported by a Spinoza Grant from NWO, awarded to Cisca Wijmenga.

The authors wish to acknowledge the services of the Lifelines Cohort Study, the contributing research centers delivering data to Lifelines, and all the study participants.

CHARGE consortium

Cohorts for Heart and Aging Research in Genomic Epidemiology (CHARGE) is a consortium formed to facilitate meta-analyses of genome-wide association studies of aging and cardiovascular traits, and the replication of genotype – phenotype associations identified in such studies.

Cardiovascular Health Research Unit, Departments of Medicine and Epidemiology, University of Washington, Seattle, WA, USA

International Consortium of Blood Pressure (ICBP)

Authors & Contributors from the International Consortium of Blood Pressure (ICBP) associated with the Evangelou et al, 2018 Nature Genetics publication: "Genetic analysis of over one million people identifies 535 new loci associated with blood pressure traits."

Evangelos Evangelou[1,2], Helen R Warren[3,4], He Gao[1,5], Georgios Ntritsos[2], Niki Dimou[2], Tonu Esko[16,17], Reedik Mägi[16], Lili Milani[16], Peter Almgren[18], Thibaud Boutin[19], Stéphanie Debette[20,21], Jun Ding[22], Franco Giulianini[23], Elizabeth G Holliday[24], Anne U Jackson[25], Ruifang Li-Gao[26], Wei-Yu Lin[27], Jian'an Luan[28], Massimo Mangino[29,30], Christopher Oldmeadow[24], Bram Peter Prins[31], Yong Qian[22], Muralidharan Sargurupremraj[21], Nabi Shah[32,33], Praveen Surendran[27], Sébastien Thériault[34,35], Niek Verweij[17,36,37], Sara M Willems[28], Jing-Hua Zhao[28], Philippe Amouyel[38], John Connell[39], Renée de Mutsert[26], Alex SF Doney[32], Martin Farrall[40,41], Cristina Menni[29], Andrew D Morris[42], Raymond Noordam[43], Guillaume Paré[34], Neil R Poulter[44], Denis C Shields[45], Alice Stanton[46], Simon Thom[47], Gonçalo Abecasis[48], Najaf Amin[49], Dan E Arking[50], Kristin L Ayers[51,52], Caterina M Barbieri[53], Chiara Batini[54], Joshua C Bis[55], Tineka Blake[54], Murielle Bochud[56], Michael Boehnke[25], Eric Boerwinkle[57], Dorret I Boomsma[58], Erwin P Bottinger[59], Peter S Braund[60,61], Marco Brumat[62], Archie Campbell[63,64], Harry Campbell[65], Aravinda Chakravarti[50], John C Chambers[1,5,66-68], Ganesh Chauhan[69], Marina Ciullo[70,71], Massimiliano Cocca[72], Francis Collins[73], Heather J Cordell[51], Gail Davies[74,75], Martin H de Borst[76], Eco J de Geus[58], Ian J Deary[74,75], Joris Deelen[77], Fabiola Del Greco M[78], Cumhur Yusuf Demirkale[79], Marcus Dörr[80,81], Georg B Ehret[50,82], Roberto Elosua[83,84], Stefan Enroth[85], A Mesut Erzurumluoglu[54], Teresa Ferreira[86,87], Mattias Frånberg[88-90], Oscar H Franco[91], Ilaria Gandin[62], Paolo Gasparini[62,72], Vilmantas Giedraitis[92], Christian Gieger[93-95], Giorgia Girotto[62,72], Anuj Goel[40,41], Alan J Gow[74,96], Vilmundur Gudnason[97,98], Xiuqing Guo[99], Ulf Gyllensten[85], Anders Hamsten[88,89], Tamara B Harris[100], Sarah E Harris[63,74], Catharina A Hartman[101], Aki S Havulinna[102,103], Andrew A Hicks[78], Edith Hofer[104,105], Albert Hofman[91,106], Jouke-Jan Hottenga[58], Jennifer E Huffman[19,107,108], Shih-Jen Hwang[107,108], Erik Ingelsson[109,110], Alan James[111,112], Rick Jansen[113], Marjo-Riitta Jarvelin[1,5,114-116], Roby Joehanes[107,117], Åsa Johansson[85], Andrew D Johnson[107,118], Peter K Joshi[65], Pekka Jousilahti[102], J Wouter Jukema[119], Antti Jula[102], Mika Kähönen[120,121], Sekar Kathiresan[17,36,122], Bernard D Keavney[123,124], Kay-Tee Khaw[125], Paul Knekt[102], Joanne Knight[126], Ivana Kolcic[127], Jaspal S Kooner[5,67,68,128], Seppo Koskinen[102], Kati Kristiansson[102], Zoltan Kutalik[56,129], Maris Laan[130], Marty Larson[107], Lenore J Launer[100], Benjamin Lehne[1], Terho Lehtimäki[131,132], David CM Liewald[74,75], Li Lin[82], Lars Lind[133], Cecilia M Lindgren[40,87,134], YongMei Liu[135], Ruth JF Loos[28,59,136], Lorna M Lopez[74,137,138], Yingchang Lu[59], Leo-Pekka Lyytikäinen[131,132], Anubha Mahajan[40], Chrysovalanto Mamasoula[139], Jaume Marrugat[83], Jonathan Marten[19], Yuri Milaneschi[140], Anna Morgan[62], Andrew P Morris[40,141], Alanna C Morrison[142], Peter J Munson[79], Mike A Nalls[143,144], Priyanka Nandakumar[50], Christopher P Nelson[60,61], Teemu Niiranen[102,145], Ilja M Nolte[146], Teresa Nutile[70], Albertine J Oldehinkel[147], Ben A Oostra[49], Paul F O'Reilly[148], Elin Org[16], Sandosh Padmanabhan[64,149], Walter Palmas[150], Aarno Palotie[103,151,152], Alison Pattie[75], Brenda WJH Penninx[140], Markus Perola[102,103,153], Annette Peters[94,95,154], Ozren Polasek[127,155], Peter P Pramstaller[78,156,157], Quang Tri Nguyen[79], Olli T Raitakari[158,159], Rainer Rettig[161], Kenneth Rice[162], Paul M Ridker[23,163], Janina S Ried[94], Harriëtte Riese[147], Samuli Ripatti[103,164], Antonietta Robino[72], Lynda M Rose[23], Jerome I Rotter[99], Igor Rudan[165], Daniela Ruggiero[70,71], Yasaman Saba[166], Cinzia F Sala[53], Veikko Salomaa[102], Nilesh J Samani[60,61], Antti-Pekka Sarin[103], Reinhold Schmidt[104], Helena Schmidt[166], Nick Shrine[54], David Siscovick[167], Albert V Smith[97,98], Harold Snieder[146], Siim Sõber[130], Rossella Sorice[70], John M Starr[74,168], David J Stott[169], David P Strachan[170], Rona J Strawbridge[88,89], Johan Sundström[133], Morris A Swertz[171], Kent D Taylor[99], Alexander Teumer[81,172], Martin D Tobin[54], Maciej Tomaszewski[123,124], Daniela Toniolo[53], Michela

Traglia[53], Stella Trompet[119,173], Jaakko Tuomilehto[174-177], Christophe Tzourio[21], André G Uitterlinden[91,178], Ahmad Vaez[146,179], Peter J van der Most[146], Cornelia M van Duijn[49], Germaine C Verwoert[91], Veronique Vitart[19], Uwe Völker[81,180], Peter Vollenweider[181], Dragana Vuckovic[62,182], Hugh Watkins[40,41], Sarah H Wild[183], Gonneke Willemsen[58], James F Wilson[19,65], Alan F Wright[19], Jie Yao[99], Tatijana Zemunik[184], Weihua Zhang[1,67], John R Attia[24], Adam S Butterworth[27,185], Daniel I Chasman[23,163], David Conen[186,187], Francesco Cucca[188,189], John Danesh[27,185], Caroline Hayward[19], Joanna MM Howson[27], Markku Laakso[190], Edward G Lakatta[191], Claudia Langenberg[28], Olle Melander[18], Dennis O Mook-Kanamori[26,192], Colin NA Palmer[32], Lorenz Risch[193-195], Robert A Scott[28], Rodney J Scott[24], Peter Sever[128], Tim D Spector[29], Pim van der Harst[196], Nicholas J Wareham[28], Eleftheria Zeggini[31], Daniel Levy[107,118], Patricia B Munroe[3,4], Christopher Newton-Cheh[134,197,198], Morris J Brown[3,4], Andres Metspalu[16], Bruce M. Psaty[201,202], Louise V Wain[54], Paul Elliott[1,5,203-205], Mark J Caulfield[3,4]

1. Department of Epidemiology and Biostatistics, Imperial College London, London, UK.
2. Department of Hygiene and Epidemiology, University of Ioannina Medical School, Ioannina, Greece.
3. William Harvey Research Institute, Barts and The London School of Medicine and Dentistry, Queen Mary University of London, London, UK.
4. National Institute for Health Research, Barts Cardiovascular Biomedical Research Center, Queen Mary University of London, London, UK.
5. MRC-PHE Centre for Environment and Health, Imperial College London, London, UK.
7. Division of Epidemiology, Department of Medicine, Institute for Medicine and Public Health, Vanderbilt Genetics Institute, Vanderbilt University Medical Center, Tennessee Valley Healthcare System (626)/Vanderbilt University, Nashville, TN, USA.
8. Vanderbilt Genetics Institute, Vanderbilt Epidemiology Center, Department of Obstetrics and Gynecology, Vanderbilt University Medical Center; Tennessee Valley Health Systems VA, Nashville, TN, USA.
9. Department of Epidemiology, Emory University Rollins School of Public Health, Atlanta, GA, USA.
10. Department of Biomedical Informatics, Emory University School of Medicine, Atlanta, GA, USA.
11. Massachusetts Veterans Epidemiology Research and Information Center (MAVERIC), VA Boston Healthcare System, Boston, USA.
12. Division of Aging, Department of Medicine, Brigham and Women’s Hospital, Boston, MA, Department of Medicine, Harvard Medical School, Boston, MA, USA.
13. Atlanta VAMC and Emory Clinical Cardiovascular Research Institute, Atlanta, GA, USA.
14. VA Palo Alto Health Care System; Division of Cardiovascular Medicine, Stanford University School of Medicine, CA, USA.
15. Nephrology Section, Memphis VA Medical Center and University of Tennessee Health Science Center, Memphis, TN, USA.
16. Estonian Genome Center, University of Tartu, Tartu, Estonia.
17. Program in Medical and Population Genetics, Broad Institute of Harvard and MIT, Cambridge, MA, USA.

18. Department Clinical Sciences, Malmö, Lund University, Malmö, Sweden.
19. MRC Human Genetics Unit, MRC Institute of Genetics and Molecular Medicine, University of Edinburgh, Western General Hospital, Edinburgh, Scotland, UK
20. Department of Neurology, Bordeaux University Hospital, Bordeaux, France.
21. Univ. Bordeaux, Inserm, Bordeaux Population Health Research Center, CHU Bordeaux, Bordeaux, France.
22. Laboratory of Genetics and Genomics, NIA/NIH , Baltimore, MD, USA.
23. Division of Preventive Medicine, Brigham and Women's Hospital, Boston, MA, USA.
24. Hunter Medical Reseach Institute and Faculty of Health, University of Newcastle, New Lambton Heights, New South Wales, Australia.
25. Department of Biostatistics and Center for Statistical Genetics, University of Michigan, Ann Arbor, MI, USA.
26. Department of Clinical Epidemiology, Leiden University Medical Center, Leiden, the Netherlands.
27. MRC/BHF Cardiovascular Epidemiology Unit, Department of Public Health and Primary Care, University of Cambridge, Cambridge, UK.
28. MRC Epidemiology Unit, University of Cambridge School of Clinical Medicine, Cambridge, UK.
29. Department of Twin Research and Genetic Epidemiology, Kings College London, London, UK.
30. NIHR Biomedical Research Centre at Guy's and St Thomas' Foundation Trust, London, UK.
31. Wellcome Trust Sanger Institute, Hinxton, UK.
32. Division of Molecular and Clinical Medicine, School of Medicine, University of Dundee, UK.
33. Department of Pharmacy, COMSATS Institute of Information Technology, Abbottabad, Pakistan.
34. Department of Pathology and Molecular Medicine, McMaster University, Hamilton, Canada.
35. Institut universitaire de cardiologie et de pneumologie de Québec-Université Laval, , Quebec City, Canada.
36. Cardiovascular Research Center and Center for Human Genetic Research, Massachusetts General Hospital, Boston, Massachusetts, MA, USA.
37. University of Groningen, University Medical Center Groningen, Department of Cardiology, Groningen, The Netherlands.
38. University of Lille, Inserm, Centre Hosp. Univ Lille, Institut Pasteur de Lille, UMR1167 - RID-AGE - Risk factors and molecular determinants of aging-related diseases, Epidemiology and Public Health Department, Lille, France.
39. University of Dundee, Ninewells Hospital & Medical School, Dundee, , UK.
40. Wellcome Trust Centre for Human Genetics, University of Oxford, Oxford, UK.
41. Division of Cardiovascular Medicine, Radcliffe Department of Medicine, University of Oxford, Oxford, UK.
42. Usher Institute of Population Health Sciences and Informatics, University of Edinburgh, UK.
43. Department of Internal Medicine, Section Gerontology and Geriatrics, Leiden University Medical Center, Leiden, The Netherlands.
44. Imperial Clinical Trials Unit, Stadium House, 68 Wood Lane, London, UK.
45. School of Medicine, University College Dublin, Ireland.
46. Molecular and Cellular Therapeutics, Royal College of Surgeons in Ireland, Dublin, Ireland.

47. International Centre for Circulatory Health, Imperial College London, London, UK.
48. Center for Statistical Genetics, Dept. of Biostatistics, SPH II, Washington Heights, Ann Arbor, MI, USA.
49. Genetic Epidemiology Unit, Department of Epidemiology, Erasmus MC, Rotterdam, the Netherlands.
50. Center for Complex Disease Genomics, McKusick-Nathans Institute of Genetic Medicine, Johns Hopkins University School of Medicine, Baltimore, MD, USA.
51. Institute of Genetic Medicine, Newcastle University, Newcastle upon Tyne, UK.
52. Sema4, a Mount Sinai venture, Stamford, CT, USA.
53. Division of Genetics and Cell Biology, San Raffaele Scientific Institute, Milano, Italy.
54. Department of Health Sciences, University of Leicester, Leicester, UK.
55. Cardiovascular Health Research Unit, Department of Medicine, University of Washington, Seattle, WA, USA.
56. Institute of Social and Preventive Medicine, University Hospital of Lausanne, Lausanne, Switzerland.
57. Human Genetics Center, School of Public Health, The University of Texas Health Science Center at Houston and Human Genome Sequencing Center, Baylor College of Medicine, One Baylor Plaza, Houston, TX, USA.
58. Department of Biological Psychology, Vrije Universiteit Amsterdam, EMGO+ institute, VU University medical center, Amsterdam, the Netherlands.
59. The Charles Bronfman Institute for Personalized Medicine, Icachn School of Medicine at Mount Sinai, NY, USA.
60. Department of Cardiovascular Sciences, University of Leicester, Leicester, UK.
61. NIHR Leicester Biomedical Research Centre, Glenfield Hospital, Groby Road, Leicester, UK.
62. Department of Medical, Surgical and Health Sciences, University of Trieste, , Trieste, Italy.
63. Medical Genetics Section, Centre for Genomic and Experimental Medicine, Institute of Genetics and Molecular Medicine, University of Edinburgh, Edinburgh, UK.
64. Generation Scotland, Centre for Genomic and Experimental Medicine, University of Edinburgh, Edinburgh, UK.
65. Centre for Global Health Research, Usher Institute of Population Health Sciences and Informatics, University of Edinburgh, Edinburgh, Scotland, UK
66. Lee Kong Chian School of Medicine, Nanyang Technological University, Singapore, Singapore.
67. Department of Cardiology, Ealing Hospital, Middlesex, UK.
68. Imperial College Healthcare NHS Trust, London, UK.
69. Centre for Brain Research, Indian Institute of Science, Bangalore, India.
70. Institute of Genetics and Biophysics "A. Buzzati-Traverso", CNR, Napoli, Italy.
71. IRCCS Neuromed, Pozzilli, Isernia, Italy.
72. Institute for Maternal and Child Health IRCCS Burlo Garofolo, Trieste, Italy.
73. Medical Genomics and Metabolic Genetics Branch, National Human Genome Research Institute, NIH, Bethesda, MD, USA.
74. Centre for Cognitive Ageing and Cognitive Epidemiology, University of Edinburgh, 7 George Square, Edinburgh, UK.
75. Department of Psychology, University of Edinburgh, 7 George Square, Edinburgh, UK.
76. Department of Internal Medicine, Division of Nephrology, University of Groningen, University Medical Center Groningen, Groningen, The Netherlands.

77. Department of Molecular Epidemiology, Leiden University Medical Center, Leiden, the Netherlands.
78. Institute for Biomedicine, Eurac Research, Bolzano, Italy - Affiliated Institute of the University of Lübeck, Lübeck, Germany.
79. Mathematical and Statistical Computing Laboratory, Office of Intramural Research, Center for Information Technology, National Institutes of Health, Bethesda, MD, USA.
80. Department of Internal Medicine B, University Medicine Greifswald, Greifswald, Germany.
81. DZHK (German Centre for Cardiovascular Research), partner site Greifswald, Greifswald, Germany.
82. Cardiology, Department of Medicine, Geneva University Hospital, Geneva, Switzerland.
83. CIBERCV & Cardiovascular Epidemiology and Genetics, IMIM. Dr Aiguader 88, Barcelona, Spain.
84. Faculty of Medicine, Universitat de Vic-Central de Catalunya, Vic, Spain.
85. Department of Immunology, Genetics and Pathology, Uppsala Universitet, Science for Life Laboratory, Uppsala, Sweden.
86. Wellcome Centre for Human Genetics, University of Oxford, Roosevelt Drive, Oxford, UK.
87. Big Data Institute, Li Ka Shing Center for Health for Health Information and Discovery, Oxford University, Old Road, Oxford, UK.
88. Cardiovascular Medicine Unit, Department of Medicine Solna, Karolinska Institutet, Stockholm, Sweden.
89. Centre for Molecular Medicine, L8:03, Karolinska Universitetsjukhuset, Solna, Sweden.
90. Department of Numerical Analysis and Computer Science, Stockholm University, Stockholm, Sweden.
91. Department of Epidemiology, Erasmus MC, Rotterdam, the Netherlands.
92. Department of Public Health and Caring Sciences, Geriatrics, Uppsala, Sweden.
93. Research Unit of Molecular Epidemiology, Helmholtz Zentrum München, German Research Center for Environmental Health, Neuherberg, Germany.
94. Institute of Epidemiology, Helmholtz Zentrum München, German Research Center for Environmental Health, Neuherberg, Germany.
95. German Center for Diabetes Research (DZD e.V.), Neuherberg, Germany.
96. Department of Psychology, School of Social Sciences, Heriot-Watt University, Edinburgh, UK.
97. Faculty of Medicine, University of Iceland, Reykjavik, Iceland.
98. Icelandic Heart Association, Kopavogur, Iceland.
99. The Institute for Translational Genomics and Population Sciences, Department of Pediatrics, LABioMed at Harbor-UCLA Medical Center, Torrance, CA, USA.
100. Intramural Research Program, Laboratory of Epidemiology, Demography, and Biometry, National Institute on Aging, Bethesda, MD, USA.
101. Department of Psychiatry, University of Groningen, University Medical Center Groningen, Groningen, The Netherlands.
102. Department of Public Health Solutions, National Institute for Health and Welfare (THL), Helsinki, Finland.
103. Institute for Molecular Medicine Finland (FIMM), University of Helsinki, Helsinki, Finland.

104. Clinical Division of Neurogeriatrics, Department of Neurology, Medical University of Graz, Graz, Austria.
105. Institute for Medical Informatics, Statistics and Documentation, Medical University of Graz, Graz, Austria.
106. Department of Epidemiology, Harvard T.H. Chan School of Public Health, Boston, MA, USA.
107. National Heart, Lung and Blood Institute's Framingham Heart Study, Framingham, MA, USA.
108. The Population Science Branch, Division of Intramural Research, National Heart Lung and Blood Institute national Institute of Health, Bethesda, MD, USA.
109. Department of Medical Sciences, Molecular Epidemiology and Science for Life Laboratory, Uppsala University, Uppsala, Sweden.
110. Division of Cardiovascular Medicine, Department of Medicine, Stanford University School of Medicine, Stanford, CA USA.
111. Department of Pulmonary Physiology and Sleep, Sir Charles Gairdner Hospital, Hospital Avenue, Nedlands, Australia.
112. School of Medicine and Pharmacology, University of Western Australia.
113. Department of Psychiatry, VU University Medical Center, Amsterdam Neuroscience, Amsterdam, the Netherlands.
114. Biocenter Oulu, University of Oulu, Oulu, Finland.
115. Center For Life-course Health Research, University of Oulu, Oulu Finland.
116. Unit of Primary Care, Oulu University Hospital, Oulu, Oulu, Finland.
117. Hebrew SeniorLife, Harvard Medical School, Boston, MA, USA.
118. Population Sciences Branch, National Heart, Lung and Blood Institute, National Institutes of Health, Bethesda, MD, USA.
119. Department of Cardiology, Leiden University Medical Center, Leiden, the Netherlands.
120. Department of Clinical Physiology, Tampere University Hospital, Tampere, Finland.
121. Department of Clinical Physiology, Finnish Cardiovascular Research Center - Tampere, Faculty of Medicine and Life Sciences, University of Tampere, Tampere, Finland.
122. Broad Institute of the Massachusetts Institute of Technology and Harvard University, Cambridge, MA, USA.
123. Division of Cardiovascular Sciences, Faculty of Biology, Medicine and Health, The University of Manchester, Manchester, UK.
124. Division of Medicine, Manchester University NHS Foundation Trust, Manchester Academic Health Science Centre, Manchester, UK
125. Department of Public Health and Primary Care, Institute of Public Health, University of Cambridge, Cambridge, UK.
126. Data Science Institute and Lancaster Medical School, Lancaster, UK.
127. Department of Public Health, Faculty of Medicine, University of Split, Croatia.
128. National Heart and Lung Institute, Imperial College London, London, UK.
129. Swiss Institute of Bioinformatics, Lausanne, Switzerland.
130. Institute of Biomedicine and Translational Medicine, University of Tartu, Tartu, Estonia.
131. Department of Clinical Chemistry, Fimlab Laboratories, Tampere, Finland.
132. Department of Clinical Chemistry, Finnish Cardiovascular Research Center - Tampere, Faculty of Medicine and Life Sciences, University of Tampere, Tampere, Finland

133. Department of Medical Sciences, Cardiovascular Epidemiology, Uppsala University, Uppsala, Sweden.
134. Program in Medical and Population Genetics, Broad Institute, Cambridge, MA, USA.
135. Division of Public Health Sciences, Wake Forest School of Medicine, Winston-Salem, NC, USA.
136. Mindich Child health Development Institute, The Icahn School of Medicine at Mount Sinai, New York, NY, USA.
137. Department of Psychiatry, Royal College of Surgeons in Ireland, Education and Research Centre, Beaumont Hospital, Dublin, Ireland.
138. University College Dublin, UCD Conway Institute, Centre for Proteome Research, UCD, Belfield, Dublin, Ireland.
139. Institute of Health and Society, Newcastle University, Newcastle upon Tyne, UK.
140. Department of Psychiatry, Amsterdam Public Health and Amsterdam Neuroscience, VU University Medical Center/GGZ inGeest, Amsterdam, The Netherlands.
141. Department of Biostatistics, University of Liverpool, Block F, Waterhouse Building, Liverpool, UK.
142. Department of Epidemiology, Human Genetics and Environmental Sciences, School of Public Health, University of Texas Health Science Center at Houston, Houston, TX, USA.
143. Data Tecnica International, Glen Echo, MD, USA.
144. Laboratory of Neurogenetics, National Institute on Aging, Bethesda, USA.
145. Department of Medicine, Turku University Hospital and University of Turku, Finland.
146. Department of Epidemiology, University of Groningen, University Medical Center Groningen, Groningen, The Netherlands.
147. Interdisciplinary Center Psychopathology and Emotion regulation (ICPE), University of Groningen, University Medical Center Groningen, Groningen, The Netherlands.
148. SGDP Centre, Institute of Psychiatry, Psychology and Neuroscience, King's College London, London, UK.
149. British Heart Foundation Glasgow Cardiovascular Research Centre, Institute of Cardiovascular and Medical Sciences, College of Medical, Veterinary and Life Sciences, University of Glasgow, Glasgow, UK.
150. Department of Medicine, Columbia University Medical Center, New York, NY, USA.
151. Analytic and Translational Genetics Unit, Department of Medicine, Department of Neurology and Department of Psychiatry Massachusetts General Hospital, Boston, MA, USA.
152. The Stanley Center for Psychiatric Research and Program in Medical and Population Genetics, The Broad Institute of MIT and Harvard, Cambridge, MA, USA.
153. University of Tartu, Tartu, Estonia.
154. German Center for Cardiovascular Disease Research (DZHK), partner site Munich, Neuherberg, Germany.
155. Psychiatric hospital “Sveti Ivan”, Zagreb, Croatia.
156. Department of Neurology, General Central Hospital, Bolzano, Italy.
157. Department of Neurology, University of Lübeck, Lübeck, Germany.
158. Department of Clinical Physiology and Nuclear Medicine, Turku University Hospital, Turku, Finland.
159. Research Centre of Applied and Preventive Cardiovascular Medicine, University of Turku, Turku, Finland.
161. Institute of Physiology, University Medicine Greifswald, Karlsburg, Germany.
162. Department of Biostatistics University of Washington, Seattle, WA, USA.

163. Harvard Medical School, Boston MA.
164. Public health, Faculty of Medicine, University of Helsinki, Finland
165. Centre for Global Health Research, Usher Institute of Population Health Sciences and Informatics, University of Edinburgh, Scotland, UK.
166. Gottfried Schatz Research Center for Cell Signaling, Metabolism & Aging, Molecular Biology and Biochemistry, Medical University of Graz, Graz, Austria.
167. The New York Academy of Medicine, New York, NY, USA.
168. Alzheimer Scotland Dementia Research Centre, University of Edinburgh, Edinburgh, UK.
169. Institute of Cardiovascular and Medical Sciences, Faculty of Medicine, University of Glasgow, United Kingdom.
170. Population Health Research Institute, St George's, University of London, London, UK.
171. Department of Genetics, University of Groningen, University Medical Center Groningen, Groningen, The Netherlands.
172. Institute for Community Medicine, University Medicine Greifswald, Greifswald, Germany.
173. Department of Gerontology and Geriatrics, Leiden University Medical Center, Leiden, the Netherlands.
174. Dasman Diabetes Institute, Dasman, Kuwait.
175. Chronic Disease Prevention Unit, National Institute for Health and Welfare, Helsinki, Finland.
176. Department of Public Health, University of Helsinki, Helsinki, Finland.
177. Saudi Diabetes Research Group, King Abdulaziz University, Jeddah, Saudi Arabia.
178. Department of Internal Medicine, Erasmus MC, Rotterdam, the Netherlands.
179. Research Institute for Primordial Prevention of Non-communicable Disease, Isfahan University of Medical Sciences, Isfahan, Iran.
180. Interfaculty Institute for Genetics and Functional Genomics, University Medicine Greifswald, Greifswald, Germany.
181. Department of Internal Medicine, University Hospital, CHUV, Lausanne, Switzerland.
182. Experimental Genetics Division, Sidra Medical and Research Center, Doha, Qatar.
183. Centre for Population Health Sciences, Usher Institute of Population Health Sciences and Informatics, University of Edinburgh, Scotland, UK
184. Department of Biology, Faculty of Medicine, University of Split, Croatia.
185. The National Institute for Health Research Blood and Transplant Research Unit in Donor Health and Genomics, University of Cambridge, UK.
186. Division of Cardiology, University Hospital, Basel, Switzerland.
187. Division of Cardiology, Department of Medicine, McMaster University, Hamilton, Canada.
188. Institute of Genetic and Biomedical Research, National Research Council (CNR), Monserrato, Cagliari, Italy.
189. Department of Biomedical Sciences, University of Sassari, Sassari, Italy.
190. Institute of Clinical Medicine, Internal Medicine, University of Eastern Finland and Kuopio University Hospital, Kuopio, Finland.
191. Laboratory of Cardiovascular Science, NIA/NIH , Baltimore, MD, USA.
192. Department of Public Health and Primary Care, Leiden University Medical Center, Leiden, the Netherlands.
193. Labormedizinisches Zentrum Dr. Risch, Schaan, Liechtenstein.
194. Private University of the Principality of Liechtenstin, Triesen, Liechtenstein.

195. University Insitute of Clinical Chemistry, Inselspital, Bern University Hospital, University of Bern, Bern, Switzerland.
196. Department of Cardiology, University of Groningen, University Medical Center Groningen, Groningen, The Netherlands.
197. Center for Genomic Medicine, Massachusetts General Hospital, Boston, MA, USA.
198. Cardiovascular Research Center, Massachusetts General Hospital, Boston, MA, USA.

201. Cardiovascular Health Research Unit, Departments of Medicine, Epidemiology and Health Services, University of Washington, Seattle, WA, USA.
202. Kaiser Permanente Washington Health Research Institute, Seattle, WA, USA.
203. National Institute for Health Research Imperial Biomedical Research Centre, Imperial College Healthcare NHS Trust and Imperial College London, London, UK.
204. UK Dementia Research Institute (UK DRI) at Imperial College London, London, UK
205. Health Data Research-UK London substantive site, London, U.K

Supplementary Figures

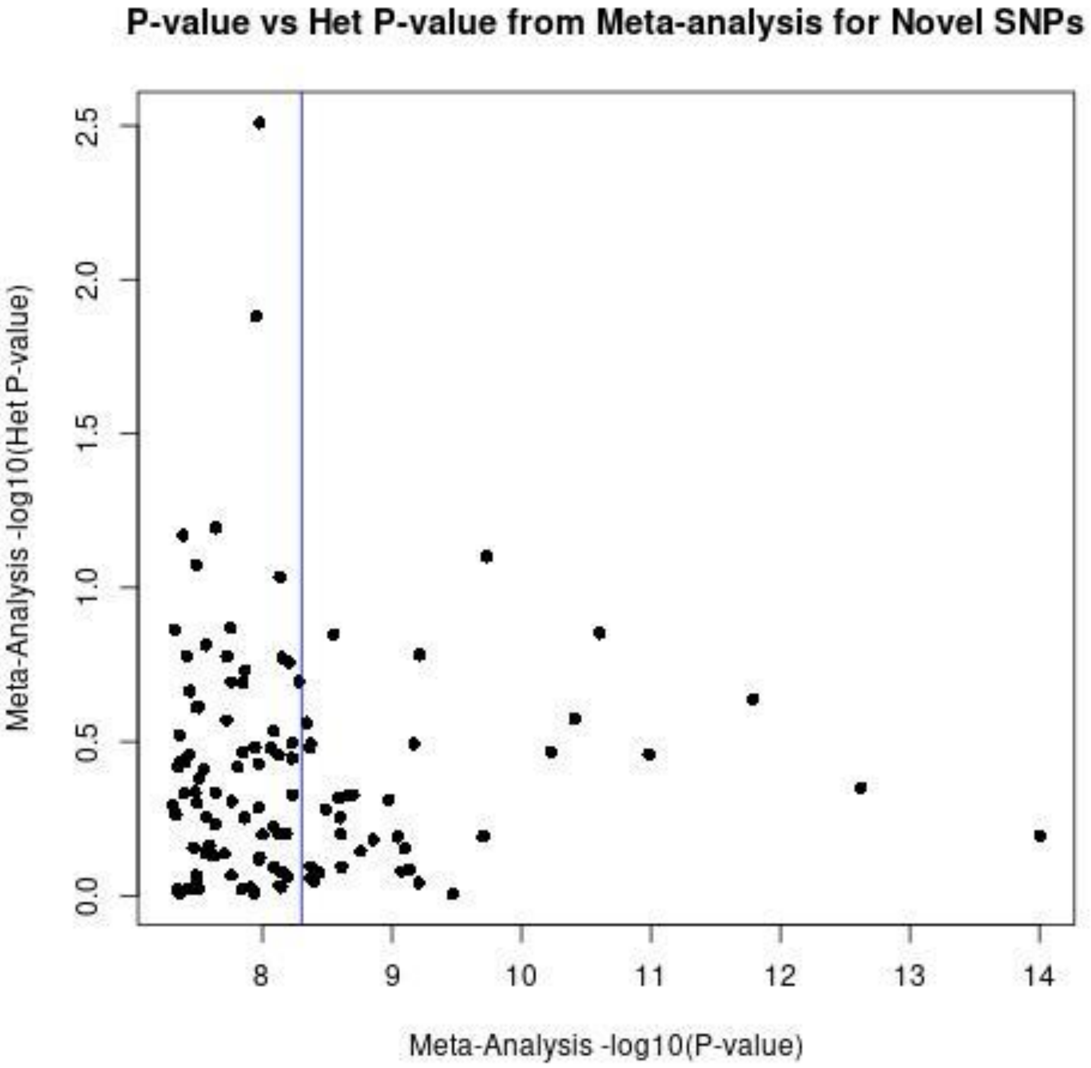

Supplementary Figure 1: Meta-Analysis Heterogeneity P-values of 113 Novel Loci: Plotting the GWAS meta-analysis association p-values from inverse variance-weighted method on the x-axis vs the meta-analysis heterogeneity ("het") p-values from Q statistics on the y-axis, with all p-values on the -log10 scale. The blue vertical reference line indicates the stricter significance p-value threshold for the one-stage GWAS design ($P<5x10^{-9}$). "SNPs" = Single Nucleotide Polymorphisms.

a

9:94252964
rs10991952

Cohort name	beta	95% CI
ICBP	0.196	(0.098 to 0.295)
UKBio	0.167	(0.089 to 0.244)
BioVu	0.247	(0.057 to 0.437)
MVP	0.125	(0.029 to 0.221)
**Fixed effects meta**	**0.169**	**(0.117 to 0.221)**
**Meta p-value**		**1.984E-10**
**I2 Heterogeneity**		**0**
**Q statistic p-value**		**0.641**
**Number of samples**		**1008680**

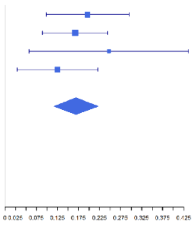

9:132465304
rs3861882

Cohort name	beta	95% CI
ICBP	-0.21	(-0.311 to -0.109)
UKBio	-0.15	(-0.229 to -0.071)
BioVu	-0.241	(-0.433 to -0.05)
MVP	-0.059	(-0.154 to 0.036)
**Fixed effects meta**	**-0.146**	**(-0.199 to -0.094)**
**Meta p-value**		**4.785E-8**
**I2 Heterogeneity**		**45.8**
**Q statistic p-value**		**0.1365**
**Number of samples**		**1007030**

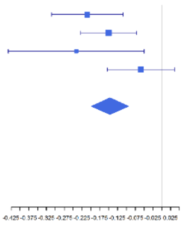

10:17266389
rs10904910

Cohort name	beta	95% CI
ICBP	0.178	(0.08 to 0.275)
UKBio	0.164	(0.087 to 0.241)
BioVu	0.009	(-0.18 to 0.198)
MVP	0.158	(0.066 to 0.25)
**Fixed effects meta**	**0.155**	**(0.104 to 0.206)**
**Meta p-value**		**2.559E-9**
**I2 Heterogeneity**		**0**
**Q statistic p-value**		**0.4809**
**Number of samples**		**1009750**

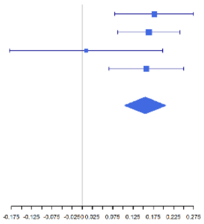

11:19736996
rs1319701

Cohort name	beta	95% CI
ICBP	0.086	(-0.006 to 0.177)
UKBio	0.14	(0.069 to 0.211)
BioVu	0.232	(0.059 to 0.405)
MVP	0.146	(0.061 to 0.232)
**Fixed effects meta**	**0.134**	**(0.087 to 0.182)**
**Meta p-value**		**3.194E-8**
**I2 Heterogeneity**		**0**
**Q statistic p-value**		**0.4994**
**Number of samples**		**1008750**

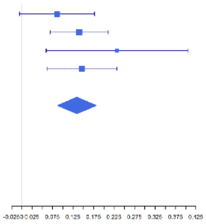

11:66325484
rs61890399

Cohort name	beta	95% CI
ICBP	-0.251	(-0.43 to -0.071)
UKBio	-0.233	(-0.348 to -0.119)
BioVu	-0.141	(-0.458 to 0.176)
MVP	-0.275	(-0.417 to -0.134)
**Fixed effects meta**	**-0.244**	**(-0.326 to -0.163)**
**Meta p-value**		**4.022E-9**
**I2 Heterogeneity**		**0**
**Q statistic p-value**		**0.8955**
**Number of samples**		**1009760**

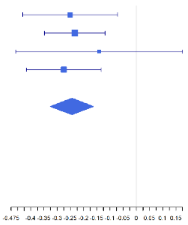

11:113655696
rs17542254

Cohort name	beta	95% CI
ICBP	0.108	(0.006 to 0.209)
UKBio	0.182	(0.104 to 0.261)
BioVu	0.267	(0.075 to 0.46)
MVP	0.112	(0.015 to 0.209)
**Fixed effects meta**	**0.148**	**(0.095 to 0.201)**
**Meta p-value**		**3.983E-8**
**I2 Heterogeneity**		**4.9**
**Q statistic p-value**		**0.3682**
**Number of samples**		**1008640**

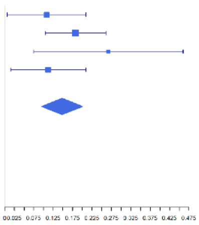

12:51355243
rs76637716

Cohort name	beta	95% CI
ICBP	-0.451	(-0.687 to -0.214)
UKBio	-0.303	(-0.456 to -0.149)
BioVu	-0.14	(-0.556 to 0.277)
MVP	-0.346	(-0.536 to -0.157)
**Fixed effects meta**	**-0.337**	**(-0.445 to -0.228)**
**Meta p-value**		**1.187E-9**
**I2 Heterogeneity**		**0**
**Q statistic p-value**		**0.5943**
**Number of samples**		**1010530**

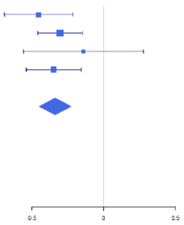

12:120124578
rs278123

Cohort name	beta	95% CI
ICBP	0.102	(0.006 to 0.197)
UKBio	0.202	(0.125 to 0.278)
BioVu	0.181	(-0.008 to 0.37)
MVP	0.096	(0.003 to 0.189)
**Fixed effects meta**	**0.142**	**(0.091 to 0.193)**
**Meta p-value**		**4.366E-8**
**I2 Heterogeneity**		**18.1**
**Q statistic p-value**		**0.3002**
**Number of samples**		**1017400**

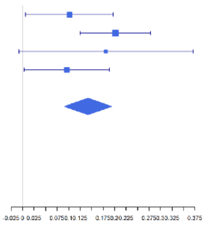

4:153006312
rs7665985

Cohort name	beta	95% CI
ICBP	-0.202	(-0.3 to -0.104)
UKBio	-0.169	(-0.243 to -0.094)
BioVu	-0.107	(-0.29 to 0.076)
MVP	-0.058	(-0.149 to 0.033)
**Fixed effects meta**	**-0.141**	**(-0.191 to -0.091)**
**Meta p-value**		**3.845E-8**
**I2 Heterogeneity**		**40.9**
**Q statistic p-value**		**0.1665**
**Number of samples**		**998915**

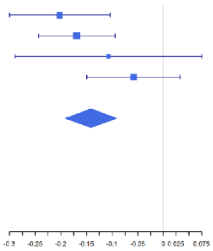

5:39444718
rs13162174

Cohort name	beta	95% CI
ICBP	-0.159	(-0.251 to -0.066)
UKBio	-0.115	(-0.187 to -0.042)
BioVu	-0.159	(-0.337 to 0.018)
MVP	-0.159	(-0.245 to -0.072)
**Fixed effects meta**	**-0.143**	**(-0.191 to -0.095)**
**Meta p-value**		**6.452E-9**
**I2 Heterogeneity**		**0**
**Q statistic p-value**		**0.8628**
**Number of samples**		**1009750**

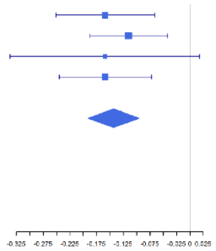

7:156990554
rs2286130

Cohort name	beta	95% CI
ICBP	-0.07	(-0.175 to 0.035)
UKBio	-0.232	(-0.315 to -0.149)
BioVu	-0.219	(-0.422 to -0.016)
MVP	-0.164	(-0.263 to -0.065)
**Fixed effects meta**	**-0.167**	**(-0.222 to -0.112)**
**Meta p-value**		**2.819E-9**
**I2 Heterogeneity**		**45**
**Q statistic p-value**		**0.1416**
**Number of samples**		**1008640**

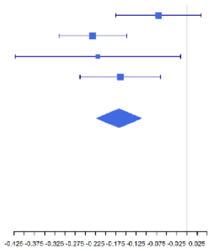

8:1212030
rs11136373

Cohort name	beta	95% CI
ICBP	0.099	(-0.004 to 0.203)
UKBio	0.199	(0.125 to 0.273)
BioVu	0.119	(-0.06 to 0.298)
MVP	0.107	(0.018 to 0.195)
**Fixed effects meta**	**0.141**	**(0.09 to 0.191)**
**Meta p-value**		**4.403E-8**
**I2 Heterogeneity**		**4.6**
**Q statistic p-value**		**0.3701**
**Number of samples**		**1003140**

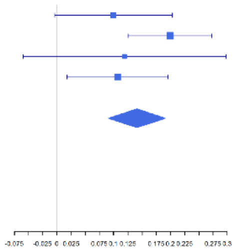

8:57153503
rs11988716

Cohort name	beta	95% CI
ICBP	0.184	(0.045 to 0.324)
UKBio	0.248	(0.142 to 0.354)
BioVu	0.248	(-0.004 to 0.501)
MVP	0.181	(0.059 to 0.303)
**Fixed effects meta**	**0.211**	**(0.141 to 0.281)**
**Meta p-value**		**3.624E-9**
**I2 Heterogeneity**		**0**
**Q statistic p-value**		**0.8403**
**Number of samples**		**1009760**

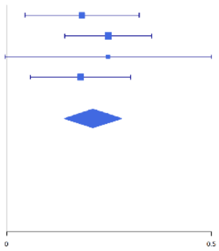

8:146130326
rs2978398

Cohort name	beta	95% CI
ICBP	-0.197	(-0.291 to -0.102)
UKBio	-0.128	(-0.2 to -0.055)
BioVu	-0.203	(-0.394 to -0.012)
MVP	-0.132	(-0.219 to -0.045)
**Fixed effects meta**	**-0.152**	**(-0.201 to -0.104)**
**Meta p-value**		**9.06E-10**
**I2 Heterogeneity**		**0**
**Q statistic p-value**		**0.6445**
**Number of samples**		**1006830**

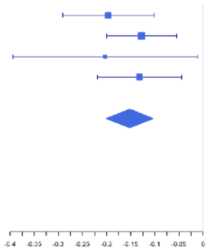

9:27230388
rs9886857

Cohort name	beta	95% CI
ICBP	-0.164	(-0.288 to -0.041)
UKBio	-0.21	(-0.31 to -0.111)
BioVu	-0.153	(-0.396 to 0.089)
MVP	-0.189	(-0.313 to -0.065)
**Fixed effects meta**	**-0.187**	**(-0.253 to -0.121)**
**Meta p-value**		**3.127E-8**
**I2 Heterogeneity**		**0**
**Q statistic p-value**		**0.9442**
**Number of samples**		**1017400**

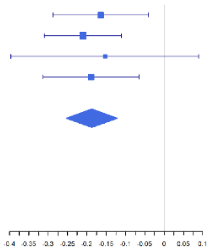

9:83432105
rs2224858

Cohort name	beta	95% CI
ICBP	0.095	(-0.02 to 0.209)
UKBio	0.159	(0.068 to 0.25)
BioVu	0.39	(0.165 to 0.616)
MVP	0.234	(0.124 to 0.344)
**Fixed effects meta**	**0.179**	**(0.118 to 0.239)**
**Meta p-value**		**7.374E-9**
**I2 Heterogeneity**		**53.4**
**Q statistic p-value**		**0.09224**
**Number of samples**		**1017400**

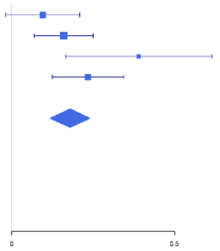

2:105205551
rs6729623

Cohort name	beta	95% CI
ICBP	-0.123	(-0.215 to -0.032)
UKBio	-0.176	(-0.247 to -0.105)
BioVu	-0.244	(-0.423 to -0.065)
MVP	-0.089	(-0.174 to -0.003)
**Fixed effects meta**	**-0.141**	**(-0.188 to -0.094)**
**Meta p-value**		**5.88E-9**
**I2 Heterogeneity**		**14.6**
**Q statistic p-value**		**0.3191**
**Number of samples**		**1009750**

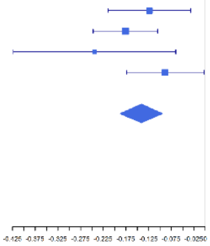

2:125429006
rs11123059

Cohort name	beta	95% CI
ICBP	0.14	(0.049 to 0.23)
UKBio	0.133	(0.061 to 0.205)
BioVu	0.176	(0.002 to 0.35)
MVP	0.12	(0.033 to 0.207)
**Fixed effects meta**	**0.134**	**(0.087 to 0.182)**
**Meta p-value**		**3.467E-8**
**I2 Heterogeneity**		**0**
**Q statistic p-value**		**0.9544**
**Number of samples**		**1009750**

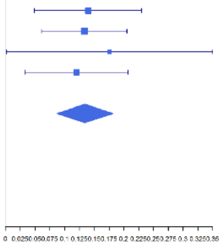

2:127839534
rs11690153

Cohort name	beta	95% CI
ICBP	0.131	(0.015 to 0.248)
UKBio	0.179	(0.087 to 0.271)
BioVu	0.356	(0.133 to 0.58)
MVP	0.159	(0.041 to 0.277)
**Fixed effects meta**	**0.174**	**(0.111 to 0.236)**
**Meta p-value**		**4.475E-8**
**I2 Heterogeneity**		**2.2**
**Q statistic p-value**		**0.3814**
**Number of samples**		**1009750**

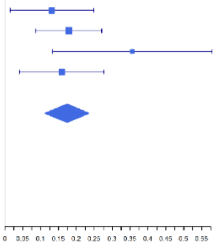

2:128822702
rs13022015

Cohort name	beta	95% CI
ICBP	-0.125	(-0.242 to -0.008)
UKBio	-0.228	(-0.32 to -0.136)
BioVu	-0.255	(-0.479 to -0.031)
MVP	-0.129	(-0.24 to -0.019)
**Fixed effects meta**	**-0.173**	**(-0.234 to -0.112)**
**Meta p-value**		**3.083E-8**
**I2 Heterogeneity**		**0**
**Q statistic p-value**		**0.417**
**Number of samples**		**1000480**

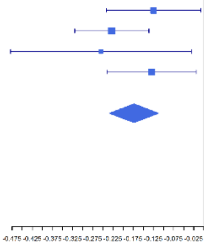

3:16363689
rs538180

Cohort name	beta	95% CI
ICBP	-0.133	(-0.225 to -0.042)
UKBio	-0.162	(-0.235 to -0.09)
BioVu	-0.085	(-0.264 to 0.093)
MVP	-0.168	(-0.255 to -0.081)
**Fixed effects meta**	**-0.151**	**(-0.199 to -0.103)**
**Meta p-value**		**8.467E-10**
**I2 Heterogeneity**		**0**
**Q statistic p-value**		**0.833**
**Number of samples**		**1008750**

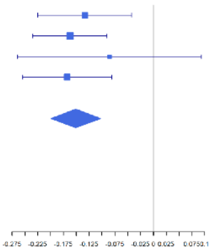

3:43992455
rs9877020

Cohort name	beta	95% CI
ICBP	0.19	(0.065 to 0.315)
UKBio	0.224	(0.128 to 0.319)
BioVu	0.086	(-0.151 to 0.322)
MVP	0.151	(0.032 to 0.27)
**Fixed effects meta**	**0.184**	**(0.119 to 0.249)**
**Meta p-value**		**2.57E-8**
**I2 Heterogeneity**		**0**
**Q statistic p-value**		**0.6876**
**Number of samples**		**1009750**

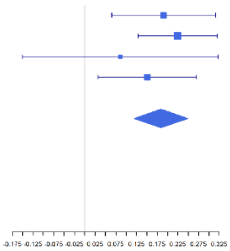

3:71607861
rs844218

Cohort name	beta	95% CI
ICBP	-0.089	(-0.187 to 0.008)
UKBio	-0.135	(-0.212 to -0.058)
BioVu	-0.173	(-0.361 to 0.014)
MVP	-0.198	(-0.29 to -0.105)
**Fixed effects meta**	**-0.143**	**(-0.195 to -0.092)**
**Meta p-value**		**4.027E-8**
**I2 Heterogeneity**		**0**
**Q statistic p-value**		**0.4646**
**Number of samples**		**1009750**

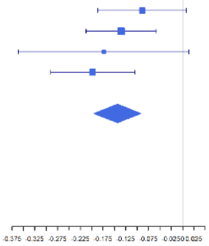

4:84452950
rs10018970

Cohort name	beta	95% CI
ICBP	-0.171	(-0.262 to -0.081)
UKBio	-0.103	(-0.174 to -0.032)
BioVu	-0.27	(-0.443 to -0.097)
MVP	-0.13	(-0.216 to -0.045)
**Fixed effects meta**	**-0.142**	**(-0.189 to -0.094)**
**Meta p-value**		**4.243E-9**
**I2 Heterogeneity**		**14.1**
**Q statistic p-value**		**0.3216**
**Number of samples**		**1006740**

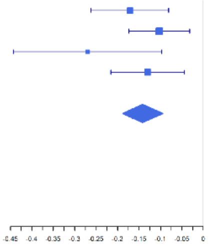

1:61877445
rs2092867

Cohort name	beta	95% CI
ICBP	0.121	(0.026 to 0.217)
UKBio	0.164	(0.09 to 0.238)
BioVu	0.15	(-0.03 to 0.331)
MVP	0.142	(0.054 to 0.23)
**Fixed effects meta**	**0.145**	**(0.096 to 0.194)**
**Meta p-value**		**7.403E-9**
**I2 Heterogeneity**		**0**
**Q statistic p-value**		**0.9279**
**Number of samples**		**1001040**

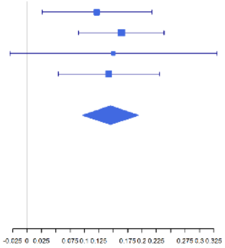

1:93524045
rs12145044

Cohort name	beta	95% CI
ICBP	-0.53	(-0.759 to -0.301)
UKBio	-0.235	(-0.397 to -0.072)
BioVu	-0.609	(-1.027 to -0.192)
MVP	-0.182	(-0.417 to 0.053)
**Fixed effects meta**	**-0.328**	**(-0.445 to -0.211)**
**Meta p-value**		**4.103E-8**
**I2 Heterogeneity**		**58**
**Q statistic p-value**		**0.06769**
**Number of samples**		**1009760**

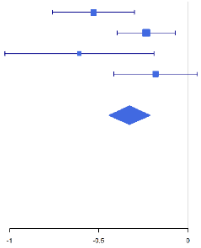

1:166023209
rs4573493

Cohort name	beta	95% CI
ICBP	-0.156	(-0.247 to -0.065)
UKBio	-0.175	(-0.246 to -0.103)
BioVu	-0.2	(-0.374 to -0.025)
MVP	-0.057	(-0.144 to 0.03)
**Fixed effects meta**	**-0.138**	**(-0.186 to -0.09)**
**Meta p-value**		**1.415E-8**
**I2 Heterogeneity**		**34.7**
**Q statistic p-value**		**0.2038**
**Number of samples**		**1007630**

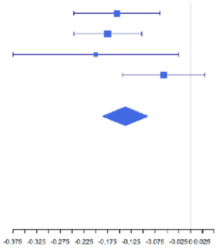

1:193271526
rs817140

Cohort name	beta	95% CI
ICBP	0.106	(0.007 to 0.206)
UKBio	0.199	(0.12 to 0.279)
BioVu	0.204	(0.009 to 0.398)
MVP	0.154	(0.058 to 0.251)
**Fixed effects meta**	**0.161**	**(0.108 to 0.214)**
**Meta p-value**		**2.523E-9**
**I2 Heterogeneity**		**0**
**Q statistic p-value**		**0.5583**
**Number of samples**		**1009750**

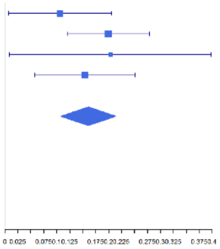

2:12994692
rs6723772

Cohort name	beta	95% CI
ICBP	-0.326	(-0.474 to -0.177)
UKBio	-0.159	(-0.274 to -0.043)
BioVu	-0.158	(-0.437 to 0.121)
MVP	-0.241	(-0.392 to -0.091)
**Fixed effects meta**	**-0.227**	**(-0.306 to -0.148)**
**Meta p-value**		**1.552E-8**
**I2 Heterogeneity**		**1.9**
**Q statistic p-value**		**0.3829**
**Number of samples**		**1009750**

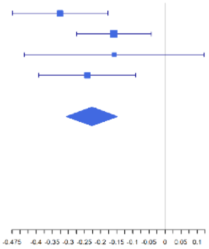

2:32620888
rs10172510

Cohort name	beta	95% CI
ICBP	0.114	(0.023 to 0.204)
UKBio	0.159	(0.088 to 0.231)
BioVu	0.251	(0.075 to 0.427)
MVP	0.095	(0.01 to 0.179)
**Fixed effects meta**	**0.134**	**(0.087 to 0.182)**
**Meta p-value**		**2.849E-8**
**I2 Heterogeneity**		**0.6**
**Q statistic p-value**		**0.3889**
**Number of samples**		**1009760**

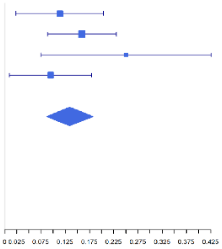

2:39061959
rs56350535

Cohort name	beta	95% CI
ICBP	-0.214	(-0.355 to -0.074)
UKBio	-0.225	(-0.335 to -0.115)
BioVu	-0.145	(-0.418 to 0.128)
MVP	-0.192	(-0.327 to -0.056)
**Fixed effects meta**	**-0.207**	**(-0.281 to -0.133)**
**Meta p-value**		**3.847E-8**
**I2 Heterogeneity**		**0**
**Q statistic p-value**		**0.9534**
**Number of samples**		**1009760**

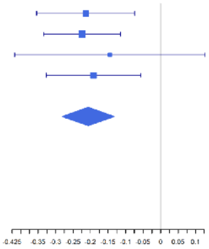

2:54738168
rs75243511

Cohort name	beta	95% CI
ICBP	0.264	(0.041 to 0.486)
UKBio	0.39	(0.22 to 0.559)
BioVu	0.021	(-0.409 to 0.451)
MVP	0.427	(0.205 to 0.649)
**Fixed effects meta**	**0.338**	**(0.221 to 0.455)**
**Meta p-value**		**1.417E-8**
**I2 Heterogeneity**		**10.1**
**Q statistic p-value**		**0.3426**
**Number of samples**		**1009760**

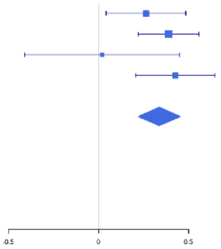

13:40671137
rs190533862

Cohort name	beta	95% CI
ICBP	0.422	(0.214 to 0.63)
UKBio	0.143	(-0.005 to 0.291)
BioVu	0.397	(0.026 to 0.768)
MVP	0.34	(0.16 to 0.519)
**Fixed effects meta**	**0.288**	**(0.186 to 0.389)**
**Meta p-value**		**2.754E-8**
**I2 Heterogeneity**		**43**
**Q statistic p-value**		**0.1538**
**Number of samples**		**1010960**

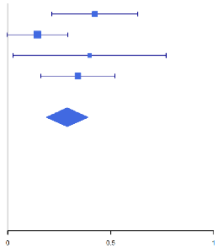

13:54264395
rs9596839

Cohort name	beta	95% CI
ICBP	-0.104	(-0.203 to -0.005)
UKBio	-0.178	(-0.257 to -0.099)
BioVu	-0.264	(-0.454 to -0.074)
MVP	-0.149	(-0.244 to -0.054)
**Fixed effects meta**	**-0.155**	**(-0.208 to -0.103)**
**Meta p-value**		**5.865E-9**
**I2 Heterogeneity**		**0**
**Q statistic p-value**		**0.4706**
**Number of samples**		**1009140**

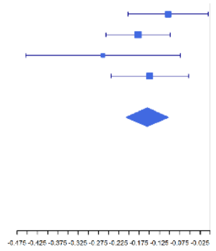

14:71352648
rs36563

Cohort name	beta	95% CI
ICBP	0.224	(0.098 to 0.349)
UKBio	0.225	(0.125 to 0.325)
BioVu	0.15	(-0.086 to 0.386)
MVP	0.183	(0.067 to 0.298)
**Fixed effects meta**	**0.206**	**(0.141 to 0.271)**
**Meta p-value**		**6.213E-10**
**I2 Heterogeneity**		**0**
**Q statistic p-value**		**0.908**
**Number of samples**		**1017410**

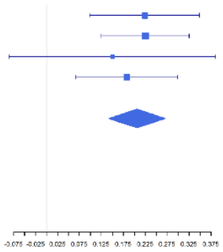

14:88825415
rs7160184

Cohort name	beta	95% CI
ICBP	-0.16	(-0.312 to -0.009)
UKBio	-0.282	(-0.407 to -0.158)
BioVu	-0.52	(-0.819 to -0.221)
MVP	-0.202	(-0.349 to -0.054)
**Fixed effects meta**	**-0.241**	**(-0.322 to -0.16)**
**Meta p-value**		**6.221E-9**
**I2 Heterogeneity**		**39.5**
**Q statistic p-value**		**0.1749**
**Number of samples**		**1017400**

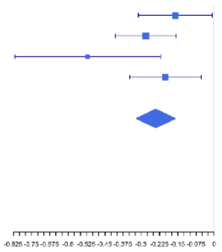

17:81036344
rs9675039

Cohort name	beta	95% CI
ICBP	0.226	(0.124 to 0.327)
UKBio	0.123	(0.049 to 0.197)
BioVu	0.106	(-0.072 to 0.284)
MVP	0.121	(0.033 to 0.21)
**Fixed effects meta**	**0.146**	**(0.096 to 0.196)**
**Meta p-value**		**1.067E-8**
**I2 Heterogeneity**		**4.1**
**Q statistic p-value**		**0.3721**
**Number of samples**		**985779**

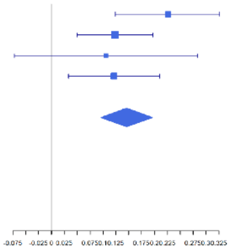

18:7131618
rs880132

Cohort name	beta	95% CI
ICBP	-0.195	(-0.297 to -0.094)
UKBio	-0.148	(-0.222 to -0.074)
BioVu	-0.345	(-0.54 to -0.149)
MVP	-0.178	(-0.281 to -0.076)
**Fixed effects meta**	**-0.182**	**(-0.235 to -0.13)**
**Meta p-value**		**1.036E-11**
**I2 Heterogeneity**		**9.2**
**Q statistic p-value**		**0.3469**
**Number of samples**		**1016400**

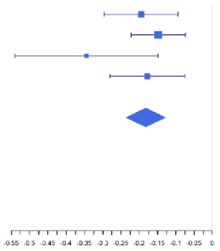

18:44040660
rs17766830

Cohort name	beta	95% CI
ICBP	0.147	(0.041 to 0.252)
UKBio	0.172	(0.091 to 0.253)
BioVu	0.159	(-0.056 to 0.374)
MVP	0.177	(0.065 to 0.289)
**Fixed effects meta**	**0.165**	**(0.108 to 0.222)**
**Meta p-value**		**1.158E-8**
**I2 Heterogeneity**		**0**
**Q statistic p-value**		**0.9804**
**Number of samples**		**1016400**

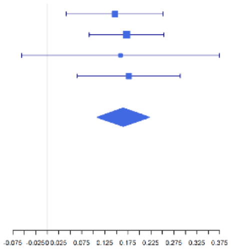

18:46461487
rs72917789

Cohort name	beta	95% CI
ICBP	-0.427	(-0.615 to -0.24)
UKBio	-0.208	(-0.349 to -0.067)
BioVu	-0.222	(-0.561 to 0.118)
MVP	-0.248	(-0.419 to -0.077)
**Fixed effects meta**	**-0.277**	**(-0.372 to -0.182)**
**Meta p-value**		**1.141E-8**
**I2 Heterogeneity**		**12.6**
**Q statistic p-value**		**0.3294**
**Number of samples**		**1002320**

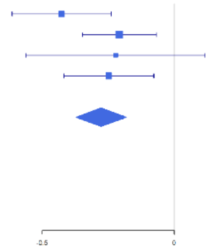

b

11:108350451
rs11212666

Cohort name	beta	95% CI
ICBP	0.083	(0.029 to 0.137)
UKBio	0.088	(0.047 to 0.128)
BioVu	0.05	(-0.104 to 0.204)
MVP	0.091	(0.021 to 0.162)
**Fixed effects meta**	**0.085**	**(0.055 to 0.116)**
**Meta p-value**		**4.39E-8**
**I2 Heterogeneity**		**0**
**Q statistic p-value**		**0.9706**
**Number of samples**		**1027900**

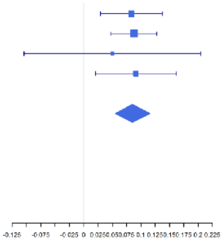

11:124619407
rs11604175

Cohort name	beta	95% CI
ICBP	0.097	(0.036 to 0.157)
UKBio	0.111	(0.065 to 0.158)
BioVu	0.216	(0.053 to 0.379)
MVP	0.074	(0.001 to 0.148)
**Fixed effects meta**	**0.104**	**(0.07 to 0.138)**
**Meta p-value**		**1.992E-9**
**I2 Heterogeneity**		**0**
**Q statistic p-value**		**0.4698**
**Number of samples**		**1028900**

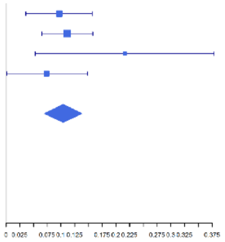

11:128769876
rs3765618

Cohort name	beta	95% CI
ICBP	-0.106	(-0.211 to -0.002)
UKBio	-0.216	(-0.286 to -0.146)
BioVu	-0.054	(-0.314 to 0.205)
MVP	-0.213	(-0.326 to -0.101)
**Fixed effects meta**	**-0.18**	**(-0.234 to -0.127)**
**Meta p-value**		**3.867E-11**
**I2 Heterogeneity**		**24.2**
**Q statistic p-value**		**0.2662**
**Number of samples**		**1025020**

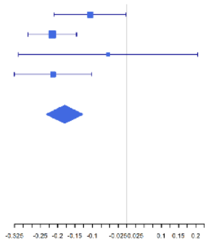

12:52418075
rs1732235

Cohort name	beta	95% CI
ICBP	-0.064	(-0.118 to -0.01)
UKBio	-0.077	(-0.117 to -0.037)
BioVu	-0.073	(-0.218 to 0.073)
MVP	-0.141	(-0.204 to -0.078)
**Fixed effects meta**	**-0.087**	**(-0.116 to -0.057)**
**Meta p-value**		**8.177E-9**
**I2 Heterogeneity**		**19.5**
**Q statistic p-value**		**0.2925**
**Number of samples**		**1027790**

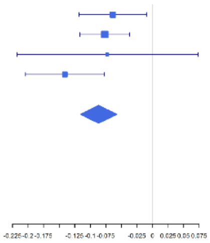

13:38249726
rs56312513

Cohort name	beta	95% CI
ICBP	0.065	(0.005 to 0.125)
UKBio	0.16	(0.114 to 0.206)
BioVu	0.028	(-0.137 to 0.192)
MVP	0.03	(-0.044 to 0.103)
**Fixed effects meta**	**0.098**	**(0.064 to 0.132)**
**Meta p-value**		**1.118E-8**
**I2 Heterogeneity**		**72.1**
**Q statistic p-value**		**0.01317**
**Number of samples**		**1028900**

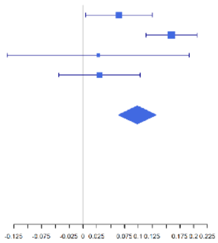

14:21841154
rs7350752

Cohort name	beta	95% CI
ICBP	-0.201	(-0.286 to -0.116)
UKBio	-0.119	(-0.18 to -0.059)
BioVu	-0.078	(-0.297 to 0.141)
**Fixed effects meta**	**-0.146**	**(-0.198 to -0.095)**
**Meta p-value**		**1.886E-8**
**I2 Heterogeneity**		**23.7**
**Q statistic p-value**		**0.2695**
**Number of samples**		**807694**

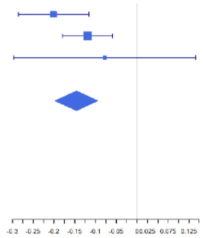

14:59900020
rs2774052

Cohort name	beta	95% CI
ICBP	0.081	(0.028 to 0.134)
UKBio	0.07	(0.029 to 0.11)
BioVu	0.105	(-0.038 to 0.247)
MVP	0.13	(0.064 to 0.195)
**Fixed effects meta**	**0.087**	**(0.057 to 0.116)**
**Meta p-value**		**1.065E-8**
**I2 Heterogeneity**		**0**
**Q statistic p-value**		**0.5171**
**Number of samples**		**1027890**

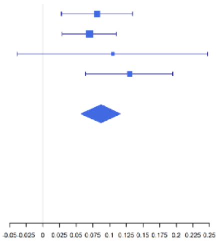

14:71874638
rs2041330

Cohort name	beta	95% CI
ICBP	0.065	(0.012 to 0.119)
UKBio	0.104	(0.063 to 0.144)
BioVu	0.057	(-0.087 to 0.201)
MVP	0.075	(0.009 to 0.141)
**Fixed effects meta**	**0.084**	**(0.054 to 0.114)**
**Meta p-value**		**3.419E-8**
**I2 Heterogeneity**		**0**
**Q statistic p-value**		**0.6981**
**Number of samples**		**1027890**

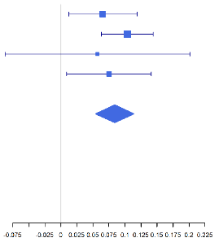

6:10034452
rs9477605

Cohort name	beta	95% CI
ICBP	0.076	(0.021 to 0.132)
UKBio	0.086	(0.044 to 0.128)
BioVu	0.128	(-0.021 to 0.277)
MVP	0.097	(0.03 to 0.163)
**Fixed effects meta**	**0.087**	**(0.056 to 0.118)**
**Meta p-value**		**3.219E-8**
**I2 Heterogeneity**		**0**
**Q statistic p-value**		**0.9171**
**Number of samples**		**1027790**

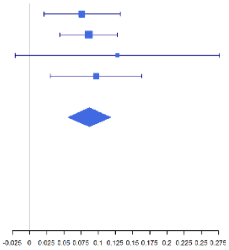

6:17477425
rs9370995

Cohort name	beta	95% CI
ICBP	0.11	(0.056 to 0.164)
UKBio	0.08	(0.039 to 0.12)
BioVu	0.129	(-0.016 to 0.274)
MVP	0.045	(-0.02 to 0.111)
**Fixed effects meta**	**0.084**	**(0.054 to 0.114)**
**Meta p-value**		**3.312E-8**
**I2 Heterogeneity**		**0**
**Q statistic p-value**		**0.4601**
**Number of samples**		**1028900**

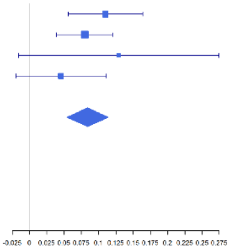

6:100629078
rs57989773

Cohort name	beta	95% CI
ICBP	-0.079	(-0.148 to -0.011)
UKBio	-0.168	(-0.215 to -0.12)
BioVu	-0.048	(-0.224 to 0.128)
MVP	-0.096	(-0.173 to -0.019)
**Fixed effects meta**	**-0.123**	**(-0.159 to -0.087)**
**Meta p-value**		**2.494E-11**
**I2 Heterogeneity**		**45.1**
**Q statistic p-value**		**0.1406**
**Number of samples**		**1026780**

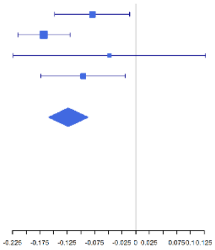

6:109625797
rs1546722

Cohort name	beta	95% CI
ICBP	-0.066	(-0.12 to -0.013)
UKBio	-0.116	(-0.156 to -0.076)
BioVu	-0.083	(-0.225 to 0.06)
MVP	-0.054	(-0.117 to 0.008)
**Fixed effects meta**	**-0.087**	**(-0.116 to -0.057)**
**Meta p-value**		**7.461E-9**
**I2 Heterogeneity**		**8.7**
**Q statistic p-value**		**0.3497**
**Number of samples**		**1028900**

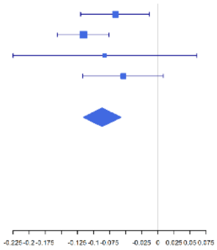

8:49391836
rs10087280

Cohort name	beta	95% CI
ICBP	-0.081	(-0.152 to -0.011)
UKBio	-0.178	(-0.232 to -0.125)
BioVu	-0.211	(-0.401 to -0.022)
MVP	-0.074	(-0.157 to 0.01)
**Fixed effects meta**	**-0.127**	**(-0.166 to -0.088)**
**Meta p-value**		**1.859E-10**
**I2 Heterogeneity**		**55.8**
**Q statistic p-value**		**0.07922**
**Number of samples**		**1028900**

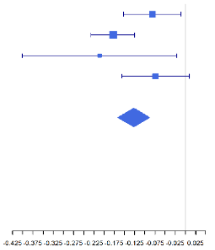

8:134229535
rs6982341

Cohort name	beta	95% CI
ICBP	0.047	(-0.006 to 0.1)
UKBio	0.114	(0.074 to 0.155)
BioVu	0.014	(-0.129 to 0.158)
MVP	0.095	(0.031 to 0.159)
**Fixed effects meta**	**0.085**	**(0.056 to 0.115)**
**Meta p-value**		**1.741E-8**
**I2 Heterogeneity**		**35**
**Q statistic p-value**		**0.2025**
**Number of samples**		**1028900**

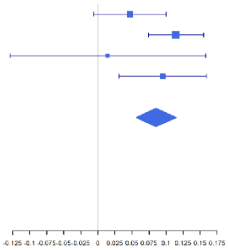

9:129643296
rs10819246

Cohort name	beta	95% CI
ICBP	0.136	(0.046 to 0.227)
UKBio	0.163	(0.096 to 0.23)
BioVu	0.379	(0.135 to 0.622)
MVP	0.175	(0.067 to 0.284)
**Fixed effects meta**	**0.166**	**(0.116 to 0.216)**
**Meta p-value**		**5.909E-11**
**I2 Heterogeneity**		**10.4**
**Q statistic p-value**		**0.3409**
**Number of samples**		**1020640**

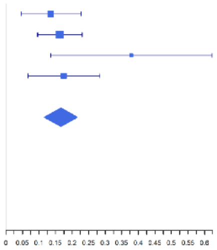

11:96151677
rs61909958

Cohort name	beta	95% CI
ICBP	-0.164	(-0.236 to -0.092)
UKBio	-0.105	(-0.156 to -0.053)
BioVu	-0.272	(-0.462 to -0.082)
MVP	-0.075	(-0.162 to 0.013)
**Fixed effects meta**	**-0.123**	**(-0.162 to -0.084)**
**Meta p-value**		**6.114E-10**
**I2 Heterogeneity**		**41.1**
**Q statistic p-value**		**0.1654**
**Number of samples**		**1027890**

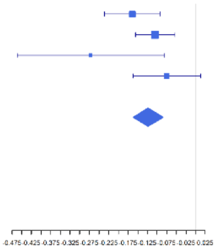

3:136692308
rs73231988

Cohort name	beta	95% CI
ICBP	0.117	(0.034 to 0.2)
UKBio	0.14	(0.075 to 0.204)
BioVu	0.135	(-0.092 to 0.362)
MVP	0.153	(0.054 to 0.252)
**Fixed effects meta**	**0.135**	**(0.089 to 0.182)**
**Meta p-value**		**1.448E-8**
**I2 Heterogeneity**		**0**
**Q statistic p-value**		**0.9556**
**Number of samples**		**1028900**

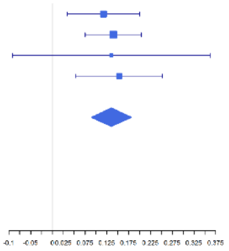

4:72002332
rs6822301

Cohort name	beta	95% CI
ICBP	0.101	(0.033 to 0.169)
UKBio	0.096	(0.046 to 0.146)
BioVu	0.04	(-0.147 to 0.227)
MVP	0.165	(0.08 to 0.25)
**Fixed effects meta**	**0.108**	**(0.07 to 0.146)**
**Meta p-value**		**1.728E-8**
**I2 Heterogeneity**		**0**
**Q statistic p-value**		**0.4933**
**Number of samples**		**1027890**

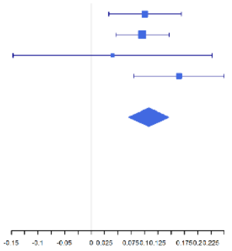

4:152163489
rs7671332

Cohort name	beta	95% CI
ICBP	0.191	(0.042 to 0.34)
UKBio	0.271	(0.17 to 0.373)
BioVu	0.145	(-0.221 to 0.512)
MVP	0.219	(0.048 to 0.389)
**Fixed effects meta**	**0.233**	**(0.155 to 0.311)**
**Meta p-value**		**4.265E-9**
**I2 Heterogeneity**		**0**
**Q statistic p-value**		**0.8033**
**Number of samples**		**1027790**

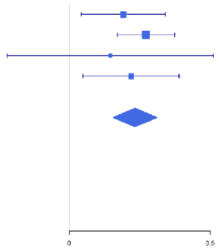

4:187818466
rs9685837

Cohort name	beta	95% CI
ICBP	-0.066	(-0.124 to -0.007)
UKBio	-0.101	(-0.144 to -0.057)
BioVu	-0.088	(-0.241 to 0.065)
MVP	-0.115	(-0.186 to -0.044)
**Fixed effects meta**	**-0.092**	**(-0.124 to -0.06)**
**Meta p-value**		**1.983E-8**
**I2 Heterogeneity**		**0**
**Q statistic p-value**		**0.7305**
**Number of samples**		**1025880**

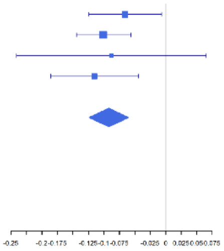

5:38616887
rs172906

Cohort name	beta	95% CI
ICBP	0.113	(0.051 to 0.174)
UKBio	0.096	(0.054 to 0.138)
BioVu	0.094	(-0.062 to 0.251)
MVP	0.067	(-0.006 to 0.14)
**Fixed effects meta**	**0.095**	**(0.063 to 0.127)**
**Meta p-value**		**7.125E-9**
**I2 Heterogeneity**		**0**
**Q statistic p-value**		**0.8365**
**Number of samples**		**1027900**

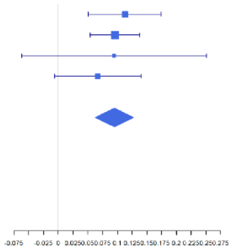

5:42515027
rs62370646

Cohort name	beta	95% CI
ICBP	-0.148	(-0.218 to -0.078)
UKBio	-0.107	(-0.158 to -0.056)
BioVu	-0.047	(-0.231 to 0.137)
MVP	-0.117	(-0.198 to -0.035)
**Fixed effects meta**	**-0.119**	**(-0.157 to -0.081)**
**Meta p-value**		**7.973E-10**
**I2 Heterogeneity**		**0**
**Q statistic p-value**		**0.7011**
**Number of samples**		**1027900**

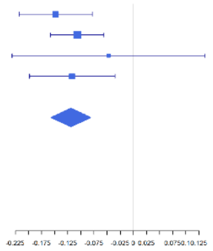

5:58352210
rs10061553

Cohort name	beta	95% CI
ICBP	-0.076	(-0.133 to -0.019)
UKBio	-0.092	(-0.135 to -0.049)
BioVu	-0.105	(-0.261 to 0.051)
MVP	-0.099	(-0.169 to -0.029)
**Fixed effects meta**	**-0.089**	**(-0.121 to -0.057)**
**Meta p-value**		**4.596E-8**
**I2 Heterogeneity**		**0**
**Q statistic p-value**		**0.9528**
**Number of samples**		**1027890**

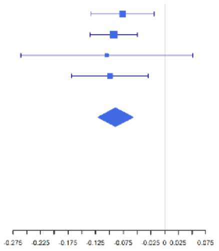

5:76884661
rs34237622

Cohort name	beta	95% CI
ICBP	-0.113	(-0.187 to -0.04)
UKBio	-0.133	(-0.187 to -0.08)
BioVu	-0.203	(-0.403 to -0.002)
MVP	-0.044	(-0.137 to 0.049)
**Fixed effects meta**	**-0.114**	**(-0.154 to -0.073)**
**Meta p-value**		**3.719E-8**
**I2 Heterogeneity**		**8.8**
**Q statistic p-value**		**0.3493**
**Number of samples**		**1027900**

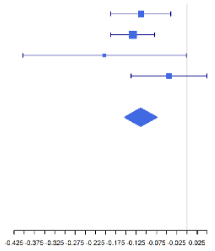

1:21155195
rs2320590

Cohort name	beta	95% CI
ICBP	0.069	(0.016 to 0.122)
UKBio	0.091	(0.051 to 0.131)
BioVu	0.009	(-0.137 to 0.155)
MVP	0.111	(0.048 to 0.174)
**Fixed effects meta**	**0.085**	**(0.056 to 0.114)**

**Meta p-value**	**1.384E-8**
**I2 Heterogeneity**	**0**
**Q statistic p-value**	**0.5587**
**Number of samples**	**1027880**

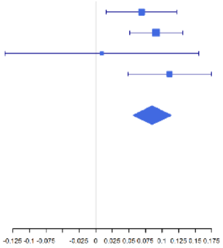

1:68143195
rs10889711

Cohort name	beta	95% CI
ICBP	-0.075	(-0.131 to -0.02)
UKBio	-0.113	(-0.155 to -0.072)
BioVu	-0.064	(-0.212 to 0.084)
MVP	-0.069	(-0.136 to -0.003)
**Fixed effects meta**	**-0.09**	**(-0.121 to -0.06)**

**Meta p-value**	**6.566E-9**
**I2 Heterogeneity**	**0**
**Q statistic p-value**	**0.6282**
**Number of samples**	**1019060**

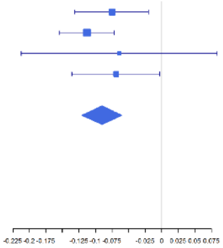

1:110229787
rs36209093

Cohort name	beta	95% CI
UKBio	0.158	(0.111 to 0.205)
BioVu	0.155	(-0.032 to 0.343)
MVP	0.205	(0.122 to 0.287)
**Fixed effects meta**	**0.17**	**(0.127 to 0.213)**

**Meta p-value**	**9.938E-15**
**I2 Heterogeneity**	**0**
**Q statistic p-value**	**0.6396**
**Number of samples**	**729874**

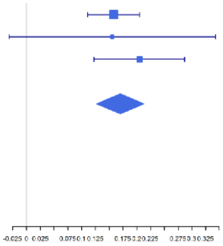

1:112261533
rs565522

Cohort name	beta	95% CI
ICBP	-0.082	(-0.136 to -0.028)
UKBio	-0.085	(-0.125 to -0.045)
BioVu	-0.149	(-0.294 to -0.005)
MVP	-0.081	(-0.147 to -0.014)
**Fixed effects meta**	**-0.086**	**(-0.116 to -0.056)**

**Meta p-value**	**1.739E-8**
**I2 Heterogeneity**	**0**
**Q statistic p-value**	**0.8574**
**Number of samples**	**1020640**

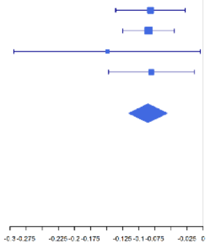

1:118223275
rs6669446

Cohort name	beta	95% CI
ICBP	-0.073	(-0.127 to -0.019)
UKBio	-0.091	(-0.131 to -0.051)
BioVu	-0.088	(-0.233 to 0.058)
MVP	-0.108	(-0.172 to -0.044)
**Fixed effects meta**	**-0.089**	**(-0.118 to -0.059)**

**Meta p-value**	**4.288E-9**
**I2 Heterogeneity**	**0**
**Q statistic p-value**	**0.8761**
**Number of samples**	**1028900**

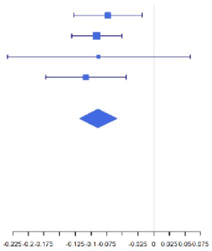

2:9803203
rs57503539

Cohort name	beta	95% CI
ICBP	-0.125	(-0.193 to -0.057)
UKBio	-0.115	(-0.165 to -0.065)
BioVu	-0.14	(-0.317 to 0.038)
MVP	-0.112	(-0.191 to -0.032)
**Fixed effects meta**	**-0.118**	**(-0.155 to -0.081)**

**Meta p-value**	**3.421E-10**
**I2 Heterogeneity**	**0**
**Q statistic p-value**	**0.9879**
**Number of samples**	**1026780**

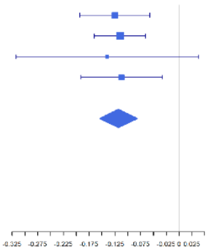

3:25424929
rs2306623

Cohort name	beta	95% CI
ICBP	0.126	(0.07 to 0.183)
UKBio	0.055	(0.013 to 0.097)
BioVu	0.139	(-0.015 to 0.292)
MVP	0.116	(0.049 to 0.183)
**Fixed effects meta**	**0.093**	**(0.062 to 0.124)**

**Meta p-value**	**5.22E-9**
**I2 Heterogeneity**	**35.1**
**Q statistic p-value**	**0.2017**
**Number of samples**	**1027780**

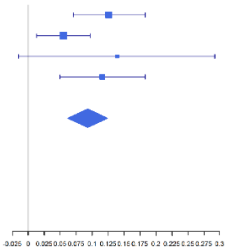

3:117492152
rs6805393

Cohort name	beta	95% CI
ICBP	-0.041	(-0.094 to 0.011)
UKBio	-0.108	(-0.148 to -0.068)
BioVu	-0.137	(-0.279 to 0.005)
MVP	-0.079	(-0.143 to -0.016)
**Fixed effects meta**	**-0.083**	**(-0.112 to -0.053)**

**Meta p-value**	**3.274E-8**
**I2 Heterogeneity**	**27.9**
**Q statistic p-value**	**0.2444**
**Number of samples**	**1028900**

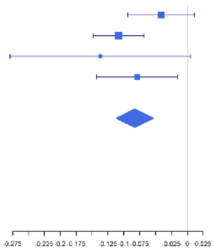

14:84911548
rs12883344

Cohort name	beta	95% CI
ICBP	0.067	(0.013 to 0.121)
UKBio	0.087	(0.046 to 0.128)
BioVu	0.007	(-0.138 to 0.151)
MVP	0.115	(0.051 to 0.178)
**Fixed effects meta**	**0.083**	**(0.053 to 0.113)**
**Meta p-value**		**4.944E-8**
**I2 Heterogeneity**		**0**
**Q statistic p-value**		**0.5073**
**Number of samples**		**1028900**

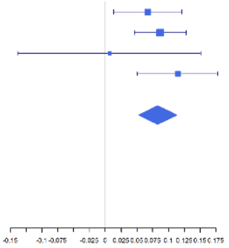

15:51559845
rs28490942

Cohort name	beta	95% CI
ICBP	-0.06	(-0.113 to -0.007)
UKBio	-0.109	(-0.15 to -0.069)
BioVu	-0.052	(-0.195 to 0.091)
MVP	-0.097	(-0.161 to -0.033)
**Fixed effects meta**	**-0.089**	**(-0.119 to -0.06)**
**Meta p-value**		**3.248E-9**
**I2 Heterogeneity**		**0**
**Q statistic p-value**		**0.5244**
**Number of samples**		**1028900**

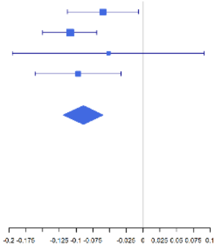

15:72429989
rs2034879

Cohort name	beta	95% CI
ICBP	0.093	(0.032 to 0.154)
UKBio	0.104	(0.058 to 0.151)
BioVu	0.077	(-0.097 to 0.251)
MVP	0.089	(0.007 to 0.171)
**Fixed effects meta**	**0.097**	**(0.062 to 0.132)**
**Meta p-value**		**4.392E-8**
**I2 Heterogeneity**		**0**
**Q statistic p-value**		**0.9798**
**Number of samples**		**1028900**

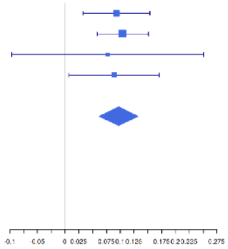

15:82186535
rs983353

Cohort name	beta	95% CI
ICBP	0.092	(0.034 to 0.15)
UKBio	0.121	(0.077 to 0.165)
BioVu	0.018	(-0.139 to 0.175)
MVP	0.037	(-0.034 to 0.108)
**Fixed effects meta**	**0.091**	**(0.058 to 0.123)**
**Meta p-value**		**3.653E-8**
**I2 Heterogeneity**		**32.6**
**Q statistic p-value**		**0.2167**
**Number of samples**		**1026790**

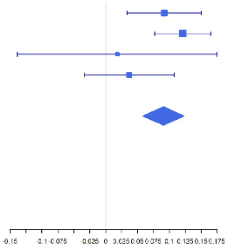

15:94214587
rs7174977

Cohort name	beta	95% CI
ICBP	0.088	(0.031 to 0.145)
UKBio	0.11	(0.068 to 0.152)
BioVu	0.024	(-0.127 to 0.174)
MVP	0.068	(0.001 to 0.135)
**Fixed effects meta**	**0.091**	**(0.06 to 0.122)**
**Meta p-value**		**8.152E-9**
**I2 Heterogeneity**		**0**
**Q statistic p-value**		**0.5978**
**Number of samples**		**1022910**

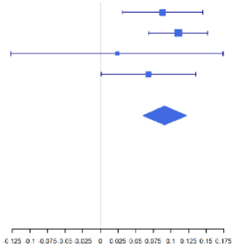

16:56859216
rs12919839

Cohort name	beta	95% CI
ICBP	-0.133	(-0.191 to -0.074)
UKBio	-0.093	(-0.138 to -0.049)
BioVu	-0.026	(-0.183 to 0.131)
MVP	-0.077	(-0.148 to -0.007)
**Fixed effects meta**	**-0.099**	**(-0.132 to -0.067)**
**Meta p-value**		**2.149E-9**
**I2 Heterogeneity**		**0**
**Q statistic p-value**		**0.4709**
**Number of samples**		**1028900**

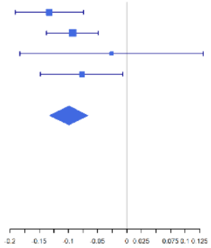

16:84082650
rs8056413

Cohort name	beta	95% CI
ICBP	-0.072	(-0.128 to -0.017)
UKBio	-0.11	(-0.152 to -0.069)
BioVu	-0.056	(-0.201 to 0.089)
MVP	-0.095	(-0.162 to -0.029)
**Fixed effects meta**	**-0.093**	**(-0.124 to -0.063)**
**Meta p-value**		**1.746E-9**
**I2 Heterogeneity**		**0**
**Q statistic p-value**		**0.7176**
**Number of samples**		**1027890**

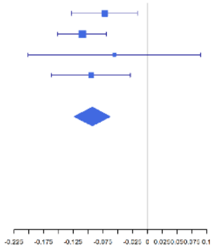

18:77161324
rs117777118

Cohort name	beta	95% CI
UKBio	-0.39	(-0.503 to -0.278)
BioVu	-0.558	(-1.072 to -0.045)
MVP	-0.287	(-0.443 to -0.13)
**Fixed effects meta**	**-0.358**	**(-0.454 to -0.262)**
**Meta p-value**		**2.398E-13**
**I2 Heterogeneity**		**0**
**Q statistic p-value**		**0.4463**
**Number of samples**		**729881**

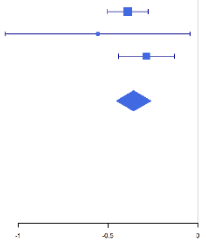

19:44746657
rs2125578

Cohort name	beta	95% CI
ICBP	-0.102	(-0.155 to -0.049)
UKBio	-0.083	(-0.123 to -0.043)
BioVu	-0.133	(-0.276 to 0.01)
MVP	-0.048	(-0.111 to 0.014)
**Fixed effects meta**	**-0.083**	**(-0.113 to -0.054)**
**Meta p-value**		**2.7E-8**
**I2 Heterogeneity**		**0**
**Q statistic p-value**		**0.5558**
**Number of samples**		**1025680**

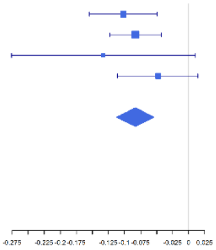

20:35169916
rs146827176

Cohort name	beta	95% CI
ICBP	-0.142	(-0.278 to -0.006)
UKBio	-0.226	(-0.319 to -0.132)
BioVu	-0.172	(-0.532 to 0.187)
MVP	-0.252	(-0.417 to -0.086)
**Fixed effects meta**	**-0.205**	**(-0.277 to -0.133)**
**Meta p-value**		**2.754E-8**
**I2 Heterogeneity**		**0**
**Q statistic p-value**		**0.7265**
**Number of samples**		**1020700**

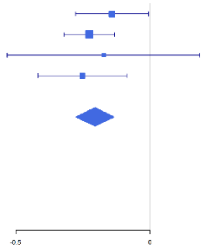

c

9:80751434
rs112324977

Cohort name	beta	95% CI
ICBP	-0.167	(-0.257 to -0.077)
UKBio	-0.16	(-0.234 to -0.086)
BioVu	-0.065	(-0.293 to 0.164)
MVP	-0.115	(-0.214 to -0.016)
**Fixed effects meta**	**-0.147**	**(-0.197 to -0.096)**
**Meta p-value**		**1.067E-8**
**I2 Heterogeneity**		**0**
**Q statistic p-value**		**0.763**
**Number of samples**		**1017040**

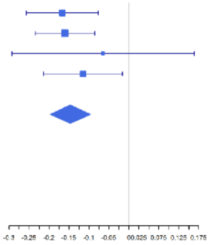

9:122890934
rs72751391

Cohort name	beta	95% CI
ICBP	0.172	(0.073 to 0.271)
UKBio	0.139	(0.065 to 0.214)
BioVu	0.129	(-0.111 to 0.368)
MVP	0.172	(0.06 to 0.284)
**Fixed effects meta**	**0.156**	**(0.102 to 0.209)**
**Meta p-value**		**1.226E-8**
**I2 Heterogeneity**		**0**
**Q statistic p-value**		**0.9418**
**Number of samples**		**1016040**

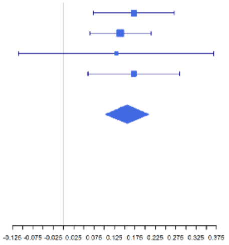

9:133711263
rs2987903

Cohort name	beta	95% CI
ICBP	-0.174	(-0.265 to -0.083)
UKBio	-0.151	(-0.225 to -0.076)
BioVu	-0.264	(-0.488 to -0.039)
MVP	-0.113	(-0.211 to -0.015)
**Fixed effects meta**	**-0.154**	**(-0.205 to -0.103)**
**Meta p-value**		**2.486E-9**
**I2 Heterogeneity**		**0**
**Q statistic p-value**		**0.6305**
**Number of samples**		**1017040**

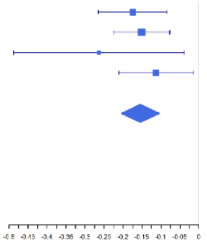

11:11793978
rs11022023

Cohort name	beta	95% CI
ICBP	-0.128	(-0.24 to -0.017)
UKBio	-0.215	(-0.306 to -0.125)
BioVu	-0.047	(-0.323 to 0.229)
MVP	-0.189	(-0.314 to -0.063)
**Fixed effects meta**	**-0.174**	**(-0.236 to -0.111)**
**Meta p-value**		**4.798E-8**
**I2 Heterogeneity**		**0**
**Q statistic p-value**		**0.5385**
**Number of samples**		**1008280**

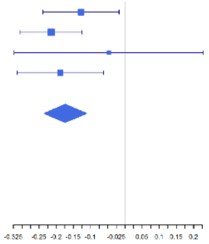

11:73783478
rs4944038

Cohort name	beta	95% CI
ICBP	-0.056	(-0.117 to 0.005)
UKBio	-0.118	(-0.167 to -0.069)
BioVu	-0.169	(-0.32 to -0.019)
MVP	-0.114	(-0.18 to -0.049)
**Fixed effects meta**	**-0.101**	**(-0.134 to -0.067)**
**Meta p-value**		**4.298E-9**
**I2 Heterogeneity**		**12.5**
**Q statistic p-value**		**0.3301**
**Number of samples**		**1009390**

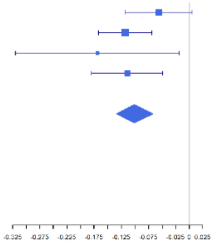

11:110657616
rs77759442

Cohort name	beta	95% CI
ICBP	0.165	(0.076 to 0.254)
UKBio	0.132	(0.057 to 0.206)
BioVu	0.337	(0.114 to 0.56)
MVP	0.122	(0.019 to 0.224)
**Fixed effects meta**	**0.15**	**(0.1 to 0.201)**
**Meta p-value**		**5.905E-9**
**I2 Heterogeneity**		**7.1**
**Q statistic p-value**		**0.3576**
**Number of samples**		**1009390**

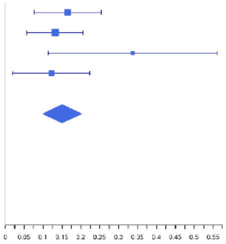

12:12045264
rs1062298

Cohort name	beta	95% CI
ICBP	0.047	(-0.016 to 0.109)
UKBio	0.088	(0.038 to 0.138)
BioVu	0.15	(-0.005 to 0.305)
MVP	0.165	(0.096 to 0.233)
**Fixed effects meta**	**0.097**	**(0.063 to 0.132)**
**Meta p-value**		**3.241E-8**
**I2 Heterogeneity**		**54.8**
**Q statistic p-value**		**0.08414**
**Number of samples**		**1015920**

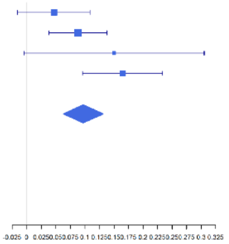

12:46385848
rs12828693

Cohort name	beta	95% CI
ICBP	0.129	(0.051 to 0.206)
UKBio	0.111	(0.05 to 0.171)
BioVu	0.211	(0.022 to 0.4)
MVP	0.126	(0.04 to 0.212)
**Fixed effects meta**	**0.125**	**(0.082 to 0.167)**
**Meta p-value**		**8.199E-9**
**I2 Heterogeneity**		**0**
**Q statistic p-value**		**0.8063**
**Number of samples**		**1016040**

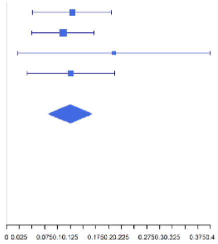

6:99548729
rs72943226

Cohort name	beta	95% CI
ICBP	0.118	(0.053 to 0.183)
UKBio	0.122	(0.069 to 0.175)
BioVu	0.16	(-0.002 to 0.323)
MVP	0.038	(-0.033 to 0.109)
**Fixed effects meta**	**0.102**	**(0.066 to 0.138)**
**Meta p-value**		**3.118E-8**
**I2 Heterogeneity**		**27.8**
**Q statistic p-value**		**0.245**
**Number of samples**		**1009390**

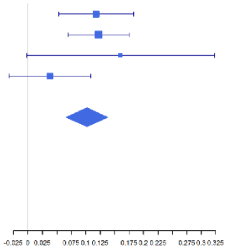

6:121258543
rs9320778

Cohort name	beta	95% CI
ICBP	0.143	(0.073 to 0.214)
UKBio	0.091	(0.034 to 0.148)
BioVu	0.106	(-0.068 to 0.279)
MVP	0.12	(0.042 to 0.199)
**Fixed effects meta**	**0.115**	**(0.076 to 0.154)**
**Meta p-value**		**1.046E-8**
**I2 Heterogeneity**		**0**
**Q statistic p-value**		**0.7503**
**Number of samples**		**1009380**

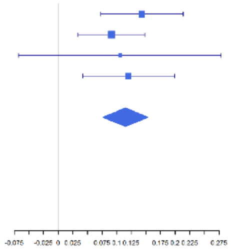

7:15421023
rs67615620

Cohort name	beta	95% CI
ICBP	0.135	(0.057 to 0.213)
UKBio	0.087	(0.026 to 0.148)
BioVu	0.227	(0.038 to 0.415)
MVP	0.144	(0.058 to 0.23)
**Fixed effects meta**	**0.122**	**(0.079 to 0.165)**
**Meta p-value**		**2.321E-8**
**I2 Heterogeneity**		**0**
**Q statistic p-value**		**0.4625**
**Number of samples**		**1009390**

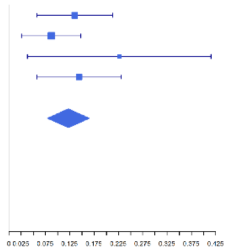

7:16117030
rs75177877

Cohort name	beta	95% CI
ICBP	0.075	(-0.006 to 0.155)
UKBio	0.191	(0.126 to 0.257)
BioVu	0.189	(-0.017 to 0.395)
MVP	0.11	(0.011 to 0.208)
**Fixed effects meta**	**0.136**	**(0.09 to 0.182)**
**Meta p-value**		**7.015E-9**
**I2 Heterogeneity**		**40.5**
**Q statistic p-value**		**0.1687**
**Number of samples**		**1009390**

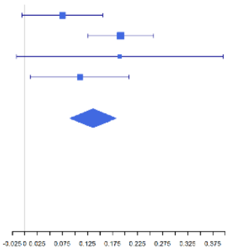

8:34164285
rs2953937

Cohort name	beta	95% CI
ICBP	0.118	(0.028 to 0.207)
UKBio	0.204	(0.132 to 0.277)
BioVu	0.215	(-0.008 to 0.438)
MVP	0.061	(-0.039 to 0.16)
**Fixed effects meta**	**0.143**	**(0.093 to 0.193)**
**Meta p-value**		**1.755E-8**
**I2 Heterogeneity**		**46**
**Q statistic p-value**		**0.1353**
**Number of samples**		**1009390**

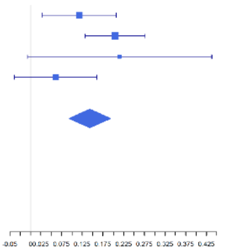

8:108319395
rs36036692

Cohort name	beta	95% CI
ICBP	0.044	(-0.018 to 0.107)
UKBio	0.119	(0.068 to 0.17)
BioVu	0.189	(0.034 to 0.345)
MVP	0.12	(0.052 to 0.189)
**Fixed effects meta**	**0.1**	**(0.065 to 0.135)**
**Meta p-value**		**1.888E-8**
**I2 Heterogeneity**		**40.9**
**Q statistic p-value**		**0.1666**
**Number of samples**		**1009390**

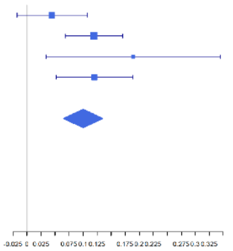

9:14535119
rs34361301

Cohort name	beta	95% CI
ICBP	0.08	(0.011 to 0.148)
UKBio	0.162	(0.106 to 0.219)
BioVu	0.155	(-0.016 to 0.325)
MVP	0.102	(0.024 to 0.18)
**Fixed effects meta**	**0.122**	**(0.083 to 0.16)**
**Meta p-value**		**6.762E-10**
**I2 Heterogeneity**		**14.1**
**Q statistic p-value**		**0.3215**
**Number of samples**		**1017040**

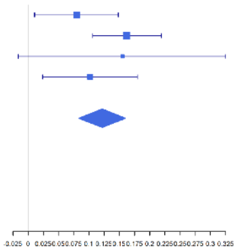

9:35191014
rs61241090

Cohort name	beta	95% CI
ICBP	-0.161	(-0.232 to -0.089)
UKBio	-0.109	(-0.167 to -0.051)
BioVu	-0.085	(-0.263 to 0.094)
MVP	-0.105	(-0.186 to -0.025)
**Fixed effects meta**	**-0.123**	**(-0.163 to -0.083)**
**Meta p-value**		**1.395E-9**
**I2 Heterogeneity**		**0**
**Q statistic p-value**		**0.6579**
**Number of samples**		**1015830**

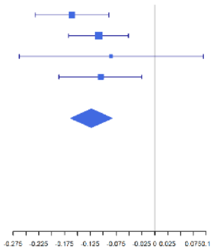

1:40763095
rs12134085

Cohort name	beta	95% CI
ICBP	-0.017	(-0.099 to 0.065)
UKBio	-0.17	(-0.233 to -0.106)
BioVu	-0.351	(-0.558 to -0.144)
MVP	-0.181	(-0.28 to -0.082)
**Fixed effects meta**	**-0.133**	**(-0.179 to -0.087)**
**Meta p-value**		**1.054E-8**
**I2 Heterogeneity**		**78.4**
**Q statistic p-value**		**0.003098**
**Number of samples**		**1008380**

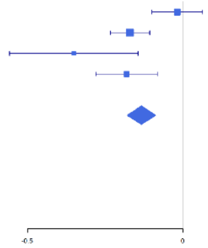

1:72229240
rs12084868

Cohort name	beta	95% CI
ICBP	0.213	(0.015 to 0.41)
UKBio	0.321	(0.174 to 0.469)
BioVu	0.171	(-0.313 to 0.655)
MVP	0.429	(0.187 to 0.671)
**Fixed effects meta**	**0.302**	**(0.194 to 0.41)**
**Meta p-value**		**4.665E-8**
**I2 Heterogeneity**		**0**
**Q statistic p-value**		**0.5455**
**Number of samples**		**1009390**

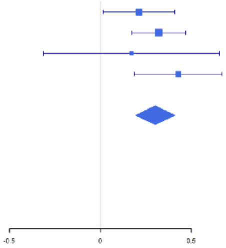

1:115019239
rs71664847

Cohort name	beta	95% CI
ICBP	0.09	(0.012 to 0.169)
UKBio	0.153	(0.09 to 0.215)
BioVu	0.073	(-0.119 to 0.266)
MVP	0.135	(0.051 to 0.219)
**Fixed effects meta**	**0.126**	**(0.083 to 0.169)**
**Meta p-value**		**9.987E-9**
**I2 Heterogeneity**		**0**
**Q statistic p-value**		**0.633**
**Number of samples**		**1009390**

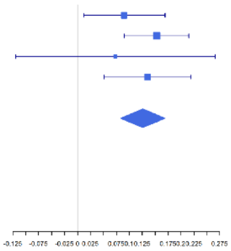

2:209622
rs300753

Cohort name	beta	95% CI
ICBP	0.065	(0.003 to 0.127)
UKBio	0.124	(0.075 to 0.173)
BioVu	0.116	(-0.035 to 0.267)
MVP	0.126	(0.058 to 0.194)
**Fixed effects meta**	**0.106**	**(0.072 to 0.14)**
**Meta p-value**		**1.055E-9**
**I2 Heterogeneity**		**0**
**Q statistic p-value**		**0.4874**
**Number of samples**		**1007270**

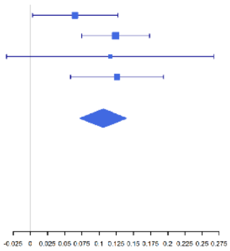

2:196590414
rs10208493

Cohort name	beta	95% CI
ICBP	-0.046	(-0.108 to 0.015)
UKBio	-0.136	(-0.186 to -0.086)
BioVu	-0.131	(-0.284 to 0.022)
MVP	-0.099	(-0.167 to -0.031)
**Fixed effects meta**	**-0.099**	**(-0.133 to -0.065)**
**Meta p-value**		**1.364E-8**
**I2 Heterogeneity**		**37.7**
**Q statistic p-value**		**0.1855**
**Number of samples**		**1009390**

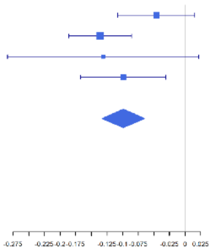

3:69919744
rs62253186

Cohort name	beta	95% CI
ICBP	0.247	(0.112 to 0.381)
UKBio	0.204	(0.101 to 0.308)
BioVu	0.258	(-0.067 to 0.584)
MVP	0.192	(0.042 to 0.342)
**Fixed effects meta**	**0.217**	**(0.143 to 0.29)**
**Meta p-value**		**7.138E-9**
**I2 Heterogeneity**		**0**
**Q statistic p-value**		**0.9397**
**Number of samples**		**1009390**

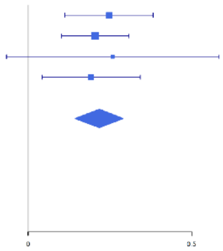

3:187456904
rs3821817

Cohort name	beta	95% CI
ICBP	-0.103	(-0.185 to -0.021)
UKBio	-0.188	(-0.253 to -0.123)
BioVu	-0.137	(-0.348 to 0.075)
MVP	-0.085	(-0.176 to 0.006)
**Fixed effects meta**	**-0.135**	**(-0.181 to -0.09)**
**Meta p-value**		**4.592E-9**
**I2 Heterogeneity**		**22.8**
**Q statistic p-value**		**0.2741**
**Number of samples**		**1007280**

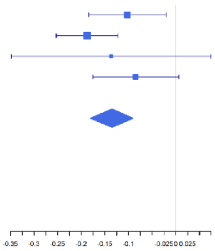

6:85988429
rs4053778

Cohort name	beta	95% CI
ICBP	-0.107	(-0.169 to -0.045)
UKBio	-0.109	(-0.16 to -0.058)
BioVu	-0.222	(-0.38 to -0.065)
MVP	-0.063	(-0.133 to 0.008)
**Fixed effects meta**	**-0.103**	**(-0.138 to -0.068)**
**Meta p-value**		**8.672E-9**
**I2 Heterogeneity**		**12.1**
**Q statistic p-value**		**0.3321**
**Number of samples**		**1009390**

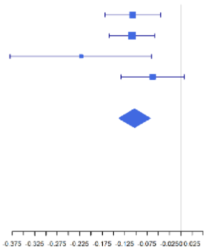

13:94417873
rs4517643

Cohort name	beta	95% CI
ICBP	0.106	(0.043 to 0.168)
UKBio	0.121	(0.071 to 0.171)
BioVu	0.035	(-0.118 to 0.188)
MVP	0.077	(0.01 to 0.143)
**Fixed effects meta**	**0.101**	**(0.067 to 0.135)**
**Meta p-value**		**7.472E-9**
**I2 Heterogeneity**		**0**
**Q statistic p-value**		**0.6328**
**Number of samples**		**1016040**

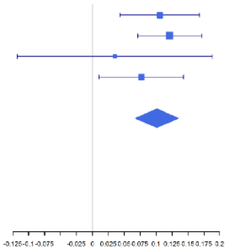

13:98859019
rs9554446

Cohort name	beta	95% CI
ICBP	0.133	(0.026 to 0.241)
UKBio	0.145	(0.059 to 0.231)
BioVu	0.156	(-0.102 to 0.415)
MVP	0.233	(0.123 to 0.343)
**Fixed effects meta**	**0.166**	**(0.108 to 0.224)**
**Meta p-value**		**2.322E-8**
**I2 Heterogeneity**		**0**
**Q statistic p-value**		**0.5863**
**Number of samples**		**1008320**

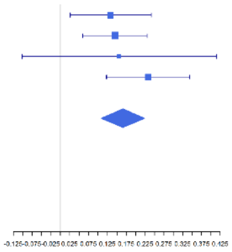

14:103330144
rs9671694

Cohort name	beta	95% CI
ICBP	0.076	(0.011 to 0.141)
UKBio	0.109	(0.057 to 0.161)
BioVu	0.135	(-0.028 to 0.298)
MVP	0.127	(0.052 to 0.203)
**Fixed effects meta**	**0.104**	**(0.067 to 0.14)**
**Meta p-value**		**2.404E-8**
**I2 Heterogeneity**		**0**
**Q statistic p-value**		**0.7431**
**Number of samples**		**1016030**

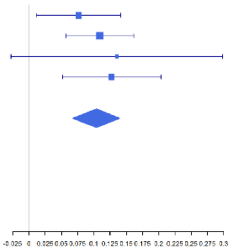

15:101791212
rs116643984

Cohort name	beta	95% CI
ICBP	-0.14	(-0.223 to -0.057)
UKBio	-0.164	(-0.232 to -0.096)
BioVu	-0.165	(-0.373 to 0.044)
MVP	-0.104	(-0.199 to -0.009)
**Fixed effects meta**	**-0.142**	**(-0.189 to -0.096)**
**Meta p-value**		**2.453E-9**
**I2 Heterogeneity**		**0**
**Q statistic p-value**		**0.8058**
**Number of samples**		**1013920**

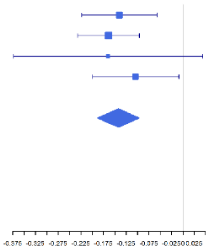

16:88534923
rs34139656

Cohort name	beta	95% CI
ICBP	-0.134	(-0.205 to -0.063)
UKBio	-0.121	(-0.174 to -0.069)
BioVu	-0.136	(-0.306 to 0.034)
MVP	-0.088	(-0.159 to -0.017)
**Fixed effects meta**	**-0.117**	**(-0.154 to -0.08)**
**Meta p-value**		**7.303E-10**
**I2 Heterogeneity**		**0**
**Q statistic p-value**		**0.8191**
**Number of samples**		**995827**

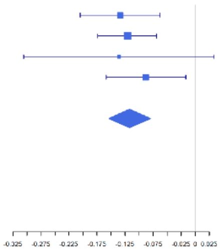

17:78238645
rs12943001

Cohort name	beta	95% CI
ICBP	-0.024	(-0.102 to 0.055)
UKBio	-0.15	(-0.202 to -0.098)
BioVu	-0.19	(-0.369 to -0.011)
MVP	-0.109	(-0.186 to -0.032)
**Fixed effects meta**	**-0.111**	**(-0.15 to -0.072)**
**Meta p-value**		**2.294E-8**
**I2 Heterogeneity**		**58.7**
**Q statistic p-value**		**0.06394**
**Number of samples**		**963320**

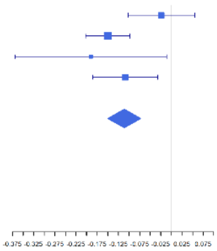

22:37462936
rs855791

Cohort name	beta	95% CI
ICBP	-0.08	(-0.142 to -0.019)
UKBio	-0.094	(-0.144 to -0.044)
BioVu	-0.084	(-0.238 to 0.071)
MVP	-0.12	(-0.185 to -0.054)
**Fixed effects meta**	**-0.096**	**(-0.13 to -0.062)**
**Meta p-value**		**3.24E-8**
**I2 Heterogeneity**		**0**
**Q statistic p-value**		**0.8613**
**Number of samples**		**1017050**

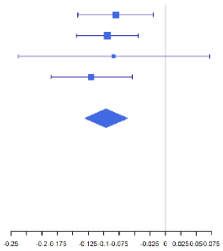

Supplementary Figure 2a-c. Forest plots for all novel loci for systolic (a), diastolic (b), and pulse pressure (c). ICBP = International Consortium of Blood Pressure meta-analysis (n=299,024); UKBio = UK Biobank (n=458,577); BioVU = Biobank Repository of the Vanderbilt University (n=50,649); MVP = Million Veterans Program (n=220,501); CI =

Confidence Interval. Meta-analysis p-values are calculated by inverse variance-weighted method, heterogeneity is calculated by I^2 statistic and Cochran's Q Test.

a

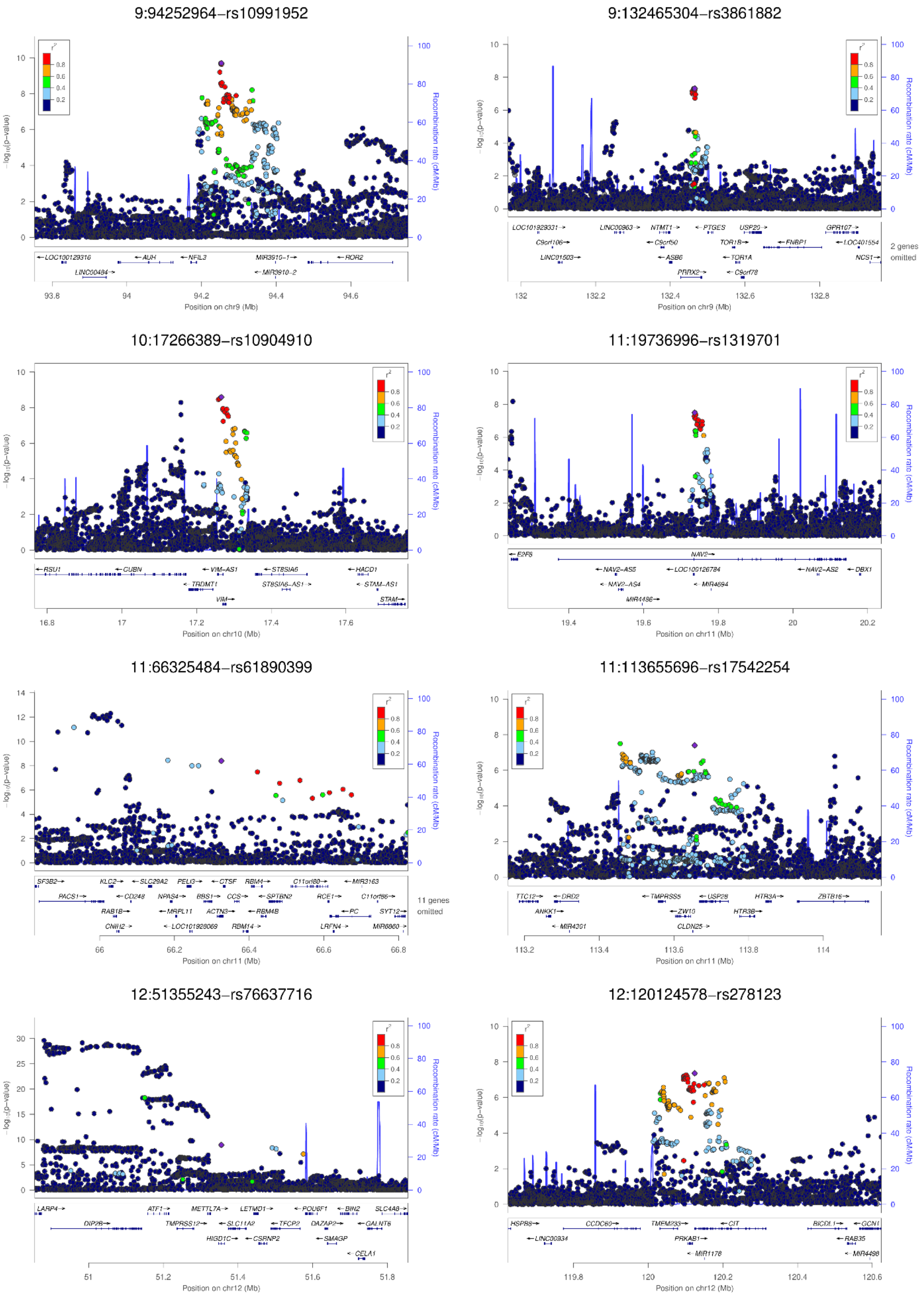

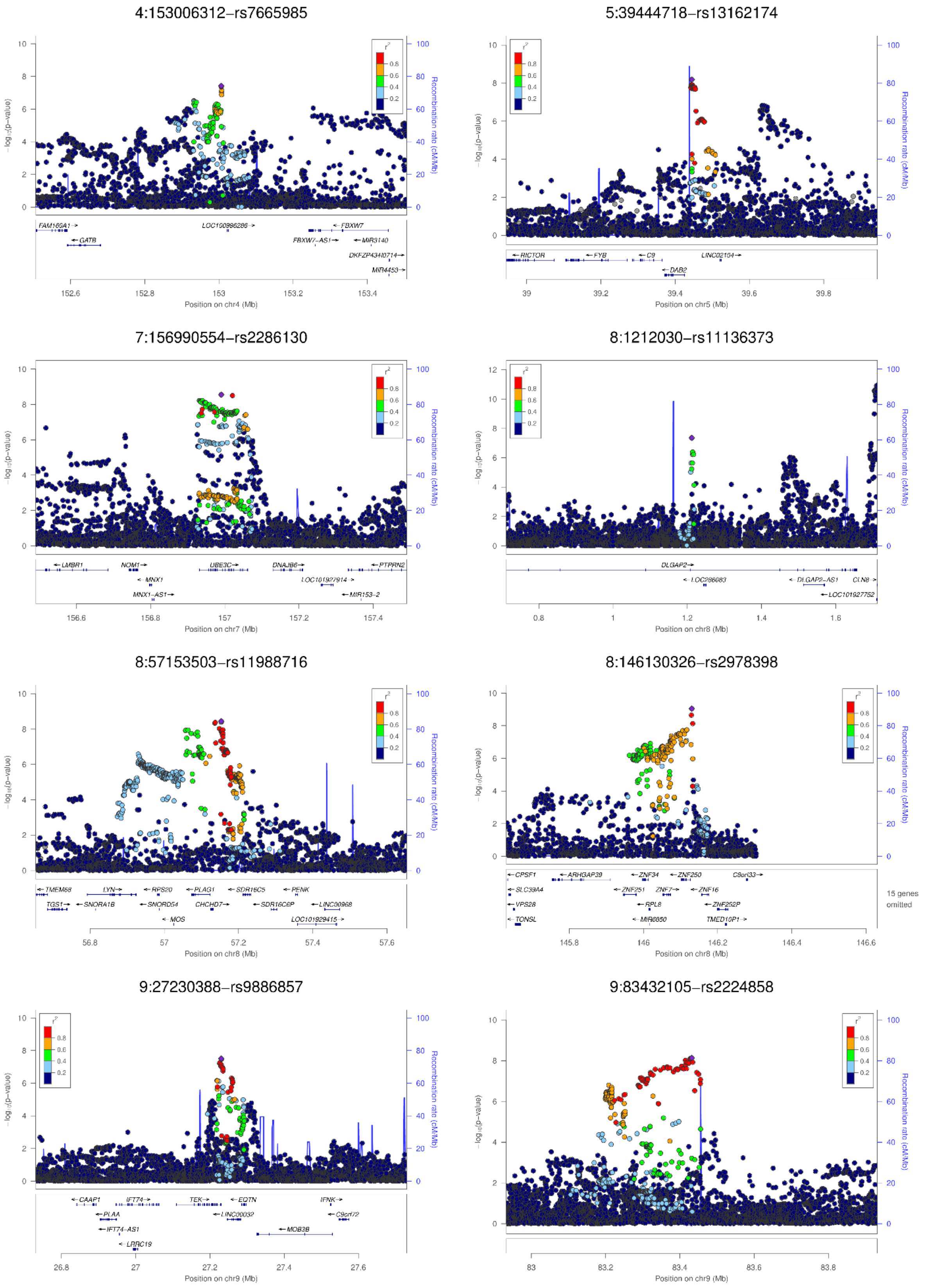
4:153006312–rs7665985
5:39444718–rs13162174
7:156990554–rs2286130
8:1212030–rs11136373
8:57153503–rs11988716
8:146130326–rs2978398
9:27230388–rs9886857
9:83432105–rs2224858
−log10(p−value)
Recombination rate (cM/Mb)
Position on chr4 (Mb)
Position on chr5 (Mb)
Position on chr7 (Mb)
Position on chr8 (Mb)
Position on chr9 (Mb)
15 genes omitted
FAM160A1
GATB
LOC100996286
FBXW7
FBXW7−AS1
MIR3140
DKFZP434I0714
MIR4453
RICTOR
FYB
C9
DAB2
LINC02104
LMBR1
NOM1
MNX1
MNX1−AS1
UBE3C
DNAJB6
LOC101927914
PTPRN2
MIR153−2
DLGAP2
LOC286083
DLGAP2−AS1
CLN8
LOC101927752
TMEM68
TGS1
LYN
SNORA1B
RPS20
SNORD54
PLAG1
CHCHD7
MOS
SDR16C5
SDR16C6P
PENK
LINC00968
LOC101929415
CPSF1
SLC39A4
VPS28
TONSL
ARHGAP39
ZNF34
ZNF251
ZNF7
RPL8
MIR6850
ZNF250
ZNF16
ZNF252P
TMED10P1
C8orf33
CAAP1
PLAA
IFT74−AS1
LRRC19
IFT74
TEK
LINC00032
EQTN
MOB3B
IFNK
C9orf72

2:105205551−rs6729623

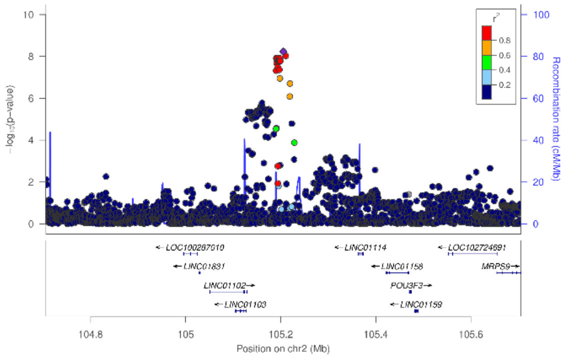

2:125429006−rs11123059

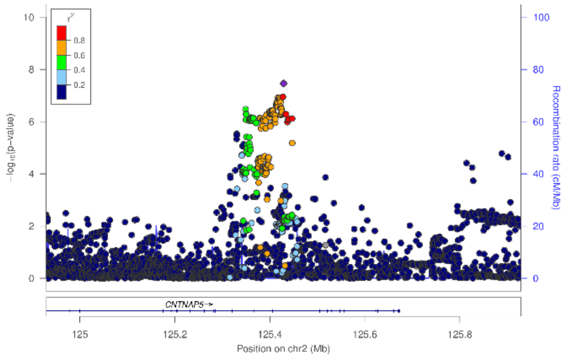

2:127839534−rs11690153

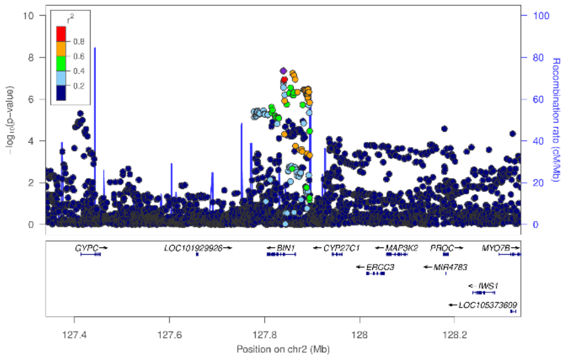

2:128822702−rs13022015

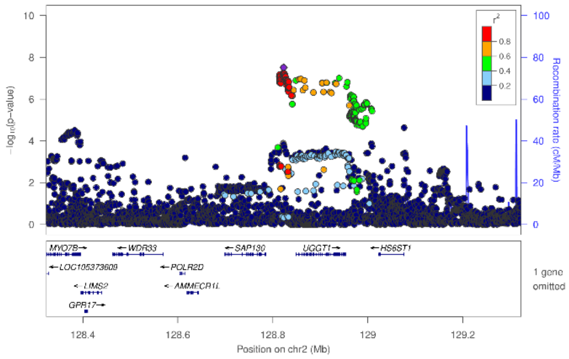

3:16363689−rs538180

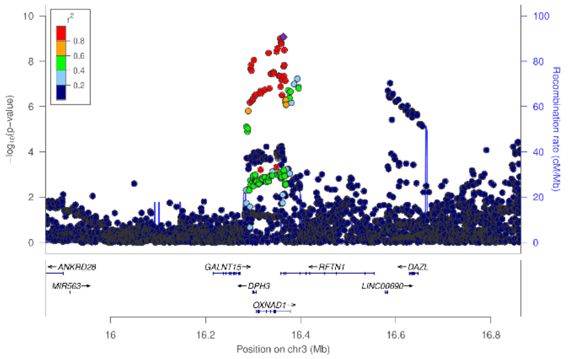

3:43992455−rs9877020

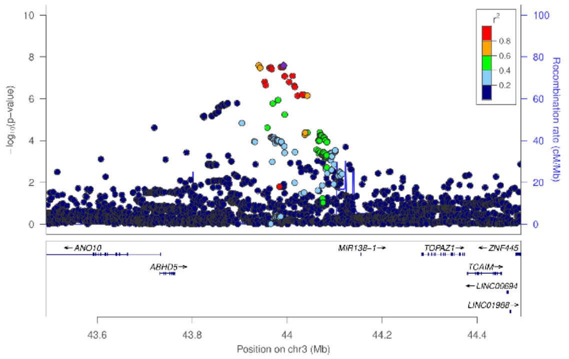

3:71607861−rs844218

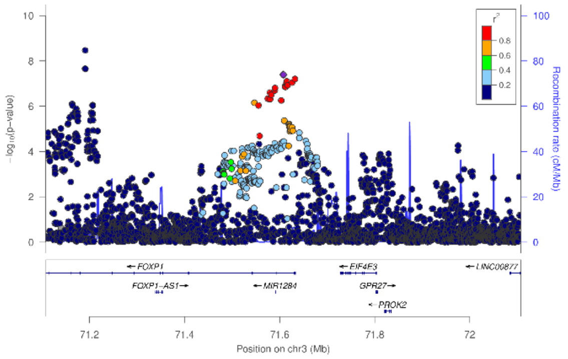

4:84452950−rs10018970

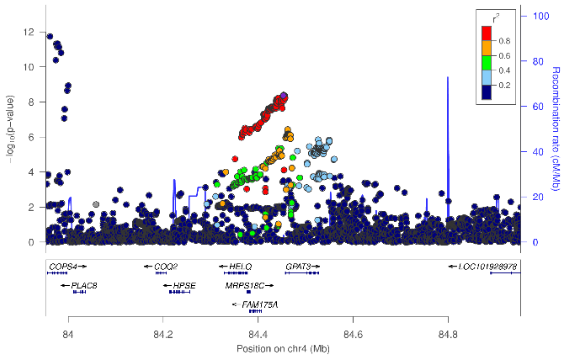

1:61877445–rs2092867

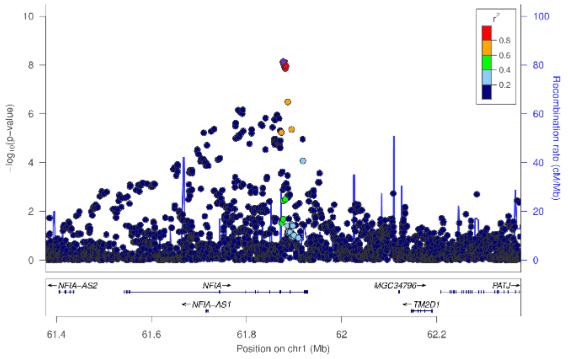

1:93524045–rs12145044

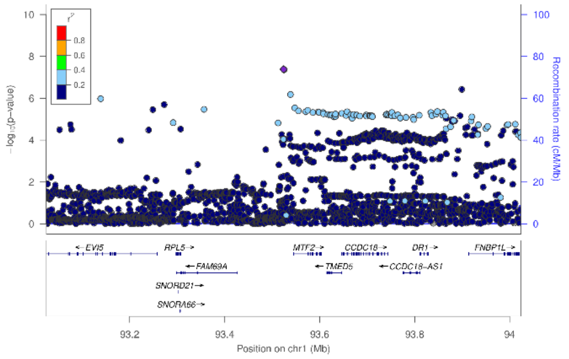

1:166023209–rs4573493

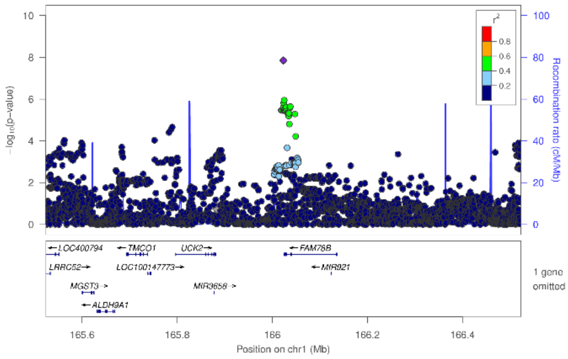

1:193271526–rs817140

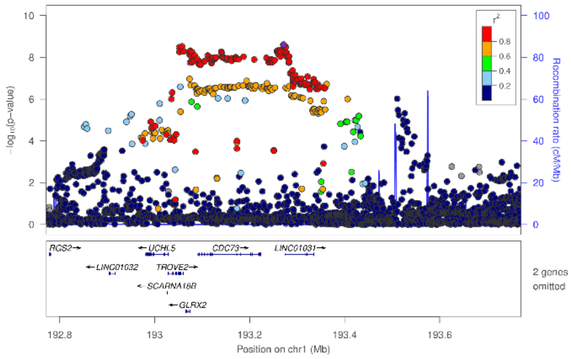

2:12994692–rs6723772

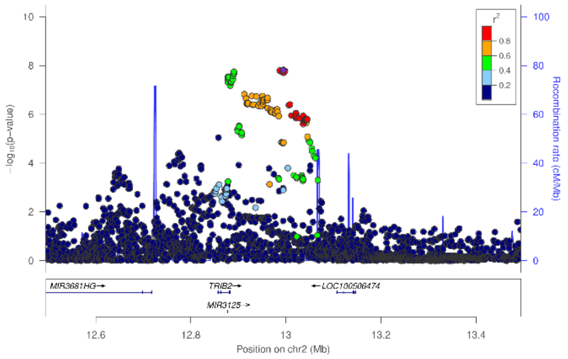

2:32620888–rs10172510

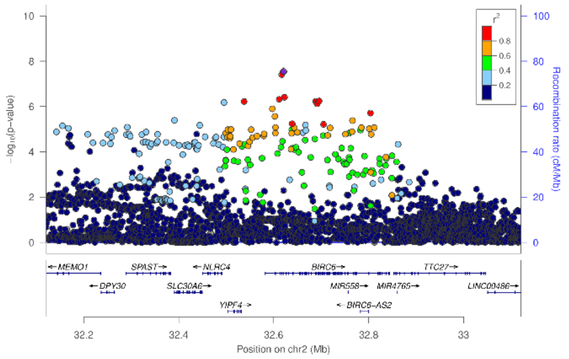

2:39061959–rs56350535

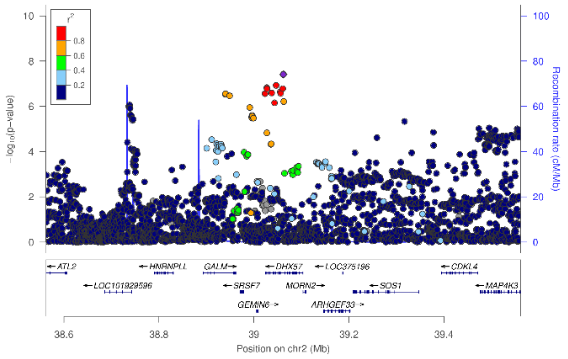

2:54738168–rs75243511

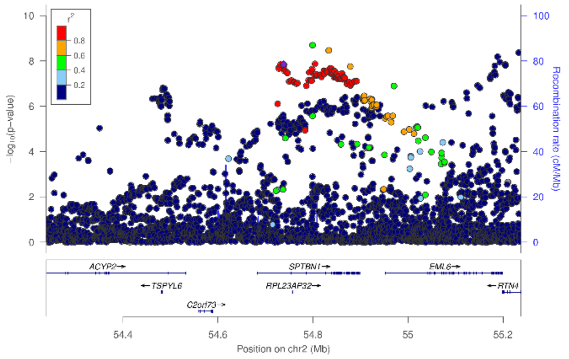

13:40671137–rs190533862

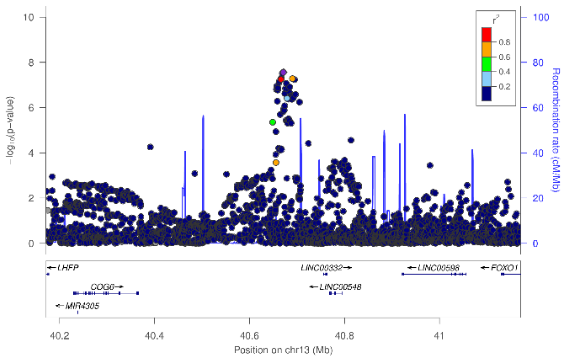

13:54264395–rs9596839

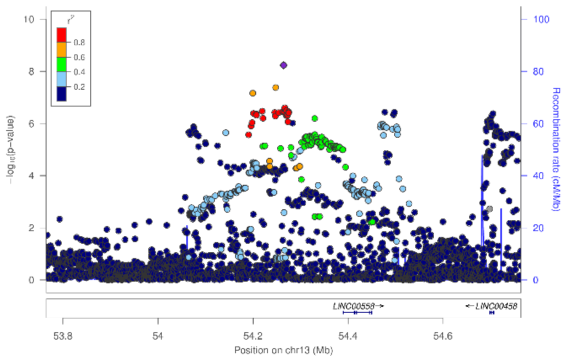

14:71352648–rs36563

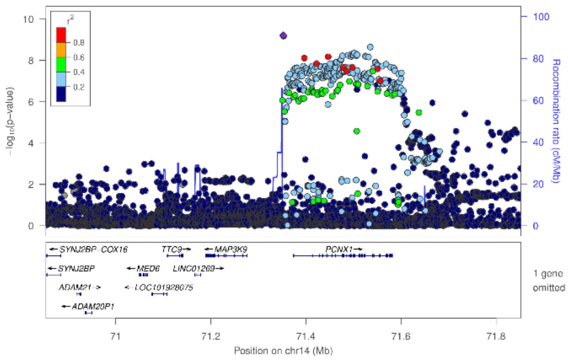

14:88825415–rs7160184

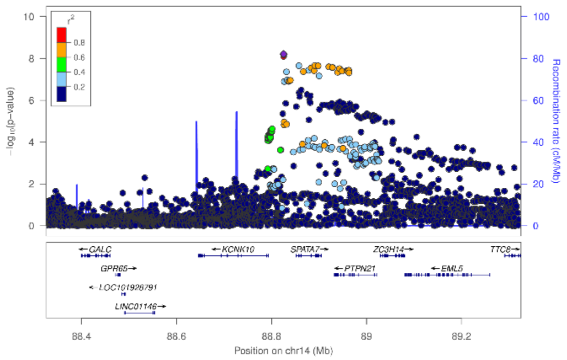

17:81036344–rs9675039

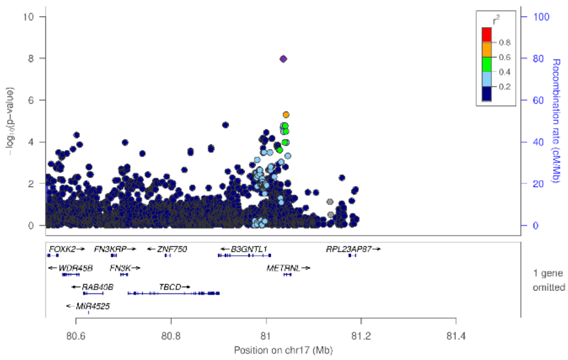

18:7131618–rs880132

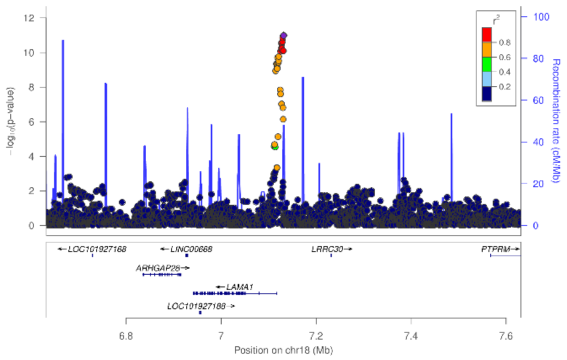

18:44040660–rs17766830

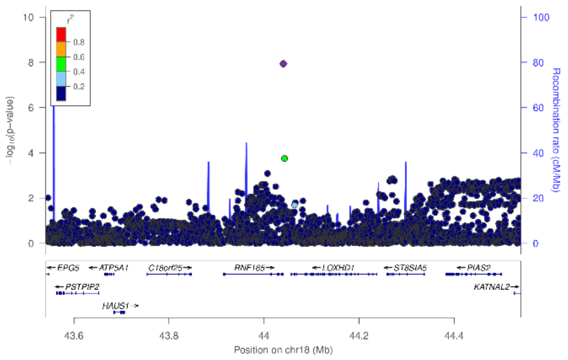

18:46461487–rs72917789

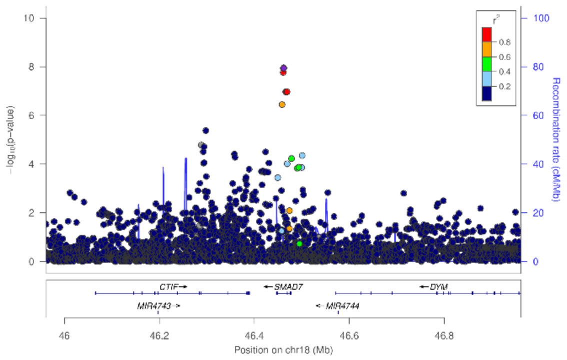

b

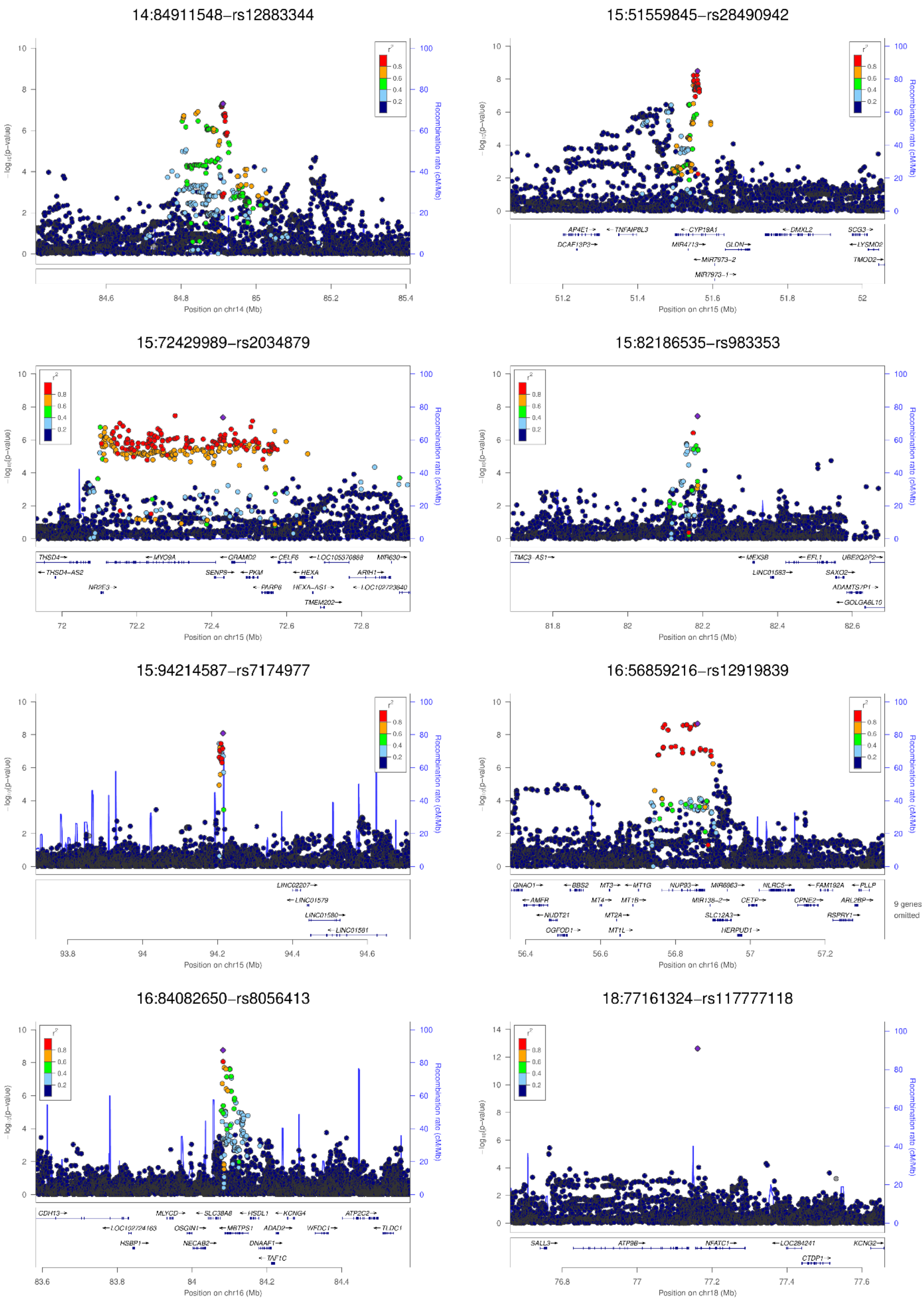
14:84911548–rs12883344
15:51559845–rs28490942
15:72429989–rs2034879
15:82186535–rs983353
15:94214587–rs7174977
16:56859216–rs12919839
16:84082650–rs8056413
18:77161324–rs117777118
−log10(p−value)
Recombination rate (cM/Mb)
r²
Position on chr14 (Mb)
Position on chr15 (Mb)
Position on chr16 (Mb)
Position on chr18 (Mb)
AP4E1
TNFAIP8L3
CYP19A1
DMXL2
SCG3
DCAF13P3
MIR4713
GLDN
LYSMD2
MIR7973−2
TMOD2
MIR7973−1
THSD4
MYO9A
GRAMD2
CELF6
LOC105370888
MIR630
THSD4−AS2
SENP8
PKM
HEXA
ARIH1
NR2E3
PARP6
HEXA−AS1
LOC102723640
TMEM202
TMC3−AS1
MEX3B
EFL1
UBE2Q2P2
LINC01583
SAXO2
ADAMTS7P1
GOLGA6L10
LINC02207
LINC01579
LINC01580
LINC01581
GNAO1
BBS2
MT3
MT1G
NUP93
MIR6863
NLRC5
FAM192A
PLLP
AMFR
MT4
MT1B
MIR138−2
CETP
CPNE2
ARL2BP
NUDT21
MT2A
SLC12A3
RSPRY1
OGFOD1
MT1L
HERPUD1
9 genes omitted
CDH13
MLYCD
SLC38A8
HSDL1
KCNG4
ATP2C2
LOC102724163
OSGIN1
MBTPS1
ADAD2
WFDC1
TLDC1
HSBP1
NECAB2
DNAAF1
TAF1C
SALL3
ATP9B
NFATC1
LOC284241
KCNG2
CTDP1

11:108350451–rs11212666

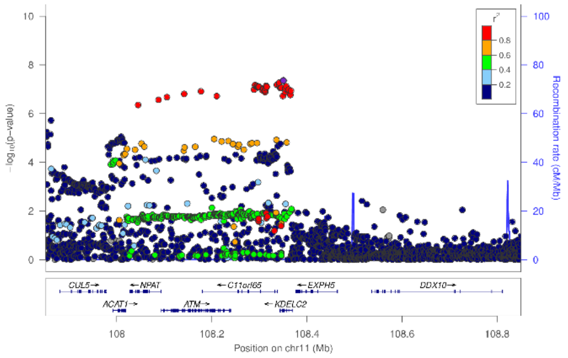

11:124619407–rs11604175

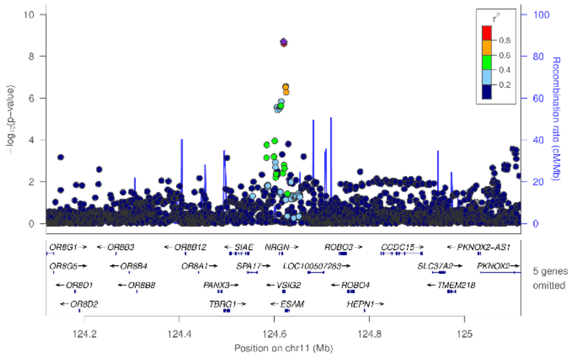

11:128769876–rs3765618

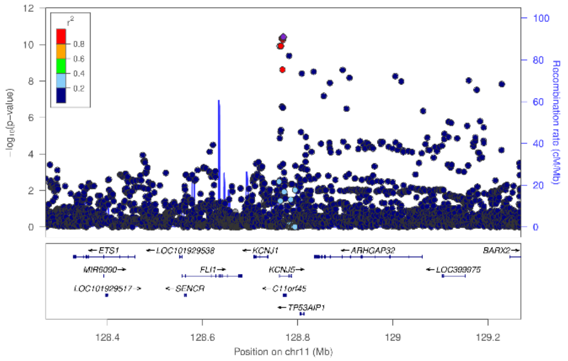

12:52418075–rs1732235

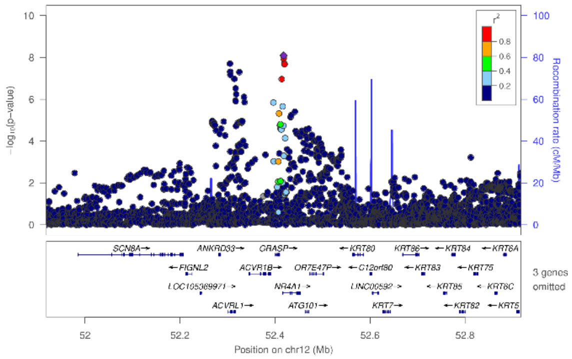

13:38249726–rs56312513

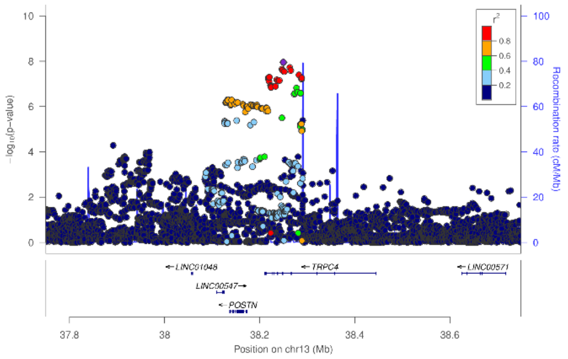

14:21841154–rs7350752

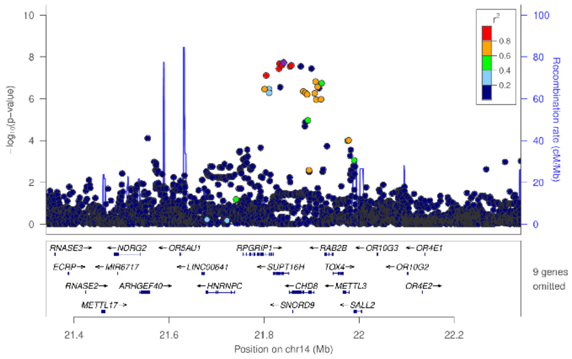

14:59900020–rs2774052

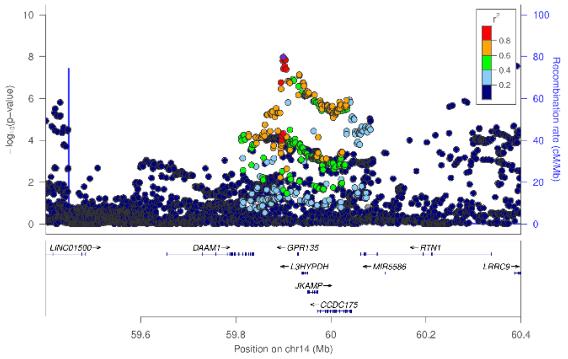

14:71874638–rs2041330

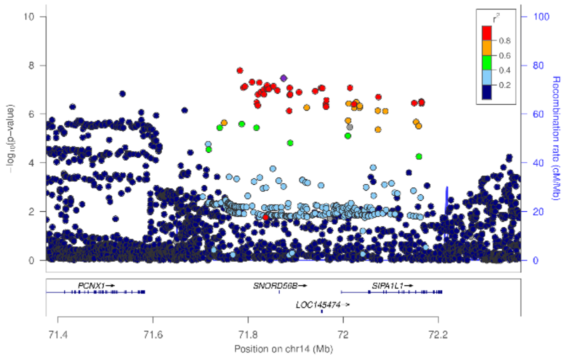

6:10034452–rs9477605

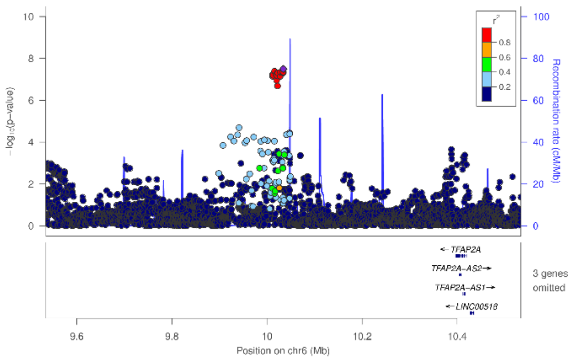

6:17477425–rs9370995

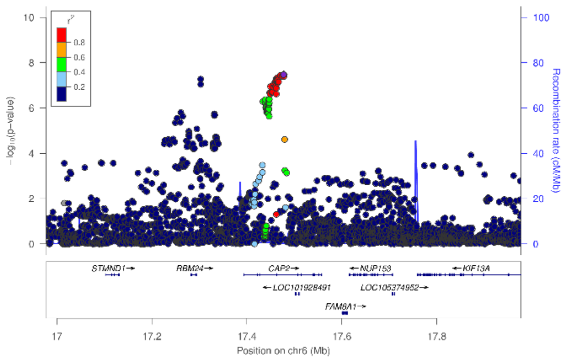

6:100629078–rs57989773

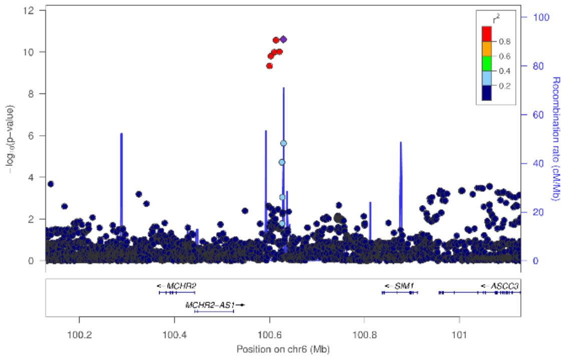

6:109625797–rs1546722

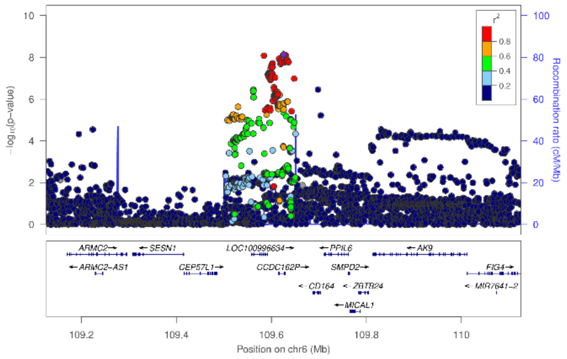

8:49391836–rs10087280

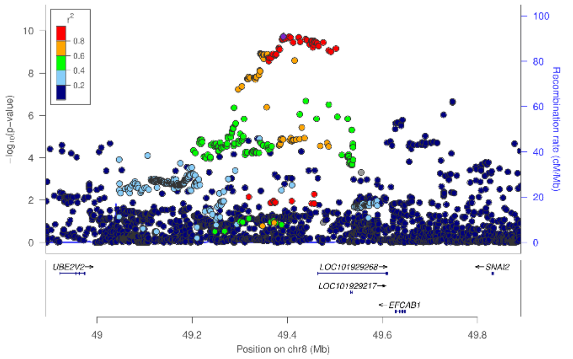

8:134229535–rs6982341

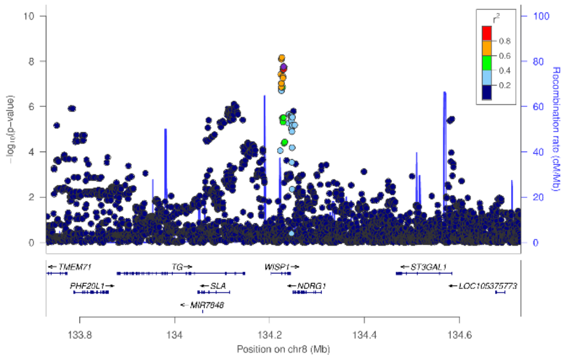

9:129643296–rs10819246

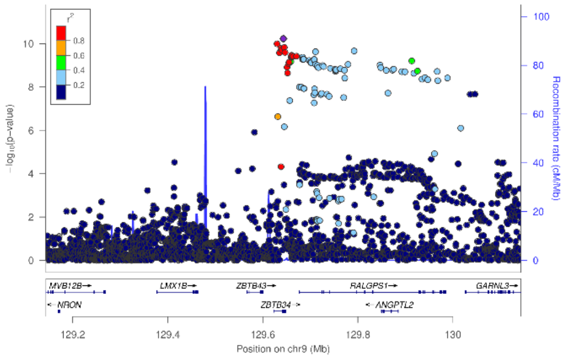

11:96151677–rs61909958

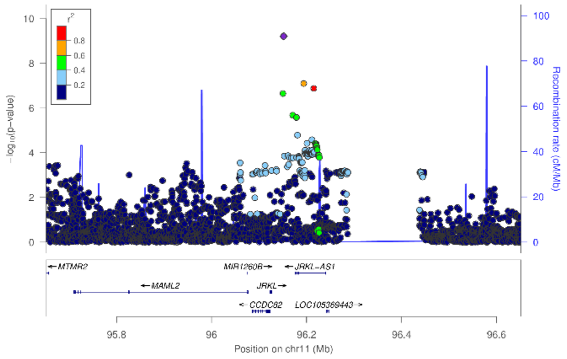

3:136692308–rs73231988

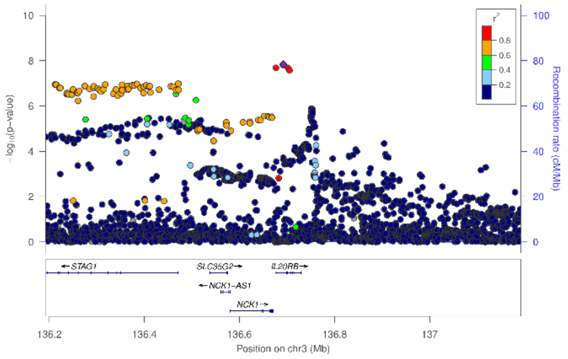

4:72002332–rs6822301

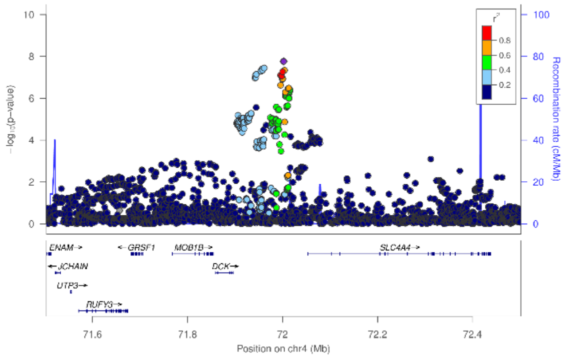

4:152163489–rs7671332

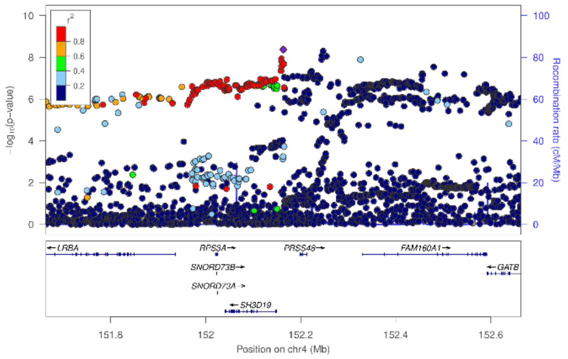

4:187818466–rs9685837

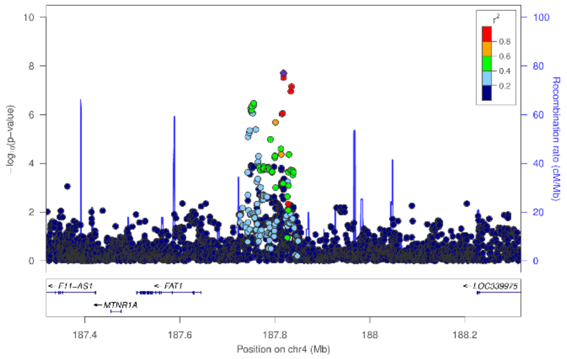

5:38616887–rs172906

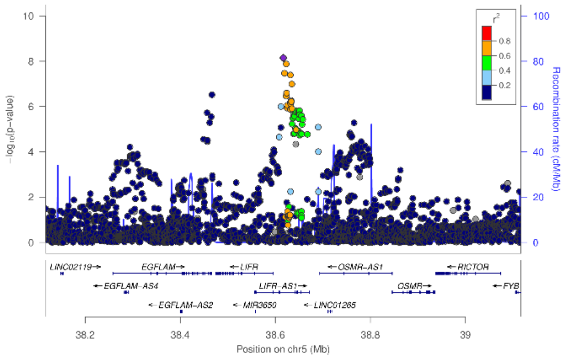

5:42515027–rs62370646

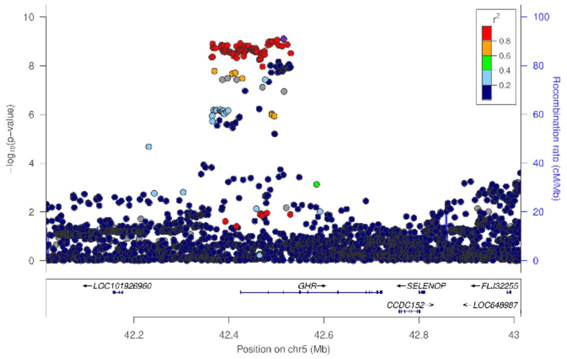

5:58352210–rs10061553

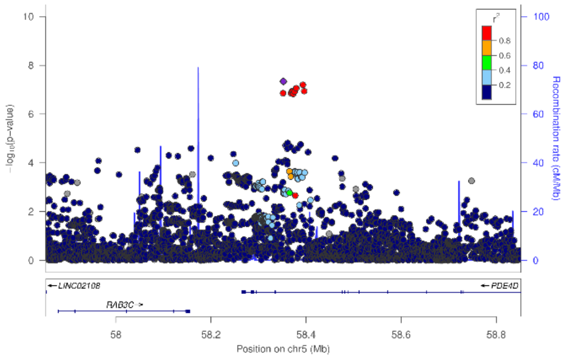

5:76884661–rs34237622

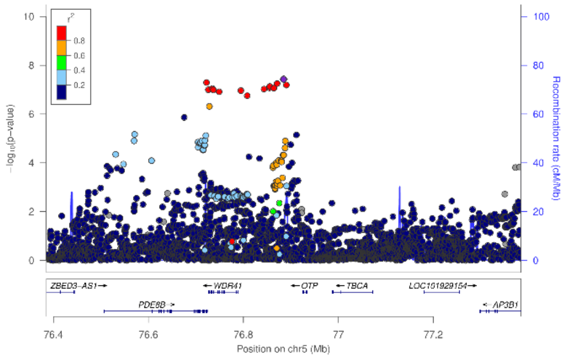

1:21155195–rs2320590

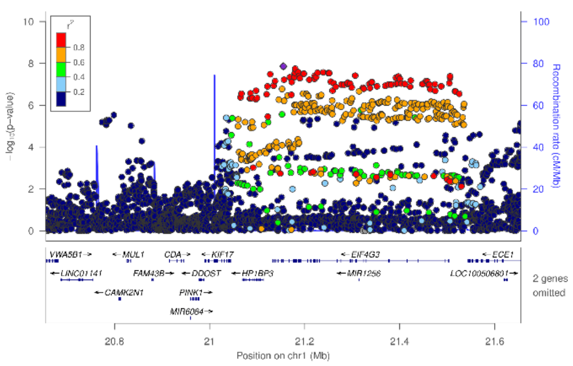

1:68143195–rs10889711

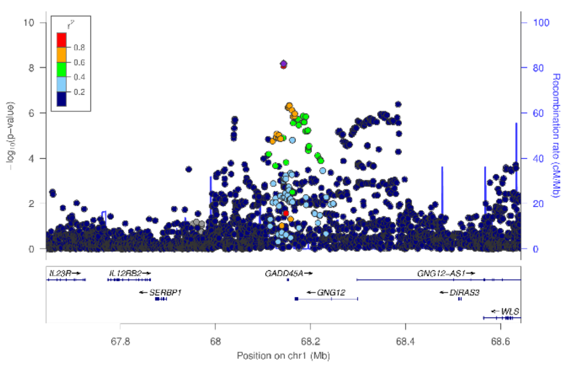

1:110229787–rs36209093

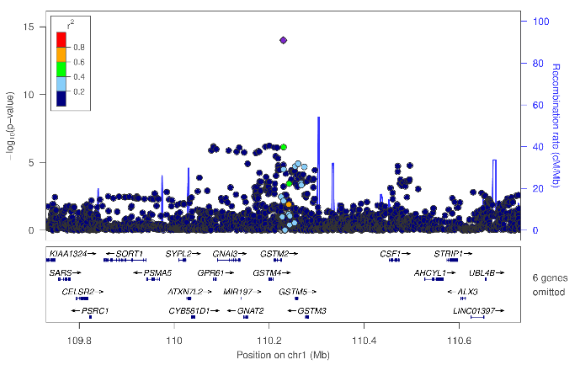

1:112261533–rs565522

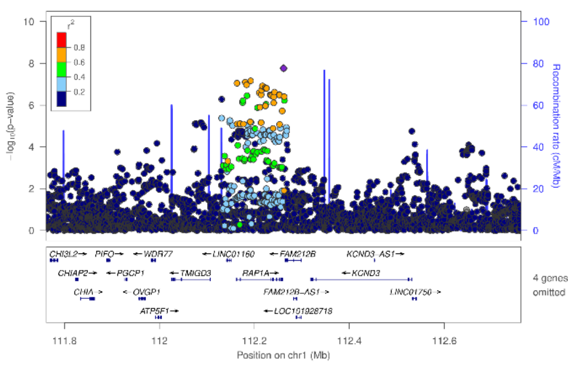

1:118223275–rs6669446

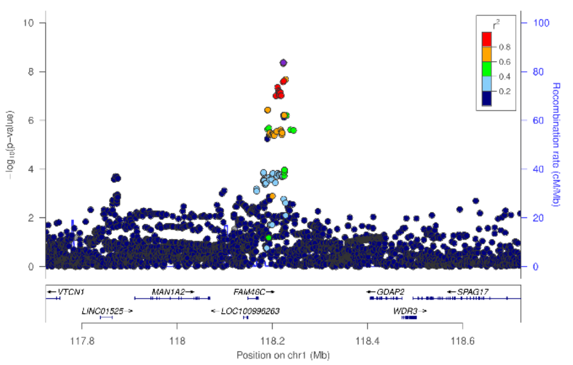

2:9803203–rs57503539

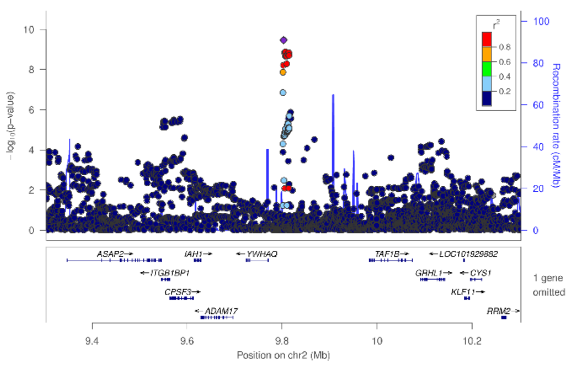

3:25424929–rs2306623

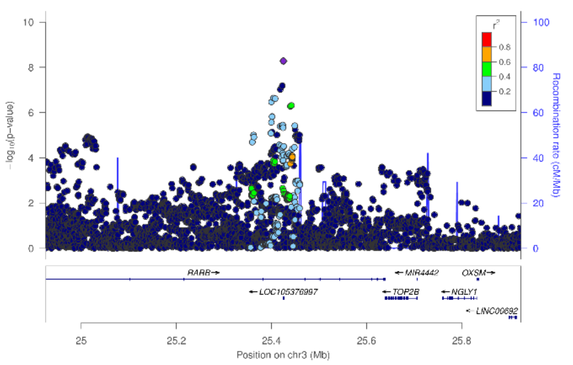

3:117492152–rs6805393

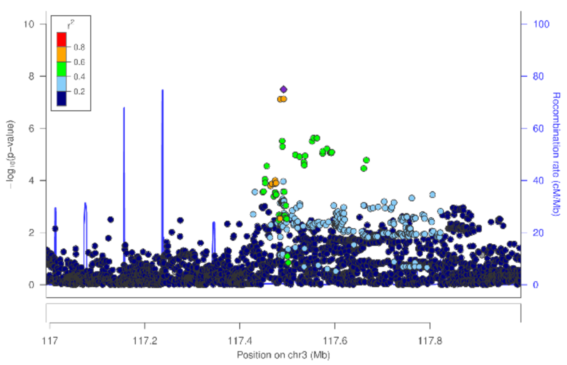

19:44746657–rs2125578

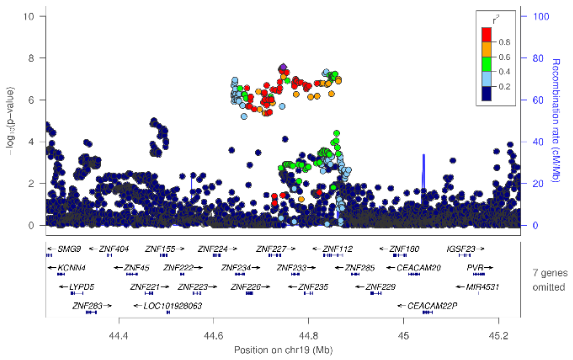

20:35169916–rs146827176

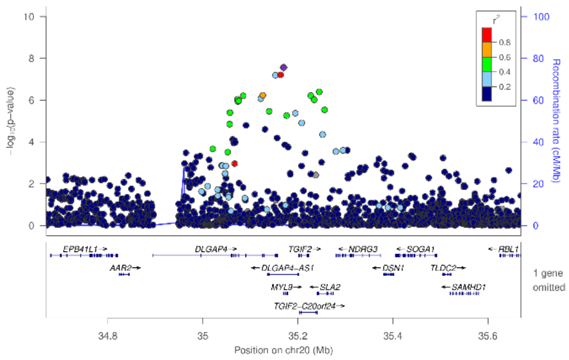

c

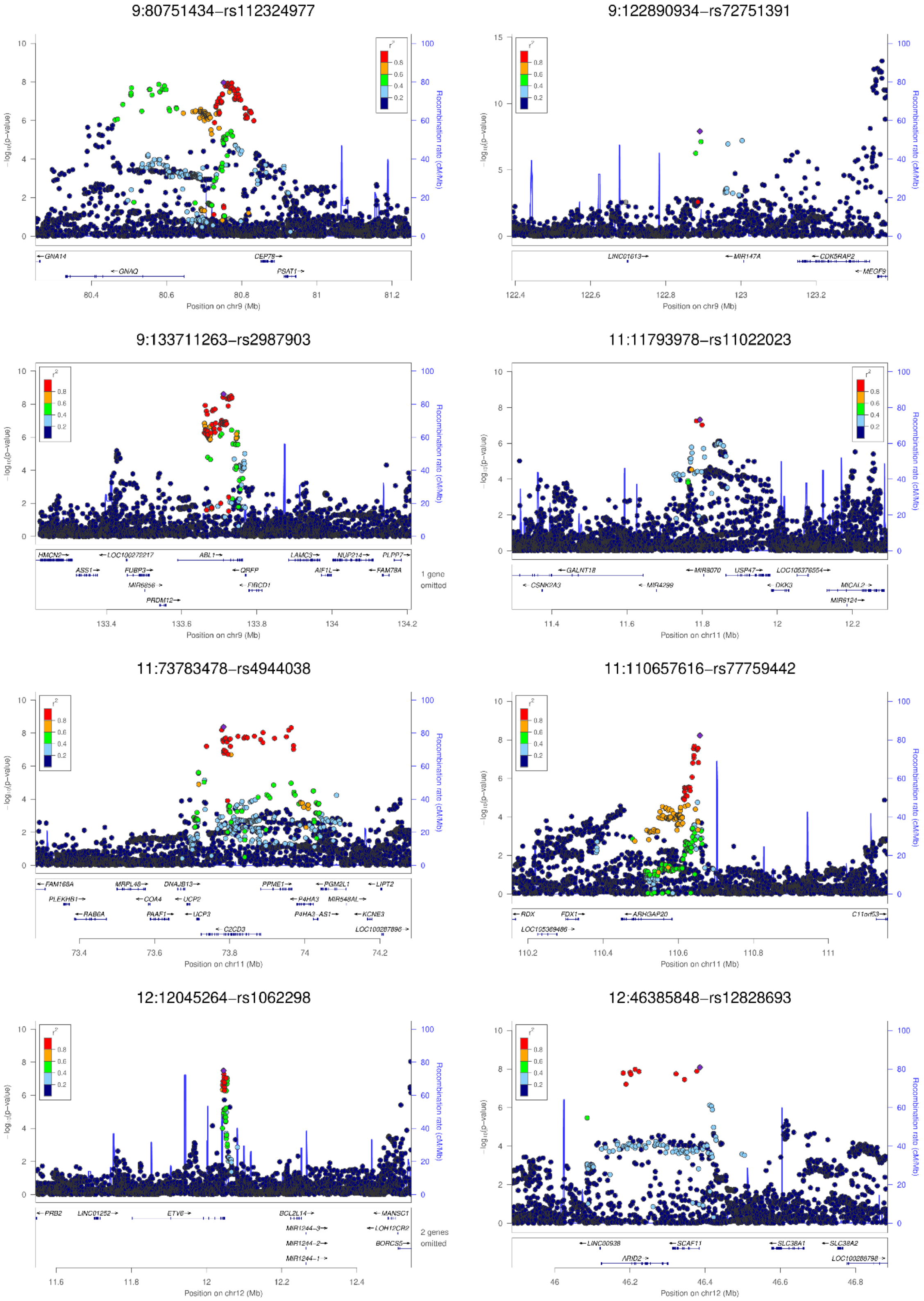
9:80751434–rs112324977
GNA14
GNAQ
CEP78
PSAT1
Position on chr9 (Mb)
9:122890934–rs72751391
LINC01613
MIR147A
CDK5RAP2
MEGF9
Position on chr9 (Mb)
9:133711263–rs2987903
HMCN2
LOC100272217
ABL1
LAMC3
NUP214
PLPP7
ASS1
FUBP3
QRFP
AIF1L
FAM78A
MIR6856
FIBCD1
PRDM12
1 gene omitted
Position on chr9 (Mb)
11:11793978–rs11022023
GALNT18
MIR8070
USP47
LOC105376554
CSNK2A3
MIR4299
DKK3
MICAL2
MIR6124
Position on chr11 (Mb)
11:73783478–rs4944038
FAM168A
MRPL48
DNAJB13
PPME1
PGM2L1
LIPT2
PLEKHB1
COA4
UCP2
P4HA3
MIR548AL
RAB6A
PAAF1
UCP3
P4HA3-AS1
KCNE3
C2CD3
LOC100287896
Position on chr11 (Mb)
11:110657616–rs77759442
RDX
FDX1
ARHGAP20
C11orf53
LOC105369486
Position on chr11 (Mb)
12:12045264–rs1062298
PRB2
LINC01252
ETV6
BCL2L14
MANSC1
MIR1244-3
LOH12CR2
MIR1244-2
BORCS5
MIR1244-1
2 genes omitted
Position on chr12 (Mb)
12:46385848–rs12828693
LINC00938
SCAF11
SLC38A1
SLC38A2
ARID2
LOC100288798
Position on chr12 (Mb)
−log10(p-value)
Recombination rate (cM/Mb)
r2

6:99548729–rs72943226

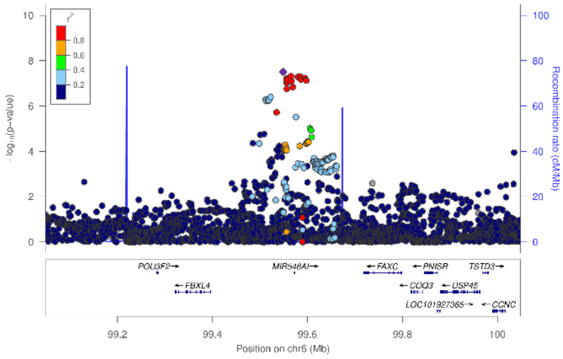

6:121258543–rs9320778

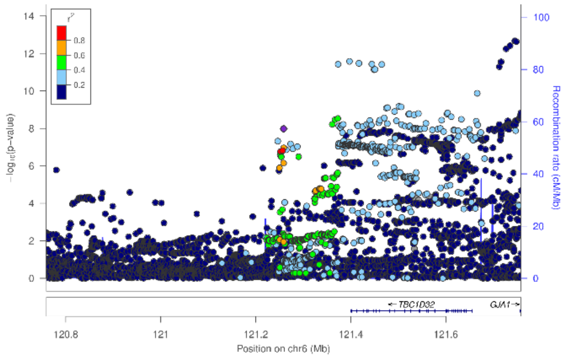

7:15421023–rs67615620

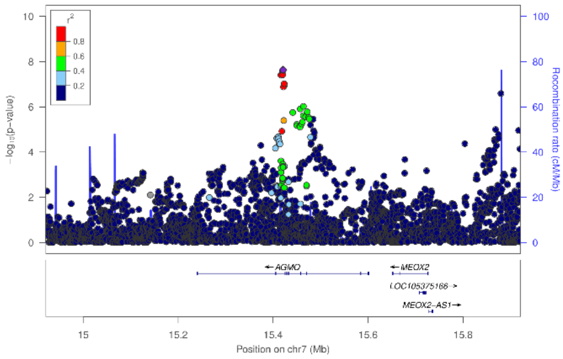

7:16117030–rs75177877

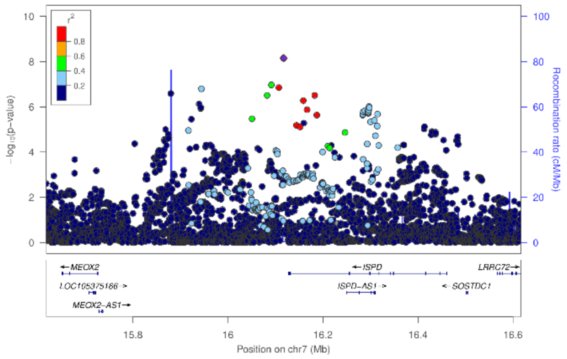

8:34164285–rs2953937

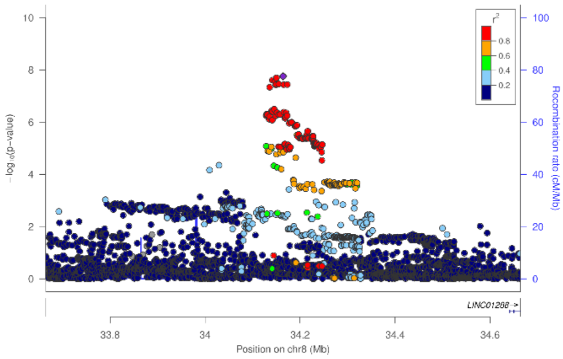

8:108319395–rs36036692

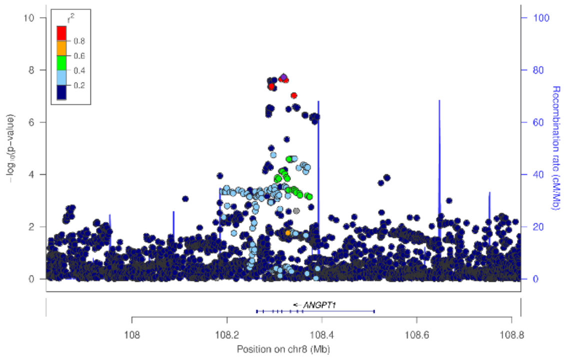

9:14535119–rs34361301

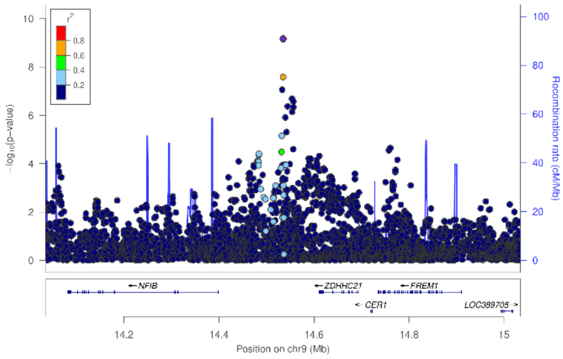

9:35191014–rs61241090

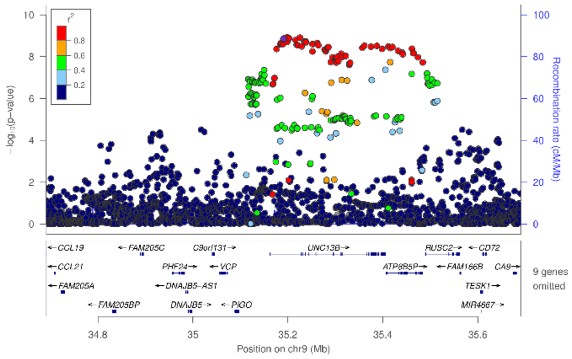

1:40763095–rs12134085

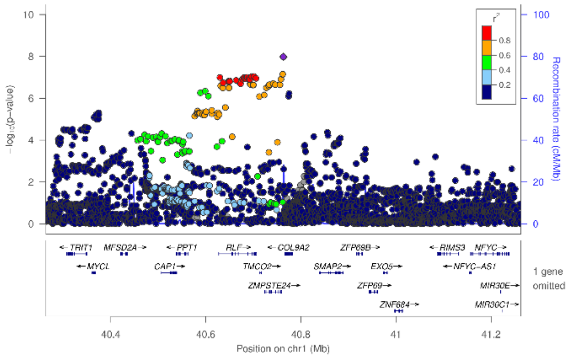

1:72229240–rs12084868

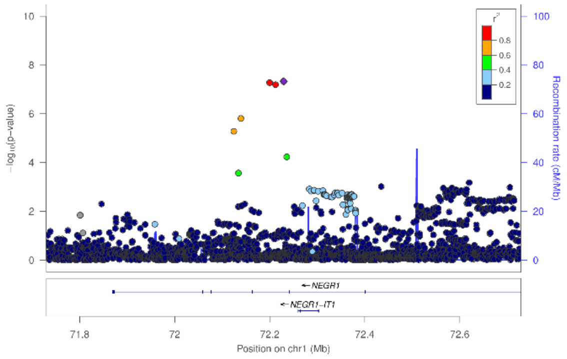

1:115019239–rs71664847

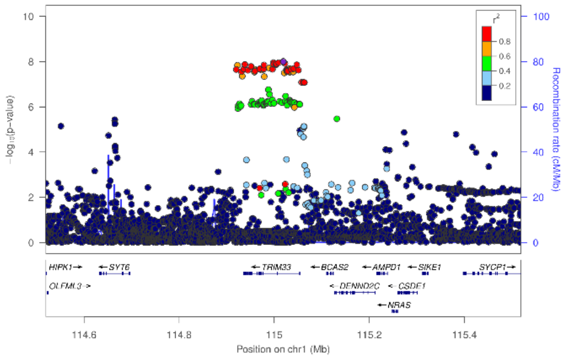

2:209622–rs300753

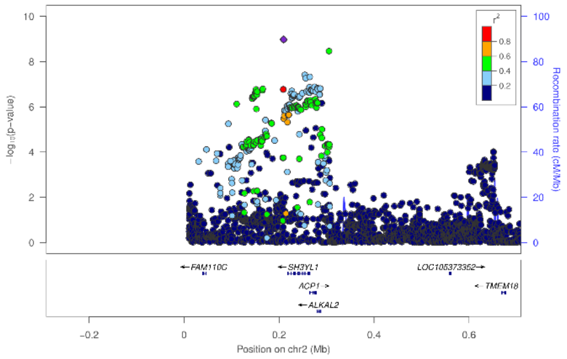

2:196590414–rs10208493

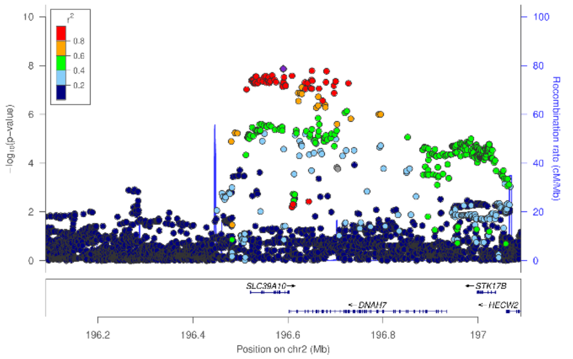

3:69919744–rs62253186

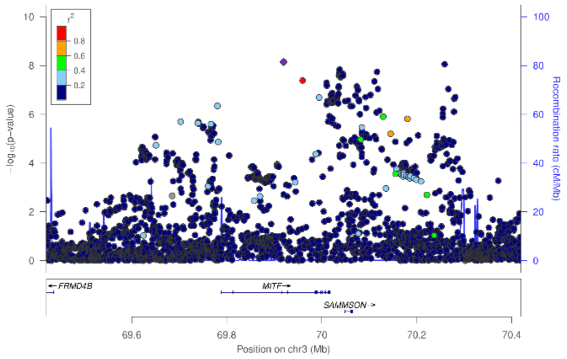

3:187456904–rs3821817

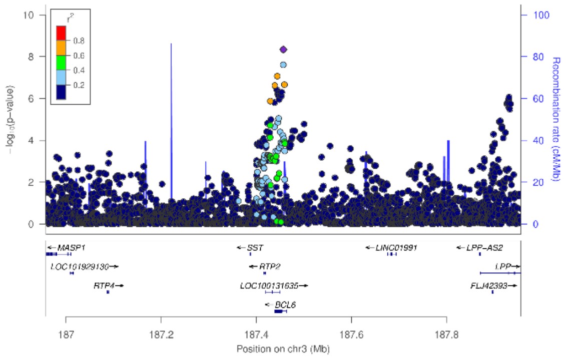

6:85988429–rs4053778

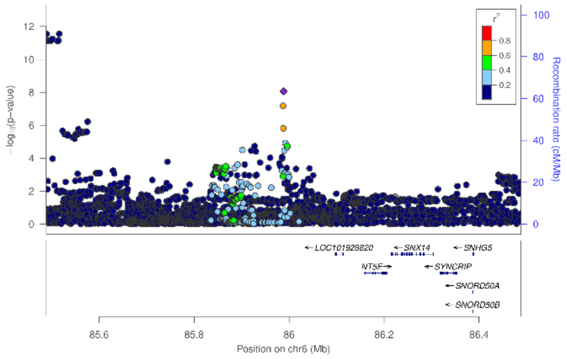

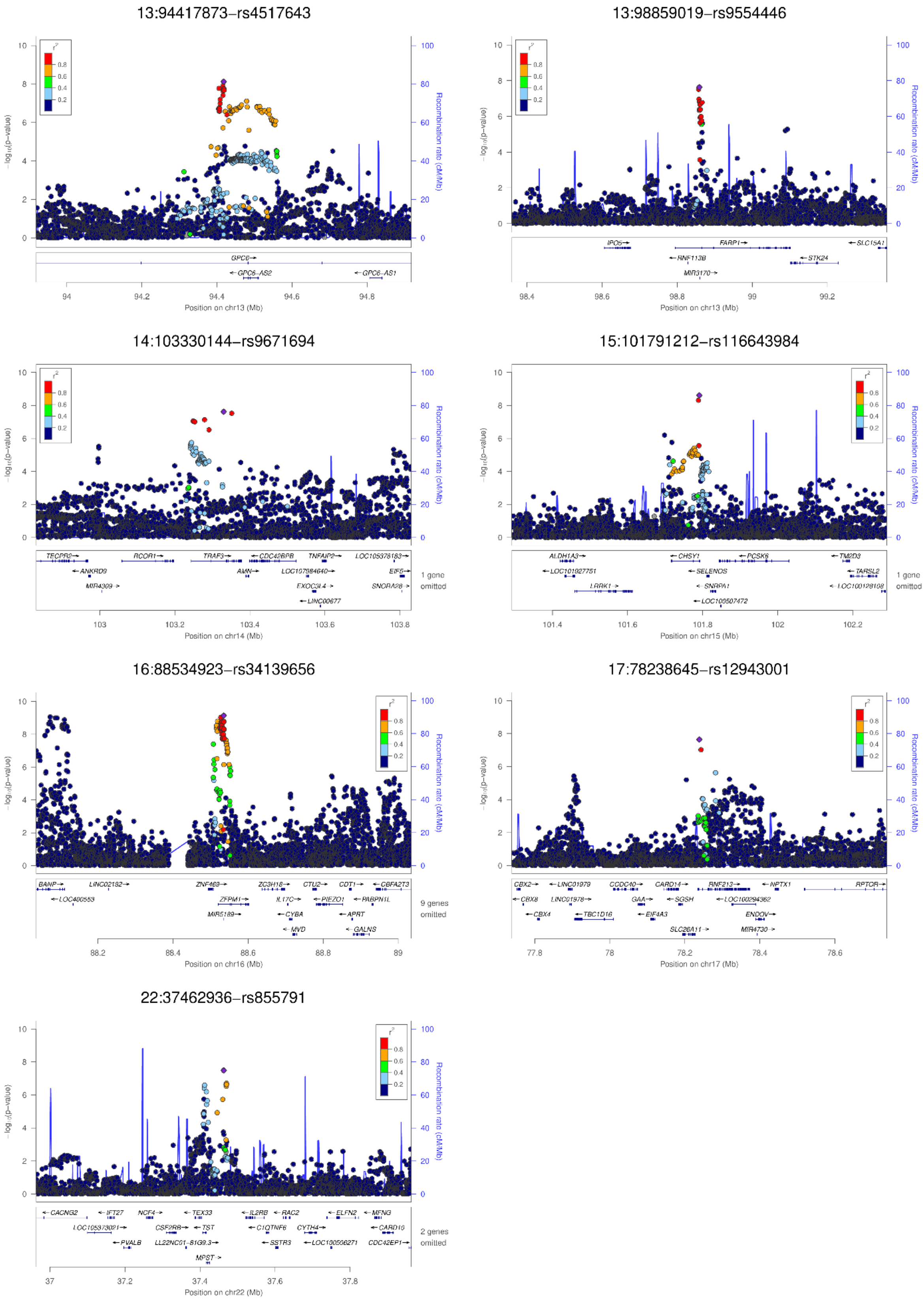

Supplementary Figure 3a-c. Locus Zoom plots for all novel loci for systolic (a), diastolic (b), and pulse pressure (c). P-values are from the inverse variance-weighted meta-analyses for each blood pressure trait and presented in logarithmic scale.

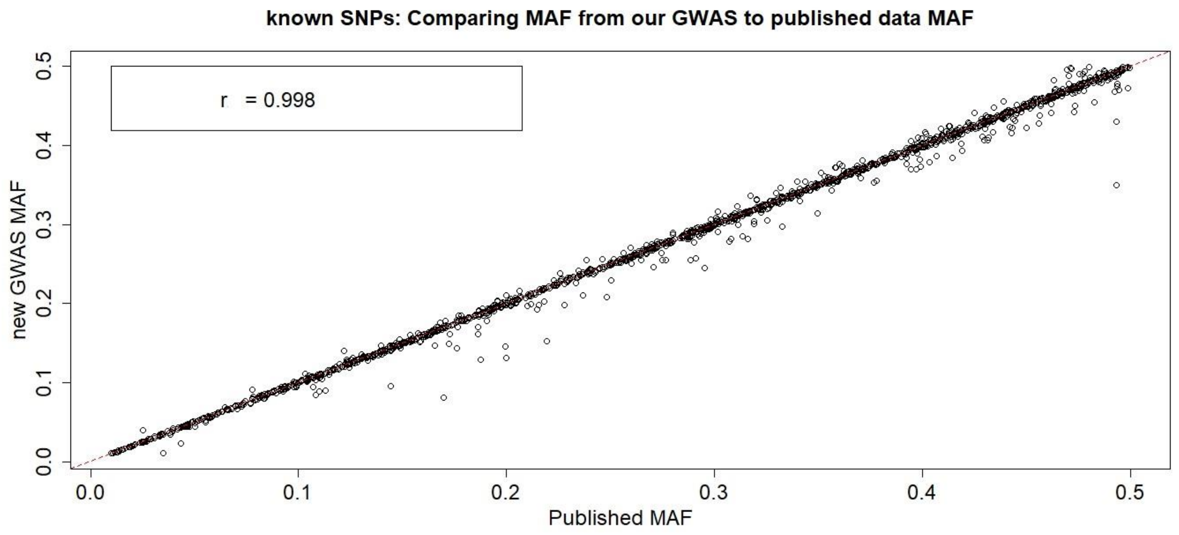

Supplementary Figure 4. Known SNPs: Comparing MAF from our GWAS to published data MAF. The x-axis shows the published MAF according to data taken from the publication where SNP is first reported (see PMID in Sup Table of all 3,800 known SNPs). The y-axis shows the MAF from our GWAS meta-analysis. The correlation between the published and observed MAF values is shown in the plot legend. For fair comparison, SNPs shown in this plot are restricted only to known SNPs published from main-effect primary GWAS analyses (i.e. excluding SNPs from conditional analyses, stratified or interaction analyses) in studies of predominantly European ancestry, with MAF > 1%, covered in our GWAS meta-analysis data, and with available data on both MAF and untransformed Effect Sizes on the mmHg scale reported in the original publication's published Tables, resulting in 1,483 SNPs plotted of the total 3,800 published SNPs. MAF: Minor Allele Frequency; GWAS: Genome-Wide Association Study; r: Pearson's Correlation Coefficient

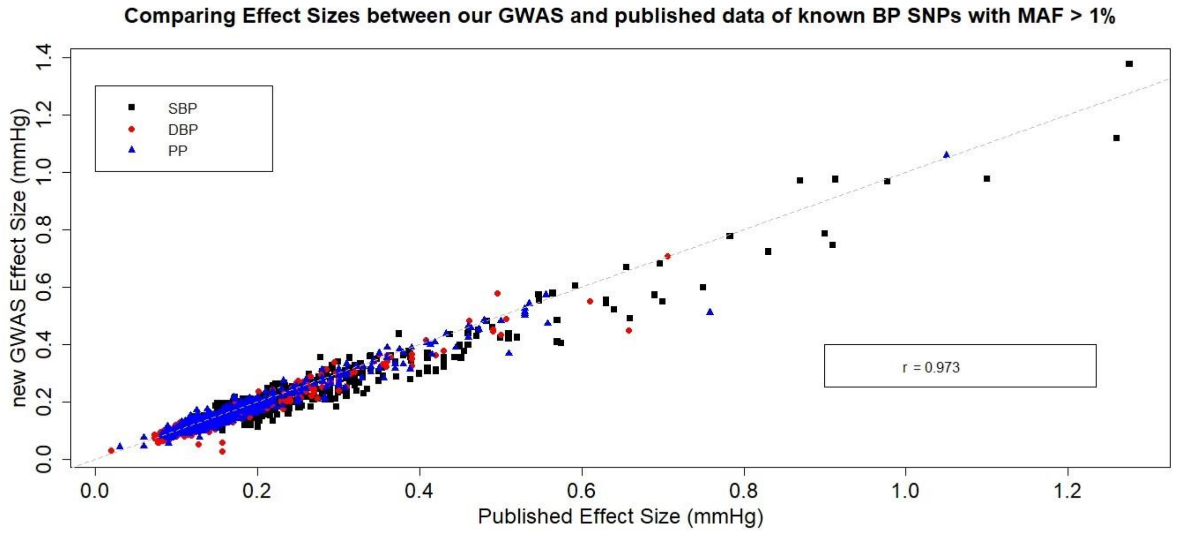

Supplementary Figure 5. Comparing Effect Sizes between our GWAS and published data of known BP SNPs. The x-axis shows the magnitude of the published effect size estimate (mmHg) according to data taken from the publication where SNP is first reported (see PMID

in Sup Table of all 3,800 known SNPs). The y-axis shows the magnitude of the effect size estimate (mmHg) from our GWAS meta-analysis. Each SNP is only plotted once according to the primary, most significant BP trait that the SNP was reported for from the original publication, as indicated by the differentiating colour-coded plot shapes per BP trait, explained in the plot legend. The correlation between the published and observed effect sizes is shown in the plot legend. For fair comparison, SNPs shown in this plot are restricted only to known SNPs published from main-effect primary GWAS analyses (i.e. excluding SNPs from conditional analyses, stratified or interaction analyses) in studies of predominantly European ancestry, with MAF > 1%, covered in our GWAS meta-analysis data, and with available data on both MAF and untransformed Effect Sizes on the mmHg scale reported in the original publication's published Tables, resulting in 1,483 SNPs plotted of the total 3,800 published SNPs. BP: blood pressure; MAF: Minor Allele Frequency; GWAS: Genome-Wide Association Study; r: Pearson's Correlation Coefficient

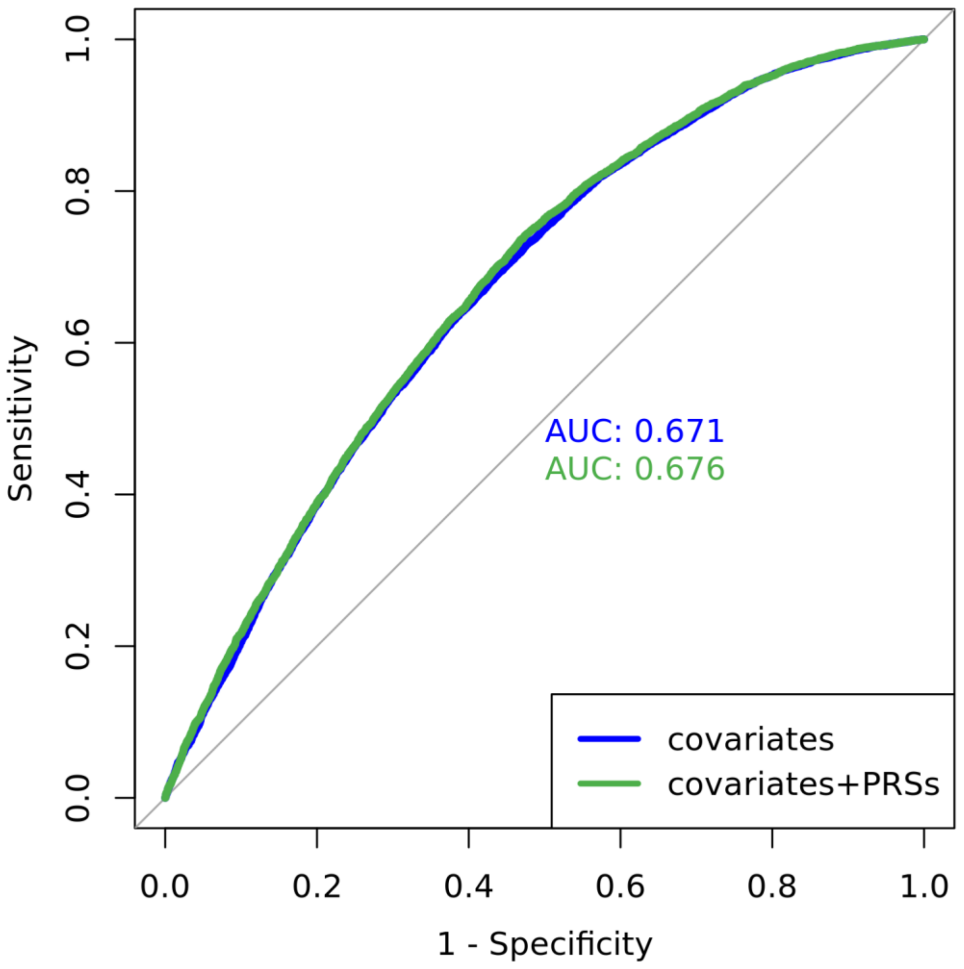

Supplementary Figure 6: PRS AUROC in AA: Area under the ROC curve (AUROC) of the two models (covariates only and covariates plus SBayesRC PRS) for HTN in African-American ancestry sub-sample of the All-Of-Us cohort (n=21,843).

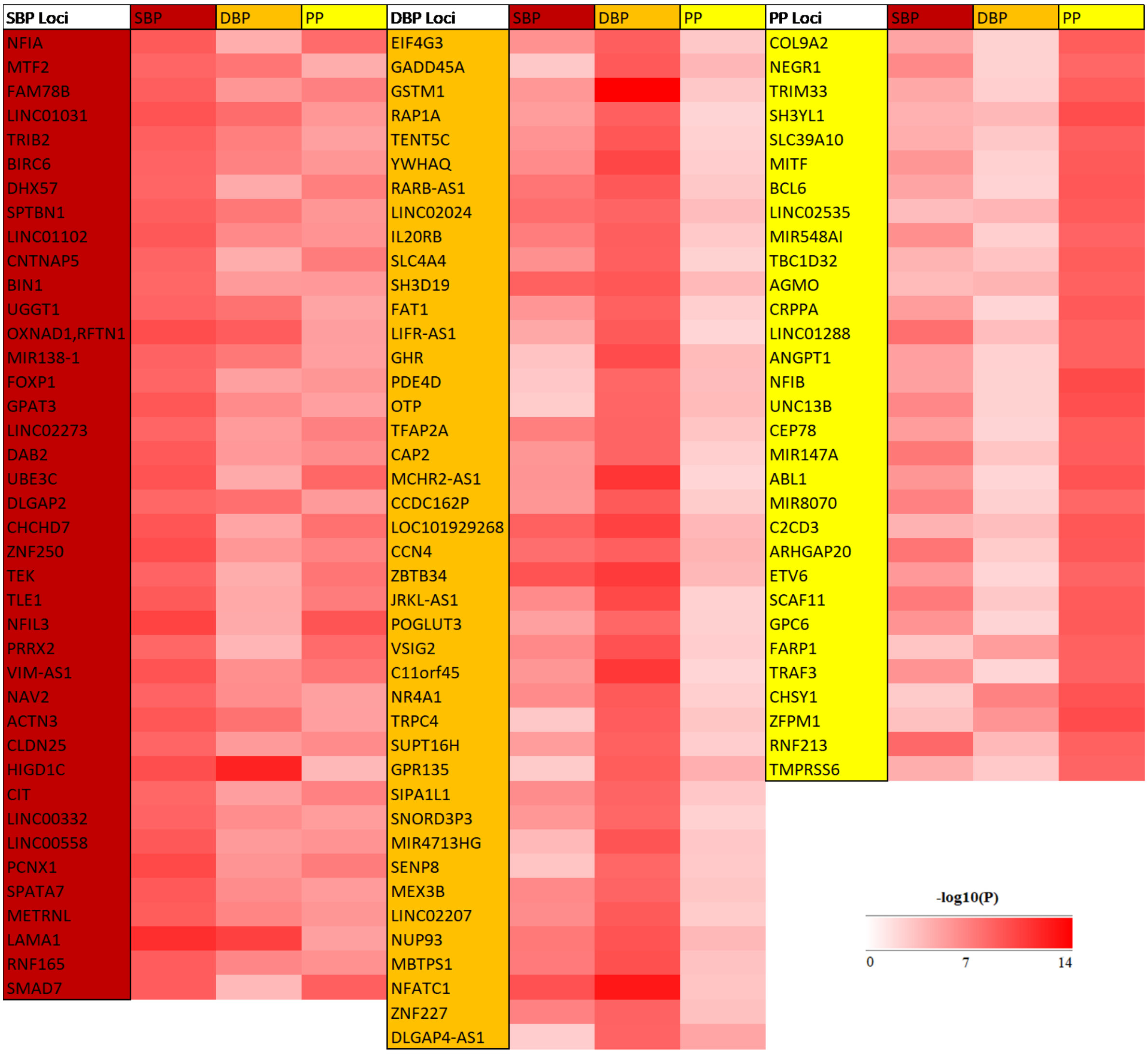

Supplementary Figure 7. Heatmap comparing significance of associations for 113 BP (Blood Pressure) novel loci across the other BP traits: SBP = systolic BP; DBP = diastolic BP; PP = Pulse Pressure. P-values are from the inverse variance-weighted meta-analyses for each blood pressure trait and presented in logarithmic scale.

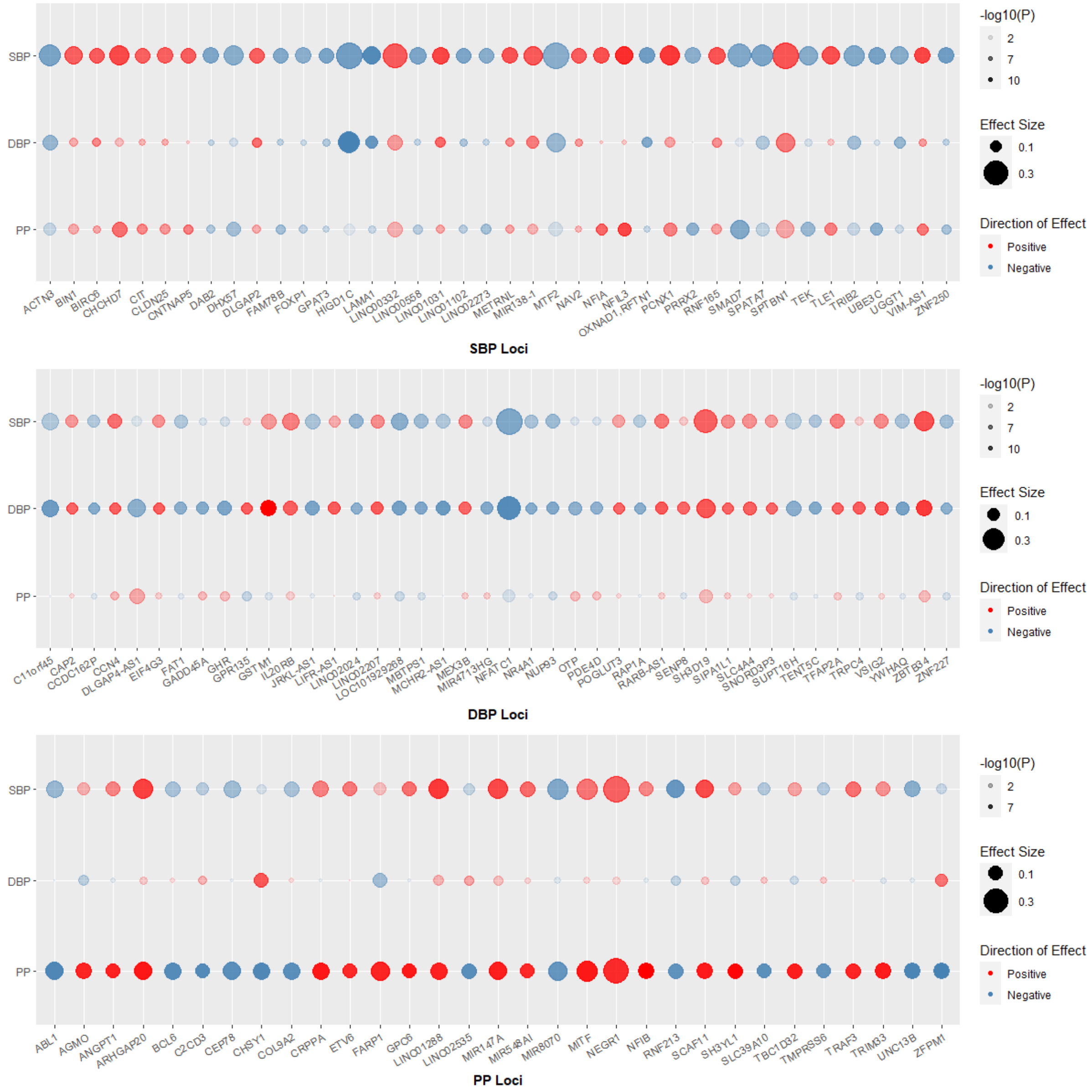

Supplementary Figure 8a-c. Bubble plots of associations for 113 Blood Pressure (BP) novel loci across other BP traits: SBP = systolic BP; DBP = diastolic BP; PP = Pulse Pressure. P-values are from the inverse variance-weighted meta-analyses for each blood pressure trait and presented in logarithmic scale.

a

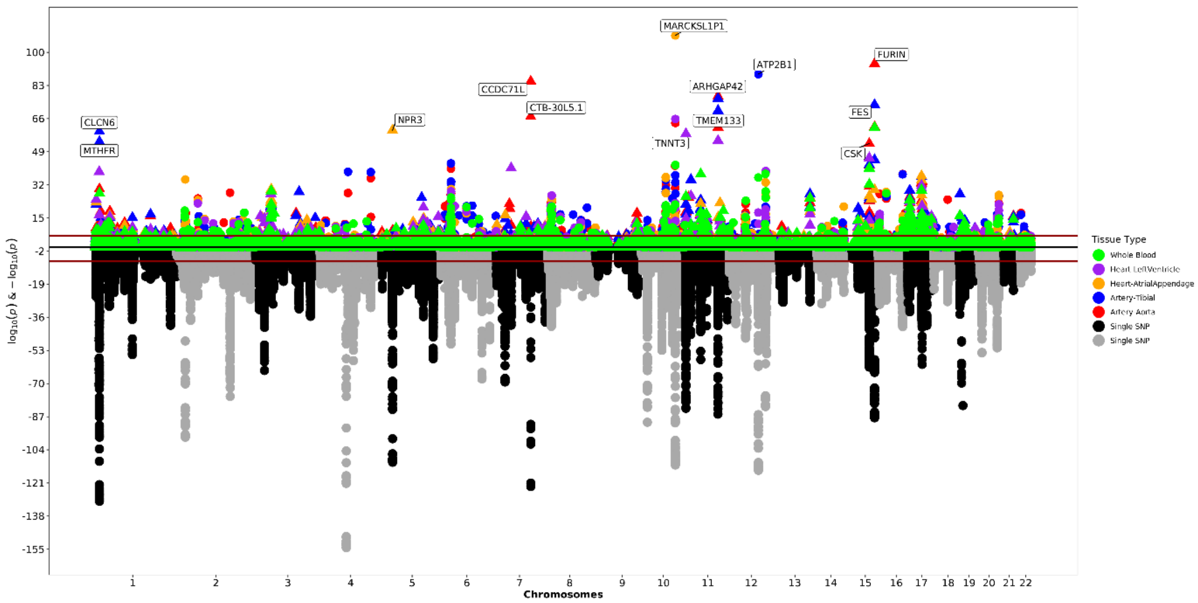

MARCKSL1P1
ATP2B1
FURIN
CCDC71L
ARHGAP42
CTB-30L5.1
CLCN6
NPR3
TMEM133
FES
TNNT3
MTHFR
CSK
log10(p) & −log10(p)
Chromosomes
Tissue Type
Whole Blood
Heart LeftVentricle
Heart-AtrialAppendage
Artery-Tibial
Artery Aorta
Single SNP
Single SNP

b

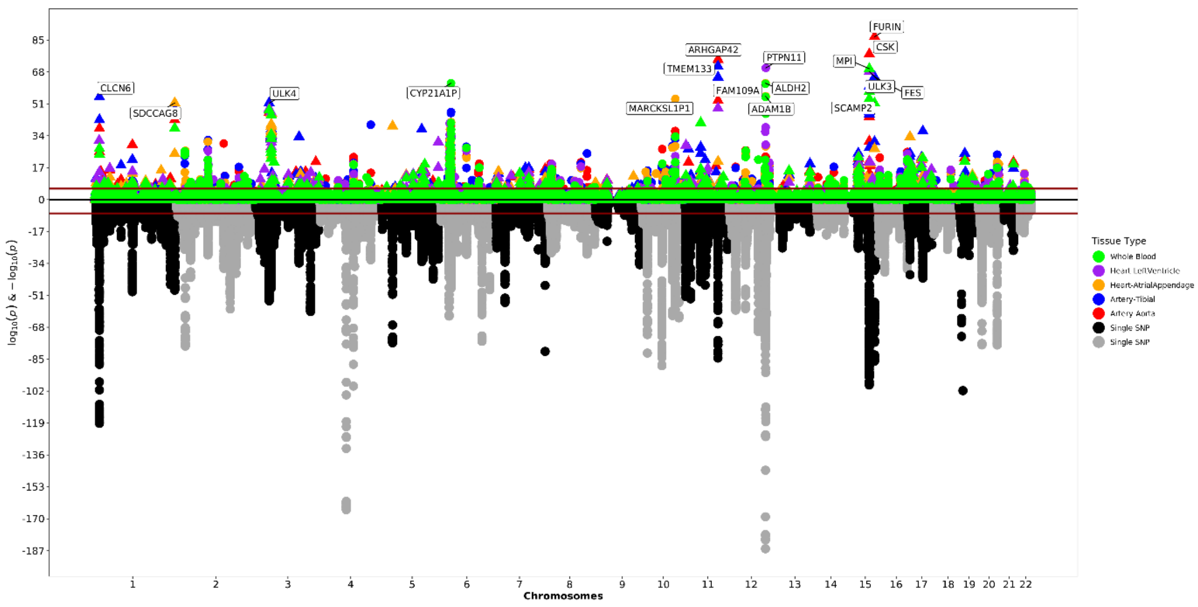

FURIN
CSK
ARHGAP42
PTPN11
MPI
TMEM133
CLCN6
FAM109A
ALDH2
ULK4
CYP21A1P
ULK3
FES
SDCCAG8
MARCKSL1P1
ADAM1B
SCAMP2
log10(p) & −log10(p)
Chromosomes
Tissue Type
Whole Blood
Heart LeftVentricle
Heart-AtrialAppendage
Artery-Tibial
Artery Aorta
Single SNP
Single SNP

c

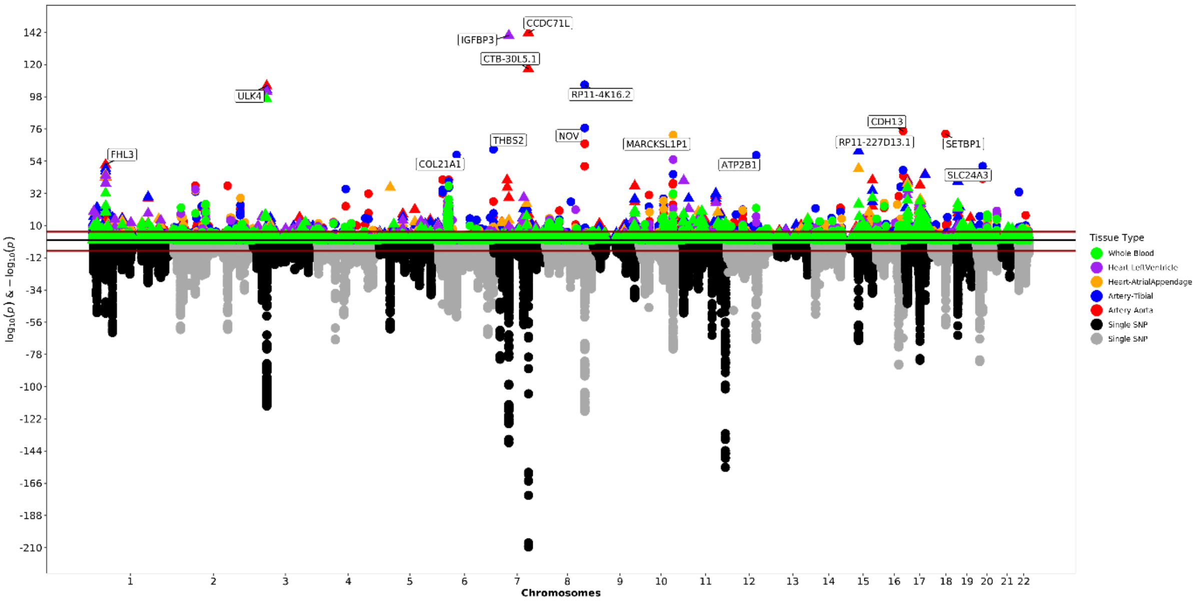

Supplementary Figure 9a-c. Opposed Manhattan plots for S-PrediXcan (–log10p) and GWAS (log10p) for BP-traits. –Log$_{10}$ p-values for associations between genetically predicted gene expression analyses with BP-traits in 5 tissues are juxtaposed with log$_{10}$ p-values from the inverse variance-weighted GWAS meta-analyses for SBP (a); DBP (b) and PP (c).

a

Tissue Specificity Test

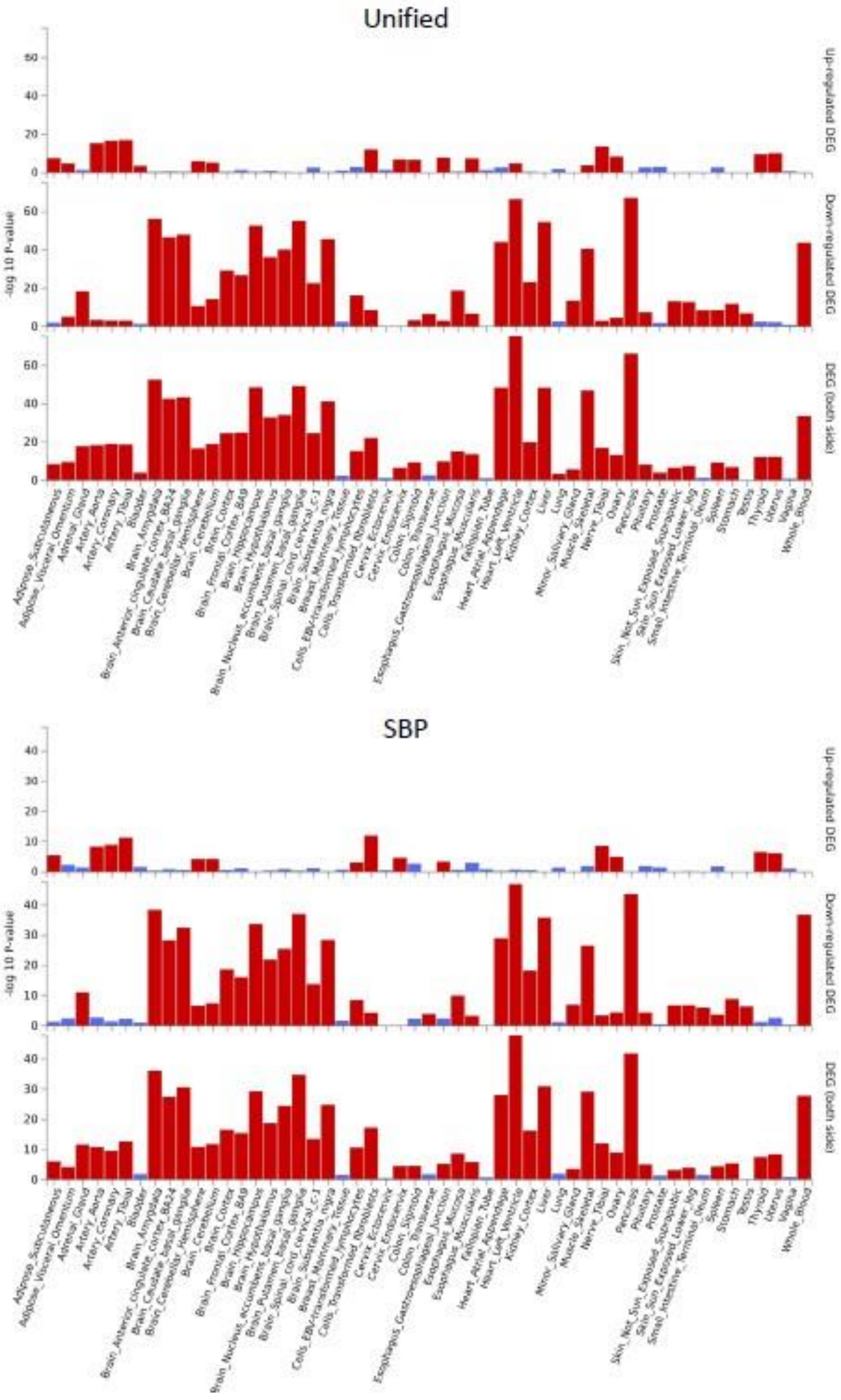

b

Tissue Specificity Test

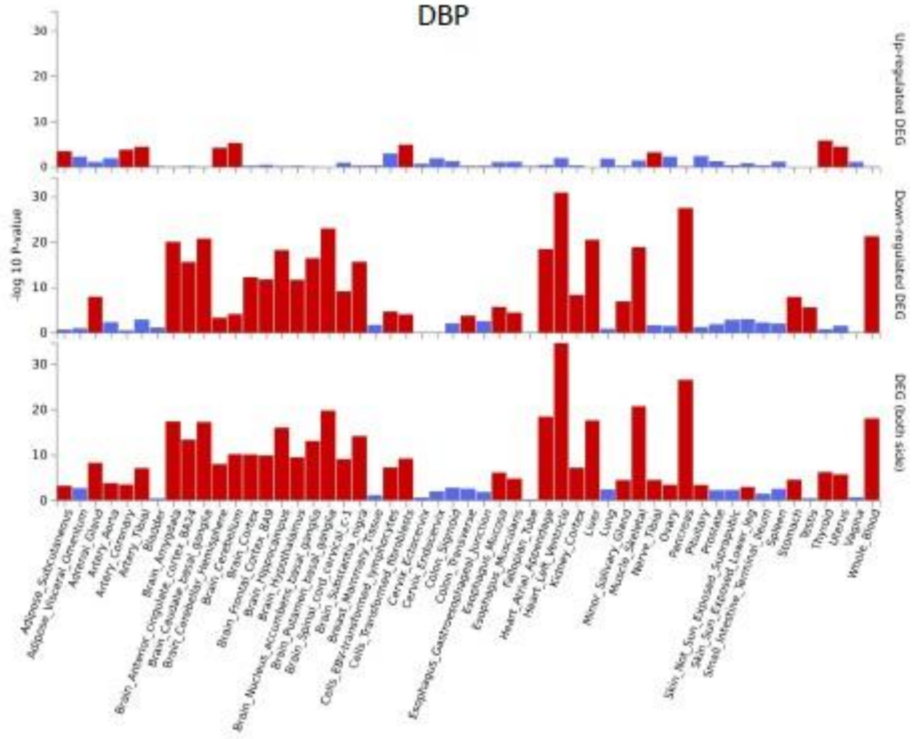

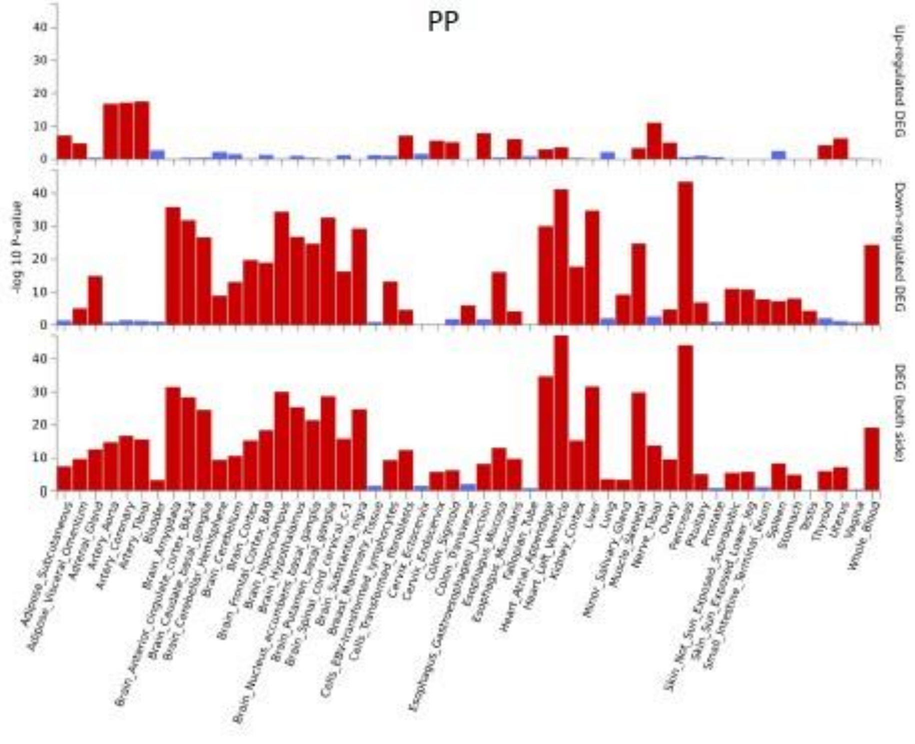

Supplementary Figure 10: Results Output from FUMA Pathway Analyses: Tissue Specificity Test: (a) Unified Blood Pressure (BP) and Systolic BP (SBP); (b) Diastolic BP (DBP) and Pulse Pressure (PP). P-values of enrichment tests in different tissues are given on the y-axis in logarithmic scale.

a

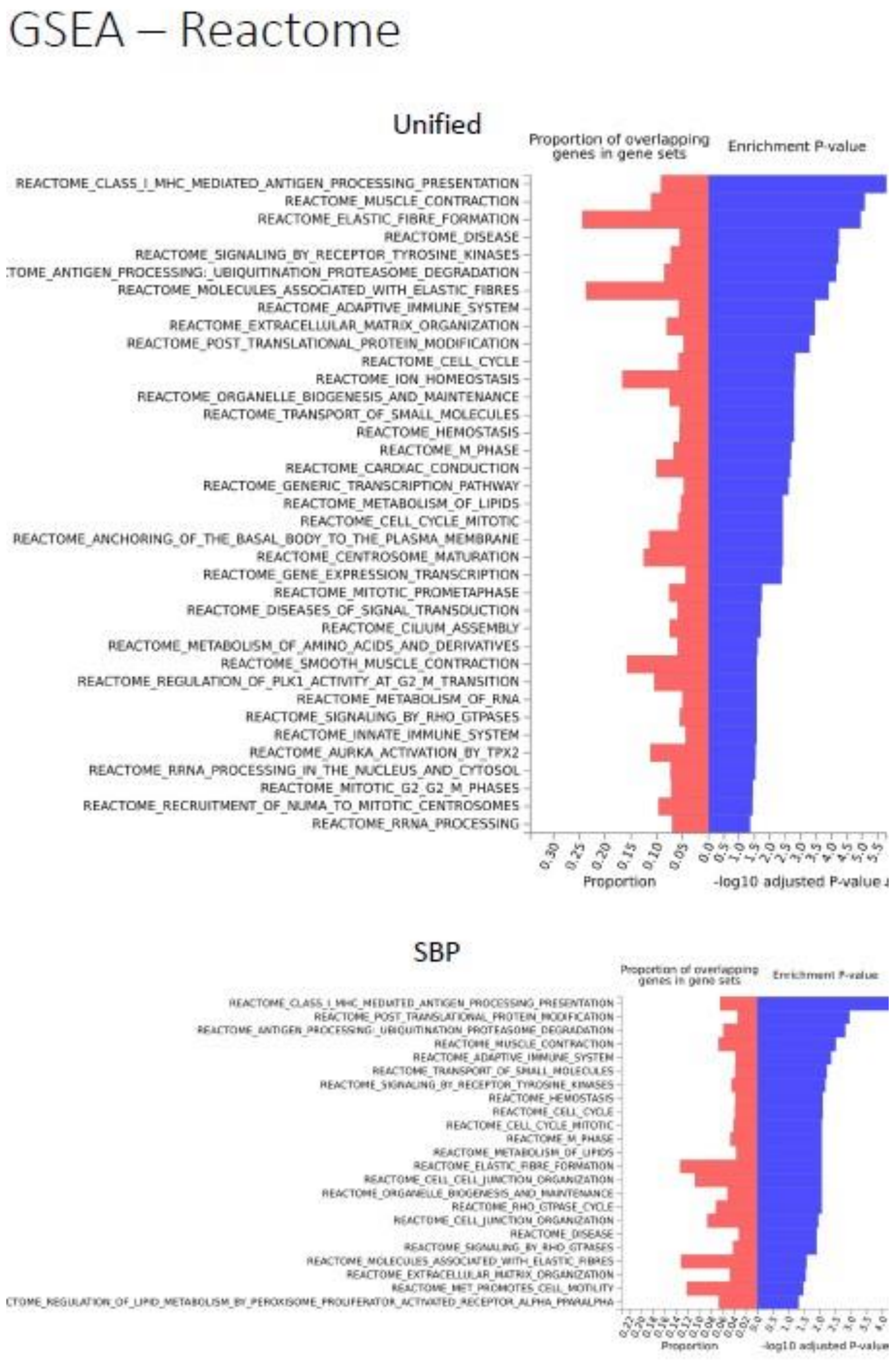

b

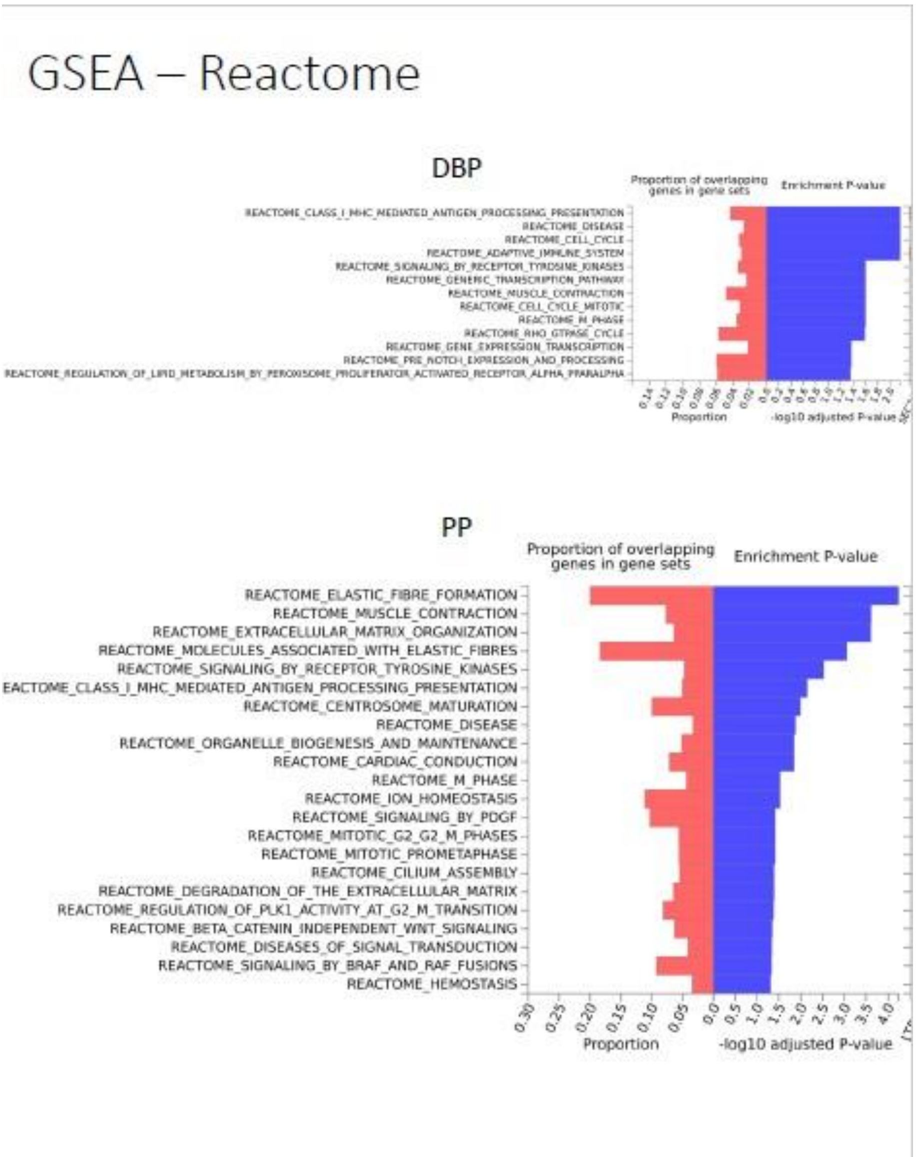

Supplementary Figure 11: Results Output from FUMA Pathway Analyses: GSEA Reactome: (a) Unified Blood Pressure (BP) and Systolic BP (SBP); (b) Diastolic BP (DBP) and Pulse Pressure (PP). P-values of enrichment tests in Reactome pathways are given on the x-axis in logarithmic scale as blue bars, together with the proportion of overlapping genes in gene sets as red bars.

GSEA – KEGG

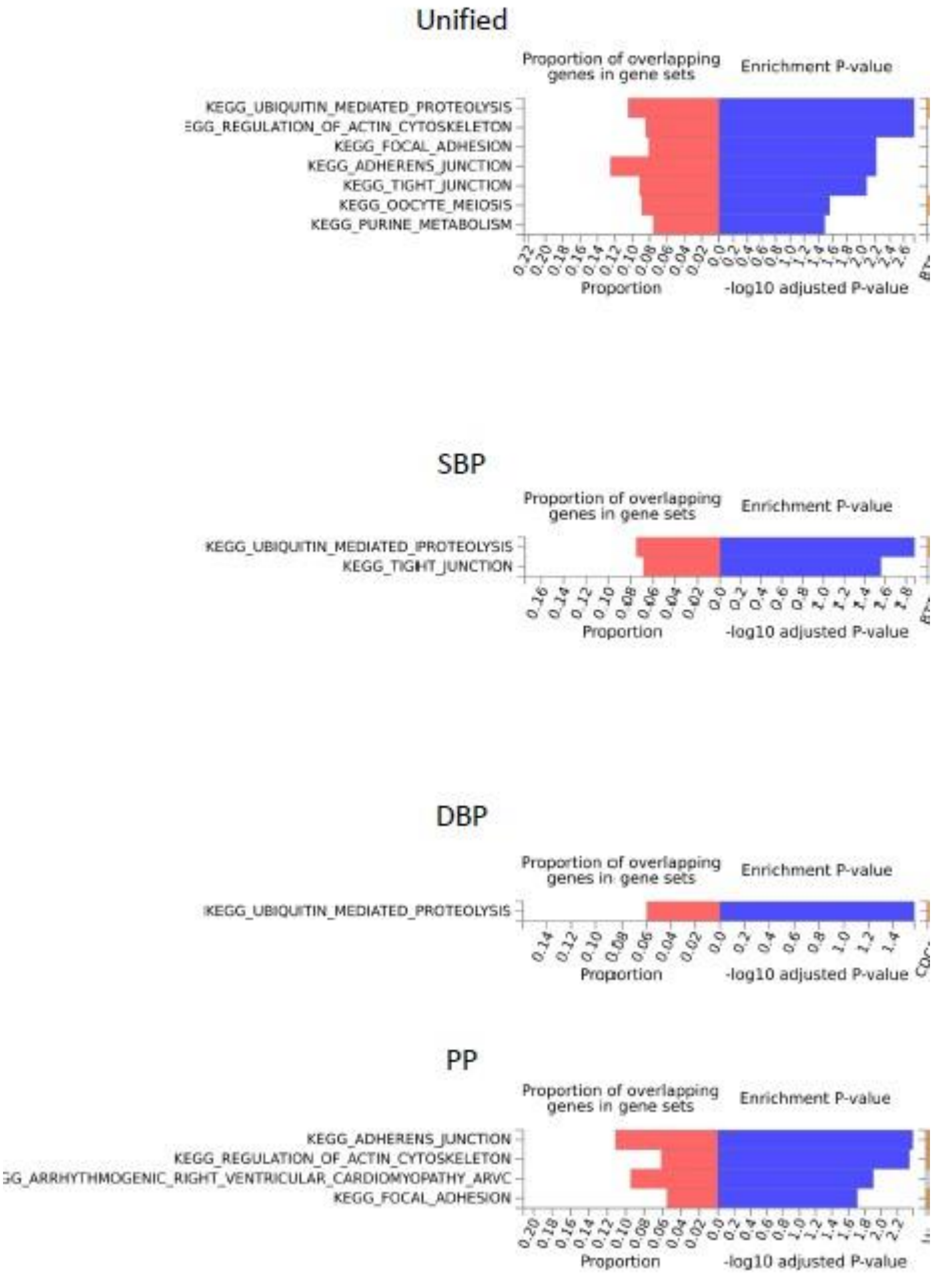

Supplementary Figure 12: Results Output from FUMA Pathway Analyses: KEGG Pathways database: Unified Blood Pressure (BP); Systolic BP (SBP); Diastolic BP (DBP); Pulse Pressure (PP). P-values of enrichment tests in KEGG pathways are given on the x-axis in logarithmic scale as blue bars, together with the proportion of overlapping genes in gene sets as red bars.

GSEA – WikiPathways

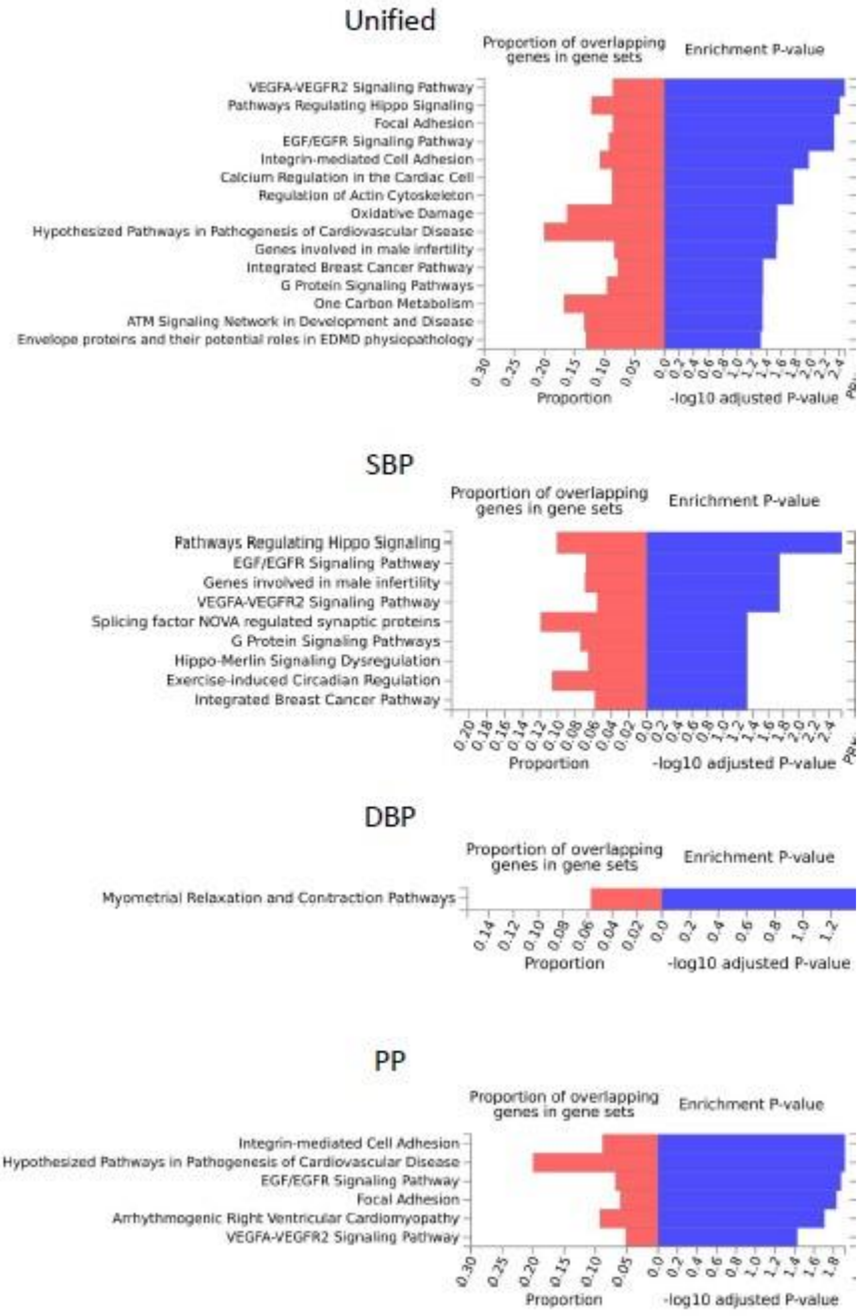

Supplementary Figure 13: Results Output from FUMA Pathway Analyses: Wiki Pathways: Unified Blood Pressure (BP); Systolic BP (SBP); Diastolic BP (DBP); Pulse Pressure (PP). P-values of enrichment tests in WikiPathways are given on the x-axis in logarithmic scale as blue bars, together with the proportion of overlapping genes in gene sets as red bars.

a

BETA plots: meta vs UKB: known / novel / sec

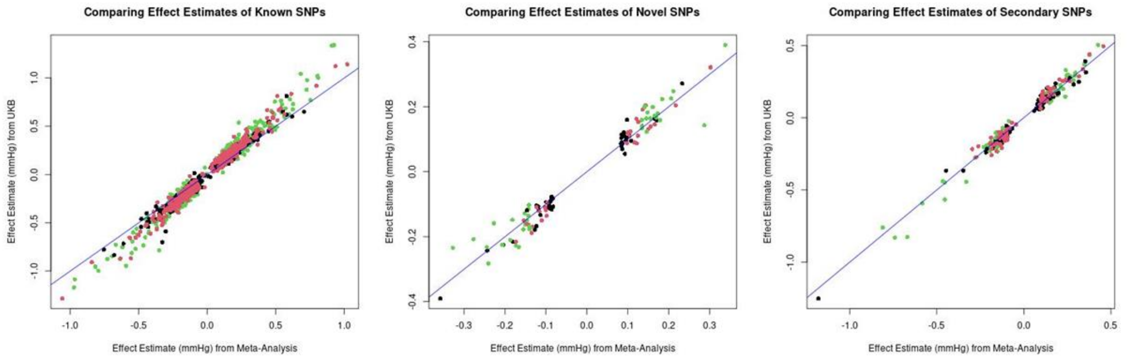

r = 0.987 r = 0.980 r = 0.989

b

BETA plots: meta vs ICBP: known / novel / sec

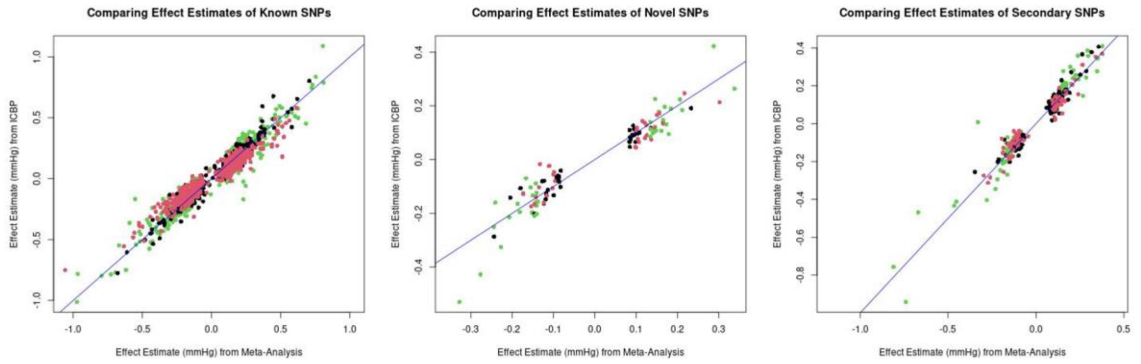

r = 0.964 r = 0.951 r = 0.965

c

BETA plots: meta vs MVP: known / novel / sec

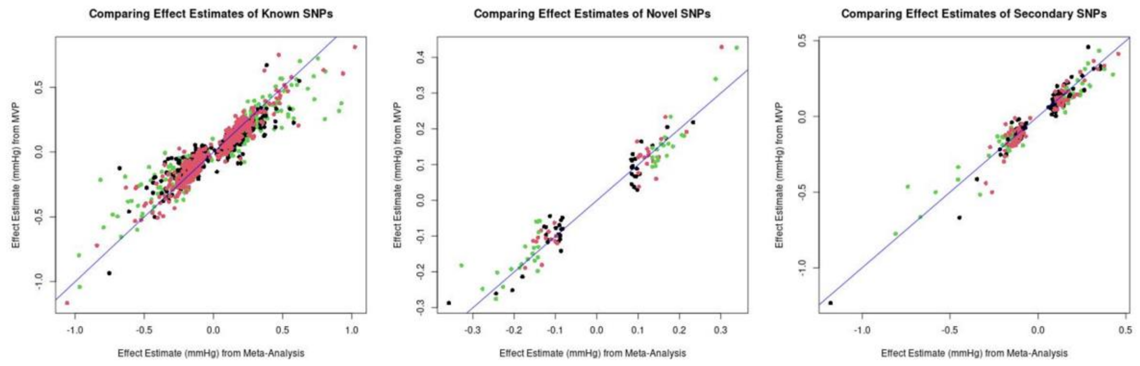

r = 0.935 r = 0.967 r = 0.962

d

BETA plots: meta vs BioVU: known / novel / sec

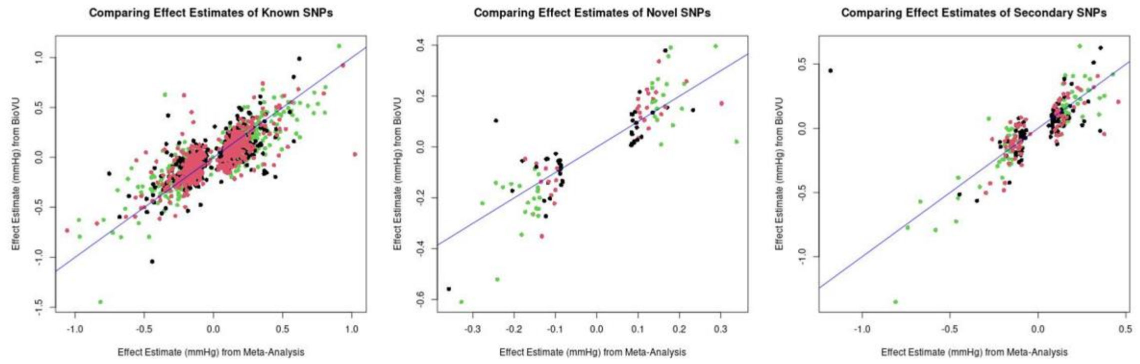

r = 0.796 r = 0.865 r = 0.756

e

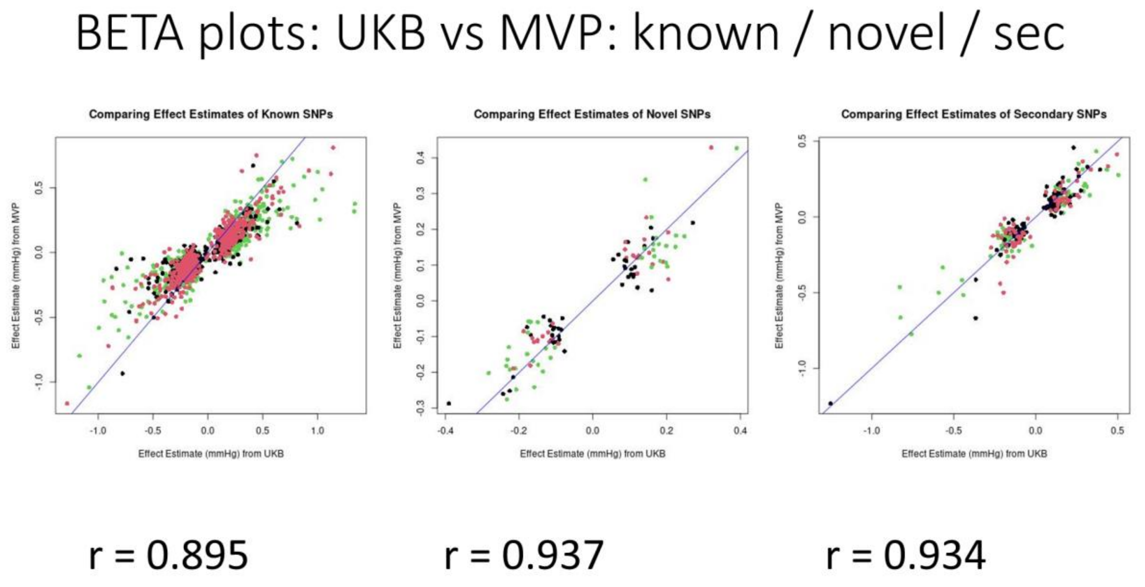

Supplementary Figure 14: Comparison of Beta Effect Estimates for the 113 Novel Loci: (a) meta-analysis vs UKB; (b) meta-analysis vs ICBP; (c) meta-analysis vs MVP; (d) meta-analysis vs BioVU; (e) UKB vs MVP. UKB = UK Biobank; ICBP = International Consortium of Blood Pressure; MVP = Million Veterans Program; BioVU = Biobank Repository of Vanderbilt University; “sec” = secondary variants; r = Pearson correlation coefficient. Points are colour coded to differentiate between SBP, DBP, PP.

SBP:

a

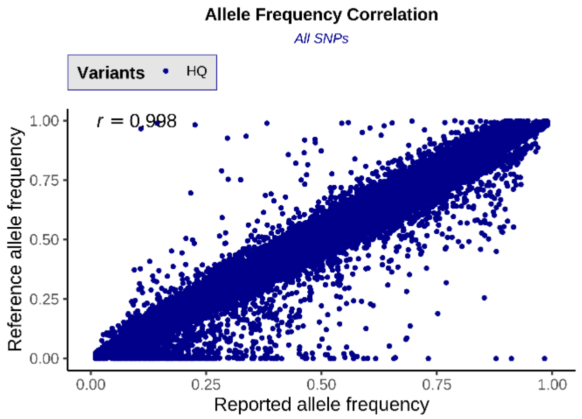

b

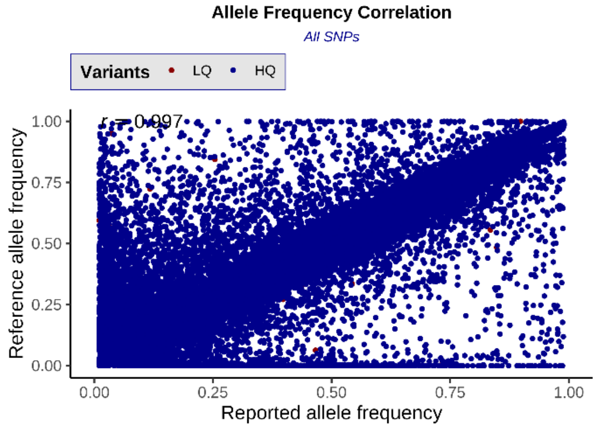

c

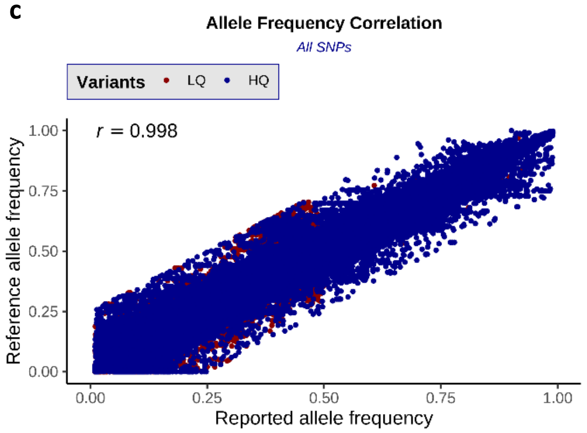

d

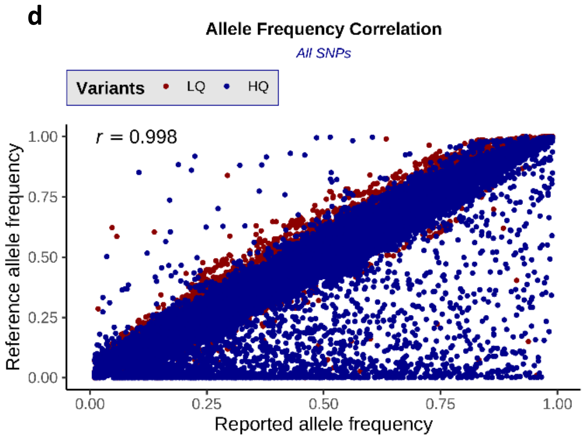

DBP:

a

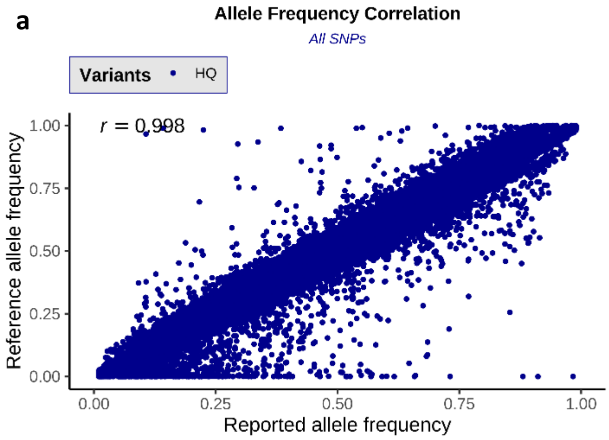

b

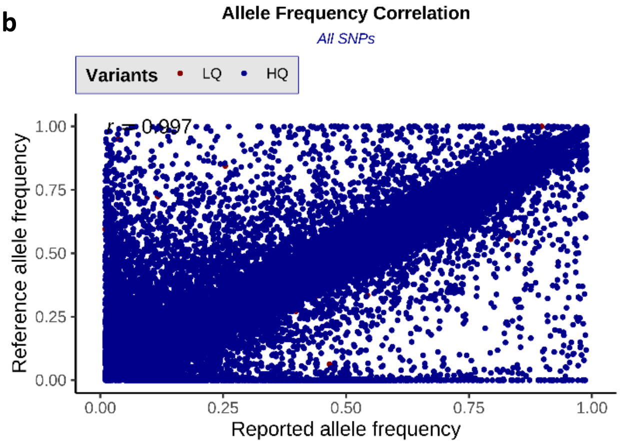

c

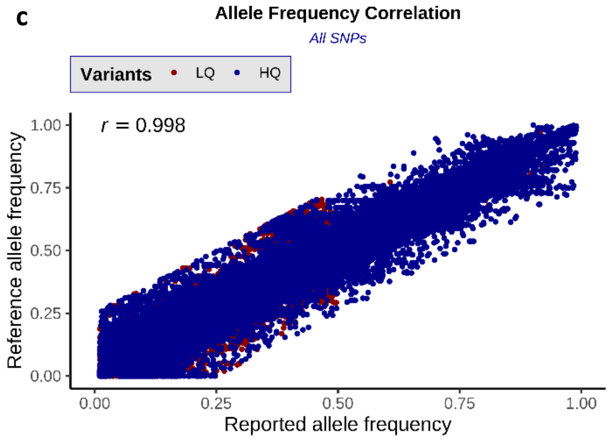

d

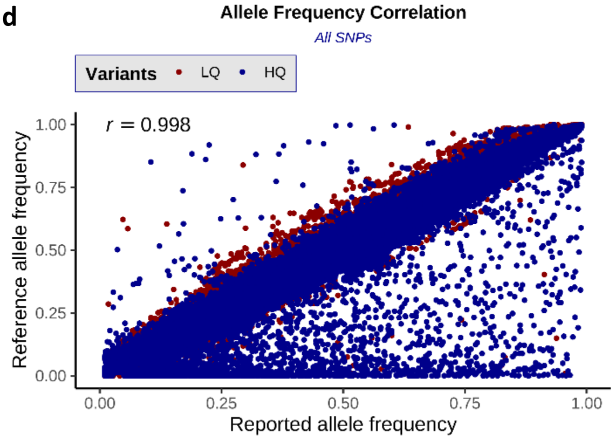

PP:

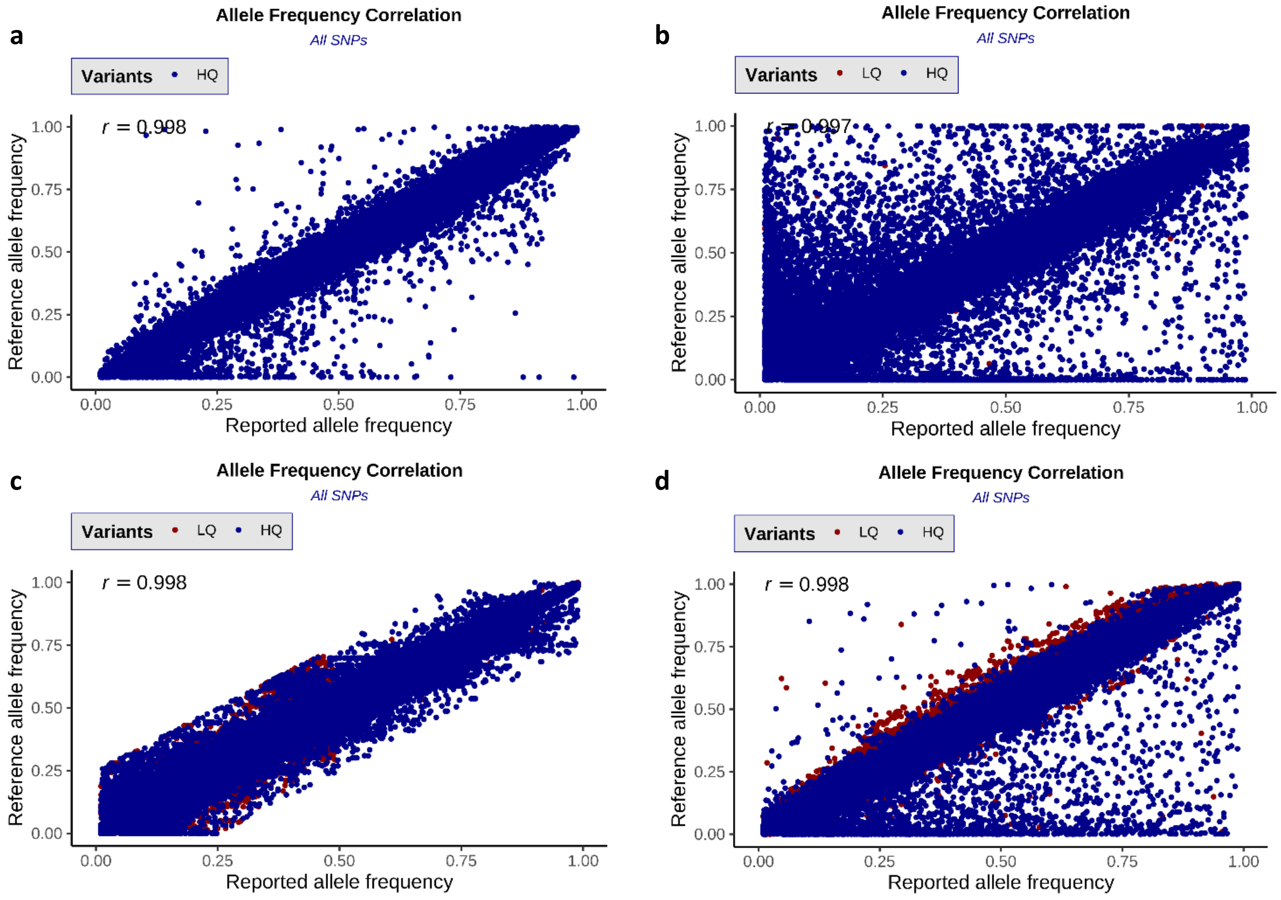

Supplementary Figures 15a-d. Allele frequency correlation of ICBP (a), UKB (b), MVP (c), and BioVU (d) datasets with the 1000 Genomes reference panel of European individuals in study-level QC for systolic-, diastolic-, and pulse pressure (SBP, DBP, and PP respectively). HQ: high-quality SNPs, LQ: low-quality SNPs. Filters for selecting high-quality SNPs include MAF ≥ 0.01, two-sided Hardy–Weinberg p-value $\geq 1 \times 10^{-6}$, and imputation quality > 0.3, where available.

SBP:

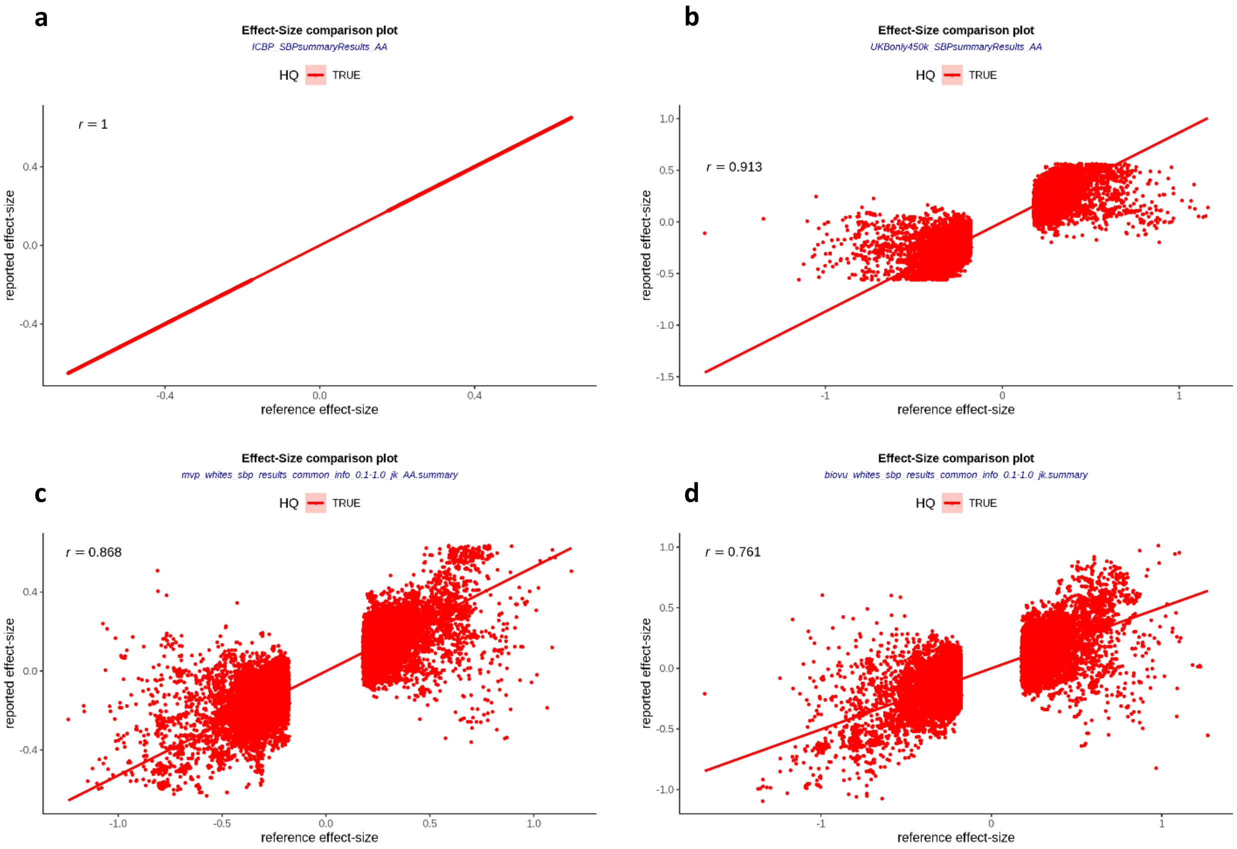
a
Effect-Size comparison plot
ICBP SBPsummaryResults AA
HQ TRUE
r = 1
reported effect-size
reference effect-size
b
Effect-Size comparison plot
UKBonly450k SBPsummaryResults AA
HQ TRUE
r = 0.913
reported effect-size
reference effect-size
c
Effect-Size comparison plot
mvp whites sbp results common info 0.1-1.0 jk AA.summary
HQ TRUE
r = 0.868
reported effect-size
reference effect-size
d
Effect-Size comparison plot
biovu whites sbp results common info 0.1-1.0 jk.summary
HQ TRUE
r = 0.761
reported effect-size
reference effect-size

DBP:

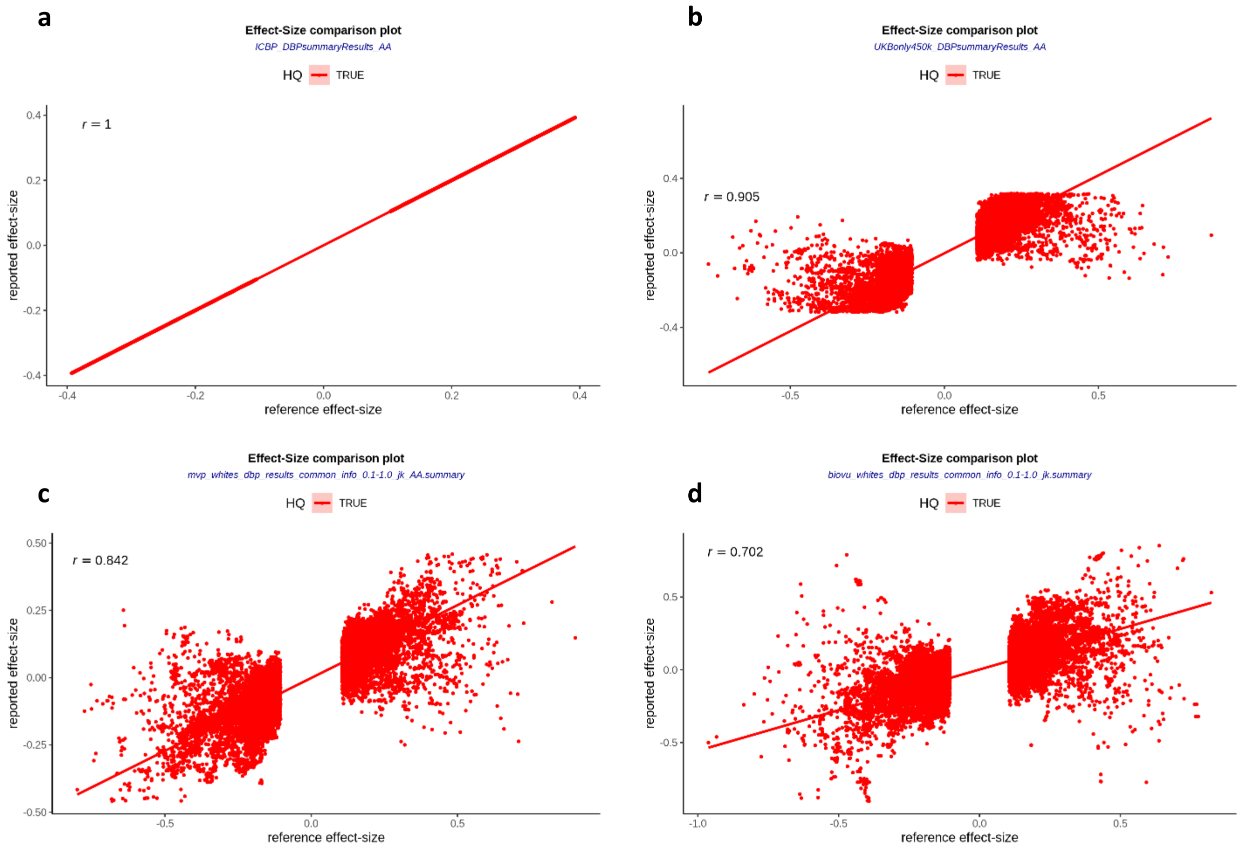
a
Effect-Size comparison plot
ICBP DBPsummaryResults AA
HQ TRUE
r = 1
reported effect-size
reference effect-size
b
Effect-Size comparison plot
UKBonly450k DBPsummaryResults AA
HQ TRUE
r = 0.905
reported effect-size
reference effect-size
c
Effect-Size comparison plot
mvp whites dbp results common info 0.1-1.0 jk AA.summary
HQ TRUE
r = 0.842
reported effect-size
reference effect-size
d
Effect-Size comparison plot
biovu whites dbp results common info 0.1-1.0 jk.summary
HQ TRUE
r = 0.702
reported effect-size
reference effect-size

PP:

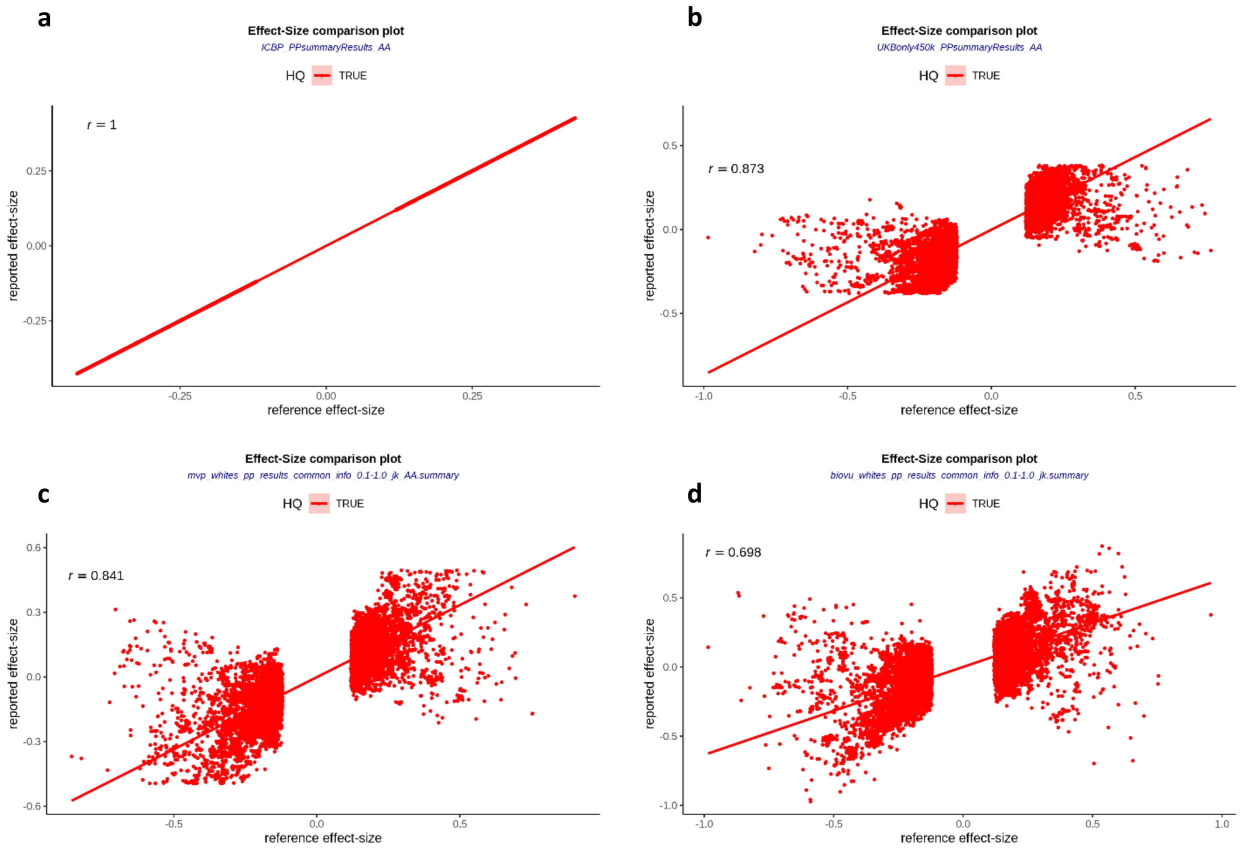

Supplementary Figures 16a-d. Effect size correlation of the ICBP (a), UKB (b), MVP (c), and BioVU (d) GWAS datasets of systolic-, diastolic-, and pulse pressure (SBP, DBP, and PP respectively) with the ICBP GWAS results as reference. HQ: high-quality SNPs, LQ: low-quality SNPs. Filters for selecting high-quality SNPs include MAF ≥ 0.01, two-sided Hardy–Weinberg p-value ≥$1x10^{-6}$, and imputation quality > 0.3, where available.

SBP:

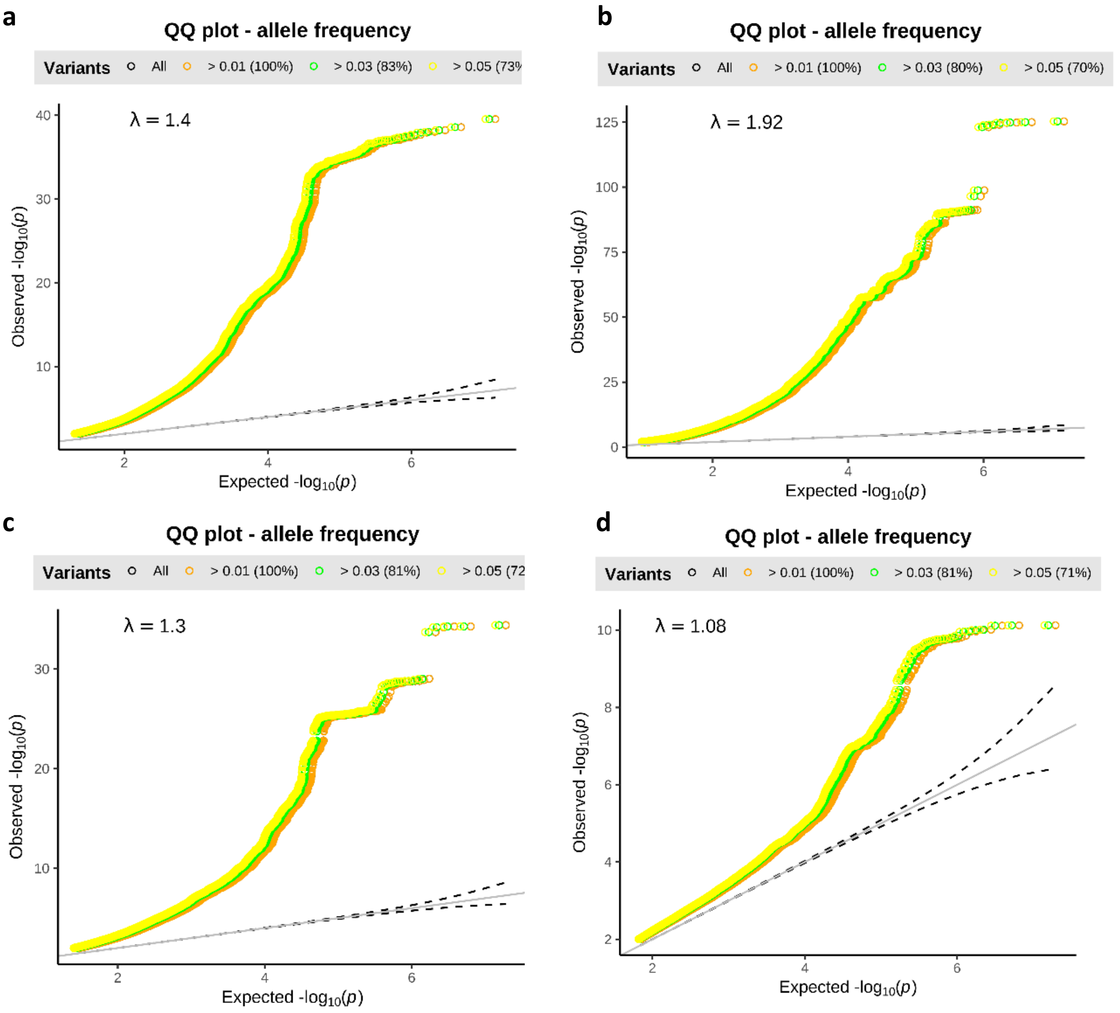
a
QQ plot - allele frequency
Variants All > 0.01 (100%) > 0.03 (83%) > 0.05 (73%
λ = 1.4
Observed -log10(p)
Expected -log10(p)
b
QQ plot - allele frequency
Variants All > 0.01 (100%) > 0.03 (80%) > 0.05 (70%)
λ = 1.92
Observed -log10(p)
Expected -log10(p)
c
QQ plot - allele frequency
Variants All > 0.01 (100%) > 0.03 (81%) > 0.05 (72
λ = 1.3
Observed -log10(p)
Expected -log10(p)
d
QQ plot - allele frequency
Variants All > 0.01 (100%) > 0.03 (81%) > 0.05 (71%)
λ = 1.08
Observed -log10(p)
Expected -log10(p)

a

DBP:

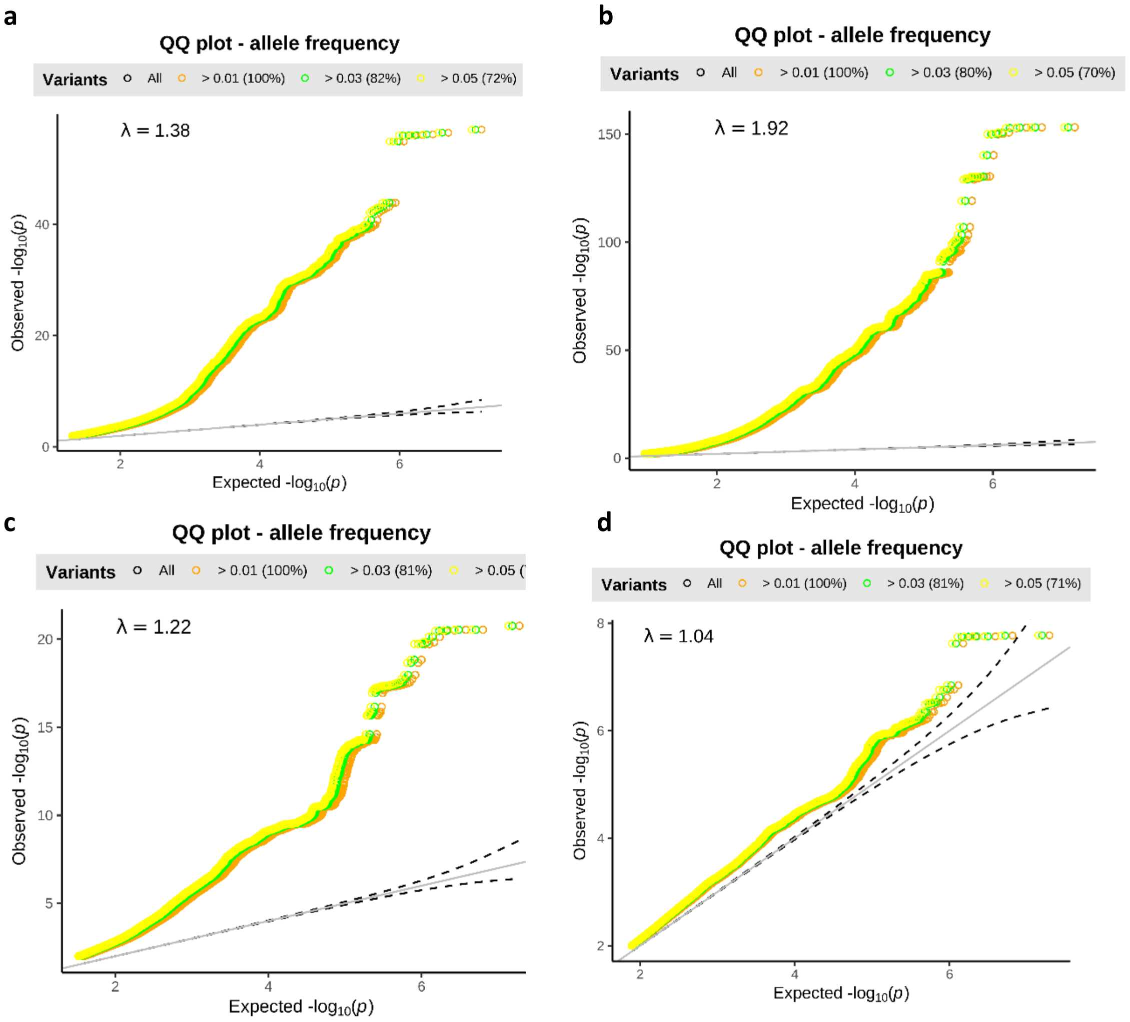
a
QQ plot - allele frequency
Variants All > 0.01 (100%) > 0.03 (82%) > 0.05 (72%)
λ = 1.38
Observed -log10(p)
Expected -log10(p)
b
QQ plot - allele frequency
Variants All > 0.01 (100%) > 0.03 (80%) > 0.05 (70%)
λ = 1.92
Observed -log10(p)
Expected -log10(p)
c
QQ plot - allele frequency
Variants All > 0.01 (100%) > 0.03 (81%) > 0.05 (
λ = 1.22
Observed -log10(p)
Expected -log10(p)
d
QQ plot - allele frequency
Variants All > 0.01 (100%) > 0.03 (81%) > 0.05 (71%)
λ = 1.04
Observed -log10(p)
Expected -log10(p)

PP:

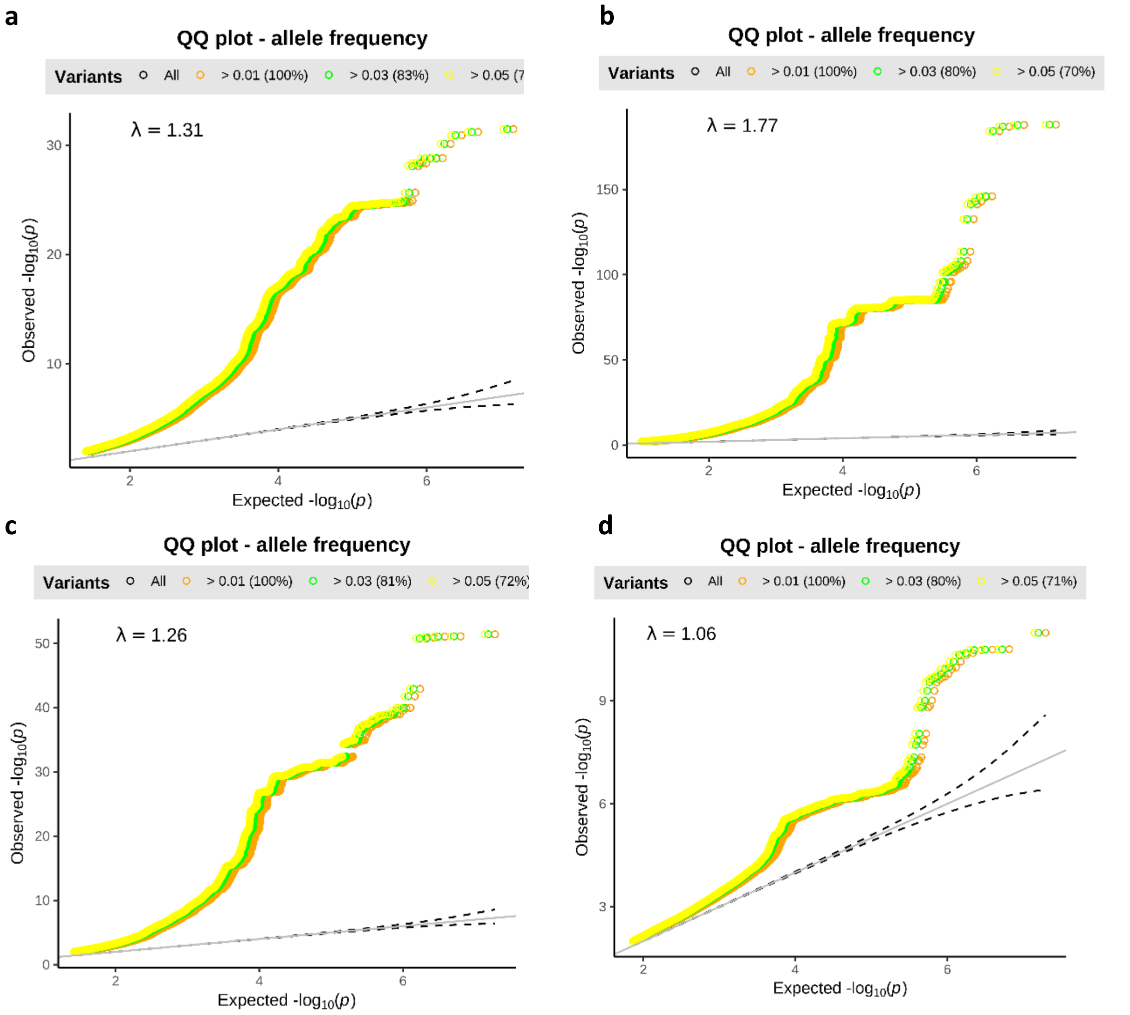

Supplementary Figure 17. QQ plots of the ICBP (a), UKB (b), MVP (c), and BioVU (d) GWAS datasets of systolic-, diastolic-, and pulse pressure (SBP, DBP, and PP, respectively). Observed p-values are from individual linear regression analyses and expected p-values are calculated under the null hypothesis that p-values are uniformly distributed between 0 and 1. QQ plot lines are presented according to different Minor Allele Frequency ranges.

SBP:

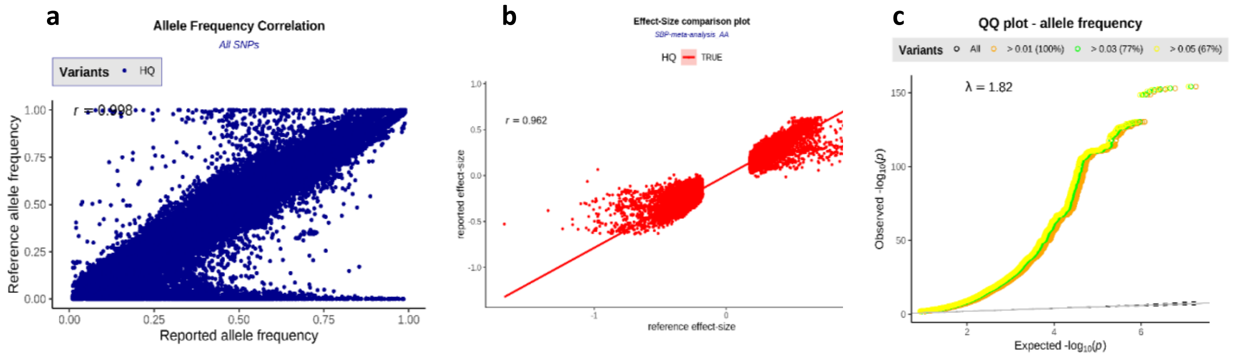

DBP:

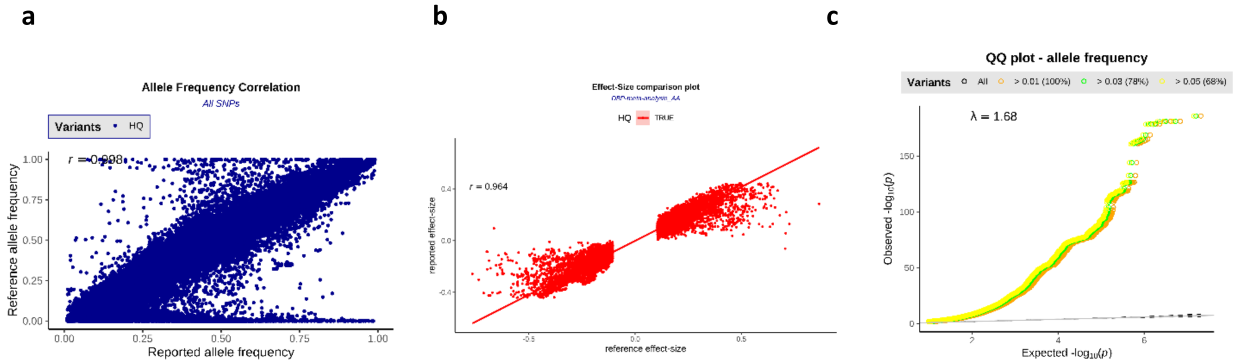

PP:

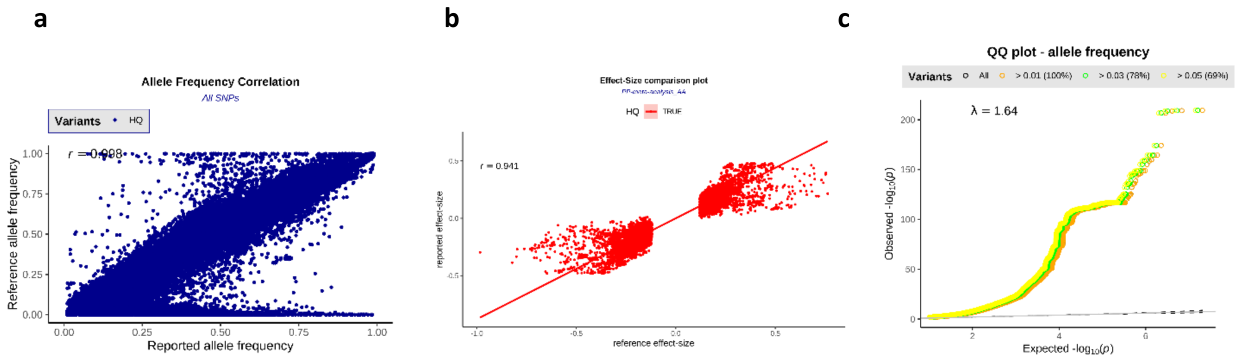

Supplementary Figures 18a-c. Quality checks of the meta-analysis results for systolic-, diastolic-, and pulse pressure (SBP, DBP, and PP respectively). a) Weighted allele frequency correlation with 1KG reference panel; b) correlation of Meta effect sizes with ICBP data, the same reference as for study-level QC; c) QQ plot of the meta-analysis results. Observed p-values are from the inverse variance-weighted GWAS meta-analysis results and expected p-values are calculated under the null hypothesis that p-values are uniformly distributed between 0 and 1. QQ plot lines are presented according to different Minor Allele Frequency ranges.

SBP:

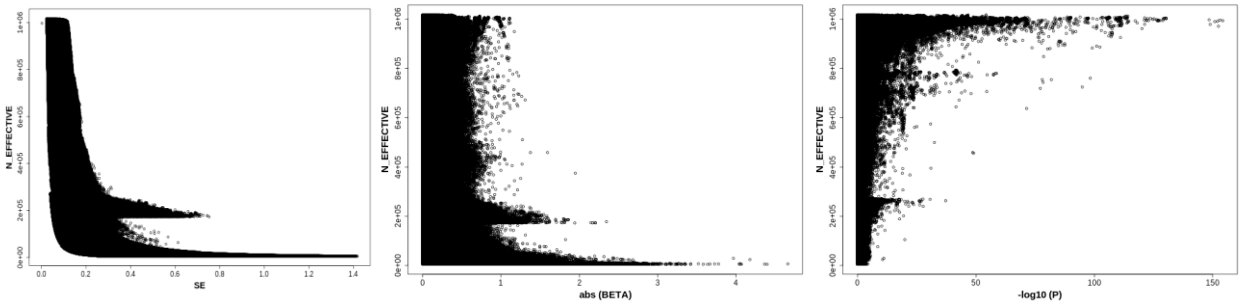

DBP:

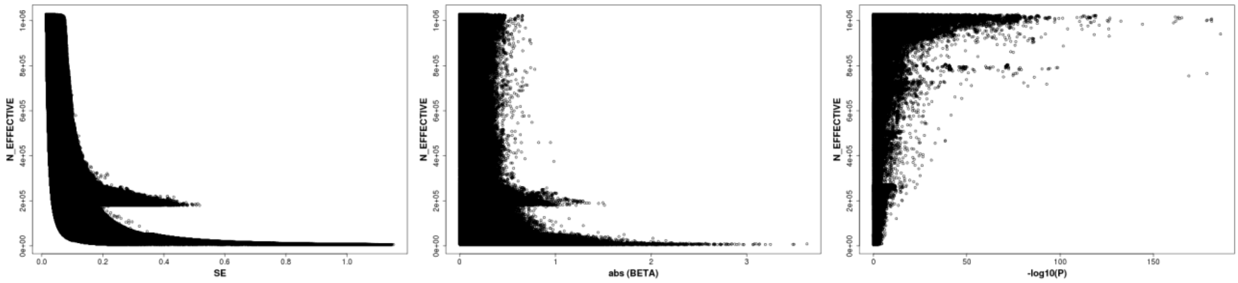

PP:

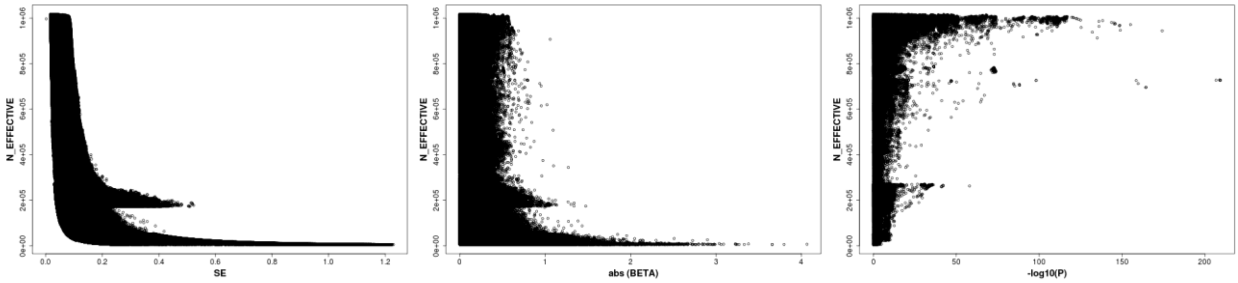

Supplementary Figure 19. Bivariate scatter plots of the key summary statistics in inverse variance-weighted GWAS meta-analysis results of systolic-, diastolic-, and pulse pressure (SBP, DBP, and PP respectively). N_EFFECTIVE = effective sample size; SE = standard error of estimates; abs(BETA) = absolute effect estimate; -log10(P) = minus of meta-GWAS p-values in logarithmic scale.

SBP:

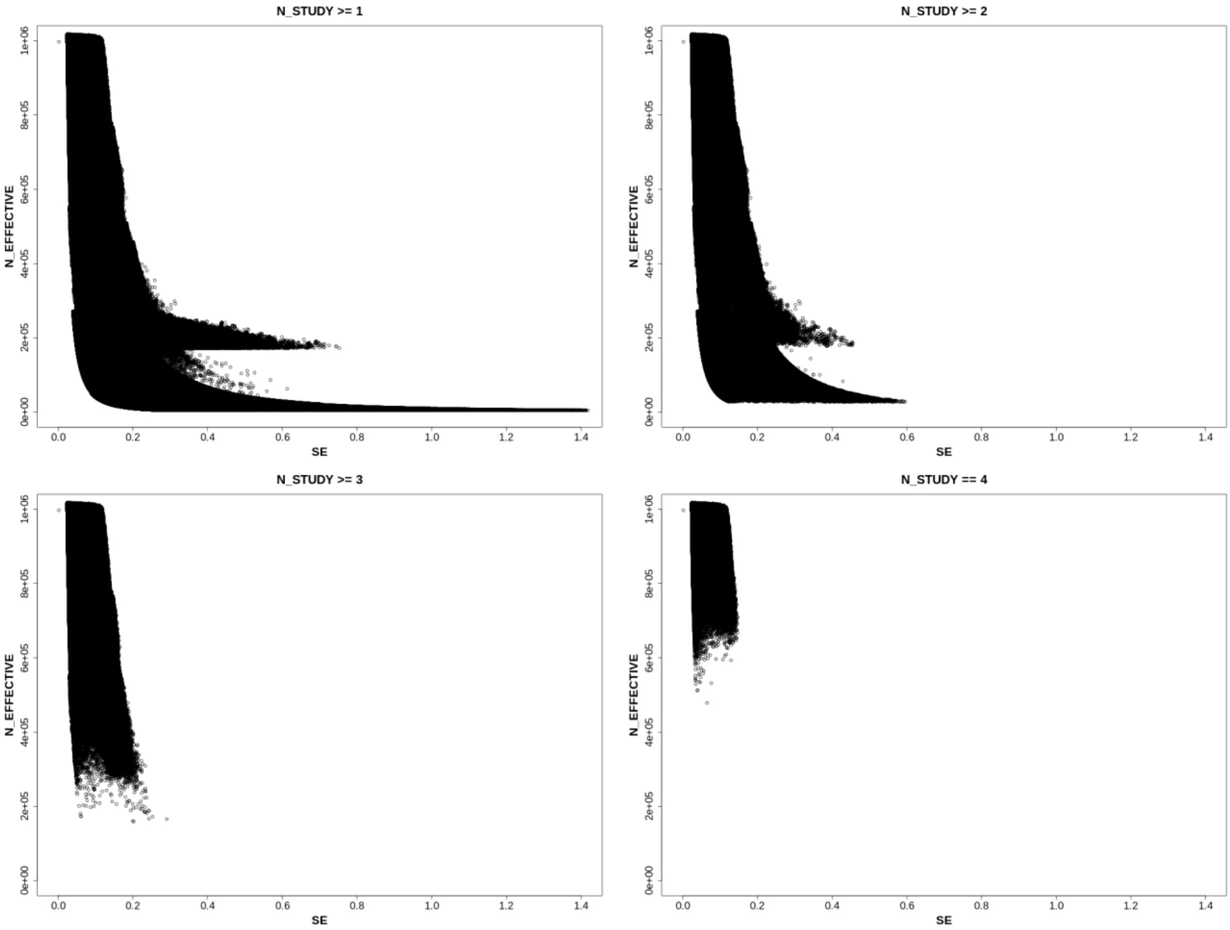

N_STUDY >= 1
N_STUDY >= 2
N_STUDY >= 3
N_STUDY == 4
N_EFFECTIVE
SE

DBP:

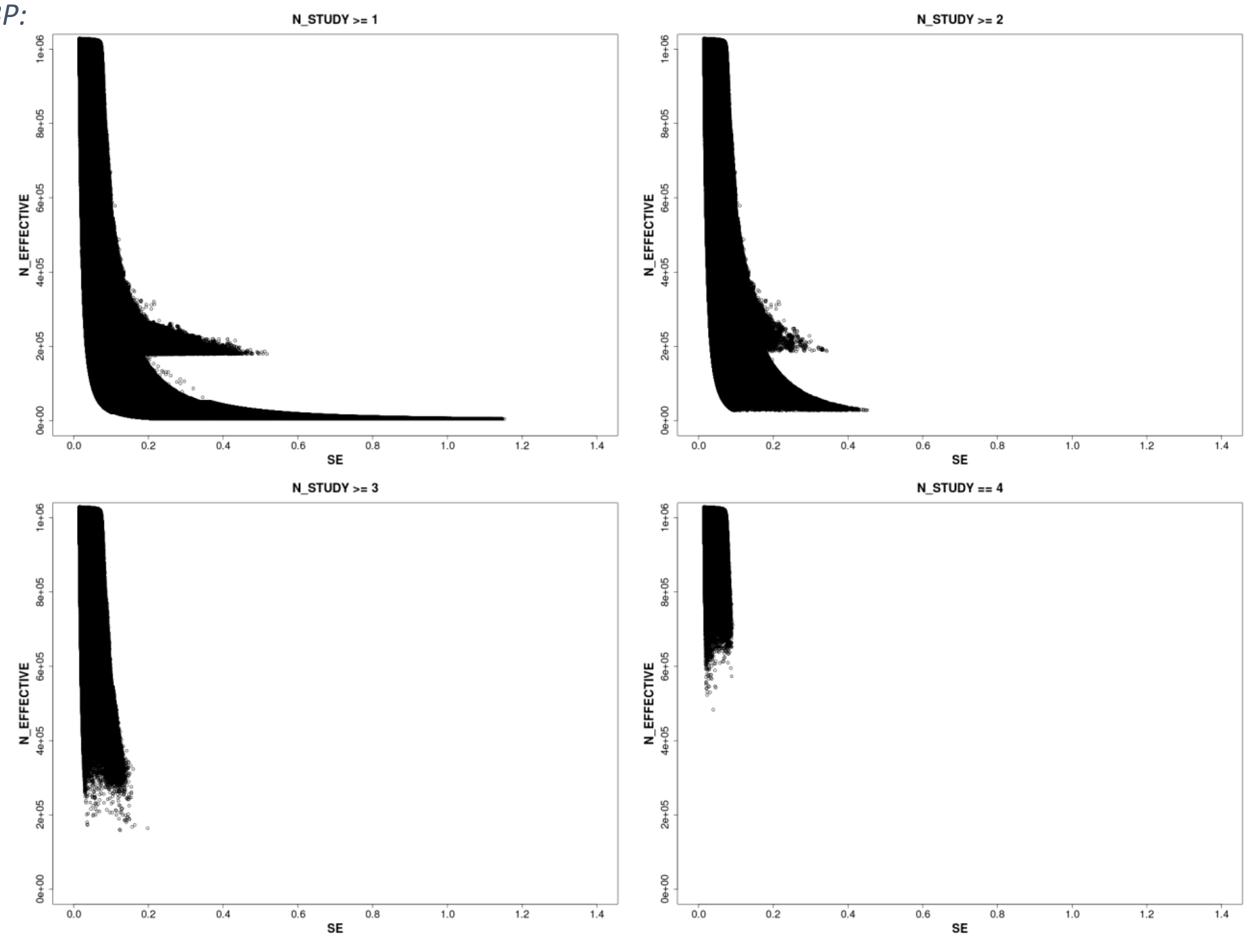

N_STUDY >= 1
N_STUDY >= 2
N_STUDY >= 3
N_STUDY == 4
N_EFFECTIVE
SE

PP:

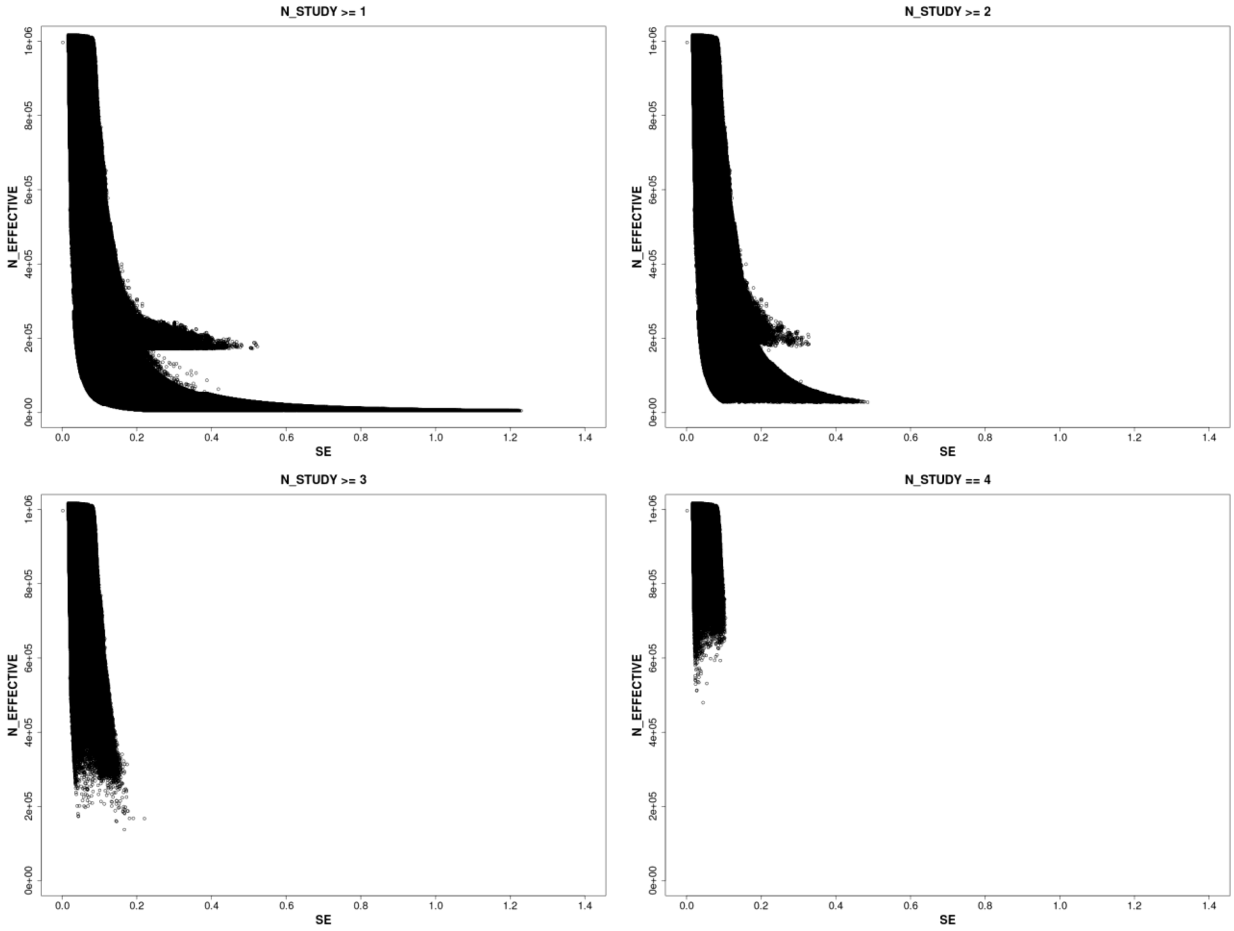

Supplementary Figure 20. Selection of filtering criteria based on N_STUDY (the number of study sub-datasets within the meta-analysis (up to 4) with available data for each variant. Data shown is for meta-analysis results of systolic-, diastolic-, and pulse pressure (SBP, DBP, and PP respectively).

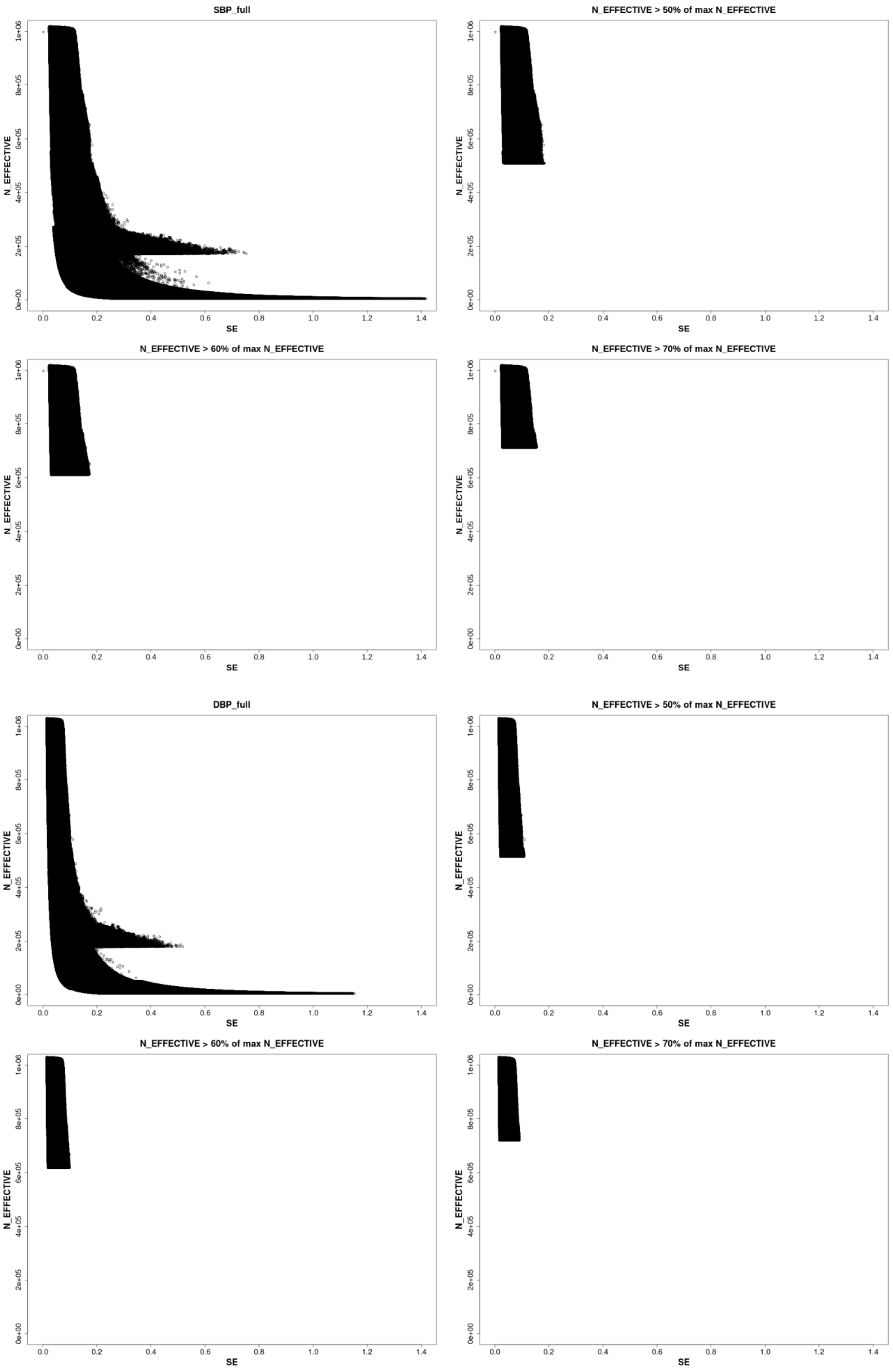
SBP_full
N_EFFECTIVE > 50% of max N_EFFECTIVE
N_EFFECTIVE > 60% of max N_EFFECTIVE
N_EFFECTIVE > 70% of max N_EFFECTIVE
DBP_full
N_EFFECTIVE > 50% of max N_EFFECTIVE
N_EFFECTIVE > 60% of max N_EFFECTIVE
N_EFFECTIVE > 70% of max N_EFFECTIVE
N_EFFECTIVE
SE
0e+00
2e+05
4e+05
6e+05
8e+05
1e+06
0.0
0.2
0.4
0.6
0.8
1.0
1.2
1.4

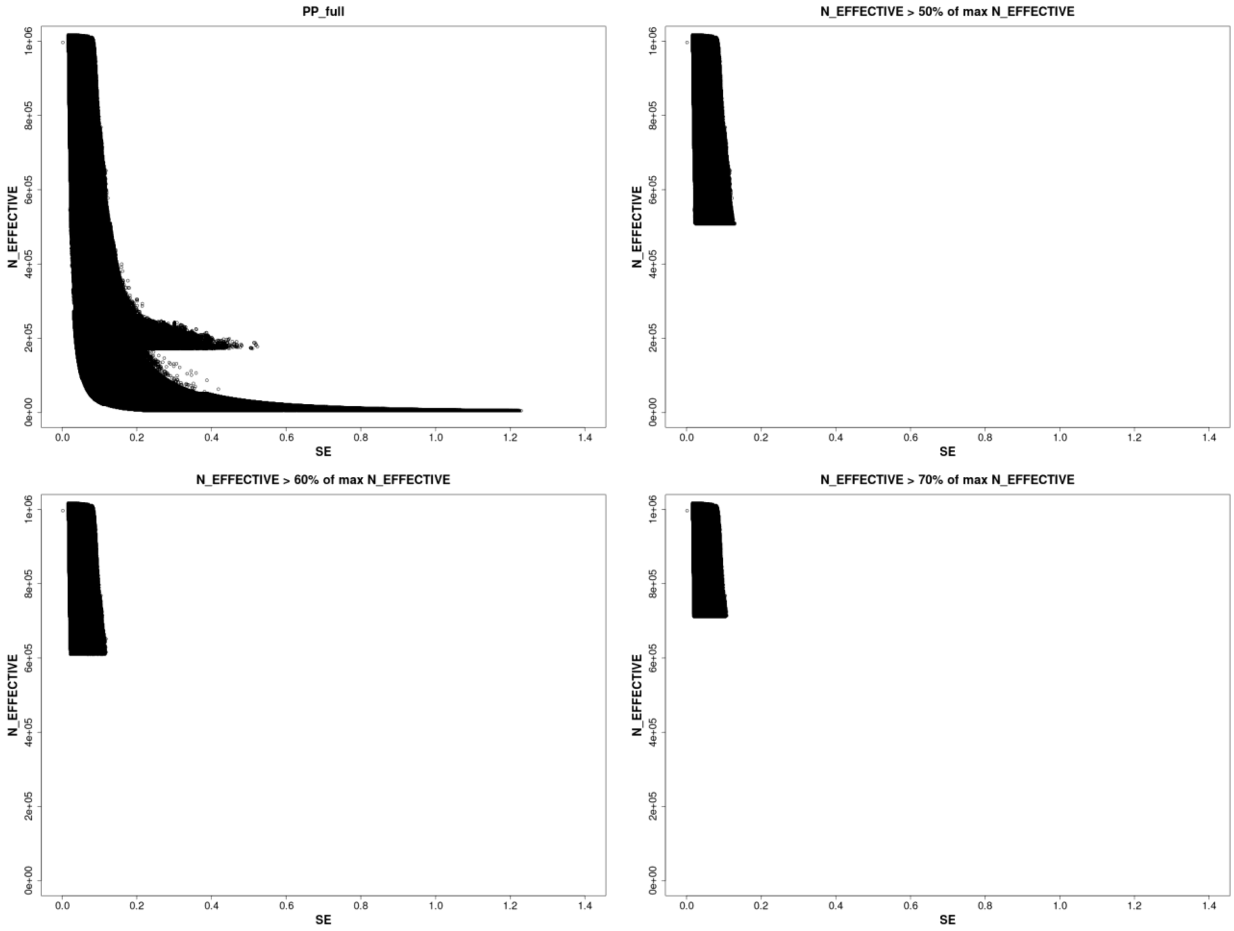

Supplementary Figure 21. Selection of filtering criteria based on N_EFFECTIVE. Data shown is for meta-analysis results of systolic-, diastolic-, and pulse pressure (SBP, DBP, and PP respectively).

SBP:

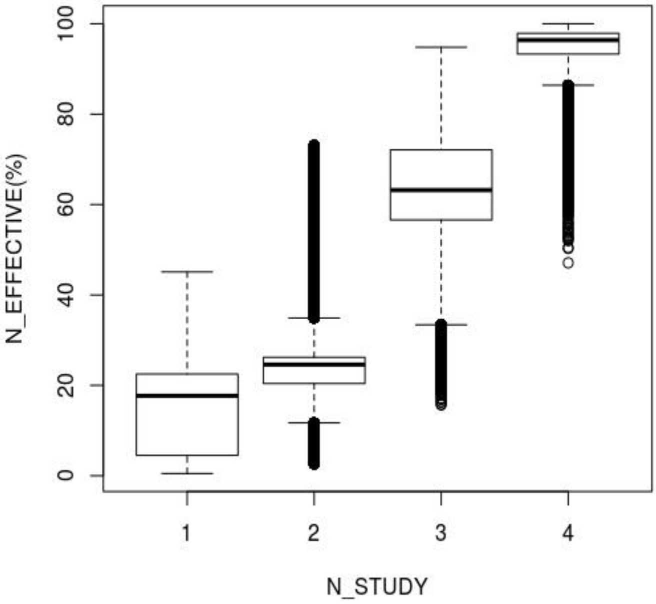

DBP:

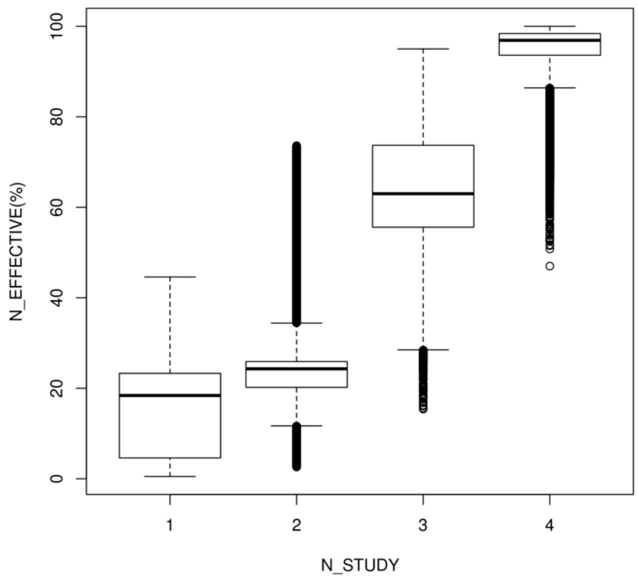

PP:

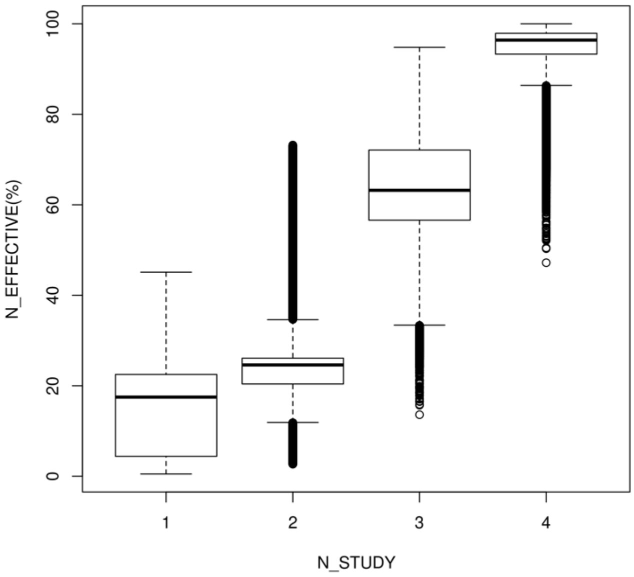

Supplementary Figure 22. The distribution of N_EFFECTIVE/max(N_EFFECTIVE) in percent, for SNPs present in different number of studies (N_STUDY) in meta-analysis results *of* systolic-, diastolic-, and pulse pressure (SBP, DBP, and PP respectively). These studies are ICBP (n=299,024), UKB (n=458,577), BioVU (n= 50,649), and MVP (n= 220,501). Each box represents the interquartile range (IQR) of the data, with the median value shown as a horizontal line in the middle of the box. Whiskers show the 1.5 IQR range, with outlier values drawn as individual points outside.

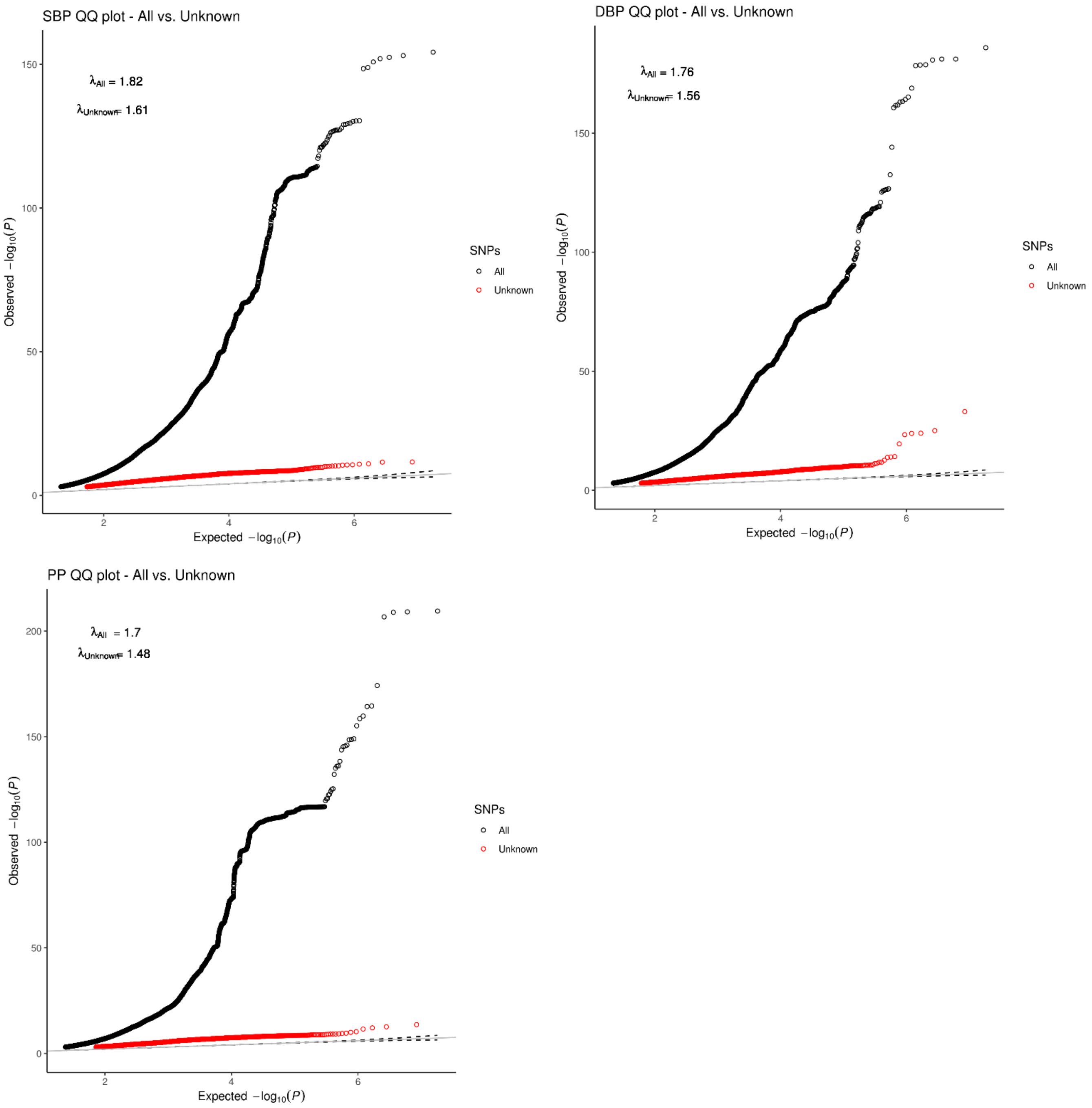

Supplementary Figure 23a-c. QQ plots of meta-analysis results for all variants versus novel variants in the "unknown" subset of the GWAS data, for systolic-, diastolic-, and pulse pressure (SBP, DBP, and PP respectively). Genomic Inflation values λ are provided for both GWAS subsets.
